# Proteomic characterization of *Tityus championi* venom and recombinant expression of its major neurotoxin

**DOI:** 10.1590/1678-9199-JVATITD-2026-0024

**Published:** 2026-09-14

**Authors:** Milena del C. Muñoz-Agames, Marcos H. Salazar, Herlinda Clement, Lorena Hernández-Orihuela, Magdalena Hernández-Ortiz, Sergio Encarnación-Guevara, John Cleghorn, Hildaura Acosta, Gerardo Corzo

**Affiliations:** 1 Universidad de Panama, Research and Information Center for Medicines and Toxic Substances, School of Medicine, Vice-Rectorate for Research and Graduate Studies, Panama City, Panama.; 2 Universidad de Panama, Department of Biochemistry, School of Natural, Exact Sciences and Technology, Panama City, Panama.; 3 Center for Genomic Sciences, National Autonomous University of Mexico, Cuernavaca, Morelos, Mexico.; 4 Department of Molecular Medicine and Bioprocesses, Institute of Biotechnology, National Autonomous University of Mexico, Cuernavaca, Morelos, Mexico.

**Keywords:** Protein, Recombinant expression, Scorpion toxin, Sodium channel, Tityus, Venom

## Abstract

**Background:**

*Tityus championi* is a species endemic to the southern Talamanca Mountain Range, along the border region between Costa Rica and Panama, and has been associated with severe clinical cases. Despite its medical relevance, the composition of its venom remains poorly studied. The present study aimed to characterize the proteomic composition of *T. championi* venom, identify its main toxin families, and recombinantly produce one of its most abundant and lethal toxins for potential use in antivenom development.

**Methods:**

Venom composition was analyzed by tandem mass spectrometry (MS/MS), enabling the identification of venom proteins. Subsequently, one of its primary lethal toxins (Tcham27) was identified and recombinantly expressed.

**Results:**

Proteomic analysis revealed that the most abundant family in the venom corresponded to metalloproteases, with 43 protein groups (17% of the total identifications), which are associated with processes such as hemorrhage, edema, inflammation, hypotension, and necrosis. CIIMET family toxins comprised 27 protein groups (11%). Among ion channel-acting toxins, 18 protein groups (7%) corresponded to sodium channel toxins and 14 (6%) to potassium channel toxins, homologous to components from geographically proximate species such as *Tityus discrepans*, *Tityus* cf. *asthenes*, and *Tityus jaimei*. Other relevant families included cysteine-rich secretory proteins (CRISPs; 6 proteins, 3%), serine proteases (5 proteins, 2%), and lectins (5 proteins, 2%). In addition, low-abundance components such as insulin-like growth factors, nucleotide pyrophosphatases, hyaluronidase, α-amylase, lipolysis-activated toxins, and chitinase were detected, contributing to the functional diversity of the venom.

**Conclusions:**

Proteomic characterization of *T. championi* venom demonstrates that metalloproteases constitute a major protein family alongside neurotoxins. Recombinant production of its most abundant toxic peptide, which is identical to toxins in the venom of geographically proximate *Tityus* species, provides a key tool for developing specific antivenoms.

## Background

Scorpion stings, mainly from the Buthidae family, are of medical and epidemiological relevance [1]. They are the second leading cause of animal-related poisonings in humans, surpassed only by snakebites. Scorpionism, a term used to describe poisoning caused by scorpion stings, constitutes a public health problem in various tropical and subtropical regions [2]. Among scorpion genera, *Tityus* is the most diverse within the order Scorpiones, with over 220 described species distributed throughout the Caribbean, Central, and South America. Several of its species are of medical importance and are responsible for severe envenomation cases and even fatalities reported in countries such as Costa Rica, Panama, Colombia, Ecuador, Venezuela, Guyana, and Brazil [3].

In Panama, scorpions are represented by five genera, and the genus *Tityus* comprises 16 described species. Among them, *Tityus championi* has been associated with severe clinical cases, some requiring treatment in intensive care units [4]. Originally described by Pocock in 1898 [5], this species is endemic to Costa Rica and Panama, with confirmed distribution in the localities of Río Sereno, Cañas Gordas, Alto Quiel, Potrerillos Arriba, Quebrada de Vuelta, Caizán, Breñón, Potrerillos Abajo, and Sortová (Chiriquí Province) [6]. *T. championi* primarily inhabits humid forests and agricultural areas, where it shelters under logs, branches, and leaf litter. Morphologically, it exhibits sexual dimorphism in size and a characteristic coloration pattern [2].

Previous studies on *T. championi* venom have documented its involvement in moderate to severe envenomations, which highlights the necessity of studying its toxic components of its venom for therapeutic and antivenom purposes [7]. In Costa Rica, recent proteomic investigations characterized the venom components of several *Tityus* species from tropical forests, noting similarities with other species of the *Atreus* subgenus and suggesting that the low clinical incidence in that country is more related to ecological factors than to the intrinsic toxicity of the venom [3]. In Panama, on the other hand, there is a reported increase in cases of scorpionism due to *T. championi*, whose venom showed an LD_50_ of 2.9 mg/kg and contains neurotoxins shared with other local species, which underscores the relevance of this group as a target for antivenom development [7]. Moreover, the modern strategy for the functional and structural study of scorpion venom components is the production of recombinant toxins through heterologous expression in *Escherichia coli* [8]. This methodology yields sufficient correctly folded and functional peptides using high-expression vectors and optimized bacterial strains [8]. Furthermore, recombinant scorpion toxins enable detailed studies of receptor interactions, immunogenic analyses, and neutralization assays without the need to collect crude venom [9].

In this work, the venom of *T. championi* was analyzed by MS/MS to identify and characterize its main toxins. The MS/MS analyses also revealed the presence of molecules with potential antimicrobial, antiviral, antiparasitic, hypotensive, immunomodulatory, insecticidal, antitumor, and/or antinociceptive activities [10], which accentuates the biomedical relevance of these compounds. Additionally, the venom of *T. championi* was fractionated using high-performance liquid chromatography (HPLC), and the highest-intensity fractions were also analyzed by mass spectrometry to correlate the toxins with MS/MS data. One of the major and lethal fractions was selected for recombinant expression and compared to other recombinant toxins from Panamanian scorpions, which could allow progress in antivenom production.

## Methods

### Experimental animals

Mice of the CD-1 strain were used for the toxicity evaluation of the HPLC fractions. The project identified by the Vice-Rectorate for Research and Graduate Studies under code VIP-041601-2025-007 was approved by the “Comité de Ética de la Investigación y Bienestar Animal de la Universidad de Panamá (CEIBAUP)” [Research Ethics and Animal Welfare Committee of the University of Panama (CEIBAUP)], as stated in note CEIBA-UP-032-2023 dated September 29, 2023. This approval certifies that the study complies with the ethical standards and procedures for the use of animals in experimentation, in accordance with the principles established in the *Guide for the Care and Use of Laboratory Animals* [11].

### Biological materials

**Figure 1:**
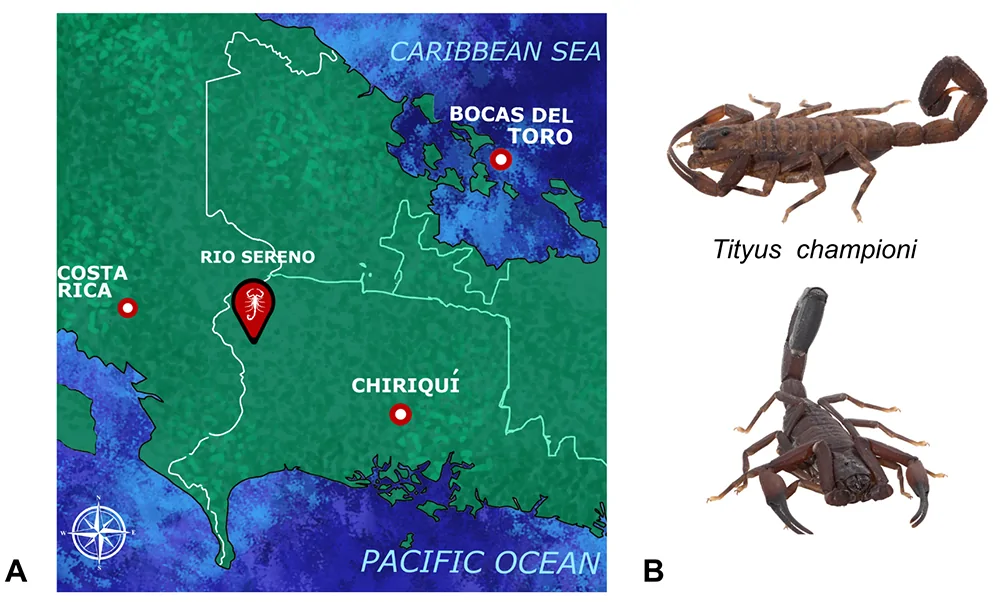
(A) Collection site map of the specimens in Río Sereno, Chiriquí Province, Panama. (B) Adult specimen of *Tityus championi*. Photograph courtesy of D. Constable.

Adult specimens of *T. championi* (n = 33; including both males and females) were collected in the Río Sereno region, Chiriquí Province, Panama, by personnel from the “Centro de Investigación e Información en Medicamentos y Tóxicos de la Universidad de Panamá (CIIMET-UP)” [Center for Research and Information on Medicines and Toxins of the University of Panama (CIIMET-UP)] (Figure 1).

They were maintained individually under controlled conditions that ensured their welfare and optimal health, housed in plastic cages at a constant temperature of 27 °C. Venom was manually extracted using physical stimulation method of the telson, collected on a watch glass lined with paraffin paper, and then recovered in deionized water at room temperature, lyophilized, and stored at -40 °C until use. The total yield of lyophilized venom was 7.6 mg (*ca.* 0.23 mg/individual).

### Fractionation of venom

Lyophilized crude venom (2 mg) was dissolved in 500 μL of type I deionized water, and insoluble material was removed by centrifugation at 14,000 g for 5 min. The soluble fraction was separated using a reversed-phase analytical HPLC system equipped with a C_18_ column (Shimadzu^®^, 250 × 4.6 mm, 5 μm). The column was equilibrated with solvent A (0.1% TFA in water), and solvent B (0.1% TFA in acetonitrile) was used for the elution of venom components. The elution gradient was as follows: the samples were eluted with a linear gradient of acetonitrile 0 to 60% over 60 min (1%/min). The flow rate was 1 mL/min, and eluent absorbance was monitored at 215 nm. Fraction Tcham27 was reconstituted in 20 μL of type I deionized water and loaded onto the system, using the same methodology previously described, to optimize the separation of the two peptides in such a fraction, but with a gradient from 20 to 50% in 60 min (0.5%/min). This procedure enabled maximum resolution and purity of the protein components, facilitating their subsequent analysis.

### Mass spectrometry

For MS/MS, the venom was dissolved in a 50 mM ammonium bicarbonate solution and subsequently subjected to a sequential process of reduction, alkylation, and precipitation. Reduction was performed with 10 mM dithiothreitol (DTT) for 30 min at 56 °C, followed by alkylation with 50 mM iodoacetamide for 30 min at room temperature in the dark. The samples were then digested with trypsin or chymotrypsin at a 1:50 enzyme-to-substrate ratio and incubated for 16 h at 37 °C. The resulting peptides were desalted using reversed-phase chromatography (ZipTip C_18_, Millipore, Billerica, MA, USA), dried, and stored at −80 °C until LC-MS/MS analysis. The digested peptides were resuspended in water with 0.1% formic acid and separated using a Vanquish Neo nanoflow system coupled online to a high-resolution Q-Exactive Plus mass spectrometer (Thermo Fisher Scientific). The mobile phases used were (A) water with 0.1% formic acid and (B) acetonitrile/water (80:20, v/v) with 0.1% formic acid. Two micrograms of sample were injected onto a C_18_ precolumn (PepMap 100, 5 µm, 100 Å, 300 µm × 5 mm) at a maximum pressure of 1,500 bar, using a gradient from 0% B (0 min) to 20% B (60 min) and then to 35% B (90 min). Subsequently, separation was performed on an EASY-Spray ES903 capillary column (PepMap RSLC, C_18_, 2 µm, 100 Å, 75 µm × 50 cm) at 300 nL/min, 50 °C, and a maximum pressure of 1,000 bar.

The mass spectrometer was operated in data-independent acquisition (DIA) mode with positive ionization. Full scans were acquired over an *m/z* range of 390-1,210 at a resolution of 70,000. For DIA analysis, all ions were isolated and fragmented, and the spectra were recorded at a resolution of 35,000. The acquired spectral libraries were processed using the DIA-NN (v2.2.0) software suite, which leverages deep neural networks and interference correction to enable deep proteome coverage in a high-throughput manner [12]. Cysteine residues were carbamidomethylated as a fixed modification, whereas methionine oxidation was permitted as a variable modification, with a maximum of two variable modifications per peptide. The false discovery rate (FDR) threshold was set to 0.01 at both the precursor and protein levels. Mass accuracy optimization was performed automatically using the first run of the dataset. Protein identification employed a highly heuristic protein grouping strategy. The mass spectrometry proteomics data have been deposited to the ProteomeXchange Consortium via the iProX partner repository (project ID IPX0013698000; subproject ID PXD069266) [13].

For HPLC venom fraction mass analysis (MS), the protein fractions (2-3 µg) were reconstituted in 60% acetonitrile containing 0.1% acetic acid. Samples were directly injected into an ion trap mass spectrometer (Thermo Scientific LCQ Fleet, Thermo Inc., San Jose, California), coupled to a Surveyor MS syringe pump delivery system. The eluent flow rate was 10 µL/min, of which only 5% (0.5 µL/min) entered the nanospray ionization source [14]. Spray voltage was set to 1.5 kV, and the capillary temperature was maintained at 150 °C. Fragmentation was performed using collision energies of 25-35 V and normalized collision energies of 35-45% (arbitrary units). Both energy and broadband scanning were activated, and all spectra were acquired in positive ion mode. Data were analyzed using Xcalibur software (Thermo Scientific) on a PC running Windows NT [15]. The average molecular masses obtained have an accuracy of ± 2 Da, due to the limited resolution of the instrument.

### Construction and cloning of *Tcham27*

The *Tcham27* gene (227 bp) was constructed by overlap extension PCR using four overlapping oligonucleotides (Additional file 1). Additionally, a Factor Xa (FXa) protease cleavage site was introduced between the 6×His tag and the mature toxin sequence to allow removal of the tag should it interfere with the biological activity of the peptide. This strategy enabled the production of recombinant peptides derived from the *Tcham27* gene with an N-terminal hexahistidine tag (6×His) to facilitate purification via Ni-NTA affinity chromatography. Once the nucleotide sequence was confirmed, it was digested with *Bam*HI and *Pst*I (New England Biolabs Inc., Ipswich, MA, USA) and cloned into the pQE30 vector (Qiagen, CA, USA) (Additional file 2). Colony PCR was performed to evaluate 12 colonies, all of which displayed an amplification band of the expected size (~500 bp). The colonies expressed a protein of the predicted size (~10 kDa), as confirmed by SDS-PAGE and Western blot analyses (Additional file 3). The plasmid containing the sequence was chosen for subsequent protein expression [16].

### Expression and purification of recombinant proteins

*Escherichia coli* Shuffle^®^ strains (New England Biolabs^®^, MA, USA) were transformed with the corresponding pQE30 plasmid and grown for 16 h on LB (Luria-Bertani) agar plates supplemented with 100 µg/mL ampicillin at 37 °C [17, 18]**.** Individual colonies were selected and inoculated into 50 mL of LB medium with 100 µg/mL ampicillin and cultured at 37 °C for 16 h under constant agitation. Subsequently, 30 mL of this preculture was transferred into 1 L of LB medium containing 100 µg/mL ampicillin. Cultures were incubated at 37 °C under agitation until reaching an optical density at 600 nm (OD_600_) of 0.6-0.8, at which point expression was induced with 0.5 mM isopropyl β-D-1- thiogalactopyranoside (IPTG, Promega, Madison, WI, USA). Expression proceeded for 20 h at 16 °C.

Cells were harvested by centrifugation in an Eppendorf centrifuge (rotor S-4×750) at 4,300 × *g* for 30 min at 4 °C. The cell pellet was resuspended in 50 mM Tris-HCl, pH 8.0, and lysed using an ultrasonic disruptor (BIOBASE, UCD-150). The lysate was centrifuged at 1.6 × 10⁵ × g for 30 min at 4 °C (Eppendorf centrifuge, FA-6×50) to separate the soluble fraction from the insoluble fraction corresponding to inclusion bodies (IBs). The IBs were subsequently solubilized in 6 M guanidine hydrochloride (GndHCl) in 50 mM Tris-HCl, pH 8.0, for 16 h [18].

Recombinant toxins recovered from IBs were purified by nickel affinity chromatography using an automated ÄKTA pure M liquid chromatography system (Cytiva), which allows precise programming and control of all purification steps (column preparation, sample application, washing, and elution) and real-time monitoring of critical parameters such as absorbance, pH, and conductivity, facilitating the identification of fractions containing the protein of interest. Affinity purification was performed on a 5 mL HiTrap^™^ Talon^®^ Crude column (Cytiva), consisting of a metal ion-charged resin matrix that specifically interacts with the His-tag of recombinant toxins.

The column was initially equilibrated with 12 mL of solution A (6 M GndHCl, 50 mM Tris-HCl, pH 8.0) at a flow rate of 1.0 mL/min, conditioning the resin and establishing optimal protein-binding conditions. The solubilized cell lysate was then applied in the same buffer, promoting specific interaction between the His-tag and the column’s metal ions. The column was washed with 20 mL of solution A at 4.0 mL/min to remove nonspecifically bound proteins and other impurities. Elution of the target protein was carried out using a linear gradient from 0 to 100% of solution B (6 M GndHCl, 300 mM imidazole, pH 8.0) over 30 mL at 3.0 mL/min. The gradual increase in imidazole concentration displaced bound proteins from the matrix, which were collected in successive fractions monitored at 215 nm to identify those containing the recombinant toxin. Under these conditions, dilution from 6 M to 2 M GndHCl in 50 mM Tris-HCl, pH 8.0, allowed the peptide to refold spontaneously.

Fractions were further purified by high-resolution RP-HPLC using a Shimadzu^®^ C18 column (250 × 4.6 mm, 5 µm) with solvent A (0.1 % TFA in water) and solvent B (0.1 % TFA in acetonitrile), applying a linear gradient from 15 to 45% B over 65 min. Fractions were collected manually by monitoring absorbance at 215 nm [19].

Molecular mass was confirmed by mass spectrometry to verify the presence of the recombinant toxin (Thermo Scientific LCQ Fleet, Thermo Inc., San Jose, California).

### Circular dichroism

The secondary structure of the recombinant proteins rTapp2, rTcham27-4, and rTcham27-5 was determined by circular dichroism (CD) spectroscopy. Spectra of the recombinant proteins were recorded within a wavelength range from 190 to 260 nm at room temperature in 1 mm path-length quartz cells using a Jasco J-710 spectropolarimeter (Jasco, Japan). Data were acquired at 1 nm intervals at a scan rate of 50 nm/min. Each protein was prepared to a concentration of 100 µg/mL in 60% trifluoroethanol, which increases secondary structure by sequestering water from the sampling solution. The CD values represent the mean of three recordings. The spectra were deconvoluted employing the Beta Structure Selection (BeStSel) web server (http://bestsel.elte.hu/index.php). 

### Evaluation of the toxic activity of rTcham27

To evaluate the biological activity of the recombinant toxin, fractions obtained by RP-HPLC purification were used. Following the guidelines of our institute’s Animal Welfare Committee and to minimize the number of animals used, male CD-1 mice (18-20 g) were evaluated via intracranial injection. The volume required to administer a dose of 10 µg per animal was calculated. Injections were performed intracranially near the central region of the parietal bone, directed laterally toward the midline suture to avoid damage to the transverse venous sinus [20]. Animals were observed for 24 h to assess biological effects, which were evaluated using the Hippocratic screening technique to provide an initial qualitative toxicological profile [1]. Behavioral and neurotoxic parameters evaluated included motor activity, grooming behavior, forced abdominal respiration, Straub sign, piloerection, and forelimb and hindlimb paralysis. These parameters were recorded qualitatively based on their presence or absence during the 24 h observation period.

The intracranial LD_50_ was determined using a maximum of 12 mice (six doses, two mice per dose). Initial doses of 1 and 10 µg per mouse for nTcham27 and rTcham27, respectively, were used, based on previous data, after which lower doses were administered to establish the LD_50_.

### Statistical analysis

Statistical analyses were performed using GraphPad Prism version 9 software (San Diego, CA, USA).

## Results

### Mass spectrometry analysis of *Tityus championi* venom

Proteomic analysis of *Tityus championi* venom by MS/MS enabled the identification of 339 sequences. All files of the identified sequences, along with their corresponding scores and intensities, are available in the iProX repository (project ID IPX0013698000; subproject ID PXD069266). Additional file 4 and Additional file 5 provide the complete set of identified protein sequences and annotation details.

**Figure 2:**
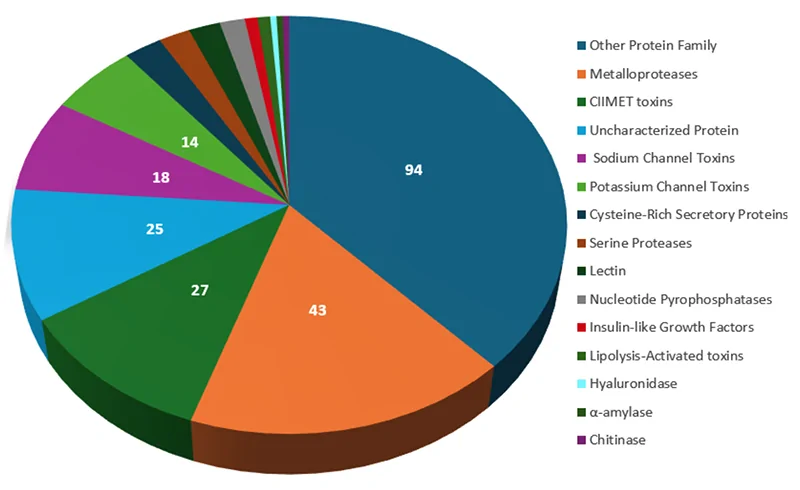
Number of proteins identified in the *Tityus championi* venom proteome. They were classified into protein families through comparative analysis using BLASTp and InterProScan. The numbers represent absolute counts per family.

Analysis with DIA-NN enabled the identification of 248 protein groups, classified into 51 distinct protein families, based on their homology (BLASTp), reported biological activities, and information from the VenomZone database (https://venomzone.expasy.org) (Figure 2, Additional file 5). The most abundant family corresponded to metalloproteases, with 43 protein groups (17%). CIIMET toxins were represented by 27 protein groups (11%), showing homology with a hypothetical protein from *Tityus discrepans* (GenBank: CAY61861.1). Regarding ion channel-acting toxins, 18 sodium channel toxins (7%) and 14 potassium channel toxins (6%) were identified.

Other relevant families included cysteine-rich secretory proteins (CRISP; 6 proteins, 3%), serine proteases (5 proteins, 2%), and lectins (5 proteins, 2%). In addition, several low-abundance components were detected, including insulin-like growth factors, nucleotide pyrophosphatases, hyaluronidase, α-amylase, lipolysis-activated toxins, and chitinase, each represented by one or two sequences. The category of other protein families comprised 94 protein groups (38%), including components such as cytoskeletal elements (actins), lipid metabolism enzymes (phospholipases and lipoxygenases), and defense and regulatory proteins, such as copper-zinc superoxide dismutase and lysosomal aspartic proteases.

Finally, the uncharacterized protein family comprised 25 proteins (10% of total identifications), mostly corresponding to uncharacterized or hypothetical proteins, mainly from *Centruroides sculpturatus*.

### Chromatographic analysis of *Tityus championi* venom

**Figure 3:**
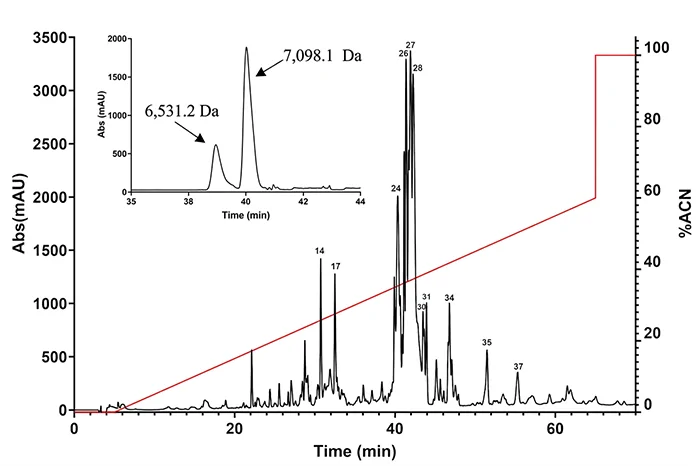
Complete chromatogram of crude *Tityus championi* venom obtained by RP-HPLC. Fractions were collected from crude *Tityus championi* venom (2 mg) using high-resolution reversed-phase HPLC on a C_18_ column (4.6 × 250 mm, 5 µm particle size). Elution was performed using a gradient with solvent A (0.1% TFA in water) and solvent B (0.1% TFA in acetonitrile) at a flow rate of 1 mL/min. Inset: HPLC chromatogram of fraction Tcham27 (0.07 mg) using the same C_18_ column, where protein elution was performed using a gradient from 20 to 50% B over 60 min (0.5%/min) at a flow rate of 1 mL/min.

In addition to the MS/MS analysis, *T. championi* venom was separated by reversed-phase HPLC on a C18 column, yielding 37 fractions to identify mammalian toxic peptides (Figure 3). Eleven fractions were selected based on their peak intensity at 215 nm and analyzed by mass spectrometry to correlate them with the MS/MS results.

The venom fractions contained more than one protein, with molecular masses ranging from 3 to 11 kDa (Table 1). The fraction identified as Tcham24 (2,674.2 Da) closely matched the mass of fraction Tch4 (2,675.2 Da) previously reported by Salazar et al. [7] and Díaz et al. [3] for *T. championi* from Panama and Costa Rica, respectively. The molecular masses in fractions Tcham17 (3,969.6 Da) and Tcham35 (7,624.7 Da) showed similarity to those of 3,971.7 and 7,626.4 Da reported by Díaz et al. [3]. Likewise, fractions Tcham14, Tcham24, Tcham26, Tcham27, Tcham28, Tcham30, Tcham31, and Tcham35 exhibited molecular masses comparable to fractions Tch1, Tch2, Tch4, Tch7, Tch9, Tch11, and Tch15 described by Salazar et al. [7].

**Table 1:** *Tityus championi* venom fractions, concentration, molecular masses, and toxicity.

Fraction	Concentration (µg)	Molecular mass (Da)	Toxicity in mice i.c. (1 µg)
Tcham14	2.6	3871.8, 3908.8	Lethal
Tcham17	12.8	3561.4, 3969.6	NT
Tcham24	12.2	2604.2, **2674.2**	Lethal
Tcham26	40.4	7329.2, 7143.1	Lethal
Tcham27	73.0	6531.2, **7098.1**	Lethal
Tcham28	71.2	6953.1, 10699.9	Lethal
Tcham30	4.8	7168.2, 7380.2	Lethal
Tcham31	1.4	7168.2, 7380.2	Lethal
Tcham34	12.0	7380.2, 11114.2	Lethal
Tcham35	1.0	7169.4, 7624.7	Lethal
Tcham37	3.4	No data found	ND

Fraction: *Tityus championi* venom fractions. Collected peaks were analyzed by mass spectrometry to determine their molecular masses. The toxicity data were obtained from Salazar et al. [7], where the toxicity of homologous fractions of the *Tityus championi* venom was evaluated by intracranial (i.c.) injection in a murine model. NT: non-toxic after 2 h, ND: not determined. Protein concentration was estimated by absorbance at 280 nm, assuming one absorbance unit equals 1 mg/mL.

**Figure 4:**
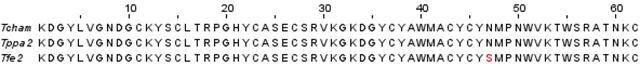
Toxin Tcham27 (*Tityus championi*); toxin Tppa2 (*Tityus pachyurus/Tityus jaimei*); toxin Tfe2 (*Tityus festae*). The amino acid in red indicates a substitution of the corresponding serine in the Tfe2 sequence with asparagine in Tcham27 and Tppa2.

Among these, Tcham27 contained two peptides, with one being the most abundant in this venom fraction, having a molecular mass of 7,098.1 Da. The analysis of the mass spectrometric data, together with comparative analyses using NCBI Protein BLAST, determined the primary structure of the most abundant peptide in Tcham27, and this designation was retained for this toxin. It was revealed that Tcham27 shares 100% sequence identity with Tppa2 (C0HLZ1.1), a β-type toxin previously identified in the venom of *Tityus pachyurus* - currently designated as *Tityus jaimei* - from Panama [14] that corresponds to fraction Tch8 described by Salazar et al. [7]. Additionally, the amino acid sequence of Tcham27 exhibited 98.4% similarity to the neurotoxin Tfe2 (QGW48885.1) from *Tityus festae*, a β-type neurotoxic peptide that selectively activates the voltage-gated sodium channel NaV1.3 (Figure 4) [21].

Since Tcham27 was the most abundant toxic peptide in the venom of *T. championi*, and its primary structure exhibits high sequence identity with other toxic peptides in the venom of species of the genus *Tityus*, it was selected as a candidate for recombinant expression.

### Heterologous expression of rTcham27

The recombinant toxin rTcham27 construct comprises 78 amino acids, including an N-terminal 6×His tag and an FXa cleavage site, which was not removed in the present study (Additional files 2 and 3). The experimental masses of the reduced and oxidized forms of rTcham27 were 62.1 and 8,954.1 Da, respectively, and agreed with the predicted molecular masses. The primary structure of Tcham27 contains eight cysteine residues forming four disulfide bonds, which is consistent with the structural characteristics of β-type scorpion sodium channel toxins, similar to the previously reported recombinant toxin named HisrTapp2 or rTapp2 (Table 2).

**Table 2:** Amino acid sequence of rTcham27 compared to rTppa2.

Toxin	Amino acid sequence^a^
rTcham27	MRGSHHHHHHGSIEGRKDGYLVGNDGCKYSCLTRPGHYCASECSRVKGKDGYCYAWMACYCYNMPNWVKTWSRATNKC
rTppa2	MRGSHHHHHHGSIEGRKDGYLVGNDGCKYSCLTRPGHYCASECSRVKGKDGYCYAWMACYCYNMPNWVKTWSRATNKC**R**

^a^ rTcham27 from this work and rTppa2 from Salazar et al. [19].

**Figure 5:**
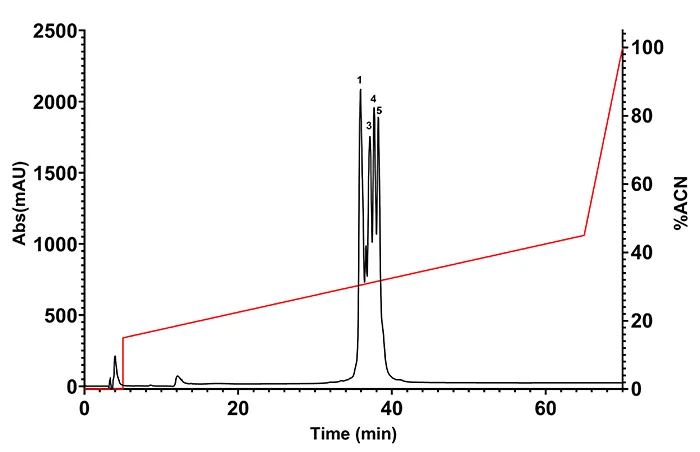
RP-HPLC of rTcham27 expression. Mobile phases consisted of solvent A (H₂O + 0.1% TFA) and solvent B (acetonitrile + 0.1% TFA), while the stationary phase was an analytical C_18_ column (Shimadzu® C_18_, 250 × 4.6 mm, 5 µm). The separation was carried out using a gradient of 15-45% solvent B over 65 min at a flow rate of 1 mL/min. Fractions were collected for further analysis. Fraction 4 corresponded to the main recombinant protein, while fraction 5 may represent a possible isoform or structurally modified variant of rTcham27.

After protein expression, rTcham27 was recovered from the insoluble fraction (inclusion bodies). The solubilization of the inclusion bodies enabled the purification of rTcham27 by nickel affinity chromatography, folding, and subsequent HPLC purification (Figure 5).

The HPLC chromatographic profile was obtained, revealing five fractions, of which fractions 4 and 5 had the experimental molecular masses of 8,953.9 and 8,951.3 Da, respectively, where fraction 4 matched the theoretically estimated oxidized molecular mass of 8,954.1 Da corresponding to the protein of interest (Additional file 6). The secondary structures of the recombinant fractions rTcham27-4 and rTcham27-5 were analyzed by CD and compared to rTapp2 (Additional file 7). The three neurotoxins exhibited prominent content of α-helical and β-strand secondary structures, consistent with canonical secondary structures of scorpion venom neurotoxins. Specifically, they demonstrated a higher proportion of α-helices, characterized by negative ellipticity bands (208-222 nm) and a positive band (198 nm). In comparison, rTcham27-5 showed a slight spectral difference relative to the recombinant proteins rTcham27-4 and rTppa2.

### Evaluation of the toxic activity of rTcham27

The biological activity of each fraction was evaluated by intracranial injection in CD-1 mice weighing 18 g (n = 6). Following administration of fractions 1 to 5, only fractions 4 and 5 exhibited transient symptoms in mice, including partially closed eyes (palpebral ptosis), reduced motor activity, anxious body grooming, forced abdominal breathing, tail erection (Straub sign), piloerection, forelimb paralysis, and hindlimb paralysis. These effects were more pronounced in mice treated with fraction 4 than in those receiving fraction 5, appearing a few minutes after injection and resolving spontaneously. All treated mice at 1 μg/mouse fully recovered within 2 h post-injection and remained asymptomatic during the 24 h observation period. No deaths or lasting effects were observed. 

In contrast, control mice receiving only water showed no signs of toxicity. The low biological activity observed in fraction 5 may be due to conformational differences that affect its activity. This reduced toxicity is likely attributable to incomplete folding, given that its measured molecular mass was 8,951.3 Da, suggesting that one disulfide bond is missing and resulting in a less toxic conformation. At higher concentrations, the intracranial lethality of rTcham27 fraction 5 was estimated at 8.2 µg/20 g mouse (Additional file 8), indicating lower toxicity compared with the native fraction nTcham27 (0.7 µg/20 g mouse); thus, the native toxin is approximately 12-fold more lethal.

## Discussion

### Mass spectrometry analysis of *Tityus championi* venom

Previous studies on *T. championi* venom utilized data-dependent acquisition (DDA) MS/MS, which only partially identified a few toxic fractions [7]. In the present work, data-independent acquisition (DIA) MS/MS, combined with the neural network-based software DIA-NN, was used to achieve a comprehensive proteomic characterization of the whole venom, identifying 339 sequences, 248 protein groups, and 51 toxin families. This revealed a high abundance of metalloproteases (which outnumbered voltage-gated sodium channel toxins), suggesting a mixed neurotoxic-enzymatic envenomation mechanism. The presence of serine proteases, lectins, CRISPs, chitinase, insulin-like growth factors, nucleotide pyrophosphatases, and other components was also identified, further expanding the functional spectrum of the venom (Additional file 5). Additionally, uncharacterized proteins containing cysteine-rich domains or repetitive regions represent previously unstudied proteins. Metalloproteases represented 17% of the total identified proteins. These metal ion-dependent enzymes, primarily zinc-dependent, display a conserved HEXXHXXGXXHD motif that includes both the catalytic site and the metal ion coordination site [22]. Although metalloproteases are commonly found in snake venoms, they have also been reported in the venoms of scorpions, insects, and spiders [23]. Their high abundance suggests that, unlike many other scorpion species in which neurotoxins predominate, envenomation by *T. championi* may involve substantial enzyme-mediated pathogenesis, associated with pathophysiological processes such as hemorrhage, edema, inflammation, hypotension, and necrosis. Indeed, clinical and experimental studies on scorpion stings from species of the genus *Tityus* highlight the occurrence of pancreatitis, which sometimes progresses to hemorrhage and pancreatic necrosis [24]. The presence of metalloproteases in the venom of the scorpion *Tityus serrulatus* has been linked to pancreatitis following scorpion stings, and these enzymes have been designated as antareases [25]. Furthermore, pancreatic alterations have been observed following the administration of venom from *T. discrepans*. Moreover, abdominal ultrasound has revealed pancreatic edema and, in some cases, necrosis of the pancreatic tail [26].

Ion channel-acting toxins constitute another major component of the venom, representing approximately 13% of the total identified proteins. Different classes of scorpion neurotoxins interact with specific sites on voltage-gated sodium channels (NaV), such as α-toxins that bind to receptor site 3, delaying channel inactivation, and β-toxins that interact with receptor site 4, promoting channel activation [24]. Together, these effects cause sustained depolarization of nerve and muscle fibers, which can lead to conduction block and contribute to the symptomatology observed in severe clinical cases associated with this species. Among these, the identification of CIIMET-designated toxins (11% of the total proteome), which are homologous to proteins from *Tityus discrepans,* is consistent with previous reports in geographically proximate species such as *Tityus* cf. *asthenes* and *Tityus jaimei* [14, 22]. These molecular similarities among species of the same genus inhabiting adjacent regions suggest shared evolutionary pressures or a common phylogenetic origin of these toxins.

At lower abundances, protein families such as CRISPs, serine proteases, and lectins contribute to the functional diversity of the venom. Particularly relevant is the detection of hyaluronidase, an enzyme that facilitates toxin diffusion by degrading hyaluronic acid in the extracellular matrix, thereby potentiating the action of other venom components.

The category designated as "other protein families", representing approximately 38% of all identified protein groups, included components related to fundamental cellular processes, such as cytoskeletal proteins, enzymes involved in lipid metabolism, and cellular defense and regulatory proteins. The detection of enzymes such as copper-zinc superoxide dismutase and lysosomal aspartic proteases suggests the presence of antioxidant mechanisms and controlled protein degradation in the venom gland, possibly associated with cellular homeostasis and protection against oxidative stress during venom synthesis and storage.

Conversely, the 10% proportion of uncharacterized proteins, which are mainly homologous to hypothetical proteins from *Centruroides sculpturatus*, reflects the limited transcriptomic coverage currently available for species of the genus *Tityus*. This knowledge gap emphasizes the need for broader transcriptomic studies to achieve a more complete characterization of these proteins and their potential biological functions, which could provide new perspectives for the identification of toxins with potential biomedical applications.

Together, these findings demonstrate the remarkable complexity of *Tityus championi* venom, the composition of which includes diverse proteins fulfilling multiple biological functions. This molecular complexity explains why the venom of this scorpion ranks among the most toxic in Panama, with an LD_50_ of 2.9 mg/kg, underscoring its clinical relevance and the importance of further study to support the development of effective therapeutic strategies.

### Chromatographic analysis of *Tityus championi venom*

Chromatographic analysis of *T. championi* venom revealed that its predominant molecular masses range from 3 to 11 kDa, values consistent with previously reported fractions showing activity on ion channels in other species of the genus *Tityus* [7]. This concordance suggests that, despite interspecific variability, a conserved mass range is associated with bioactive toxins targeting ion channels.

Likewise, the similarity of the molecular masses observed in fractions Tch24, Tch17, and Tch35 to those reported for *T. championi* populations from Panama and Costa Rica [7, 3] suggests the presence of conserved peptides in geographically distant populations of this species. This molecular conservation may reflect similar evolutionary pressures or the functional relevance of these peptides for the biological efficacy of the venom.

In this context, the identification of Tcham27 as the most abundant toxic protein and its 100% sequence identity with Tppa2 from *Tityus pachyurus*, now reclassified as *Tityus jaimei*, is particularly significant. This finding, together with its high similarity (98.4%) to Tfe2 from *Tityus festae*, demonstrates remarkable structural conservation of β-toxins among species of the genus *Tityus.* The presence of a conserved substitution (serine to asparagine) in Tcham27 and Tppa2 with compared with Tfe2 suggests evolutionary divergence events that do not compromise toxin functionality.

Both Tppa2 and Tfe2 are toxic to murine models and exhibit structural and functional properties associated with the modulation of voltage-gated sodium channels (NaV) [21]. The structural similarity of Tcham27 to these toxins, together with its abundance in the venom and its demonstrated toxicity in murine models, suggests that Tcham27 is a functional homolog of Tppa2 in *T. championi* venom, likely acting through similar mechanisms of NaV channel modulation.

### Heterologous expression of rTcham27 and its toxic activity.

Our rationale for selecting Tcham27 for recombinant expression was based on its abundance and toxicity in the venom, as well as on findings from previous studies. Specifically, Estrada et al. [27] found that expressing a recombinant *Centruroides sp*. neurotoxin (rCssII) with a cationic C-terminus (ArgCysArg) replaced the amidation of native CssII (ThrCysAsn*), rendering the recombinant rCssII as toxic as the native CssII. A similar rationale was applied to Tppa2, a *Tityus* toxin. Native Tppa2 has an amidated C-terminus (LysCys*) similar to native Tcham27, whereas for the recombinant rTppa2, an arginine residue was added to increase the cationic nature of its C-terminus (LysCysArg). In the present work, we identified Tcham27, a toxin identical to Tppa2; however, we chose to express it without an additional arginine residue to compare rTppa2-Arg and rTcham27 as immunogens in future studies. Based on this rationale, the selection of Tcham27 as a candidate for recombinant expression constitutes an important step toward the development of a specific antivenom and for future functional studies aimed at more precisely elucidating its mechanism of action and potential biomedical applications. 

The expression of rTcham27 resulted in two structural isoforms, rTcham27-4 and rTcham27-5. The latter exhibited lower biological activity, with one of its four disulfide bonds unformed, indicating misfolded disulfide pairing. Nevertheless, the circular dichroism spectrum of rTcham27-5 was slightly different from that of rTcham27-4, although all three recombinant proteins conserved the canonical α/β structure of scorpion neurotoxins that affect NaV channels. Since rTcham27-4 retained biological activity (although with lower potency compared with the native toxin), this was designated as rTcham27. The LD_50_ values were 0.94 (0.19-1.02) and 7.3 (1.6-10.0) µg/mouse for nTcham27 and rTcham27 (rTcham27-4), respectively. 

This reduction in toxicity is a common phenomenon in recombinant proteins bearing an N-terminal tag or extension. Specifically, rTcham27 contains an N-terminal extension of 16 residues (Table 2) that may affect receptor binding, as previously observed in other recombinant scorpion toxins [28, 29]. However, in this case, the reduction in biological activity may also be associated with a key structural difference: the absence of a C-terminal arginine residue present in the related toxin rTppa2 [19]. It is proposed that the absence of this residue might reduce receptor-binding affinity, thereby decreasing its lethality [28]. The omission of this residue in rTcham27 was intentional, aiming to demonstrate that the removal of certain terminal residues does not necessarily eliminate biological activity [30, 31]. However, the possible role of the missing C-terminal arginine in the reduced activity of rTcham27 remains speculative and requires experimental validation. Furthermore, alternative explanations, such as improper protein folding during recombinant expression or processing-related effects, cannot be excluded. Therefore, additional studies are required to evaluate the structural integrity of rTcham27, including secondary structure analysis by Fourier-transform infrared spectroscopy (FTIR), which could help clarify the folding state and conformational properties of the recombinant toxin [32]. Together, these results indicate that the expression system employed enables the production of the recombinant protein at a yield suitable (228 µg/L of culture) for functional studies, while retaining biological activity comparable to, albeit lower than, that of the native toxin.

This study paves the way for future research focused on the production of specific antivenoms to establish more effective therapeutic strategies against envenomation by T*. championi* and related species.

## Conclusion

This work provides a comprehensive proteomic characterization of the venom of *T. championi*, revealing a complex composition comprising 339 peptide sequences grouped into 248 protein groups belonging to 51 toxin families. Among these, metalloproteases and voltage-gated sodium channel-modulating peptides were the most abundant components, highlighting Tcham27 as a particularly relevant toxin due to its abundance and toxicity. These findings reinforce the molecular complexity and pharmacological potential of scorpion venoms from Panama and neighboring regions [33].

In addition, a heterologous expression system in *Escherichia coli* was successfully established, enabling the reproducible production and purification of the recombinant toxin rTcham27 in sufficient quantities for structural and functional characterization. Biological assays demonstrated that rTcham27 retains biological activity *in vivo*, inducing characteristic symptoms in murine models, although with reduced potency compared with the native toxin, likely due to structural variations resulting from the recombinant expression process.

Overall, these results expand current knowledge of *T. championi* venom composition and provide a basis for future studies aimed at developing specific antivenoms and exploring potential biomedical applications of its toxins.

## Data Availability

All data generated or analyzed during this study are included in this article and its additional files. The mass spectrometry proteomics data (raw and processed files) have been deposited in ProteomeXchange via the iProX repository (project ID IPX0013698000; subproject ID PXD069266).

## Supplementary material

Additional file 1 Design of oligonucleotides for *Tcham27* with restriction sites *Bam*H1 and *Pst*1.

Additional file 2 Representation of the gene construction for the heterologous expression of rTcham27. **(A)** The primary structure of rTcham27 is shown in bold. The 6×His-coding sequence is part of the pQE30 vector and is located upstream of the *Bam*HI/*Pst*I-cloned gene, such that the recombinant protein is 6×His-tagged at the N-terminus (italicized). Downstream of the *Bam*HI site, the sequence encoding the FXa recognition site is introduced (*IEGR* is underlined) right before the mature toxin sequence. Two stop codons (asterisks) are included at the end of the sequence encoding the mature toxin, downstream before the *Pst*I cloning site. **(B)** Electropherogram confirming the cloned *Tcham2*7 gene.

Additional file 3 Expression of rTcham27. **(A)** 15% SDS-PAGE and **(B)** Western blot analysis. MW: molecular weight markers (kDa); 1: uninduced cells, 2: induced cells, 3: soluble fraction, 4: inclusion bodies, 5: recirculating, 6: first wash, 7: second wash; 8: elution 1; 9: elution 3; 10: elution 5; 11: elution 7; 12: elution 9; 13: elution 11; 14: elution 13.

Additional file 4 Protein sequences identified in the venom of *T. championi*.

Additional file 5 Protein groups identified in the venom of *T. championi* by DIA-NN and classified into protein families based on homology.

Additional file 6 Mass spectra of rTcham27 structural isoforms. Fractions **(A)** rTcham27-4 and **(B)** rTcham27-5 showed molecular masses of 8,953.9 and 8,951.3 Da, respectively.

Additional file 7 Circular dichroism spectra of recombinant toxins.

Additional file 8 Mouse survival following intracranial injection. Six doses of nTcham27 and rTcham27 were injected intracranially into mice. Data were analyzed using a nonlinear regression equation, yielding calculated intracranial LD_50_ values of 0.94 (0.19-1.02) and 7.3 (1.6-10.0) µg/mouse for nTcham27 and rTcham27, respectively.

## References

[B1] Morán González M, de Patiño HA, Corzo G, Romero E, Gómez-Leija L (2025). Evaluation of some blood chemistry parameters caused by different
venom doses of Tityus and Centruroides scorpion species from
Panama. Toxicon.

[B2] Borges A, Miranda RJ, Pascale JM (2012). Scorpionism in Central America, with special reference to the
case of Panama. J Venom Anim Toxins incl Trop Dis.

[B3] Díaz C, Serna-Gonzalez M, Chang-Castillo A, Lomonte B, Bonilla F, Alfaro-Chinchilla A, Triana F, Sasa M (2023). Proteomic profile of the venom of three dark-colored Tityus
(Scorpiones: Buthidae) from the tropical rainforests of Costa
Rica. Acta Trop.

[B4] Arjona R (2022). Epidemiological situation of scorpion stings and snakebites in the
Republic of Panama. Years: 2020 and 2021.

[B5] Teruel R (2011). La verdadera identidad de Tityus championi Pocock 1898
(Scorpiones: Buthidae). Bol Soc Entomol Aragonesa.

[B6] Miranda RJ, Ríos M, Murgas IL, Araúz D, Suárez JA (2019). Escorpionismo por Tityus championi Pocock, en Panamá, reporte de
caso [Scorpionism by Tityus championi Pocock, in Panama, case
report]. Rev Med Panamá.

[B7] Salazar MH, Ortíz MH, Encarnación S, Zamudio F, Possani LD, Cleghorn J, Morán M, Acosta H, Corzo G (2023). A proteomic overview of the major venom components from Tityus
championi from Panama. Toxicon.

[B8] Lara AR (2011). Recombinant protein production in Escherichia
coli. Rev Mex Ing Quím.

[B9] Hernández-Alcántara G, García-Torres I, Alba-Martínez Z, Ramírez-Silva L (2021). Expresión de proteínas recombinantes en un sistema
heterólogo. Mens Bioquim.

[B10] Wiezel GA, Oliveira IS, Reis MB, Ferreira IG, Cordeiro KR, Bordon KCF, Arantes EC (2024). The complex repertoire of Tityus spp. venoms: Advances on their
composition and pharmacological potential of their toxins. Biochimie.

[B11] National Research Council (US) Committee for the Update of the Guide
for the Care and Use of Laboratory Animals (2011). Guide for the Care and Use of Laboratory Animals.

[B12] Demichev V, Messner CB, Vernardis SI, Lilley KS, Ralser M (2020). DIA-NN: neural networks and interference correction enable deep
proteome coverage in high throughput. Nat Methods.

[B13] Chen T, Ma J, Liu Y, Chen Z, Xiao N, Lu Y, Fu Y, Yang C, Li M, Wu S, Wang X, Li D, He F, Hermjakob H, Zhu Y (2022). iProX in 2021: connecting proteomics data sharing with big
data. Nucleic Acids Res.

[B14] Salazar MH, Samudio O, Arenas I, Hernández-Ortiz M, Clement H, Hernandez-Orihuela L, Encarnación-Guevara S, Cleghorn J, Acosta H, Corzo G (2025). Transcriptomics-informed proteomics of venom glands and crude
venom from Tityus cf. asthenes from Panama: enzymes, proteins, toxins, and
antimicrobial peptides. J Proteome Res.

[B15] Rincón-Cortés CA, Olamendi-Portugal T, Carcamo-Noriega EN, Santillán EG, Zuñiga FZ, Reyes-Montaño EA, Vega Castro NA, Possani LD (2019). Structural and functional characterization of toxic peptides
purified from the venom of the Colombian scorpion Tityus
macrochirus. Toxicon.

[B16] Clement H, Corrales-García L, Carpanta V, Jaimes G, Montero-Domínguez PA, Diego-García E, Corzo G (2026). Spider peptides from Brachypelma smithi with slightly different
amino acids at their C-terminal loops exert different insecticidal
activities. Amino Acids.

[B17] Vásquez Escobar J, Benjumea Gutiérrez DM, Lopera C, Clement HC, Bolaños DI, Higuita-Castro JL, Corzo GA, Corrales-Garcia LL (2023). Heterologous expression of an insecticidal peptide obtained from
the transcriptome of the Colombian spider Phoneutria
depilata. Toxins (Basel).

[B18] Mongeote Tlachy IG (2023). Expresión y caracterización de la toxina recombinante Tce 4 proveniente
del veneno *Tityus cerroazul*, grado de toxicidad y
reconocimiento de anticuerpos hiperinmunes ya establecidos.

[B19] Salazar MH, Clement H, Corrales-García LL, Sánchez J, Cleghorn J, Zamudio F, Possani LD, Acosta H, Corzo G (2021). Heterologous expression of four recombinant toxins from
Panamanian scorpions of the genus Tityus and Centruroides for production of
antivenom. Toxicon X.

[B20] Carbone C, Ayala MÁ, Del M, Cagliada P (2021). Ciencia y Bienestar de los Animales de Laboratorio.

[B21] Camargos TS, Bosmans F, Rego SC, Mourão CBF, Schwartz EF (2015). The Scorpion Toxin Tf2 from Tityus fasciolatus promotes Nav1.3
opening. PLoS One.

[B22] Escobar A, Salazar MH, Hernández-Ortiz M, Clement H, Encarnación-Guevara S, Cleghorn J, Acosta H, Corzo G (2026). Integration of proteomic and transcriptomic data of the venom and
venom gland from Tityus jaimei. J Proteomics.

[B23] Bordón KCF, Santos GC, Martins JG, Wiezel GA, Amorim FG, Crasset T, Redureau D, Quinton L, Procópio REL, Arantes EC (2025). Pioneering comparative proteomic and enzymatic profiling of
Amazonian scorpion venoms enables the isolation of their first α-Ktx,
metalloprotease, and phospholipase A2. Toxins.

[B24] De Sousa Alves R, do Nascimento NR, Barbosa PS, Kerntopf MR, Lessa LM, de Sousa CM, Martins RD, Sousa DF, de Queiroz MG, Toyama MH, Fonteles MC, Martins AM, Monteiro HS (2005). Renal effects and vascular reactivity induced by Tityus
serrulatus venom. Toxicon.

[B25] Fletcher PL, Fletcher MD, Weninger K, Anderson TE, Martin BM (2010). Vesicle-associated membrane protein (VAMP) cleavage by a new
metalloprotease from the Brazilian scorpion Tityus
serrulatus. J Biol Chem.

[B26] Murthy KRK, Rao RP, Natu VS, Kumar ZN, Lavanya M (2015). Suppressed insulin secretion, elevated mediators of inflammation,
hyper-insulinemia-insulin resistance: Insulin administration reverses
cardiovascular, metabolic changes, pulmonary edema and all other clinical
manifestations in scorpion envenoming syndrome. Indian J Mednodent Allied Sci.

[B27] Estrada G, Restano-Cassulini R, Ortiz E, Possani LD, Corzo G (2011). Addition of positive charges at the C-terminal peptide region of
CssII, a mammalian scorpion peptide toxin, improves its affinity for sodium
channels Nav1.6. Peptides.

[B28] Estrada G, Garcia BI, Schiavon E, Ortiz E, Cestele S, Wanke E, Possani LD, Corzo G (2007). Four disulfide-bridged scorpion beta neurotoxin CssII:
heterologous expression and proper folding in vitro. Biochim Biophys Acta.

[B29] Hernández-Salgado K, Estrada G, Olvera A, Coronas FI, Possani LD, Corzo G (2009). Heterologous expressed toxic and non-toxic peptide variants of
toxin CssII are capable to produce neutralizing antibodies against the venom
of the scorpion Centruroides suffusus suffusus. Immunol Lett.

[B30] Yang G, Zhou B, Wang J, He X, Sun X, Nie W, Tzipori S, Feng H (2008). Expression of recombinant Clostridium difficile toxin A and B in
Bacillus megaterium. BMC Microbiol.

[B31] Sánchez Mejía MA, Cardoso Arenas S, Miranda Blancas R, Clement H, Corrales García LL, Franco-Vásquez AM, Arenas I, Carbajal Saucedo A, Corzo G (2026). Recombinant expression of crotoxin and comparison to native
crotoxin as immunogens for anti-crotalid antivenoms. Protein Expr Purif.

[B32] Lee ZH, Kuek JS, Lim J, Chua AM (2018). Determination of protein secondary structures using FTIR
spectroscopy.

[B33] Samudio O, Hernández-Ortiz M, Clement H, Encarnación-Guevara S, Cleghorn J, Acosta H, Corzo G, Salazar MH (2025). Revisiting toxins with transcriptomics-informed proteomics of
venom glands and crude venom from Centruroides bicolor from
Panama. J Proteomics.

