# Supplementary material for: Proteomic characterization of Tityus championi venom and recombinant expression of its major neurotoxin
Source: J Venom Anim Toxins Incl Trop Dis. 2026 Sep 14;32:e20260024. doi: 10.1590/1678-9199-JVATITD-2026-0024 (PMC13574325; doi:10.1590/1678-9199-JVATITD-2026-0024)
Supplement: Supplementary file 1 [file 1678-9199-jvatitd-32-e20260024-s1.pdf]

J Venom Anim Toxins incl Trop Dis,
https://doi.org/10.1590/1678-9199-JVATITD-2026-0024

Supplementary Material to: Proteomic characterization of *Tityus championi* venom and recombinant expression of its major neurotoxin

Additional file 1. Design of oligonucleotides for *Tcham27* with restriction sites *Bam*H1 and *Pst*1.

Description	Length	Oligonucleotides (5' to 3')
rTcham27-pQE30-Fwr	30 nt	**GGA TCC ATC GAG GGA CGC AAA GAT GGT TAC**
rTcham27-pQE30-Rev	30 nt	**CTG CAG CTA TTA GCA TTT ATT TGT AGC ACG**

*Bam*HI site: GGA TCC; FXa site: ATC GAG GGA CGC; stop codons: TAA TAG; *Pst*I site: CTG CAG.
