# Supplementary material for: Proteomic characterization of Tityus championi venom and recombinant expression of its major neurotoxin
Source: J Venom Anim Toxins Incl Trop Dis. 2026 Sep 14;32:e20260024. doi: 10.1590/1678-9199-JVATITD-2026-0024 (PMC13574325; doi:10.1590/1678-9199-JVATITD-2026-0024)
Supplement: Supplementary file 2 [file 1678-9199-jvatitd-32-e20260024-s2.pdf]

J Venom Anim Toxins incl Trop Dis,
https://doi.org/10.1590/1678-9199-JVATITD-2026-0024

Supplementary Material to: Proteomic characterization of *Tityus championi* venom and recombinant expression of its major neurotoxin

A

***Bam*H1**

-PT5-
*MRGSHHHHHHGSIEGR***KDGYLVGNDGCKYSCLTRPGHYCASECSRVKGKDGYCYAWMACYCYNMPNWVKTWS RATNKC****LQ

***Pst*1**

B

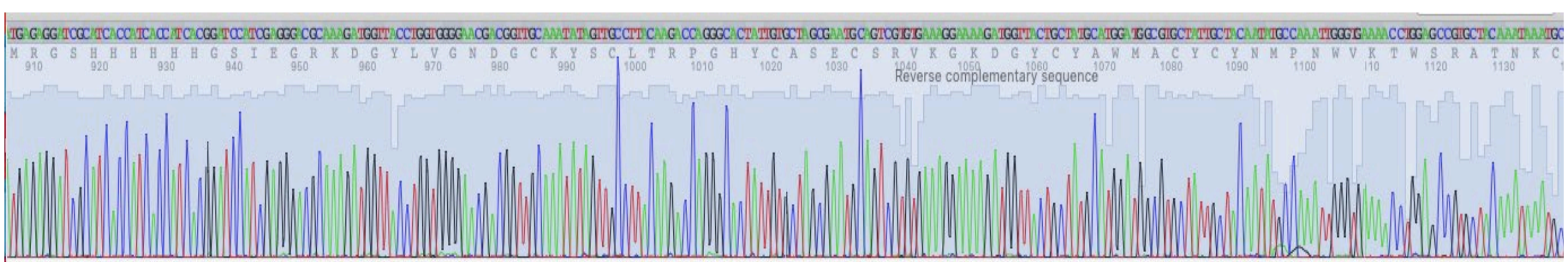

Additional file 2. Representation of the gene construction for the heterologous expression of rTcham27. **(A)** The primary structure of rTcham27 is shown in bold. The 6×His-coding sequence is part of the pQE30 vector and is located upstream of the *Bam*HI/*Pst*I-cloned gene, such that the recombinant protein is 6×His-tagged at the N-terminus (italicized). Downstream of the *Bam*HI site, the sequence encoding the FXa recognition site is introduced (*IEGR* is underlined) right before the mature toxin sequence. Two stop codons (asterisks) are included at the end of the sequence encoding the mature toxin, downstream before the *Pst*I cloning site. **(B)** Electropherogram confirming the cloned *Tcham27* gene.
