# Supplementary material for: Proteomic characterization of Tityus championi venom and recombinant expression of its major neurotoxin
Source: J Venom Anim Toxins Incl Trop Dis. 2026 Sep 14;32:e20260024. doi: 10.1590/1678-9199-JVATITD-2026-0024 (PMC13574325; doi:10.1590/1678-9199-JVATITD-2026-0024)
Supplement: Supplementary file 3 [file 1678-9199-jvatitd-32-e20260024-s3.pdf]

J Venom Anim Toxins incl Trop Dis,
https://doi.org/10.1590/1678-9199-JVATITD-2026-0024

Supplementary Material to: Proteomic characterization of *Tityus championi* venom and recombinant expression of its major neurotoxin

A

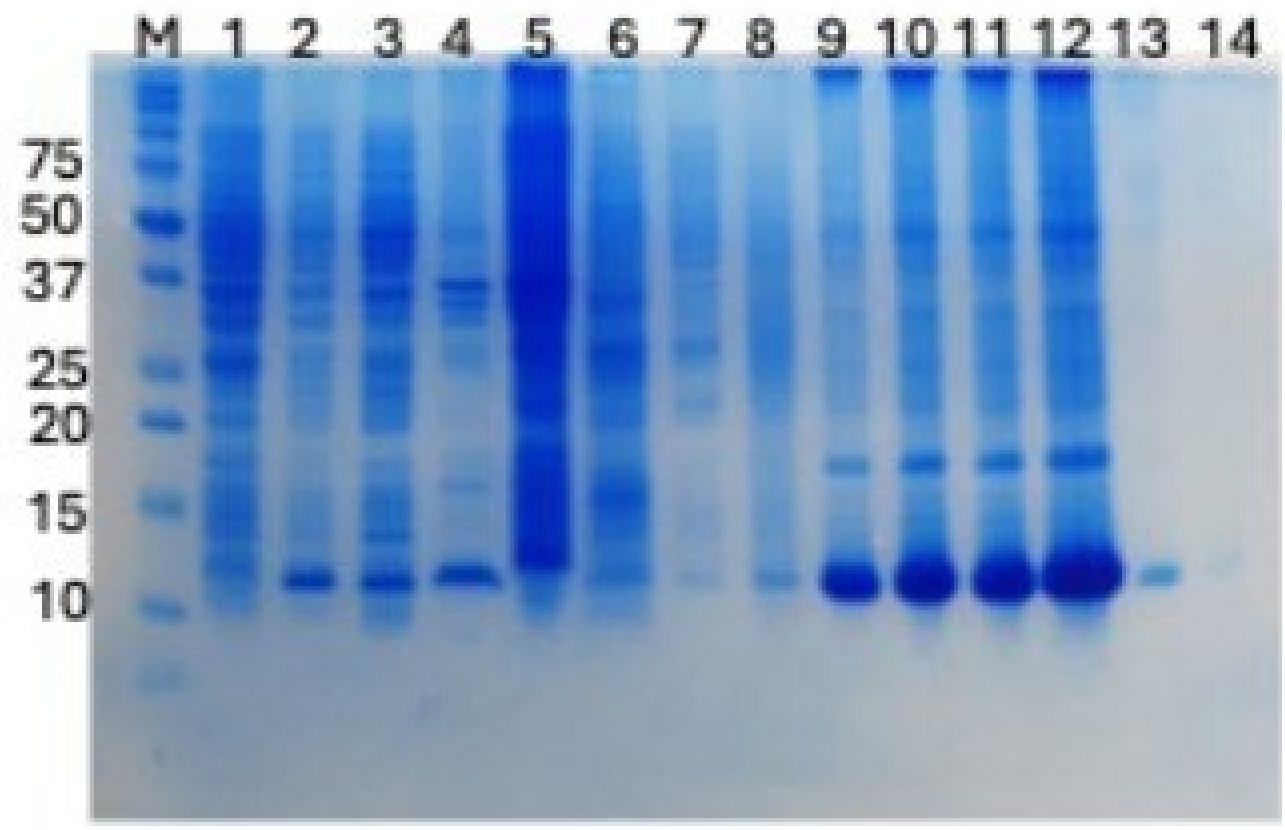

B

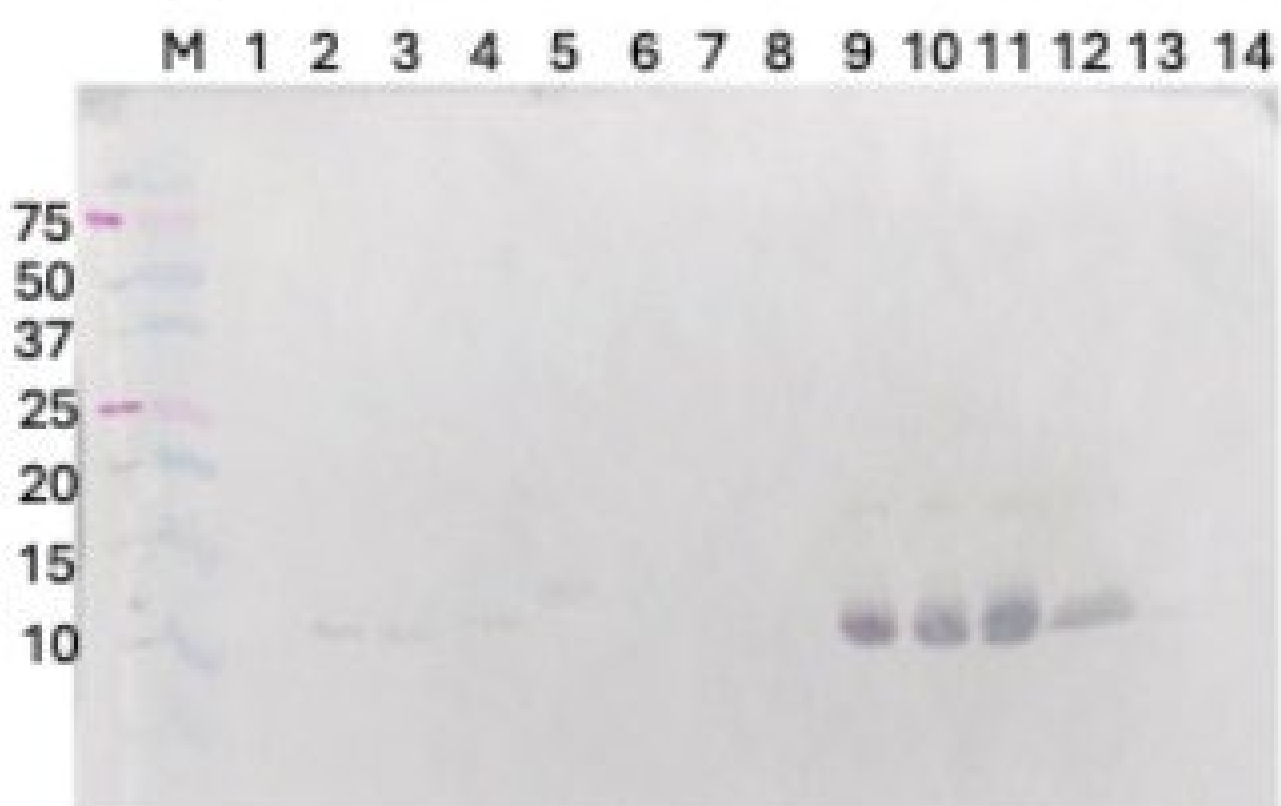

Additional file 3. Expression of rTcham27. **(A)** 15% SDS-PAGE and **(B)** Western blot analysis. MW: molecular weight markers (kDa); 1: uninduced cells, 2: induced cells, 3: soluble fraction, 4: inclusion bodies, 5: recirculating, 6: first wash, 7: second wash; 8: elution 1; 9: elution 3; 10: elution 5; 11: elution 7; 12: elution 9; 13: elution 11; 14: elution 13.
