# Supplementary material for: Proteomic characterization of Tityus championi venom and recombinant expression of its major neurotoxin
Source: J Venom Anim Toxins Incl Trop Dis. 2026 Sep 14;32:e20260024. doi: 10.1590/1678-9199-JVATITD-2026-0024 (PMC13574325; doi:10.1590/1678-9199-JVATITD-2026-0024)
Supplement: Supplementary file 4 [file 1678-9199-jvatitd-32-e20260024-s4.pdf]

J Venom Anim Toxins incl Trop Dis,
https://doi.org/10.1590/1678-9199-JVATITD-2026-0024

Supplementary Material to: Proteomic characterization of *Tityus championi* venom and recombinant expression of its major neurotoxin

Additional file 4. Protein sequences identified in the venom of *T. championi.*

Sequence identifier	Amino acid sequence[a]	MM*
**Alfa-amylase**		
>TRINITY_DN13870_c0_g1::TRINITY_DN13870_c0_g1_i2::g.10108::m.10108 Tja_AA01	>Tcham_AA01<br>SYHEPNTVQGKSVIVHLFEWRWNDVADECEQFLSPFGFGGVQISPPNENGIVWEPFWKKEVKRPWFERYQPVSYKLGTRSGSESEF<br>REMVRRCNNAGVRIYVDAIINHMTGDIGEGKGTAGSDFNPAALQYYGVPYGPSDFNTEKCHTSSGNIENYQDKYQVRDCRLSGLAD<br>LNLSKPYVRDKIVEYLNYLIDTGVAGFRFDAAKHMWPSDIQAIRDRLKNLNTEFFPPNTRPFLFQEVIDLGGGEAVKAEEYLHNGRVT<br>EFRYGKHLGDIIRKNNNQRLRYLKNFGEEWGMVSGGNAVTFIDNHDNQRGHGAGGFGSILTFFEAKMYKMAVAFMLAWPYGLPR<br>VMSSYNWPRYIENGKDKNDWIGPPHDDNYNIKPVIRNSDLSCSNGWVCEHRWRQIYNMVKFRNVAGFEPVVHWWDNDYHQIA<br>FGRKGKAFLAINNDNQPMDQNLLTGLPSGTYCDVISGNVENNSCTGKKVEVGNDGRARIFIDNKWEDPMLAIHIESKLK	57916.06
>TRINITY_DN13685_c0_g1::TRINITY_DN13685_c0_g1_i1::g.28779::m.28779 Tja_AA01	>Tcham_AA02<br>SYHEPNTVQGKSVIVHLFEWRWNDVADECEQFLSPFGFGGVQISPPNENGIVWEPFWKKEVKRPWFERYQPVSYKLGTRSGSESEF<br>REMVRRCNNAGVRIYVDAIINHMTGDIGEGKGTAGSDFNPAALQYYGVPYGPSDFNTEKCHTSSGNIENYQDKYQVRDCRLSGLAD<br>LNLSKPYVRDKIVEYLNYLIDTGVAGFRFDAAKHMWPSDIQAIRDRLKNLNTEFFPPNTRPFLFQEVIDLGGGEAVKAEEYLHNGRVT<br>EFRYGKHLGDIIRKNNNQRLRYLKNFGEEWGMVSGGNAVTFIDNHDNQRGHGAGGFGSILTFFEAKMYKMAVAFMLAWPYGLPR<br>VMSSYNWPRYIENGKDKNDWIGPPHDDNYNIKPVIRNSDLSCSNGWVCEHRWRQIYNMVKFRNVAGFEPVVHWWDNDYHQIA<br>FGRKGKAFLAINNDNQSMDQNLLTGLPSGTYCDVISGNVENNSCTGKKVEVGNDGRARIFIDNKWEDPMLAIHIESKLK	57906.02
**Hyaluronidase**		
>TRINITY_DN13885_c0_g1::TRINITY_DN13885_c0_g1_i1::g.10008::m.10008 Tja_Hyal01	>Tcham_Hyal01<br>DFKVYWEVPSFLCSKKFKINVTQSLASHKVLVNHGEGFNGDKIVIFYESQLGKYPYVNSNDVDINGGMLQIADLAEHLKVARDNITKFI<br>PNPKFNGIGVIDWEVWRPNWEFNWGKMKVYSKRSIDIVRSKHPTWSADRIEEVAKQEWEKSAEEWMVKTMKLAQDMRPDAV<br>WCYYIFPDCYNYNGNDQAKQFACNPKTKLENSRLSWLWKQSTAICNSIYMQESHIRRYNMTQRVWWTDARLREAIRVAHSNTPIY<br>PYINYILPGKYVKVVPAMDFKRMLGQIASLGLDGAVIWGSSYHVMTEHNCKITADYVNNVISPTVATVVLNTRRCSQAICKGRGNCI<br>WPEEPYTSWKYLVDPKMHVFKPTNIHCRCKGYTGRYCHISQ	44661.32
**Lipolysis Activating Peptide**		
>TRINITY_DN37510_c0_g1::TRINITY_DN37510_c0_g1_i1::g.32376::m.32376 TaPBP-LVP02	>Tcham-LVP01<br>DIPGNYPVDFQGSNYECIIYDRCKRDCKIHGVSYGYCYAGVCYCEYLPNENKNFWDVMKKQCDYMNN	7958.95
>TRINITY_DN12113_c1_g1::TRINITY_DN12113_c1_g1_i1::g.34120::m.34120 Tja_LVP01	>Tcham-LVP02<br>TTDGGYPRYFSFGYKCQDWGVNKYCNTVCKLHRGNYGYCFAGDCYCEGLTEENKLFWNVYRKYCNNPLFD	8301.27

Sequence identifier	Amino acid sequence[a]	MM*
**Alfa-amylase**		
**Insulin-like Growth Fact**		
>TRINITY_DN14489_c2_g1::TRINITY_DN14489_c2_g1_i2::g.16650::m.16650 TaPBC_SIGF_06	>Tcham_SIGF_01<br>HKCPKCEKNSCTLITPKNCPAGPVLDFCKCCAICGKGLNEKCGGKRNISGICVTGLVCKVPDNSADNIGTCKKA	7701.15
>TRINITY_DN14274_c8_g3::TRINITY_DN14274_c8_g3_i2::g.65880::m.65880 Tja_SIGF01	>Tcham_SIGF_02<br>LKCRCTDVKCVDKTEEDCPEVGVTKYGCTCCKVCAKNIGEKCGGIYNVTGICGRNLVCVKPPPPEGANELEIQRHNFNSRGNCQKKIEN	9752.27
**Cysteine-Rich Secretory Proteins**		
TRINITY_DN14269_c0_g1::TRINITY_DN14269_c0_g1_i1::g.65444::m.65444 Tja_CRISP02	>Tcham_CRISP01<br>QNCPAKYLRFSPKHTFCLPPTCKVEAGGKVSDSDIQIILAAHNELRNKLATGKENQYHALPSAANMMQLEWDNELAAIAQAHASQCKFEHDSGDQRAVGNFSVGQNLLETSGILSIDWSKVKIWYTTEVKYFRPEYNKPFQFHGNYGHFSQVIWAKTWKVGCGVAGYVKDGVKRVLYTCNYGPAGNLIRSEAYQVGKPCSACPQNTKCSSKYPGLCSSLTPDGPQISRPSSNDYLLYCDFSDKDPKACKDVKMSGSRSFTTQKLYTGKYVTAVFNEGENMTINFGKFKHKDGLCAFLIGRFGPDVAGQSAGSFVSFHFSAPGLIFPDGMKESRVISSWHTIGISMRSDREMQVSYIFQVNKGAPLQYFEFKEFGVKGGSCP	42247.97
>TRINITY_DN14447_c1_g2::TRINITY_DN14447_c1_g2_i1::g.18177::m.18177 Tja_CRISP18	>Tcham_CRISP02<br>KACSECPPGMGCDSQYKRLCAPVKPAADTIKINPATTNGKTNAAIMNTYDQEKSKSTTLASNEVLWKCDFSNRTKKQCESKMKCLANWKTVCGLKSCHKEIIIDQPNCSLMFLKPILVENQACLIFQYKKEHLPAAQGESNLTAVAVWNEGENYKTTFIYDNTNKWALANLHILAKNQKVQIGFIVRNADNSEGQKISIRKLYILNGSC	23349.97
>TRINITY_DN13590_c0_g2::TRINITY_DN13590_c0_g2_i1::g.56898::m.56898 TaPBP_CRISP02	>Tcham_CRISP03<br>DCPALYRRYSKEHTFCKTKNQKCHIKRWGVSEDDREIIIDLHNKVRNNIALGKDQSGRLPAAGDMLEMEWDDELAQIAQKLTDQCVYKHDCDDCRKVENFDVGQNIYTATITAVDPPEPFWVDAVRSWYSEIYRFTPDFIKPFTSDHATGHFTQMAWSKTWRVGCGYVLYEKRKDSWTQLYVCNYGPAGNMDDSELYKIDKPCEKCPGNTCCGPHCKKKRSTSYLGLCSVLNGRGPDFDDIDFSNYIFNCDFKPESSSDCNNKVEGSNKWQTRQIISDVYKTVVLNGGESTTLKFTSNIQYKDGFCLTISFRKGPSVAGTKNAGEFNVQLERKGASPLNFQLDSEGNEWLPYSMGIPMKQPMLINLKFSVPKGSPAQYLDISYIRARPGVCN	44659.43
>TRINITY_DN14001_c0_g1::TRINITY_DN14001_c0_g1_i1::g.14237::m.14237 TaPBP_CRISP02	>Tcham_CRISP04<br>DCPALYRRYSKEHTFCKTKNQKCHIKRWGVSEDDREIIIDLHNKVRNNIALGKDQSGRLPAAGDMLEMEWDDELAQIAQKLTDQCVYKHDCDDCRKVENFDVGQNIYTATITAVDPPEPFWVDAVRSWYSEIYRFTPDFIKPFTSDHATGHFTQMAWSKTWRVGCGYVLYEKRKDSWTQLYVCNYGPAGNMDDSELYKIDKPCEKCPGNTCCGPHCKKKRSTSYLGLCSVLNGRGPDFDDVDFSNYIFNCDFKPESSSDCNNKVEGSNKWQTRQIISDVYKTVVLNGGESTTLKFTSNIQYKDGFCLTISFRKGPSVAGTKNAGEFNVQLERKGASPLNFQLDSEGNEWLPYSMGIPMKQPMLINLKFSVPKGSPAQYLDISYIRARPGVCN	44645.40

Sequence identifier	Amino acid sequence[a]	M M*
**Alfa-amylase**		
>TRINITY_DN14447_c1_g2::TRINITY_DN14447_c1_g2_i5::g.18167::m.18167 TaPBC_Unk_90	>Tcham_CRISP05<br>ECDKKYSNITVEHSMCKPKNETCQLDRRGYNKMGTILNVHNEIRNQMRPIRKRKFSIATNMLKMEWDDELYQIAAKYVRQCVEKP<br>DCPKCHQTGGTHVEQNFAVATYGVETESGPGQRFRDIIVQWADELLNVTDSKTIRRFQSAKSPKKNWVNIFRASTYKVGCDSMSYK<br>VNEETFNEIYVCNYTPATIEEGEEVYKGGKKACSECPPGMGCDSQYKRLCAPVKPAADTIKINPATTNGKTNAAIMNTYDQEKSKSTT<br>LASNEVLWKCDFSNRTKKQCESKMKCLANWKTVCGLKSCHKEIIIDQPNCSLMFLKPILVENQACLIFQYKKEHLPAAQGESNLTAVA<br>VWNEGENHEAIFLKEDTNIWTLANLNIPVKNKKIQVGFIVRKARDSEEQKISIRYLYVLNGSC	46<br>74<br>7.5<br>6
>TRINITY_DN14685_c3_g4::TRINITY_DN14685_c3_g4_i1::g.59875::m.59875 TaPBC_Unk_90	>Tcham_CRISP06<br>CKPKNETCQFMRRGYNKMRTILKVHNDVRNNIKPIRMKKFSIPTNMLKMEWDDELYQIAAKYVRQCVEKPDCPKCHQTGGTHVE<br>QNFAVVNYDNPNEFGPAERFQGVIIQWADELLNVTDFDIIRHFKSSESSKKNWVNIFRASTYKVGCDSMSYQVDNKTINEIYVCNYT<br>PATIEEGEQIYKDGDKACSECPPGMGCDSHYKRLCAP	24<br>11<br>6.4<br>5
>TRINITY_DN14293_c0_g2::TRINITY_DN14293_c0_g2_i1::g.64694::m.64694 Tja_CRISP18	>Tcham_CRISP07<br>EKPDCPKCHQTGGTHVEQNFAVATYSVDNNLGPAQRFRDIILQWANELLSVTDFETIRHFQSAKSPQKNWVNLFRASTYKVGCDS<br>MSY	10<br>03<br>8.2<br>1
**Nucleotide Pyrophosphatase**		
>TRINITY_DN13965_c0_g1::TRINITY_DN13965_c0_g1_i1::g.69707::m.69707 Tja_NPP01	>Tcham_NPP01<br>CISKTVDKKVILISFDGFRWDYYKAFNTPNLDYIANTGVHAPNGVQAIFTTKTFPNHYSIATGLYEESHGIVDNNMYDPVFNEIFQMK<br>KPDPKWWGGEPIWVTARKQGLKSGVYFWPASDIPIKDTFPNIYFKYNNTVSMETRIETAVSWLANDEADVALIYFNEPDKTGHRKG<br>AMSDAVGKMVETVDKVVGLLLQKLLETFLLDRVNLIIMSDHGMVNLTEVVVLENYLRVKEDIKWMPMSGPVAGILPYEGKLDTVYN<br>KLKNAHPNMTVYKKEDIPERWHYKNNRRIMPIVAVADQGYLVAKEFGTDNAFGQHGYDNSLLSMRPIFFARGPAFKKNYQTDVFE<br>TVNIYPLICHLLGIEPAPNNGSLDSVIHLLKDSAKGKHYKTPLRNRLF	44<br>84<br>6.5<br>3
>TRINITY_DN12351_c0_g1::TRINITY_DN12351_c0_g1_i1::g.43835::m.43835 Tja_NPP07	>Tcham_NPP02<br>QNQSLLILMVDGVRWNYIDEEGLMGFPAMSKYGVRAQYVLPIFPSNTYPNWLTMVTGVYSENHGFVNNYMYDINEDKYFEASPTS<br>DNLWWKDVEPVWITAEKNGIKSALYNWDGCDIEVDGVKASYCHPYKSYKIWPSVDKDTGKAAFEILNLFQQGYRLGLLYYEPVDGT<br>GHIFGPESWLRKFVFRRIDRILYDIQQEIESKGMTNEVNVIVISDHGMTTIRLGQTKVIDINPVVSSNDIKVMLDVFCMSMLWPENGK<br>EEKVYNDLVKANLEGLQVYRKKDIPEIFHFREHEHIAPILMLTNNSEYIIVPLNDQKRMKPFYILGRITSFFLSGIHGCNQYNDEMVDM<br>RAIFLARGPAFKNLISPPLHMVDHYNIFCHVLGIEPLPNNGSWHRVKEMFRSNLDL	46<br>91<br>6.8<br>1
>TRINITY_DN14382_c0_g1::TRINITY_DN14382_c0_g1_i5::g.5192::m.5192 Tja_NPP05	>Tcham_NPP03<br>RDVESDTDQCLLIILLDGVRWDYVNDENLKGFPRLARNGVKAEYVTPVFPSNSYPNWYSIATGLYPENHGIVDNFMYDEKYDDYFL<br>MAPDKGVFKEHWWNDAEPLWITAEKNSIKTYMFNWVGCEVPIQGLTASYCKSFRALQLWWTSVNRETKSAIFNILDQFHSNTSRL<br>GFVYYEPIDALGHQFGPNFALTRKYVRDVDSILYDMQEEIQKRNLEKRVNVVVISDHGMSVKTLFRTKTISLDDIIPSNDIKVMLNVHY<br>NSMIWTVEGKEEEVYQSLKKANKPGLRVYKKEELPDYYHFKNHKLIAPIILIADKNYNILPLRSFSKVKPYYPLPFIFTYFQPGVHGLDPF<br>SKESEEMRTIFFASGPSLKKNYVSPPLEMVDHYNVFCHVLGITPLPNNGSYDRIKEFLIPKTV	48<br>31<br>1.2<br>6

Sequence identifier	Amino acid sequence[a]	M M*
**Alfa-amylase**		
>TRINITY_DN652_c0_g1::TRINITY_DN652_c0_g1_i1::g.68126::m.68126 Tja_NPP08	>Tcham_NPP04 RHKVLLVLVDGCRWDYPDTDSTLTGFKKLAEKGVRAEYVTPIFPSNSYPNWYTIVTGLYAESHGFVQNMMYDPIYKDFFLMGPNKTASVAHWWNHAEPIWITAEKNNLRTSLYWWDGCQVEIKGKRPTFCVPYKYVGEDWETVDADTRNAFLEALDKLKSNEVQLVQVYYEAVDHKGHKYGPDSDERKEAFRNIDRLLYELQEEITKRGLEDQVNLVVVSDHGMTLVDPRITKVIYLDSVLQPSDIKYMIYQGASCMILPEEGKDEEVYKQIKNQNITGLNVYRKEDIPDIYHLKHNYLTLPILLVADEGYVISGLDYPDKSIPGRSALTRGGSHGYDPYKVKDMRTIFYARGPDLKKGYVSPPIENVDHYNLMCHLLGIKPLSNNGSLERIEGMFTNSASYGKSISVQIMMFTLAVVLIIT	48445.30
**Serine Proteases**		
>TRINITY_DN14405_c0_g1::TRINITY_DN14405_c0_g1_i1::g.14673::m.14673 Tja_SP04	>Tcham_SP01 AAVRDEKRIYGGRFVNPGEFPWMVFIRLTDEFNCSGFLISPSFVLTAAHCMFRPVEELWARLGTVDRESGPQYRFQSLLVHPDYDNLTYYGDIALLKLTSPVDFNENVNRICLANNADYYLPNIPVLQMGWGRFSNETADITRILKVTEEGYIFDHDVCTYMFSLFNYTLWDGTVCIRNSGSDGVCEGDSGGPLVTRNGDTYTAIGLESVGFYENCSVDNTFAEIFTEVLHHRQWIIDNIDENIC	27903.33
>TRINITY_DN14624_c0_g1::TRINITY_DN14624_c0_g1_i2::g.61100::m.61100 Tja_SP10	>Tcham_SP02 DISYRSENKNENDTCLTASKIRGICKTVTDCRTITDLSRSRPIICSWRKKTPIVCCPILECGKRPSRIRNFYQTVVGGTNSIKDSWPWMVSLHRKTAIGLRYQCGGTIFKNNYILTAAHCFDSLSGTKKPSDFIIKIGGNRRDEGKDYKITKIKIHPKYKSGQYYNDIAILKLDLTVPNVTAICMPSDKEKYGKKPVKVIGWGLTSFGGPPADALQEADLNIIPNRRCNDSYSRLLTGPFPNGITSTFLCAGLTSGGKDACQEDSGSPLMMLNKFKWKIVGIVSFGYQCAKAGYPGIYTKVSSYLNWIKRNSK	34901.42
>TRINITY_DN14527_c6_g2::TRINITY_DN14527_c6_g2_i2::g.46713::m.46713 Tja_SP16	>Tcham_SP03 SSESKSSYENDDRPICITDMDFNNIKTRIVGGQDADINSFRFAAGLLATPGMSGQIRTDSAICGGTLITERHLLTAGHCLYNKEYLMSYMKVNIGDYDTRNDNEVKNYIRGIKSIYIHPKYIDEKFNNDICILELDNPVPLSRSSDLRAAILPPQDSSLSPGTVCTVWGWGRLSYKGERSHVLQSVKVPITSREECQKHLSHTINQNMICAGGEEGKDACLGDSGGSLLVQVENRYVVCGVVSFGKNCALPEVSGVYANVARYTDWIIEMTKNADCKPRIYPEEKYPH	32017.18
>TRINITY_DN14055_c0_g1::TRINITY_DN14055_c0_g1_i3::g.15337::m.15337 Tja_SP16	>Tcham_SP04 SSESKSSYENDDRPICITDMDFNNIKTRIVGGQDADINSFRFAAGLLATPGMSGQIRTDSAICGGTLITERHLLTAGHCLYNKQYLISYMKVNIGDYDTRTDNEVKNYIRGIKSIYIHPKYIDEKFNNDICILELDNPVPLSRSSDLRAAILPPQDSSLSPGTVCTVWGWGRLSYKGERSHVLQSVKVPITSREECQKHLSHTINQNMICAGGEEGKDACLGDSGGSLLVQVENRYVVCGVVSFGKNCALPEVSGVYANVARYTDWIIEMTKNADCKPRIYPEEKYPH	31985.16
>TRINITY_DN14055_c0_g1::TRINITY_DN14055_c0_g1_i2::g.15332::m.15332 Tja_SP16	>Tcham_SP05 SSESKSSYENDDRPICITDMDFNNIKTRIVGGQDADINSFRFAAGLLATPGMSGQIRTDSAICGGTLITERHLLTAGHCLYNKEYLMSYMKVNIGDYDTRTDNEVKNYIRGIKSIYIHPKYIDEKFNNDICILELDNPVPLSRSSDLRAAILPPQDSSLSPGTVCTVWGWGRLSYKGERSHVLQSVKVPITSREECQKHLSHTINQNMICAGGEEGKDACLGDSGGSLLVQVENRYVVCGVVSFGKNCALPEVSGVYANVARYTDWIIEMTKNADCKPRIYPEEKYPH	32004.18
CIIMET Toxins		
>TRINITY_DN14338_c0_g1::TRINITY_DN14338_c	>Tcham_CIIMET_01	1104

Sequence identifier	Amino acid sequence[a]	MM*
**Alfa-amylase**		
0_g1_i4::g.3426::m.3426 Tja_CIIMET_12	EGNSLADMYCGYKDMNKFYKCFKENVSEESWKNYNDCANEYNIPPEPEDRRKFFCDRGNESKVKKWYFCIITKLN NVDINAFNENLELCLKQ	6.41
>TRINITY_DN14553_c3_g1::TRINITY_DN14553_c3_g1_i4::g.47433::m.47433 TaPBC_CIIMET_10	>Tcham_CIIMET_02 EGNSLADMYCGYKDMKKFYKCFKENVSEESWKNYNDCANEYNIPPEPEDRRKFFCDRGNESKVKKWYFCIITKLNNVDINAFNENLELCLKQ	11060.48
>TRINITY_DN14615_c1_g1::TRINITY_DN14615_c1_g1_i5::g.60932::m.60932 Tja_CIIMET_03	>Tcham_CIIMET_03 DDTIMDNFCAHIDAVKFYDCLYNTAPKHAVSMYNKCASENGAPSDSKKRRDFACDHKNDDKIRLWYYCLHKSFDADEVDDFHKSVEICLKQ	10616.86
>TRINITY_DN14450_c2_g2::TRINITY_DN14450_c2_g2_i13::g.16112::m.16112 Tja_CIIMET_04	>Tcham_CIIMET_04 SDSFIDTYCAHNDAVKWYECFNTTATKETVIKFNKCASEQNAPLDSKERRQFICDRNNADKIRLWYSCFYSTMNYDQIIDFHKTVGICSKM	10729.07
>TRINITY_DN14452_c1_g5::TRINITY_DN14452_c1_g5_i1::g.15266::m.15266 Tja_CIIMET_03	>Tcham_CIIMET_05 NKSLIDSFCAHTDIVKFYDCFYNTATKETVNKFTKCANEQTIPLDSKERREYFCDRNNADGVRLWYTCLYSSMNSDEANDFHTSVDKCLKQ	10657.84
>TRINITY_DN14553_c3_g1::TRINITY_DN14553_c3_g1_i2::g.47431::m.47431 Tja_CIIMET_12	>Tcham_CIIMET_06 EGNSLADIYCGHKDIKKFYQCFEESFSTESNKNYNDCANEYNIPPEPEDRRKFFCDRGNESKVKKWYFCIITKLNNVDINAFNENLELCLKQ	10920.18
>TRINITY_DN14615_c1_g2::TRINITY_DN14615_c1_g2_i2::g.60939::m.60939 Tja_CIIMET_03	>Tcham_CIIMET_07 NDSVFDSFCAHTDAVKFFDCLYNSAPKDSVSMYNKCASEQNAPLDSKNRREFICDRNNDDKVRLWYHCLLKSFNSDDVNDFHKSLEICLKQ	10622.80
>TRINITY_DN14257_c2_g3::TRINITY_DN14257_c2_g3_i2::g.61252::m.61252 Tja_CIIMET_04	>Tcham_CIIMET_08 GDSFIDTYCAHNDAVKWYECFNTTATKETVIKFNKCASEQNAPLDSKERRQFICDRNNADEIRLWYSCFYSTMNHDQIIDFHKTVGICSKM	10673.95
>TRINITY_DN14338_c0_g1::TRINITY_DN14338_c0_g1_i5::g.3423::m.3423 TaPBC_CIIMET_11	>Tcham_CIIMET_09 EGNSLADMYCGHKDIKKFYQCFEESFSTESNKNYNDCANEYNIPPEPEDRRKFFCDRGNESIVKNWYYCLMKKFNSFDLNAYNENAELCLKQ	10999.18

Sequence identifier	Amino acid sequence[a]	MM*
**Alfa-amylase**		
>TRINITY_DN12945_c0_g1::TRINITY_DN12945_c0_g1_i1::g.30257::m.30257 Tja_Unk_66	>Tcham_CIIMET_10<br>EEISVHDKYCEYKNIEKFYECLKLGVADTTWTIFIKCSKEITMSSEPRGYQKFFCESLHEGTVRLWYACLMEKLKASEINEFFKFSEKCLFE	11020.71
>TRINITY_DN14351_c5_g1::TRINITY_DN14351_c5_g1_i7::g.4648::m.4648 Tja_Unk_51	>Tcham_CIIMET_11<br>ETISVPKETWDKFSECASDKKIPIEPEEQKKFFCDASNEVKVTWFYYCLLESFTPTEMNDFHDANEKCLSG	8306.28
>TRINITY_DN14215_c4_g4::TRINITY_DN14215_c4_g4_i3::g.66249::m.66249 TaPBC_Unk_131	>Tcham_CIIMET_12<br>EISLRERYCVFNPIEKYYECLIENIYENVWVEYNVCSKKQNFPTEYNALRDVFCDSVNDNDLRIWYFCLLSALSPSESQNFHESAEKCLQ	10822.11
>TRINITY_DN14128_c0_g1::TRINITY_DN14128_c0_g1_i3::g.75886::m.75886 TaPBC_Unk_54	>Tcham_CIIMET_13<br>GSIYDAYCGSEAGEKFHNCVQYNVSQECWNSLMKCLAKLNLPADPTSLRNFFCDPKNSKEVGKWYLCVLQTLSKSQIEEFHDTTVKCFAKT	10352.78
>TRINITY_DN14128_c0_g1::TRINITY_DN14128_c0_g1_i4::g.75891::m.75891 Tja_CIIMET_08	>Tcham_CIIMET_14<br>GSIYDAYCDSEAGEKLQNCLHDNVSQECWSGLLECLAKLNLPADPTTLRNFLCDPTKLKEVGKWYLCVLQTLSKSQIEELHDKTQKCFTET	10314.71
>TRINITY_DN13468_c0_g2::TRINITY_DN13468_c0_g2_i2::g.24479::m.24479 Tja_CIIMET_08	>Tcham_CIIMET_15<br>GSIYDAYCDSEAGEKLQNCLHDNVSQECWSGLLECLAKLNLPADPTTLRNFLCDPTKLKEIGKWYLCILQTLSKSQIEELHDKTQKCFTET	10342.76
>TRINITY_DN14450_c2_g7::TRINITY_DN14450_c2_g7_i1::g.16139::m.16139 Tja_CIIMET_03	>Tcham_CIIMET_16<br>NNTIMEKFCALNDAVKFYNCMNSTASIDTVNKFTKCANDQKIPLDSNERREFICDRSNSDELRLFYSCLFNSMGFDEVNDFHKSIETCLEQ	10583.83
>TRINITY_DN14553_c2_g2::TRINITY_DN14553_c2_g2_i1::g.47430::m.47430 Tja_CIIMET_03	>Tcham_CIIMET_17<br>NDNILDRFCAHTDAVKFFGCLYNTAPKESVSKFNKCAIERNVPSDSKERREFICDRNNANELRLWYLCLFKSFNPDEISDFHKSAIICLRQ	10682.12
>TRINITY_DN14418_c1_g3::TRINITY_DN14418_c	>Tcham_CIIMET_18	1091

Sequence identifier	Amino acid sequence[a]	MM*
**Alfa-amylase**		
1_g3_i2::g.18270::m.18270 Tja_Unk_59	EISLRERYCALNPIQKYYDCMKENAPEKIWNIFMGCIKKLNLPSNFNEQRKLFCDAAKDEETRKWYFCVMDELNTSELKKFHEHVEICLK	4.66
>TRINITY_DN14090_c1_g1::TRINITY_DN14090_c1_g1_i4::g.14718::m.14718 Tja_Unk_57	>Tcham_CIIMET_19 FIDNYCAYNDAGKFFDCLYNTAAKETVNKFKKCATEQNIPIESKERRDFICDRSNADQLRQGYTCLFGIMNSEEVIDFHKTVNTCMNQ	10239.50
>TRINITY_DN14553_c2_g1::TRINITY_DN14553_c2_g1_i7::g.47427::m.47427 Tja_Unk_57	>Tcham_CIIMET_20 FIDNYCAYNDAGKFFDCLYNTAAKETVNKFKKCATEQNIPIESKERRDFICDRSNADQLRQGYTCLFGIMNSEEVIDFHKTVNTCVNQ	10207.44
>TRINITY_DN14653_c5_g2::TRINITY_DN14653_c5_g2_i5::g.62160::m.62160 Tja_Unk_51	>Tcham_CIIMET_21 AGNSVFDRYCGYKDSNKYRKCVTSSVPKETWDKFSECASDKKIPIEPEEQKKFFCDASNEVKMKELYFCLHRSYDRIEILELHEHVESCLEL	10894.33
>TRINITY_DN14653_c5_g2::TRINITY_DN14653_c5_g2_i8::g.62162::m.62162 Tja_Unk_51	>Tcham_CIIMET_22 AGNSVFDRYCGYKDSNKYRKCVTSSVPKETWDKFSECASDKKIPIEPEEQKKFFCDASNEVKMKELYFCLHRSYDRIEILELHEHVEVNFDINN	11165.54
>TRINITY_DN14668_c8_g1::TRINITY_DN14668_c8_g1_i2::g.62238::m.62238 Tja_Unk_59	>Tcham_CIIMET_23 EETSVHDRYCSLNPIEKYYDCMKKNAPEKTWNTFNGCINKLNLPSDFNEQRKFFCDAAKDDETRKWYFCVMDELNTSELKKFHEYVEICLK	10992.40
>TRINITY_DN14375_c2_g1::TRINITY_DN14375_c2_g1_i1::g.5594::m.5594 No Match	>Tcham_CIIMET_24 KDPRYLPTLFSNVKPSAREDVYNSKESDDNSRSNLKLSDTTRKLPPYGVIRNK	6111.83
**Hypotensins (NDBP)**		
>TRINITY_DN14599_c0_g1::TRINITY_DN14599_c0_g1_i8::g.46295::m.46295 Tja_Unk_72	>Tcham_NDBD_01 MSLEDEQESVQRADDDLEGFSEEDLKAIKEHMAKNAGRFDPAV	4852.25

Sequence identifier	Amino acid sequence[a]	MM*
**Alfa-amylase**		
>TRINITY_DN14599_c0_g1::TRINITY_DN14599_c0_g1_i3::g.46290::m.46290 Tja_Unk_73	>Tcham_NDBD_02 LEDEQESVQRKENDLMGFSEQIMKLIKESMAKNAGRFDRAVFER	5204.88
>TRINITY_DN14599_c0_g1::TRINITY_DN14599_c0_g1_i7::g.46292::m.46292 Tja_Unk_73	>Tcham_NDBD_03 LEDEQESVQRKENDLMGFSEQIMKLIKESMAKNAGRFDPAV	4713.33
>TRINITY_DN14114_c2_g2::TRINITY_DN14114_c2_g2_i5::g.73104::m.73104 Tja_NBDP_18	>Tcham_NDBD_04 FFSTILNIGRKLIPGVVKLFSRK	2634.25
>TRINITY_DN14114_c2_g2::TRINITY_DN14114_c2_g2_i7::g.73105::m.73105 Tja_NBDP_18	>Tcham_NDBD_05 FFSTLFNIGRKLIPGVVKLFSRKKERSLMKRDLKYSYDPYQRSVEMERLLK	6213.40
>TRINITY_DN14114_c2_g2::TRINITY_DN14114_c2_g2_i1::g.73106::m.73106 Tja_NBDP_18	>Tcham_NDBD_06 FFSTLFNIGRKLIPGVVKLFSRKKERSLMKRDLKNSYDPYQRSVEMERLLK	6164.33
>TRINITY_DN14114_c2_g2::TRINITY_DN14114_c2_g2_i6::g.73108::m.73108 Tja_NBDP_18	>Tcham_NDBD_07 FFITLFNIGRKLIPGVVKLFSRKKERSLMKSDLKNSYDPYQRSVEMERLLK	6121.30
>TRINITY_DN14114_c2_g2::TRINITY_DN14114_c2_g2_i4::g.73103::m.73103 Tja_NBDP_18	>Tcham_NDBD_08 YFSTLFNIGRKLIPGVVKLFSRKKERSLMKRDLKNSYDPNQRSVEMERLLKELPLY	6746.98
>TRINITY_DN14441_c3_g2::TRINITY_DN14441_c3_g2_i2::g.17616::m.17616 Tja_NBDP_18	>Tcham_NDBD_09 STLFNIGRKLIPGVVKLFSRKKDRSLMKRDLKNSYDPYQRSVEMERLLKELPLY	6471.68

Sequence identifier	Amino acid sequence[a]	MM*
**Alfa-amylase**		
>TRINITY_DN13881_c0_g1::TRINITY_DN13881_c0_g1_i1::g.10602::m.10602 Tja_NBDP_18	>Tcham_NDBD_10 IIGRKLIPGVVKLFSRKKERSLMKRDLKNSYDPYQRSVELERLLKELPLY	6018.21
>TRINITY_DN14114_c2_g2::TRINITY_DN14114_c2_g2_i3::g.73107::m.73107 sp|A0A1D3IXJ5|NDB32_TITOB Antimicrobial peptide ToAP2 OS=*Tityus obscurus* OX=1221240 PE=1 SV=1	>Tcham_NDBD_11 FFITLFNIGRKLIPGVVKLFSRKKERSLMKRVLKN	4178.19
Chitinanse		
>TRINITY_DN13370_c0_g1::TRINITY_DN13370_c0_g1_i1::g.50777::m.50777 TaPBC_Chitinase_01	>Tcham_Chitinase_01 FECKKNGYFQDPDDCQKFYICDNGKAIQQTCGIGTVFNEKSKVCDFPANVKCSG	6033.81
Potassium Channel Toxins		
>TRINITY_DN14187_c9_g1::TRINITY_DN14187_c9_g1_i4::g.75296::m.75296 TaPBP-KTx_01	>Tcham-KTx_01 ITLASCGLREKHVQSLVSMIPDETLRNILKAHVHKEAKTQFGCPAYGGYCDTHCQDIEKHEGKCHGFKCKCEKS	8288.55
>TRINITY_DN14187_c9_g1::TRINITY_DN14187_c9_g1_i2::g.75299::m.75299 TaPBP-KTx_01	>Tcham-KTx_02 ITLASCGLREKHVQSLVSMIPDETLRNILKAVVHKVAKTQFGCPAYGGYCDTHCQDIEKHEGKCHGFKCKCEKS	8220.56
>TRINITY_DN14392_c0_g4::TRINITY_DN14392_c0_g4_i2::g.3856::m.3856 TaPBP-KTx_04	>Tcham-KTx_03 GKGKEFLGKINNKLNDVKENAKAGWEKLTSKSEYACPVIDKFCEDHCAAKIAIGKCDDFKCECLKS	7312.45

Sequence identifier	Amino acid sequence[a]	MM*
**Alfa-amylase**		
>TRINITY_DN14345_c5_g2::TRINITY_DN14345_c5_g2_i1::g.4012::m.4012 Tja_KTx34	>Tcham-KTx_04 TFINHKCRGSPECLPACKAAIGKASGKCINSQCKCYY	3994.71
>TRINITY_DN14508_c0_g2::TRINITY_DN14508_c0_g2_i18::g.45828::m.45828 TaPBP-KTx_06	>Tcham-KTx_05 QRDLEDVSCVPGDDCLEPCKKAIGSTKGKCINDRCTCYP	4261.86
>TRINITY_DN12896_c0_g1::TRINITY_DN12896_c0_g1_i1::g.71911::m.71911 Tja_KTx09	>Tcham-KTx_06 CSTCLDQPICGASRECYDPCYKAFGRAHGKCMNNRCRCYT	4526.20
>TRINITY_DN14508_c0_g2::TRINITY_DN14508_c0_g2_i5::g.45833::m.45833 Tja_KTx45	>Tcham-KTx_07 QPVLEYVSCTRSSECVDPCIAAYKTRGSKCQNRKCKCYPR	4572.31
>TRINITY_DN14508_c0_g2::TRINITY_DN14508_c0_g2_i7::g.45821::m.45821 Tja_KTx45	>Tcham-KTx_08 QRGIDVTCTSRNQCIDPCMAAYKTRGSKCQNGKCKCYLNIA	4543.28
>TRINITY_DN14218_c0_g5::TRINITY_DN14218_c0_g5_i1::g.59104::m.59104 TaPBP-KTx_06	>Tcham-KTx_09 QYDTHVKCSESSECLEVCKEDYGYRINKCRNGRCTCY	4384.89
>TRINITY_DN14508_c0_g2::TRINITY_DN14508_c0_g2_i15::g.45843::m.45843 TaPBP-KTx_53	>Tcham-KTx_10 ERPRPLNVTCITPLYCRDACIGCFGYYGLECRNGYCVCHP	4557.31
>TRINITY_DN14442_c2_g5::TRINITY_DN14442_c2_g5_i5::g.17242::m.17242 Tja_KTx05	>Tcham-KTx_11 YFEMKVDATGPQTTCQAAICEEGCKGLGKSMESCQGDTCKCKA	4552.21

Sequence identifier	Amino acid sequence[a]	MM*
**Alfa-amylase**		
>TRINITY_DN13470_c0_g1::TRINITY_DN13470_c0_g1_i1::g.23065::m.23065 Tja_KTx40	>Tcham-KTx_12 VIIGQRCYRAPDCASACKIAVGKPTGKCTNGRCDC	3659.31
>TRINITY_DN14508_c0_g2::TRINITY_DN14508_c0_g2_i3::g.45825::m.45825 sp|P84777|KA156_TITDI Potassium channel toxin alpha-KTx 15.6 OS=*Tityus discrepans* OX=57059 PE=1 SV=2	>Tcham-KTx_13 QHNINVACTSTRQCIDPCIGKYKNRESKCLNGKCKCYP	4318.02
Sodium Channel Toxins		
>TRINITY_DN13324_c0_g1::TRINITY_DN13324_c0_g1_i1::g.49375::m.49375 Tja_NaTx03	>Tcham_NaTx01 KEGYPMDHEGCKFSCFIRPSGFCEKYCKTHLSASTGYCAWPACYCYGVPEGQKVWDYYNNKC**<u>GK</u>**	7152.12
>TRINITY_DN12913_c0_g1::TRINITY_DN12913_c0_g1_i1::g.30036::m.30036 Tja_NaTx32	>Tcham_NaTx02 KKDDYPVDTAERNCMFDCNIMNDKGYCDNLCKERKADSGYCYKLKASCYCYGLPDDSPTKKSGRCNPNLR	8034.07
>TRINITY_DN14084_c7_g1::TRINITY_DN14084_c7_g1_i5::g.13702::m.13702 TaPBP-NaTx31	>Tcham_NaTx03 GEVRNGYPILKDGCKYTCSPLGETPRCSKICKEKAGSWYGYCYAWGCYCTKVSKETVLFGDSGAPECHVWIG	7952.11
>TRINITY_DN14264_c2_g2::TRINITY_DN14264_c2_g2_i1::g.64274::m.64274 Tja_NaTx16	>Tcham_NaTx04 NKDGYLLDGRCKMGCLTRKGSYCVDQCKEVKGKDGYCYAWLACYCYDMPDSVKIWNYNNNTCGQ**<u>GK</u>**	7354.39
>TRINITY_DN14469_c7_g6::TRINITY_DN14469_c7_g6_i7::g.15878::m.15878 Tja_NaTx01	>Tcham_NaTx05 GGKEGNPLNKSNGCKSGCFFGTNSWCDSECKRKSAEKGYCAWPSCYCYGFTDESKMWNAKTNKC	7094.94

Sequence identifier	Amino acid sequence[a]	MM*
**Alfa-amylase**		
>TRINITY_DN14469_c7_g6::TRINITY_DN14469_c7_g6_i3::g.15898::m.15898 Tja_NaTx01	>Tcham_NaTx06<br>EGYPFNKSNGCKSGCFFGTNSWCDSECKRKSAEKGYCAWPSCYCYGFTDESKMWNAKTNKC	6935.75
>TRINITY_DN14174_c0_g1::TRINITY_DN14174_c0_g1_i7::g.72816::m.72816 Tja_NaTx31	>Tcham_NaTx07<br>KKDGYPVEGTCAFPCGYDNAYCDKLCKERKADSGYCYWVNILCYCYGLPDNAAIKGYGICKP**GKK**	6919.94
>TRINITY_DN14293_c0_g2::TRINITY_DN14293_c0_g2_i2::g.59839::m.59839 Tja_NaTx18	>Tcham_NaTx08<br>GYPLSKNNYCKIYCPDDTVCKDTSKRRAGATNGKGDCKSYGCYCYDVAPGTKMYPDPRLPCNPY	7128.08
>TRINITY_DN14537_c1_g1::TRINITY_DN14537_c1_g1_i3::g.48584::m.48584 Tja_NaTx43	>Tcham_NaTx09<br>SKTGYVVTQKGCIYSCFWGSSWWCNKECTALGGTSGYCAWPSCWCYALPDNRKIWGDGPNNC**GKK**	6911.80
>TRINITY_DN14306_c2_g1::TRINITY_DN14306_c2_g1_i4::g.5771::m.5771 Tja_NaTx43	>Tcham_NaTx10<br>SKTGYVVTQKGCKYSCFWGSSWWCNKECTALGGTSGYCAWPSCWCYALPDNRKIWGDGPNNC**GKK**	6926.82
>TRINITY_DN14573_c2_g4::TRINITY_DN14573_c2_g4_i4::g.48553::m.48553 TaPBP-NaTx29	>Tcham_NaTx11<br>KEGYLLGSGGCKMNCLTRPGNYCELECSLVGGENGYCANWLACYCYNVPDSVTLWESDANDC**GKRK**	6754.55
>TRINITY_DN14400_c1_g1::TRINITY_DN14400_c1_g1_i1::g.17495::m.17495 TaPBP-NaTx30	>Tcham_NaTx12<br>RDAYPALWNGCKYSCFWGSTSWCNEECSSLGASSGYCAWPACWCYDLPDNVRYWDSNNNKCR**GK**	7162.87
>TRINITY_DN13192_c0_g1::TRINITY_DN13192_c0_g1_i1::g.49048::m.49048 Tja_NaTx09	>Tcham_NaTx13<br>DKNGYPVIEGAGSPDYGGSAECGSDDSNSEDNFCNNVCTNVGGKSGDCCLGTCFCFDLPDKQKTVEIMDRTKEYCEFVE	8481.19

Sequence identifier	Amino acid sequence[a]	M M*
**Alfa-amylase**		
>TRINITY_DN14432_c1_g1::TRINITY_DN14432_c1_g1_i2::g.17392::m.17392 Tja_NaTx51	>Tcham_NaTx14 DEGEALDGYPLSKVNNCKIYCPDNDTCKWTCENRAGATNGKGTCIWKSCFCYDVAPGTKMYPGDRPCIT	7590.55
>TRINITY_DN14573_c2_g7::TRINITY_DN14573_c2_g7_i1::g.48561::m.48561 TaPBP-NaTx39	>Tcham_NaTx15 KDGYLMGSDGCKLNCLMKKGTFCADECKRVKGKDGYCYAWLSCYCYN	5334.23
>TRINITY_DN14677_c0_g4::TRINITY_DN14677_c0_g4_i11::g.61083::m.61083 Tja_NaTx04	>Tcham_NaTx16 IYCPDDAVCKESCKIRAGATNGKGDCKSHGCYCYDVAPGTKMYPDPRFPCNPY	5810.61
>TRINITY_DN14256_c3_g3::TRINITY_DN14256_c3_g3_i1::g.66510::m.66510 Tja_NaTx18	>Tcham_NaTx17 LDGYPLSKNNYCKIYCPDDTVCKDTCKRRAGATNGKGDCISYGCYCYDVAAGTKMYPDPRLPCYPY	7380.41
>TRINITY_DN14379_c2_g2::TRINITY_DN14379_c2_g2_i11::g.5476::m.5476 Tja_NaTx48	>Tcham_NaTx18 IKNGYPRDSKGCTFQCGQDAKHGNDYCDKMCKTTLKGEGGDCDFEYAECWCDNIPDTVVTWKNKEPKCL	7803.75
>TRINITY_DN13934_c1_g1::TRINITY_DN13934_c1_g1_i2::g.67165::m.67165 Tppa2	>Tppa2 KDGYLVGNDGCKYSCLTRPGHYCASECSRVKGKDGYCYAWMACYCYNMPNWVKTWSRATNKC**GKRK**	7108.13
>TRINITY_DN14306_c1_g1::TRINITY_DN14306_c1_g1_i4::g.5753::m.5753 TaPBP-NaTx30	>Tcham_NaTx20 RDAYPALWNGCIYSCFWGSTSWCNEECSSLGASSGYCAWPACWCYDLPDNVRYWDSNNNKCR**GK**	7147.86
>TRINITY_DN14306_c1_g1::TRINITY_DN14306_c1_g1_i3::g.5758::m.5758 TaPBP-NaTx30	>Tcham_NaTx21 TRNAYPALWNGCIYSCFWGSTSWCNEECSSLGASSGYCAWPACWCYDLPDNVRYWDSNNNKCR**GK**	7247.98

Sequence identifier	Amino acid sequence[a]	MM*
**Alfa-amylase**		
>TRINITY_DN14174_c0_g1::TRINITY_DN14174_c0_g1_i4::g.72819::m.72819 sp|C9X4K6|SCNA8_TITDI Toxin TdNa8 OS=*Tityus discrepans* OX=57059 PE=1 SV=1	>Tcham_ NaTx22<br>ESGTESKKDGYPVKDGDCAFPCGYDNAYCDKLCKERKADSGYCYWVNILCYCYGLPDNAAIKGYGICKP**<u>GKK</u>**	7638.62
**Lectin**		
>TRINITY_DN14589_c0_g1::TRINITY_DN14589_c0_g1_i5::g.48616::m.48616 TaPBC_Lectin_16	>Tcham_Lectin_01<br>QVSSNLRIIDDLLHKAMERNTGTNTGTNPGPIDCKQIQDNGNKISGIYRIWPLNWKKIGSFFVYCDMQIDGGGWTVIQRRGNYRQPKDYFFKNWEQYAIGFGKLNEDFWLGNDRIFSMTNQGNYSLRIDMKDLEGNKRYAVYDKFWIENERQQYKLHVSGFKGDAGDSMAYVNEMKFSTKDRKNDIWTKNCAEAYKGGWWYAKCHHANLNGLYLNGSHSTYADGIEWRTWKGFYYSLAEVDMKIRRKV	29142.83
>TRINITY_DN14589_c0_g1::TRINITY_DN14589_c0_g1_i1::g.48623::m.48623 TaPBC_Lectin_16	>Tcham_Lectin_02<br>HFPVSEVTSNLRIIDGLLQKVIEFVEKNKGTYPGPIDCKQIQNNGNKISGIYRIWPLNWETIGSFLVYCDMEIDGGGWTVIQRRGNYKQPKDYFFKNWEQYAIGFGKLNEDFWLGNERIFSMTNQGNYSLRIDMKDKDGNKRYALYDDFWIENERQLYKLHVFGFTGDAGDSFTYMNEMKFNTKDKDNDNWNNNCAETYKGGWWYGKCHRANLNGLYLNGPHSSYADGIEWKTWKGFHYSLSEVDMKIRRKV	29721.38
>TRINITY_DN14589_c0_g1::TRINITY_DN14589_c0_g1_i3::g.48611::m.48611 TaPBC_Lectin_16	>Tcham_Lectin_03<br>QVSSNLRIIDDLLHKAMARNTGMNTGTNPGPIDCKQIQDNGHKTSGIYRIWPLNWKKIGSFFVYCDMQIDGGGWTVIQRRGNYRQPKDYFFKNWEQYAIGFGKLNEDFWLGNDRIFSMTNQGNYSLRIDMKDKDGNKRYALYDKFWIENERQQYKIHVSGFKGDAGDSITYMNEMKFSTKDRRNDIWTKNCAETYKGGWWYAKCHAANLNGLYLNGPHDSYGDGIEWKTWKGFHYSLSEVDMKIRRKV	9148.86
>TRINITY_DN14589_c0_g1::TRINITY_DN14589_c0_g1_i17::g.48606::m.48606 TaPBC_Lectin_16	>Tcham_Lectin_04<br>QVSSNLRIIDDLLHKAMERNTGTNPGPIDCKQIQDNGNKISGIYRIWPLNWETIGSFFVYCDMQIDGGGWTVIQRRGNYRQPKDYFFKNWEQYAIGFGKLNEDFWLGNDRIFSMTNQGNYSLRIDMKDLEGNKRYAVYDKFWIENERQQYKLHVSGFKGDAGDSMAYVNEMKFSTKDRKNDIWTKNCAEAYKGGWWYAKCHHANLNGLYLNGSHSTYADGIEWRTWKGFYYSLAEVDMKIRRKV	28743.34
>TRINITY_DN14589_c0_g1::TRINITY_DN14589_c0_g1_i14::g.48597::m.48597 TaPBC_Lectin_17	>Tcham_Lectin_05<br>MARNTGMNTGTNPGPIDCKQIQNNGNKISGIYRIWPLNWETIGSFLVYCDMEMDGGGWTVIQRRGNYRQPKDYFFKNWEQYAIGFGKLNEDFWL	10978.40
>TRINITY_DN14355_c0_g1::TRINITY_DN14355_c0_g1_i12::g.4706::m.4706 TaPBC_Lectin_31	>Tcham_Lectin_06<br>QNFSNLQIIDDLLHKAMEKNTGTCPGPVDCKQIQDNGNKISGIYRIWPLNWRTSGSFFVYCDMQTDGGGWTVIQRRGNYKQPKDYFFKNWEQYAIGFGKLNEDFWLGNNKIFSMTNQGNYSLRIDMKDLEGNKRYALYDNFWIENERQQYKLHVSGFKGDAGDSITYMNEMKFSTKDRKNDIWKKNCAEAYKGGWWYVKCHRANLNGLYLNGTHSTYADGIEWKTWKGLNYSLPEVDMKIRRKV	28716.41
**Metaloproteases**		

Sequence identifier	Amino acid sequence[a]	MM*
**Alfa-amylase**		
>TRINITY_DN14602_c0_g2::TRINITY_DN14602_c0_g2_i7::g.60863::m.60863 Metallojaimease127	>Tcham_Metallochampionase_01<br>HIVIGPPGTRRISTLLVGILLLRNPIMLGLAFLINVSVLIGVSAIRSGREEIVFPSMETFRSGEKRLSFRALNQDIELKLEPAGYLFGEGFFVESVHDGKSEIPELNIEDIKRSLYKDKRTGASLHIEDKERILINGIINDNLRIEPIKSEKERRDGAIPHRIYEIISDDNLRINDAVFPTDYHPVYNGRLKQLKAEEPIYLEIGIITDSAFSLSFPDTESLLSYLSVLVNGAQCRYDNIKSIKVTLVLASVKINKSTKEEPYIQNNIREEYVMIYDALNSFSVAMGKAAYPSDIIMLVSKREMGMIKNGTIDTSINGIAYVGGACAKQSARGTIRVGISHDKGHSFGSAKLFAHECAHLLGCPHDGEGPDPYLPGHPGSSKCPWSDGYMMSYVDNTKNRNYFSSCCEENIRYLANRVDCLRKNNTGYNIKSTNQKPGEYMESVLGLSASNYAKDKVPYYNDRCIKLYQPNATSFRYPDGKCEFVCRSTIKKSFRTIKCLDGEMCDVGKRCSNGMCQ	57879.28
>TRINITY_DN14297_c0_g2::TRINITY_DN14297_c0_g2_i5::g.59942::m.59942 Metallojaimease03	>Tcham_Metallochampionase_02<br>GVKIVKFRALGEDVELKLEPAGDILAEDFAIYNSNNEKQHTVDVESLRRRMYRDDFNAAVLLIDDVEEIPSIEGTISYKFKITPHEWKGMTRNGRRAHQIEEIIMDESFLVRDSVIPSDFQTEIRNLTRLDKDKECIVVEYLIATESAFTEAHKTDKELTEYVTSIVSGAEGMYRRSNLKTKLRLLGIQAFTEEDEPDFIKASLNKDGKYYSLQSSMKGLNKYYCENPTDLSIKADIIFLFTKRSLRDDKNKAVDAGVMLNSGACQKCNKIGVVADSSNYLQAMITLAHETAHGIGVPHDGDSTKTISLPHGPGALSCKYKDGYIMGTSSGVNSLYFSKCSKDCMAYFLSLPNSTCLHEKC	40499.01
>TRINITY_DN14413_c1_g2::TRINITY_DN14413_c1_g2_i3::g.15175::m.15175 metalloasthenase19	>Tcham_Metallochampionase_03<br>IPNGQTEVTYPSLETLERGIKIMKIRAFGEDLELKLEPAGDVISDDFTVVDGEGNIQDKDATFLKKRLFRDEEKGSALYIKETGSMEIDGVINSELRIEPYKSQEEGRNEFNAHLVTKILPEVRSFSDAVINVHLERLLSERNDTDDECLEIEYLFMAESNFAVIFQNSVQLQAYLGTLSIQVQILMDRLNLNLKTRLIGTMLYKDNPPFFQGSLIPGQDIIDANSIMNNITNFLCTDQGNELIKRADVIMLITTKPIGVSYPLWFVNPGLLGISNLGGACDLCKKCAVIKDSGRMLLTAYVIAHETAHLIGSPHDGVGPMFGLPGSPGSVECPPLAGFIMGNNFGVTTSMFSPCSRENIKYFLSKNEASCIKSCNSTTML	42125.32
>TRINITY_DN14413_c1_g2::TRINITY_DN14413_c1_g2_i6::g.15190::m.15190 metalloasthenase19	>Tcham_Metallochampionase_04<br>IPNGQTEVTYPSLETLERGIKIMKIRAFGEDLELKLEPAGDVISDDFTVVDGEGNIQDKDATFLKKRLFRDEEKGSALYIKETGSMEIDGVINSELRIEPYKSQEEGRNEFNAHLVTKILPEVRSFSDAVINVHLERLLSERNDTDDECLEIEYLFMAESNFAVIFQNSVQLQAYLGTLSIQVQILMDRLNLNLKTRLIGTMLYKDNPPFFQGSLIPGQDIIDANSIMNNITNFLCTDQGNELIKRADVIMLITTKPIGVSYPLWFVNPGLLGISNLGGACDLCKKCAVIKDSGRMLLTAYVIAHETAHLIGSPHDGVGPMFGLPGSPGSVECPPLAGFIMGNNFGVTTSMFSPCSRENIKYFLR	40482.44
>TRINITY_DN14075_c1_g2::TRINITY_DN14075_c1_g2_i6::g.16841::m.16841	>Tcham_Metallochampionase_05<br>IPSGRTDIVFPSLETSRSGVKIIKFATLGQDIELKLKPAGEILAKDFAIDINNQKEHLTNLEDLKRKIYRDSAKGAALLIDENGPLTLKGIINSNLRILPYESGRVIKDGKIAHQIVEIINDKRSYINDVIPLDANREMENVVRIAGKKKCIVIDYLCVTESTFTQRFAKTKDLVEYITILYTGVQNLLDTLNLQIKAHLIGITAIEKANEPSFINESAIPSDPLVLDPENLVRKMARHYCKITTGQPKDADIIMLITNRKLGELRGDGTIAYNTAGISLAGGVCKQCGKVGLAQDDTDYNERVDTVAHETAHLIGAPHDGEEPFLSGSPGAKNCPESDGYIMGGNGNKANKSKFSNCTKECVEYLFSLQSASCVLVDC	41519.61
>TRINITY_DN14500_c3_g1::TRINITY_DN14500_c	>Tcham_Metallochampionase_06<br>TGRENVVFPSVETLRSGVKTIKFRAFGEDVKLKLEPAGGILSKDFAIYNGNNEKDHSVDVESLRRRIYRDSANGAALVIDDNDEQSLSIRGVVNSKLRIEPHKLEGTTQYGRKAHRIIELTSDGNSHYSDSIILPKSEIEARNLTRMSRADKCIVVEFLCLTESKFTERFKTNKELTDYVTT	4245

Sequence identifier	Amino acid sequence[a]	MM*
**Alfa-amylase**		
3_g1_i2::g.44839::m.448<br>39 metalloasthenase129	MYTGVQNMYDTMNLGIVIRLSGIQAFTKENEPSYIKDSEVLKGQYLDGKVLANMRSYYCKNSVGLAKDADIIMLIVRRVLVGLTGSKI<br>TSKALGIAYGAGLCHKCDKVGVTLDESDYNERIITLAHESAHLLGVPHDGMGTEEVDLKDGPGAKSCPYDDGYIMGTAEEDNMLKFS<br>KCSAACAKYFLSLPQASCAIEDCKSSH	9.2<br>9
>TRINITY_DN14500_c3_<br>g1::TRINITY_DN14500_c<br>3_g1_i3::g.44843::m.448<br>43 metalloasthenase129	>Tcham_Metallochampionase_07<br>TGRENVVFPSVETSRSGVKTIKFRALGEDIELKLEPAGAILSKDFALYNGNKEEDHSVDVESLRRRIYRDSVKGAALVIDDDHEQSPSIR<br>GVVNSKLRIEPHELGGTTKYRRNAHRIIELTSEGNSYFSDNVIPPKSESEARNLTRMSRADKCIVVEFLCLTESKFTERFKTNKELTDYVT<br>TMYTGVQNMYDTMNLGIVIRLSGIQAFTKENEPSYIKDSEVLKGQYLDGKVLANMRSYYCKNSVGLAKDADIIMLIVRRVLVGLTGSK<br>ITSKALGIAYGAGLCHKCDKVGVTLDESDYNERIITLAHESAHLLGVPHDGMGTEEVDLKDGPGAKSCPYDDGYIMGTAEEDNMLKF<br>SKCSAACAKYFLSLPQASCAIEDCKSSH	42<br>50<br>1.2<br>4
>TRINITY_DN14326_c0_<br>g6::TRINITY_DN14326_c<br>0_g6_i4::g.4424::m.4424<br>Metallojaimease109	>Tcham_Metallochampionase_08<br>DNDIATPMEIEKQAENLLSMGRQDKCLVLEILCVTESKFTERFKTDEALTEYITTLFTGTENLLKGLDSRIKVRLIGIDARKNGKNPAYIK<br>QSEYNDTYIDLVTILVNMKKYYCKKMNELEKSADLIMLIVTREMVKVVDNKIVKRIVGKAAAPNVCECKKVAVSQDESDYNERVDTV<br>AHEIAHMLGVPHDGIGTEEVNLPNGPGAKDCPPNTGFI	24<br>18<br>4.8<br>8
>TRINITY_DN14567_c2_<br>g1::TRINITY_DN14567_c<br>2_g1_i11::g.48264::m.48<br>264 metalloasthenase13	>Tcham_Metallochampionase_09<br>IPSGRVELVFPSVETSRSGVKTIKFTALDQNVELKLTAAGEILGKHFDVEGVDVEKLRRKMYRDGANGAALLIDEDGPLAIEGIINSKLRI<br>EPHESGRIIKNGVIAHQIVELINDIKSYNNDTVMPKDIKRETENMARMGRDGDCIVVEYYIVTEKVFTKRFKSNKALTEYVTVMFTGV<br>QNLMDSLELGIEVRLLGVTPFNAKTQPPFIEATVYPGHDAMDPDQLVSSMGDYYCKHQTGLAKDADIIMLLNGRGMGDMMDDG<br>TVQVNTAGIASSACVCKCNKVGVATDDSKYNERVDTVAHESVHLLGSPHDGEKSNLVSLNGSPGAANCPASAGYIMGNRNKENGF<br>KFSECTKECVEYLLSKPFASCVYEECS	40<br>89<br>3.5<br>6
>TRINITY_DN14208_c2_<br>g2::TRINITY_DN14208_c<br>2_g2_i1::g.66459::m.664<br>59 metalloasthenase08	>Tcham_Metallochampionase_10<br>IPNDRSEVTHPSLETLRSGIKVMKFRAFGRHIELKLEPAGDILSDDFVVTDSNGNIRVTDVKSLKSKVYKDQENEAALYIDHDGFLEINGI<br>INSKLRIEPYESEEMVKNGIKAHIITETAAKRKPVNDAEFTIKMEKSLVEVVRNFDESQCVHIKYLCVMDNTFTSQFGSDYEIQSFLAGIF<br>LKVQNIMDTLNLKIKVTVIGSTQLIDDTQKYIRQSLIQGLETYNVAHIIQNVTKFYCHQEKDNLVKQANIVMFFTKRALGDPRPDGTIF<br>DAIVGVANLGGACNPCEKCGVIHAEKKNMLGAADTVAHETAHLIGSPHDGEGPERSVDGSPAALSCSGKLGYIMGDDYGKNEKKFS<br>SCSRANIVYFLGLSRANCIIKSCSN	42<br>35<br>0.3<br>3
>TRINITY_DN14406_c1_<br>g2::TRINITY_DN14406_c<br>1_g2_i20::g.18700::m.18<br>700<br>metalloasthenase109	>Tcham_Metallochampionase_11<br>VPTGRVEVVFPSVETSRSGVKTVKFRALDQDIELKLEPAGEILAKDFVFVNSKQPFDVEKLRRKIYRDIVNGAALLIDENGPSSIKGIVNS<br>NLRIEPHESGRMIDGIRAHQIVEIRRDERSYYNDIVTPMEIEKQEENFLSMGRKDKCLVIEILCVTESRFTERFETEAALSEYLTSLFNGAE<br>DLLKGLDSRIQLNLIGIDARKKGQSEDLSYIKESEYNNTYINDNILYKMKDYYCKEMNELERRADIIILITHRRLIEIEDNQFIDWTDGIAM<br>APDVCKCSKVAVSTDNSNYNERVDTLAHEIAHVIGVPHDGDGTEILKLPHGPGAKDCPESDGFIMGDPNDANKFKFSQCSKRCARYL<br>LSLPRASCVFEDC	42<br>61<br>4.3<br>8
>TRINITY_DN14444_c2_<br>g3::TRINITY_DN14444_c	>Tcham_Metallochampionase_12<br>SNFTERFETEQALTEYVTLMYSATETLLRQLDSRLQLRLSGIQAYTKESEPPFIKDNELEEGKYYNARGVLRAVRDHLCDEYNYYFHND<br>GSTPTKRADIVMLLITRSMMFLKPDGSSDGFPAGIAYIGTVCSYCYKVGVTRDYGNAVSGSTLLAHEISHLLGVPHDGV	18<br>90

Sequence identifier	Amino acid sequence[a]	MM*
**Alfa-amylase**		
2_g3_i7::g.18296::m.18296 metalloasthenase05		9.27
>TRINITY_DN14586_c1_g1::TRINITY_DN14586_c1_g1_i2::g.47219::m.47219 metalloasthenase109	>Tcham_Metallochampionase_13<br>IELRLEPAGNILAKDLLFVNSKQPFDVEKLRRKIYRDIVNGAALLIDENGPSSIKGIVNSNLRIEPHESGRQIGDGIRAHRIVEIRSDQVSYDNDIATPMEIEKQAENLLSMGRQDKCLVIEILCVTESKFTERFKTDEALTEYITTLFTGTENLLKGLDSRIKVRLIGIDARKKGQDLPYIKQSEYNDTYIDLVTILTNMKKYYCKKMNELEKSADLIMLIVTRELIKVVDNKIVQRLVGQAMAPYVCECKKNAVSLDESDYNERVDTVAHEIAHMLGVPHDGIGTEEVNLPNGPGAKDCPPSTGFIMGNRNDANQFKFSECSKKCAKYLLSLPR	37882.59
>TRINITY_DN14295_c2_g2::TRINITY_DN14295_c2_g2_i2::g.62101::m.62101 metalloasthenase110	>Tcham_Metallochampionase_14<br>NRIDLVFPNVETSRSGVKTIKFRALDQDVELDLQPAGKILAKDFGFVDENHKLYHPVNVRNLKSKLYRNSAEEAALLIDEDGPLTIEGIINTKLRIAPHESGRMDEDGRIAHQIVEETSERKSYFHDAVMPVNVEIEEEIIKRMDRDGKCIVIEFLSVLESNFTKRFSNDDALTKHMSVTYMGVQNQLEKLELGIQVRLLGIHAFTNETEPSFIEDSAIPGYEKYLEMNKILRNMGKYYCKQANGLGKDADIIMLTTDRPLAKSISDNKINKNIAGVARPSGVCNYCNKVGAGEHYDDFAISVHIITHEAAHLIGIPHDGEGPKSIALSDSPGAFNCSFKDGYFMGNFYNKINHDKFSECSKACARYLLSLPRADCLYENCINDDDK	43530.36
>TRINITY_DN14639_c1_g2::TRINITY_DN14639_c1_g2_i5::g.59357::m.59357 metalloasthenase116	>Tcham_Metallochampionase_15<br>TGRVEVVFPTVETSRSGIKTVKFRALDQDIELRLEPAGNILAKDFLFVNSKQPFDVEQLRRKIYRDRVNGAALLIDENGPSSIKGIVNSNLRIEPHESGRMIDGIRAHQIVEIRRDERSYYNDTITPMEIEKQAENWLSTGRQDKCLVIRILCVTEGEFTRKFDSFEYMKTLYLEPHFNEAESLLKQLDSRIKLKLIGVDARKMGEDLPYITESEYIEDNETYINNDIIFQMKKYYCKEMNEIERKADIIMLITERQLIEKLSNGSTHWSDGKAILTSVCSCSKGSVVLDTLNYYETANTIAHEIAHALGVPHDGEGIEDLDLPDSPGALDCPKSSGYIMGDPNEANEYKFSECSKRCARHLLSLPRASCVFEDC	42752.51
>TRINITY_DN14199_c0_g2::TRINITY_DN14199_c0_g2_i2::g.74071::m.74071 Metallojaimease80	>Tcham_Metallochampionase_16<br>QREKVNFTRIDRDDKCLVIELLFVTDRKFTERYETNEALTEYVTLMYSSAETILRQLDSGLQLRLSGIVAFTKETEPALFKKNELKDGEYYSKLILPEIKNYFCRNPITSSKSADIIMLLITRSMTTLKADGSIQGIVAGRAYSGTVCDKCNKVGVTRDFGNAVSGSMLSAHEMSHLLGVPHDGDPSKYTGVSGSPGAESCPSKDGFLMGYNRGANRNIFSKCSKDCAKYLLST	25920.60
>TRINITY_DN14326_c0_g1::TRINITY_DN14326_c0_g1_i7::g.4390::m.4390 metalloasthenase26	>Tcham_Metallochampionase_17<br>IPSGRVELVFPSVETSRSGVKTIKFTFLDQDVELKLISAGDILGKDFTIEDVNGQIKHSVDVENLRRKMYRDSANGAALLIDEDGLTTIEGIVDSKLRIKPYDSERIIKDGVIAHQIVELINDKISYFKDAVMTKEIKKETENVARMARNGECIVVEYLLVTESNFTQRFENAEAMSKYVTVMFTGVQNLIDTLEMGIKVRLIGGTSFTKESEPSFIEESSIPGHNEILDPDNLMTNMQNYFCNFINDEVVQKADLIMLILGRRMGKIRESGTLPLNVAGVSNSGSVCRPCIKIGACVDNSNYNERVDTVAHESVHILGSPHDGEGPEYLPFPGSPGSANCSASAGYILGTRNEQNGKKFSECTKKCVKYLLSLPSALCVYENCK	42558.56
>TRINITY_DN14208_c2_g1::TRINITY_DN14208_c2_g1_i3::g.66451::m.66451 Metallojaimease20	>Tcham_Metallochampionase_18<br>IPNDRSEVTYPSLETLRSGMKIMKFRAFGRDIELRLEPAGDVVSDDFVVVDGNGSVQVTDVKCLKSKLYKDEEKGAALYIDHDGSLEINGIINSKLRIEPYESEEMMKNGIKAHIITESSRKKKPVNDAEFTINMEKSVVEVVRDFDESECVLIKYLCVTDSALSSKFRNNNKGIQAFVASMFVKAQEMINTLNLNIKVILIGIIILKDDPSYMRESIIPETDTYNVRHIRLNFSNYYCHHTENKLVLRADVMILITGRALGKLQADGTVD	42569.88

Sequence identifier	Amino acid sequence[a]	MM*
**Alfa-amylase**		
	KNILGVAFLGGVCNQCTKYAVIRAEKHHPLNTADTLAHESAHLIGCPHDGVGLEHSIPGSPTGEKCPGSLGYIMGKDIGEHRGKFSSC<br>SRKNIIYFLGLSRGDCIVVECQSH	
>TRINITY_DN14638_c0_g3::TRINITY_DN14638_c0_g3_i4::g.59906::m.59906 Metallojaimease32	>Tcham_Metallochampionase_19<br>DNEQPPSLEGIVFSKLRIWPHEWNEISEDGKRVHRVEELTNDGDSYLSDNIILPDFRKEFANFAKIDRDNKSLVIEVLCVTEGDFRELYE<br>TNKDITKFVTIMYSAAETRLRQLDSGLKLRLSGIVAFTKETEPPFIKDKLNEYGMYDSSITYEFKNYYCKNSTSASKNADIIILLITRSIRIRTE<br>FGYGEAEGQAFPGMVCDKCFKVGVISASINPWLSSWVLAHEISHLLGVPHDGVIPEETGVVGSPGAEACPSTDGFFMGRNNGVN<br>WGIFSQCSKDCAQFLLSKPEASCVYEDSE	33503.77
>TRINITY_DN14421_c1_g3::TRINITY_DN14421_c1_g3_i2::g.18660::m.18660 Metallojaimease32	>Tcham_Metallochampionase_20<br>GVKTVKFRAFDKDVKLQLEPAGDILAKNFALYNGNHEKQHSVDVESLRRRLYRDRTNGAALLLDDDEQPPSIEGIVLPNLKITPHEWN<br>EITEGGKRAHRVEELTSDTNSYLFDNIILPDFRKEFANFAKIDRDNKSLVIEVLCVTEGDFRELYETNKDITKFVTIMYSAAETRLRQLDSG<br>LKLRLSGIVAFTKETEPPFIKDKLNEYGMYDSSITNEFKNYYCKNSTSASKNADIIILLITRSIRIRTEFGYGEAEGQAFPGMVCDKCFKVG<br>VISASINPWLSSWVLAHEISHLLGVPHDGVIPEETGVVGSPGAEACPSTDGFFMGRNNGVNWGIFSQCSKDCAQFLLSKPEASCVYE<br>DSE	40602.90
>TRINITY_DN14500_c3_g1::TRINITY_DN14500_c3_g1_i4::g.44849::m.44849 Metallojaimease55	>Tcham_Metallochampionase_21<br>TGRENVVFPSVETLRSGVKTIKFRAFGEDVKLKLEPAGGILSKDFAIYNGNNEKDHSVDVESLRRRIYRDSANGAALVIDDNDEQSLSIR<br>GVVNSKLRIEPHKLEGTTQYGRKAHRIIELTSDGNSHYSDSIILPKSEIEARNLTRMSRADKCIVVEFLCLTESKFTERFKTNKELTDYVTT<br>MYTGAQNLCDTMNLGIVLRLSGIQAFTKENEPSFIKDNEVLEGNYLDVSILANMDNYYCKNSTGLAKDADVIMLFVRRNLVILQGSKI<br>IAEYDGITYISKLCNECYKVGVTADESDYNERIITLAHLTAHALGVPHDGNDSEEAVVKDGPGAKACPYNDGYIMGTADGPNMLKFS<br>KCSAACAKYFLSLPQASCVIEDCKSSR	42571.23
>TRINITY_DN14567_c2_g1::TRINITY_DN14567_c2_g1_i12::g.48236::m.48236 metalloasthenase03	>Tcham_Metallochampionase_22<br>IPSGRVELVFPSVETSRSGVKTIKFTFLDQDVELKLISAGDILGKDFTIEDVNGQIKHSVDVENLRRKMYRDSANGAALLIDEDGPATIEG<br>IINSNLRIKPHQSGRIIKDGVIAHQIVELIDDKKSYKHDYVMPEDVKTETENVARMAREGECIVVEYYVVTDSRFTQRFDSDQAVTNYV<br>TVTFTEVQNKIDTMNLGIKVRLLGVTPYHKESEPSFIQDSLLSGHQEYVDARKIVHNMKVYFCKHKTGLAKTADIIMLLITRTLGVVEE<br>NSTTVSEIAGSSSISSVCQRCNNVGICIDNSDYNERADTVAHESVHLLGSPHDGEGPDDGLDVPGSPGAANCPDSDGYIMGTRNNK<br>NGKKFSECTKQCVKYLLSLPRASCVYENCSK	42689.18
>TRINITY_DN14523_c1_g1::TRINITY_DN14523_c1_g1_i5::g.47368::m.47368 Metallojaimease84	>Tcham_Metallochampionase_23<br>DSGLKLRLSGIVAFTKETEPPFFKDIVHQDGDYKSGVLTAIRDYFCKNSTSLSKNADIILLLITRYMKLLKPNGSTRAIAQGIAYAGEVCDR<br>CNKVSVIRGYMKPVDAAKTLAHEMAHLLGVPHDGESFTGVTGSPGAKSCPPKDGFFMGNNERANWGIFSKCSKDCAKYLLSKPQA<br>SCVYEECKNSRY	20714.82
>TRINITY_DN14430_c4_g1::TRINITY_DN14430_c4_g1_i2::g.17145::m.17145 metalloasthenase101	>Tcham_Metallochampionase_24<br>KRAHRVEELTSDRDSYLSDDIILPDFQREMVNFTRIERDDKCLVIELLFVTDRKFTERYETNEALTEYVTLMYSAAETLLRQLDSGIQLRL<br>SGIQAFTKETEPPFFKSNDIKDGKYYDAEILTGMKNYFCRNSTSSSKNADIIMLLITRYMMLLNPDGTPDGFAAGFAYSGTVCYRCHKV<br>GVTRDYWNVVSGSSLLAHEISHLLGVPHDGESPQDTGVFGSPGAESCPSSDGFLMGYNTGVNSEKFSQCSKDCAKYLLSKSQASCVY<br>EECKSSRY	0971.81

Sequence identifier	Amino acid sequence[a]	MM*
**Alfa-amylase**		
>TRINITY_DN14639_c1_g2::TRINITY_DN14639_c1_g2_i10::g.59348::m.59348 metalloasthenase116	>Tcham_Metallochampionase_25 VSTGRVEVVFPTVETSRSGVKTVKFRALDQDIELRLEPADEIMDKDYFNVEELRRKIYRDRVKGAALLIDENGPSSIRGIVNSNLRIEPHESGRIIDGIRAHQIVEIRRDERSYYNVTPMEIEKQAENWLSTGRQDKCLIIEILCVTEGEFTKRFESVEDIRKYLNPLFEGAEELLKQLDSRIQLKLIGIDARKMGEDLPYITESEYFESVRKKIYIHEDIIYKMKNYYCKEMNEIERRADIIMLIIARRFIKVVRGDFLDDVDGRAILTSVCNCTKGSVVMDKLSHDDTVDSIAHEIAHALGVPHDGEGIEDLDLPDSPGALDCPKSSGYIMGDPNEANEYKFSECSKRCARHLLSLPRASCVFEDC	42146.02
>TRINITY_DN14567_c2_g1::TRINITY_DN14567_c2_g1_i15::g.48261::m.48261 metalloasthenase09	>Tcham_Metallochampionase_26 IPTGQNEVVFPSVETSRSGVKTIKFTAFDQDVELKLISSGKILGKGFAMEGVDVENLRRKMYRDSANGAAILLDEDGPITIEGIVNSNLRIKPYESGRIIKNGVIAHQIVELIDDNESYINDTFIRKDIKWKTENMARMDRDGECIIVEYYVITERVFTNRFESNKALTEYVTVMFTGVQNLIDTLELDIEIRLLGVTAFDEEIEPWYVEKSVIPGYANVLNPYKLIKLMESYYCQHQTGLAKDADIIMLITARRMGFPRNDGTVVVNIAGIANSAAVCMPCYKVGISMDDSEYNERVDTVAHESVHLLGSPHDGEGSNEISIEGSPGAENCPASAGYIMGHRNDKNRFKFSECTKQCVEYLLSNPVASCIYEECGDF	42241.94
>TRINITY_DN14679_c0_g1::TRINITY_DN14679_c0_g1_i21::g.59128::m.59128 metalloasthenase101	>Tcham_Metallochampionase_27 IPTGRVEVVFPSVETSRSGVKTVKFRALGEDVELKLEPAGDILAKDFAFYNGNHEKQHSMDVESLRRRLYRDRTNGAALLIDDNEQPPSIEGFVIPNLSITPHDWKEITEDGRRAHRVEELTSDKDSFLSDNIILPDFQREMINFSRTDKDDECIVIEFLSVTESNFTDRFETNQALTEYVTLMYSAAETILRQLVSGIQLRLTEIHAYTKDTEPPFFKDNELEEG	25917.91
>TRINITY_DN14406_c1_g2::TRINITY_DN14406_c1_g2_i5::g.18707::m.18707 metalloasthenase109	>Tcham_Metallochampionase_28 VPTGRVEVVFPTVETSRSGIKTVKFRALDQDIELRLEPAGNILAKDFVFVNSKQPFDVEQLRRKIYRDRVNGAALLIDENGPSSIKGIVNLNLRIEPHESGRMIDGIRAHQIVEIRRDERSYYNDIVTPMEIEKQEENFLSMGRKDKCLVIEILCVTESRFTERFVTDAALSEYLTSLFNGAEDLLKGLDSRIQLNLIGIDARKGQAEDLSYIKQSEYNNTYINDNILYKMKDYYCKEMNE	27765.60
>TRINITY_DN14586_c1_g1::TRINITY_DN14586_c1_g1_i6::g.47222::m.47222 metalloasthenase114	>Tcham_Metallochampionase_29 MKKYYCEKMNELEKSADLIMLIVTRELIKVVDNKIVKRIVGQAMAPYVCECKKNAVSLDESDYNERVDTVAHEIAHMLGVPHDGIGTEEVNLPNGPGAKDCPPNTGFIMGNRNAANQFKFSECSKKCAKYLLSLPR	15192.64
>TRINITY_DN14567_c3_g1::TRINITY_DN14567_c3_g1_i1::g.48283::m.48283 metalloasthenase54	>Tcham_Metallochampionase_30 LEPAGDILAEDFAIYNSNNEKQHTVDVESLRRRMYRDDFNAAVLLIDDVEEIPSIEGTISYKFKITPHEWKGMTRNGRRAHQIEEIIMDESFLVRDSVIPSDFQTEIRNLTRLDKDKECIVVEYLIATESAFTEAHKTHKELTEYITSIVSGAEGMYRRSNLKTKLRLLGIQAFTEEDEPAFIKASLNKDGKYYSLQSSMKGLNKYYCENPTDLSKKADIIFLFTKRSLRDDKNKAVDAGVMLNSGACQKCNKIGVVADSSNYLQAMITLAHETAHGIGVPHDGDSTKTISLPDGPGALSCKLKDGYIMGTSSGSNSLYFSTCSKECMAYFLSLPRSSCVHEKC	38539.69
>TRINITY_DN14586_c1_g1::TRINITY_DN14586_c1_g1_i8::g.47228::m.47228 Metallojaimease109	>Tcham_Metallochampionase_31 LKLIVLLLLSRELIKVVDNKIVKRIVGKAAAPNVCECKKVAVSQDESDYNERVDTVAHEIAHMLGVPHDGIGTEEVNLPNGPGAKDCPPSTGFIMGNRNDANQFKFSECSKKCAKYLLSLPR	13330.49

Sequence identifier	Amino acid sequence[a]	MM*
**Alfa-amylase**		
>TRINITY_DN14068_c0_g3::TRINITY_DN14068_c0_g3_i4::g.14182::m.14182 Metallojaimease129	>Tcham_Metallochampionase_32 AEKTGREDVVYPSLETSRIGIKTLKFRTLDQDISLKLQSAAHIIADDFVFMDGKGNNLMTNDSIKALKSRIYKDSENGAALIIDEDGPLSIKGIVNSNLRIEPIESEEKIKDGVRAHRIIEVKTNDRPGIGFNDDIVIPPGIKDEVTKVKHSSKSES	17344.68
>TRINITY_DN14679_c0_g3::TRINITY_DN14679_c0_g3_i9::g.59163::m.59163 Metallojaimease90	>Tcham_Metallochampionase_33 RQLDSGLKFRLLGIMAFTKETEPSLFEENKHPNGAYKPDVFGKIRDYFLENSTSLSKNADMIAVLFTRSMRDRISDSYSIEGLAYGGSVCSDNKVCAINAYVKADSAAGNLAHEMAHSLGVPHDGASPQATGVSRSPGAKWCPFTDGFFMGTDTGVNKRKFSRCSKNCAKYLFSQPQASCVYEDCSSTRY	20832.44
>TRINITY_DN14659_c3_g1::TRINITY_DN14659_c3_g1_i14::g.61333::m.61333 metalloasthenase01	>Tcham_Metallochampionase_34 MLEKMFLMFCVALCESYFAGISLAGGVCKQCGKVGLAQDDADYNERVDTVAHETAHLIGAPHDGEEPFLSGSPGAKNCPESDGYIMGGNGNKANKSKFSNCTKECVEYLFSLQSASCVLVDC	12986.72
>TRINITY_DN14659_c3_g1::TRINITY_DN14659_c3_g1_i8::g.61322::m.61322 metalloasthenase01	>Tcham_Metallochampionase_35 MENVVRIAGKKKCIVIDYLCVTESTFTQRFAKTKDLVEYITILYTGVQNLLDTLNLQIKAHLIGITAFEKANEPSFINESAIPSDPLVLDPENLVRKMASHYCKITTGQPKDADIIMLITNRKLGELRGDGTIAYNTAGISLAGGVCKQCGKVGLAQDDTDYNERVDTVAHETAHLIGAPHDGEEPFLSGSPGAKNCPESDGYIMGGNGNKANKSKFSNCTKECVEYLFSLQSASCVLVDC	26129.81
>TRINITY_DN14567_c2_g1::TRINITY_DN14567_c2_g1_i17::g.48250::m.48250 metalloasthenase120	>Tcham_Metallochampionase_36 IPSGRVEVVFPSVETSRSGVKTIKFTALDKDVELKLTNAGEILEENFIIEDGNGEIKYSVDVENLRKNIYRDSANGAALLIDEDGPLAIEGIINSNLRIKPHESGRITKDGVIAHQIVELIDDKKSNVKNSIMPKDVNTETYNVTNMARKDNKCIVIDYLAMTESKFTGTFQSNKVLAQYVTVMFTGVQNLMDSLKLEIKVRLIGIKAYDKQSEPSFLKESLIPNHDVFNSSKLFENIEGYLCEQSSGLTVRADIIIIISSRRFGNMTSNGTISVTAVGLSNGGSVCDSCTKFAICMDDSKYNERVDTVAHESAHILGSPHDGEGSEYADVEGSPGSTKCPSSGGYIMGTRNNKNRFKFSQCTKQCIEHLLSKSQASCIFQKCHH	42373.00
>TRINITY_DN14679_c0_g3::TRINITY_DN14679_c0_g3_i7::g.59166::m.59166 metalloasthenase65	>Tcham_Metallochampionase_37 YFRKNPTTLSENADIIAVLLSRSIRKRMGDSFLIEGLAYGGSVCSGNKVCAINGSVNAESAAGNLAHEMAHS	7585.59
>TRINITY_DN14523_c1_g1::TRINITY_DN14523_c1_g1_i3::g.47355::m.47355 Metallojaimease66	>Tcham_Metallochampionase_38 DSGLKLRLSGIVAFTKETEPPFFKHIVLPNGDYNKKVLSEFKDYFCQNPTSLSKNSDIVLLLITRFMKYLSADGSYRDPAIGIAYAGSVCDSCYKVSVIDTSMFPDSSAMVLAHEISHLLGVPHDGESPKHTGVYGSPGAKSCSPADGFYMGNDRGVNRRKFSQCSKDCAKYLLSKSQASCVYEECKSSRY	21000.88
>TRINITY_DN13910_c0_g1::TRINITY_DN13910_c0_g1_i1::g.65500::m.65500 XP_023231741.1	>Tcham_Metallochampionase_39 MEGTAEDFERIRGNLLYSFTESCIDFHFSNMEWSDEDIILEIGCGPGRMTKTTLLPRLSKFKKMVCIDINPDYIEYARKMYFDERIEYKTQDILEWKDLEKGEKYDKIISFHVFHFIPDYEKLFSVIFSILKPGGRFLFVFPTKVPFFSVMLEMCADEEWKKFIKREDVLAGIPQSQFSNN	32403.58

Sequence identifier	Amino acid sequence[a]	MM*
**Alfa-amylase**		
juvenile hormone acid O-methyltransferase-like isoform X2 [*Centruroides sculpturatus*]	VENEFKDALSKFGLRVWNCKCEFVDLIFHDKKHILDMYYSALSKIFFKDLEAETKSLLWKKIEPTLMKHCSQKENGEIVAPHEIIQMSGTKTI	
>TRINITY_DN14423_c5_g1::TRINITY_DN14423_c5_g1_i8::g.18333::m.18333 XP_067125135.1 juvenile hormone acid O-methyltransferase-like [*Centruroides vittatus*]	>Tcham_Metallochampionase_40<br>LAFPLIRKINMEGTAEEYERLLGNMFYSYVKSSMDLHFPLMEWSEDDIILDIGCGPGRITKNIFLPKCPKFKKIVAIDIASNYIELAKNKYFHERIEYKTQDILQSLNPEEIGKYDKIVSFFAFHLITDYQRLFSIISSLLKPGGYFLFSIVTKCPLFFALRELSTDQEWNEFIKTEDVLAAVPPTINWKDPGAEFNNIVSKFGLRVSKNKCDSPNFVYSDIKHLLDFNFVALPRGVFKNLEPVVKNRLWKKAESIYPKYCTQMPNGKTSISYEVLNISGKKVM	32851.23
>TRINITY_DN13740_c0_g1::TRINITY_DN13740_c0_g1_i1::g.71239::m.71239 XP_067126753.1 angiotensin-converting enzyme-like isoform X1 [*Centruroides vittatus*]	>Tcham_Metallochampionase_41<br>MLPAFLLYLCSISCCKYLALEEHFAFHFIRHVNEVNEKQCNLFQRASWNYDTNITIPNKNKMIEQQMIASNISKQMWEVLTKFQWKKFFDPQLRRQFYHLSFLGDSALPSDKIRKKTSLEADMTNIYSTTTICDFRNKKKCNLSLEPDLSSIMANSNDYYELLHVWKEWRNKVGRKIRPLYWEFVDLKNEAARLNGFNDAGEFQREKYESPTLIQDLENLWYQIRPLYQQLHAYVRRKLIETYGSDKIVDDGPIPAHLLGNMWSQEWQNIINITIPYRNKPSLDVTPQMKAKGMKPVQIAKLAEQFFVSLGLQPMTKEFWSNSLFEKPKDRKVVCHASAWDLCDKRDFRIKMCMECTMDFLITTHHEMGHVQYYMQYANQPHVFRRGANPGFHEAIGDVMSLSVYTPNHLQSIGLLNDIVSDREADINFLLQMALKKIAFLPFGYTLDKWRWEVYSGEVEFEEWNNRWWEMRFKYQGVCPPIRRTEIDFDPGAKFHVSNDTPYIRYFISFIIQFQFHEALCRVSGFQGPLHKCDIYNSKEAGKLISGMMQMGSSRPWPEAFEKITGSQKMNADSLLAYFKPLYEWLKEANKNEVIGWETFDPTVCP	71552.24
>TRINITY_DN14507_c0_g1::TRINITY_DN14507_c0_g1_i5::g.48408::m.48408 XP_067121543.1 endothelin-converting enzyme homolog [*Centruroides vittatus*]	>Tcham_Metallochampionase_42<br>MSVNPMSMKGMDEKEQEFRINCQSDHEGNSTNPRERLLIILAFIFLVLVLSLLCAIVISIAVESLLPKSGLCLTKECVKKAGDILENLNEEVDPCDDFYSYTCGGWKNKNLLSLPGKSIFQEIQMKIYDQMYSFINKIENDDGIEGKVAKITKSCIDHSKSEEKKNLKDSYDKLFQSFGGFPLANVLESSAMTITEKIANLYRLLGTEPVLRIAVMPSDKNNTKNVLIIAPPDPSIFNSYDDDIKLAKKILNDVYNITDPDSDELTKIKTFYEEIKNQIPDDSQYKEEYLSSNENEKITDFFGTFEFEQFIKTLLIEMPKESITKFKKLIPSKAATPSTDIETTSESPKTEQLSDTTSTIDQPSTTAISQNKQLKQSLKRRKRENEVDRTPIIEILLVNPDIIKKVLEVFMNKTQKGEKTTIQNFLSIQFLKNLEENIVMGTSEERLSKKTMSKEVEDGTIMKCLEDISEYLMYAFDYIYIKNTNVPIDKAKLVVEHIKKAMKSFVPAYSWLDKADEILVQEKLDKMESFVGFPAWLSDKAQVQKYYEDLKLNTATAKVQKNKLVRRGSLDGDDGDGGESDYGDRNVGGNDGLGGADRSGSGPVEGGGSGNGPGESGGSVPGESGGSVPAESGGNGPAESGGSGSGVGGGSNNGKGVETIKPYGNGNEGGNGESNNEKGVETIKPYIEIYMEMVKFNKKKLLEKLFKTNDRRSWPTIPALSITTVNAYYSSQLNAIVVPAAILNPPIFDVDIPFYLNFGSLGTVIGHEITHGFDTNGRKRDKNGNIPKDSLWKEDTINSYNLQAECFQIQYSNYTVDETRVNGNKTLAENIADNGAINEALTGYRYWLSEHHKGKKENSLPALGNFTHEQLFFISYAQSWCHSATRKYLIQLIESDEHTPNQFRVKGSLSNSKEFSEAFECDNKKSMNPQNKCKLW	104408.88
>TRINITY_DN13680_c0_g1::TRINITY_DN13680_c0_g1_i2::g.28198::m.281	>Tcham_Metallochampionase_43<br>MPAAVSQSQLTLTSTESGKFKNPPWWNRRSRLERTLCGMSVVALLMFVAMAIALAVIGYHYQKTIDSRSDDIPSTADKLIEESGKGSENDYSDTSNVCLTQGCVKAAALLINNMDQTADPCQDFYKFSCGGWVEKQIIPEDKTSLSVFSLLQDELNQKLRVMIEKPLNDSAPDF	8878

Sequence identifier	Amino acid sequence[a]	MM*
**Alfa-amylase**		
98 XP_067139630.1 neprilysin-2-like [*Centruroides vittatus*]	IVRLKRMYESCMNLTVIEGKDAEPLKNVLKEIGGWPVIEGPSWDNSKFDWVDTLIQFRKYGYSHDILLDLSVTPDYRNNTNHIVDLDQTSLGMPDRSYLLKGLNDTTVSAYYRLMVDSAVFLGAEREKAEEALLKSLKFEITLANFSLPREERRNISKLYNKMTIKDLVKLAPNINWMKYFNGLLNNPITEDEQLIVNVPEFIRKFGILIDEIDKTTLANYMLWRLVGQSMSMLPKKWRDRAQKYSSVITGKTREEPRWEQCMGSLTGSLGVSLASLYVRNHFKQDSKKEALEMVNYVHKEFFKILEEIPWMDEVTRQKAKDKATTIRSYIGYPDELLNNTKISSLYENLTLTKDNYFMNIQKVRKWSTDYAFNQLRKPNEKGDWKKHARAAVVNAYYNALENSIEFPAGILQGTFFDKERPAYMNYGAIGFVIGHEITHGFDDRGRQFDKDGNNINWWDYETDGKFRKLAQCIIHQYGNYTVPENGLKINGVNTQGENIADNGGIKEAFRAYHQWVKNHGTEKLLPGLKFTQEQLFWISAANVWCGKYRPEVLKLRILSGSHSPAQFRVIGVASNSPEFSRVFNCPKGSPMNPEKKCSIW	8.36
>TRINITY_DN14275_c2_g1::TRINITY_DN14275_c2_g1_i15::g.62238::m.62238 XP_067138462.1 nicotinamide N-methyltransferase-like [*Centruroides vittatus*]	>Tcham_Metallochampionase_44 MEAVCEDKYAFSKFYEDVNFTLNYRDTITNNMKNEEEFVSYCLHKIFQDKAFQGKRLIDIGSGPTVHKLAAASAYFSEIILSDYSQHNRKEIQKWLKNESSAINWDSLFQQEAILEGYRDVEEGINEIKLRLRQKIKNVIHCDVLQKEILPINIGKFDILFTSYCLEAACISLESFDSVIKNLSNYILPNGGLVMATVLGCSQYQMKGYDKTFPV	24759.30
>TRINITY_DN12152_c0_g1::TRINITY_DN12152_c0_g1_i1::g.34292::m.34292 XP_067144456.1 adenosylhomocysteinase [*Centruroides vittatus*]	>Tcham_Metallochampionase_45 MSSKSPFKVADINLADFGRKEIIMAENEMPGLMAIRRKYGPSKPLKGARIAGCLHMTIQTAVLMETLIELGAEIQWSSCNIFSTQDHAAAAMAKRGIAVYAWKGETDDEYIWCIEQTLVFPNGQPLNMILDDGGDLTNLVHTKYPQYLSGIKGISEETTTGVHNLYRMLKEGRLKVPAINVNDSVTKSKFDNLYGCRESLTDGIKRATDIMLAGKVCVVAGYGDVGKGSAQSLRAFGARVIVTEIDPINALQAAMEGYEVTTMEEACTKASVFVTTTGCRDIIRSEHFMMMRNDSIVCNIGHFDIEIDVKWLNENSVEKVNIKPQVDRYKLSNGNHIILLAEGRLVNLGCAMGHPSFVMSNSFSNQVLAQIELWTKPMQYTIGVYFLPKTLDEEVAALHLEHLNVKLTKLTENQAHYLNLEQHGPFKPEHYRY	48261.56
>TRINITY_DN14427_c1_g1::TRINITY_DN14427_c1_g1_i1::g.14927::m.14927 XP_023231741.1 juvenile hormone acid O-methyltransferase-like isoform X2 [*Centruroides sculpturatus*]	>Tcham_Metallochampionase_46 MEGTAEDFERIRGNLLYSFTESCIDFHFSNMEWSDEDIILEIGCGPGRMTKTTLLPRLSKFKKMVCIDINPDYIEYARKMYFDERIEYKTQDILEWKDLEKGEKYDKIISFHVFHFIPDYEKLFSVISSILKPGGRFLFVFPTKVPFFSVMLEMCADEEWKKFIKREDVLAGIPQSQFSNNVENEFKDALSKFGLRVWNCKCEFVDLIFHDKKHILDMYYSALSKIFFKDLEAETKSLLWKKIEPTLMKHCSQKENGEIVAPHEIIQMSGTKTI	32343.48
>TRINITY_DN14567_c2_g1::TRINITY_DN14567_c2_g1_i1::g.48269::m.48269 metalloasthenase03	>Tcham_Metallochampionase_47 IPSGRVEVVFPSVETSRSGVKTIKFTALDKDVELKLTNAGEILEENFIIEDGNGEIKYSVDVENLRKNIYRDSANGAALLIDEDGPATIEGIINSNLRIKPHQSGRIIKDGVIAHQIVELIDDKKSYKHDYVMPEDVKTETENVARMAREGECIVVEYYVVTDSRFTQRFDSDQAVTNYVTVTFTEVQNKIDTMNLGIKVRLLGVTPYHKESEPSFIQDSLLSGHQEYVDARKIVHNMKVYFCKHKTGLAKTADIIMLLITRTLGVVEENSTTVSEIAGSSSISSVCQRCNNVGICIDNSDYNERADTVAHESVHLLGSPHDGEGPDDGLDVPGSPGAANCPDSDGYIMGTRNNKNGKKFSECTKQCVKYLLSLPRASCVYENCSK	42636.99

Sequence identifier	Amino acid sequence[a]	MM*
**Alfa-amylase**		
>TRINITY_DN14567_c2_g1::TRINITY_DN14567_c2_g1_i5::g.48257::m.48257 metalloasthenase03	>Tcham_Metallochampionase_48<br>IPSGRVELVFPSVETSRSGVKTIKFTFLDQDVELKLISAGDILGKDFTIEDVNGQIKHSVDVENLRRKMYRDSANGAALLIDEDGPATIEGIINSNLRIKPHQSGRIIKDGVIAHQIVELIDDKKSYKHDYVMPEDVKTETENVARMAREGECIVVEYYVVTDSRFTQRFDSDQAVTNYVTVTFTEVQNKIDTMNLGIKVRLLGVTPYHKESEPSFIQDSLLSGHQEYVDARKIVHNMKVYFCKHKTGLAKTADIIMLLITRTLGVVEENSTTVSEIAGSSSISSVCQRCNNVGICIDNSDYNERADTVAHESVHLLGSPHDGEGPDDGLDVPGSPGAANCPDSDGYIMGTRNNKNGKKFSECTKQCVKYLLSLPRASCVYENCPESV	42886.37
>TRINITY_DN14567_c2_g1::TRINITY_DN14567_c2_g1_i16::g.48259::m.48259 metalloasthenase25	>Tcham_Metallochampionase_49<br>IPSGRVELVFPSVETSRSGVKTIKFTALDQDVELKLTAAGEIIGRDFKFQDASGEMIDSFDVDNLRRKMYRDSANGAALCIDEDGPLTIEGIINSNLRIKPYESGRIIKDGITAHQIVELVDDKISYFKDSISVIPKDIGREAEKNIAGMARDGECIVVEYLILTESTFTQRFESDEELNKYVALTFTGVQNLMDTLEMGIKVVMIGHLSYTTETEPSFITDNSFPSKKEVLNPEKVIDSMTDYFCNNKTELSERADIIILISARKLGRVKPNGEVAADLAGLSNDGSVCKPCAKMGVCNDNSKYNERADTIAHESAHMLGSPHDGDGPEKFGVKGSPGSANCPETDGYIMGNRNKENGMKFSECTKECIKYLLSQPRASCVYENCPESV	42912.51
>TRINITY_DN14567_c2_g1::TRINITY_DN14567_c2_g1_i14::g.48267::m.48267 metalloasthenase25	>Tcham_Metallochampionase_50<br>IPSGRVELVFPSVETSRSGVKTIKFTALDQDVELKLTAAGEIIGRDFKFQDASGEMIDSFDVDNLRRKMYRDSANGAALCIDEDGPLTIEGIINSNLRIKPYESGIIIKDGITAHQIVELVDDKISYFKDSISVIPKDIGREAEKNIAGMARDGECIVVEYLILTESTFTQRFESDEELNKYVALTFTGVQNLMDTLEMGIKVVMIGHLSYTTETEPSFITDNSFPSKKEVLNPEKVIDSMTDYFCNNKTELSERADIIILISARKLGRVKPNGEVAADLAGLSNDGSVCKPCAKMGVCNDNSKYNERADTIAHESAHMLGSPHDGDGPEKFGVKGSPGSANCPETDGYIMGNRNKENGMKFSECTKECIKYLLSQPRASCVYENCPESV	42869.48
>TRINITY_DN14319_c2_g3::TRINITY_DN14319_c2_g3_i1::g.6831::m.6831 metalloasthenase129	>Tcham_Metallochampionase_51<br>TGRENVVFPSVETSRSGVKTIKFRALGEDIELKLEPAGAILSKDFALYNGNKEKDHSVDVESLRRKIYRDSANGAALIIDDDDEKSQSIRGVVNSKLRIEPHELRGTTQYGRNAHCIIELTSDGNSHYSDSIILPKSEIEARNLTRMSRADKCIVVEFLCLTESKFTERFKTNKELTDYVTTMYTGVQNMYDTMNLGIVIRLSGIQAFTKENEPSYIKDSEVLKGQYLDGKVLANMRSYYCKNSVGLAKDADIIMLIVRRVLVGLTGSKITSKALGIAYGAGLCHKCDKVGVTLDESDYNERIITLAHESAHLLGVPHDGMGTEEVDLKDGPGAKSCPYDDGYIMGTAEEDNMLKFSKCSAACAKYFLSLPQASCAIEDCKSSH	42405.17
>TRINITY_DN14319_c2_g3::TRINITY_DN14319_c2_g3_i4::g.6839::m.6839 metalloasthenase129	>Tcham_Metallochampionase_52<br>TGRENVVFPSVETSRSGVKTIKFRALGEDIELKLEPAGAILSKDFALYNGNKEEDHSVDVESLRRRIYRDSVKGAALVIDDDHEQSPSIRGVVNSKLRIEPHELGGTTKYRRNAHRIIELTSEGNSYFSDNVIPPKTESEARNLTRMSRADKCIVVEFLCLTESKFTERFKTNKELTDYVTTMYTGVQNMYDTMNLGIVIRLSGIQAFTKENEPSYIKDSEVLKGQYLDGKVLANMRSYYCKNSVGLAKDADIIMLIVRRVLVGLTGSKITSKALGIAYGAGLCHKCDKVGVTLDESDYNERIITLAHESAHLLGVPHDGMGTEEVDLKDGPGAKSCPYDDGYIMGTAEEDNMLKFSKCSAACAKYFLSLPQASCAIEDCKSSH	42515.27
>TRINITY_DN14368_c0_g1::TRINITY_DN14368_c0_g1_i5::g.7133::m.7133 Metallojaimease127	>Tcham_Metallochampionase_53<br>GPPGTRRISTLLVGILLLRNPIMLGLAFLINVSVLIGVSAIRSGREEIVFPSMETFRSGEKRLSFRALNQDIELKLEPAGYLFGEGFFVESVHDGKSEIPELNIEDIKRSLYKDKRTGASLHIEDKERILINGIINDNLRIEPINSEKERRDGAIPHRIYEIISDDNFRINDAVFPTDYHPVYNGRLKQLKAEEPIYLEIGIITDSAFSLSFPDTESLLSYLSVLVNGAQCRYDNIKSIKVTLVLASVKINKSTKEEPYIQNNIREEYVMIYDALNSFSVA	57436.64

Sequence identifier	Amino acid sequence[a]	MM*
**Alfa-amylase**		
	MGKAAYPSDIIMLVSKREMGMIKNGTIDTSINGIAYVGGACAKQSARGTIRVGISHDKGHSFGSAKLFAHECAHLLGCPHDGEGPDPYLPGHPGSSKCPWSDGYMMSYVDNTKNRNYFSSCCEENIRYLANRVDCLRKNNTGYNIKSTNQKPGEYMESVLGLSASNYAKDKVPYYNDRCIKLYQPNATSFRYPDGKCEFVCRSTIKKSFRTIKCLDGEMCDVGKRCSNGMCQ	
>TRINITY_DN14444_c2_g3::TRINITY_DN14444_c2_g3_i4::g.18304::m.18304 metalloasthenase05	>Tcham_Metallochampionase_54<br>SNFTERFETEQALTEYVTLMYSATETLLRQLDSRLQLRLSGIQAYTKESEPPFIKDNELEEGKYYNARGVLRAVRDHLCDEYNYYFHNDGSTPTKRADIVMLLITRSMMFLKPDGSSDGLPAGKCFRVLLCFFVNSFSELVFNF	166697.94
**Other Proteins Families**		
>TRINITY_DN13397_c1_g1::TRINITY_DN13397_c1_g1_i1::g.50918::m.50918 Tja_SVWC_01	>Tcham_SVWC_01<br>YTARTTVPNVNGKCDIKGNLINSGDTYYDEERCEAWLCSASTNPISYNVSKDGKFEPIYDKKAYVEILGCGVAKVEKDGKLCSVVKTTGKYPECCNGPTVCP	111127.60
>TRINITY_DN14686_c1_g1::TRINITY_DN14686_c1_g1_i32::g.59393::m.59393 XP_067141325.1 hemocytin-like [*Centruroides vittatus*]	>Tcham_01<br>DPLLLHRQPYRLEDPRLLHRLPYPIVSITGSSTFASPTVSTTVSSSSASPTVSTTFTPTSTTFSPTQSTGFSYSASPIVSTTLTPTLTTFKSPTLSTESLTVPESHRLSYRLQDPRHLHLNISKIECKTNEWLSDEIILDINCSKRYICLPVPFEEIKISVKGQDNDLQYWEIHLKHFRDIAFIEIVDSKDLVLVLVLVYSLDGSESNMTNQLLYFNEFEKVKFKETIRVKSFAFVPLAINESDISQVIMKISVCKDKECENLNETYCPENCEKNNLECNGTACVTSGSCPCVVNSIVYPPGIYIDECRNRTCENSKFTETYISCEDRGKNICKEKQTLFEDRDTCDCDCRCNNISVNCNTSELCIEREKICDGIKDCINGTDEKCCPVNVTELSNCKRTNGECGRSNATCKFRENTVIMFSKVVYTCLEVCEAILLQERGKKTFRISYHRVYQNEERFTHHVIIVVDNYSYNLPQKELVIVIKDDVETVVSLSASSSYYNEQGGVSVQVIGDAIQFRSEQKGFTVIWRERNIVDIELDACEIDRVEGLCGYFATRAIHDEYRGKDGTCFNKDAEMCSDWTFDKSNDVCGSIYEMENEKLLTAERVCDEISKKVYYTCNVDVTEQTSDNSFRANDKVCKYVIYPNCTTDNRLCQCEALKYLLVEASCKNGIADLLLEYNCRELCPEGFQWDMCDADNTCEDLTFNGNSKKKIAGCFCPKHLVRKGDKCIPSHLCENCVCDIYGDPNYQTFNKRNYRFLSTCPGPYVLVQRKLPSNEPFFKVLSVNEKCIEYVNGTCISGIHVYYNNTKIEIYRNTTVLKNDRLYTSYCEDTSCNFNGIDFKIETETNIYIVKLVEIHVVIEYKIGNATLHIAGEVSIKFSSYTFFNNTEGLCGSCNGDSSDEFRLRNGSVVDDISTFALDWLEEKNKSLCEIYKTDNCTNIQNVSKCEFPQHLNCSIVDNNRYYSRHSGSCKNDLRCNDQITDSDICKYKFLYADNCCAFRLPMDKWLEIENCVIPCKENRTFRCVDICETKCPAERNEQCEFGKQMRMVCACPDGYVERNDKCIKNTECDACSPKPTLNIPARYFNIDNCTNLDPIVNMYECNGKCNDDFSHYTIERPYNFKEPIDYIYKKKCHCCQPSKYINDIITLTCINGLKKNIIIKKPIDCTCIECNATVI	134283.31
>TRINITY_DN12815_c0_g3::TRINITY_DN12815_c0_g3_i3::g.70355::m.70355 Tja_PHM01	>Tcham_PHM01<br>MNETYFCTSFKMNKHEHEYVVKFEPNATMHVAHHILIYGCGEPGLVQRDTPRIVWNCGEMMGSNSEYASAPICAEGSQVIYAWARDAPALNLPEGVGFKVGKGSGINYLVLQVHYADVTRFLNGGTDHSGIILTLLPSSDKSVTKRAGVYLLGTSGFIRAKQLEHMETACKIDKRLVIHPFAFRTHTHALGKAVSGYVIDRDSGKWKLIGKHDPLEPQMFYPVKDKRLVIQEGDVLAARCTMYNFRNRITLIGSTAEDEMCNFYMMYYVEGDDILDDKYCFSMGPPAYYWDNDELVGDVPKWVDEDASSLRE	35175.12
>TRINITY_DN14556_c3_g1::TRINITY_DN14556_c3_g1_i1::g.47472::m.474	>Tcham_02	2468

Sequence identifier	Amino acid sequence[a]	MM*
**Alfa-amylase**		
72 XP_023220762.1 ras-related protein Rab-3 [*Centruroides sculpturatus*]	MAAGQDQKWQKDAADQNFDYMFKILIIGNSSVGKTSFLFRYADDSFTSAFVSTVGIDFKVKTVFRQDKRVKLQIWDTAGQERYRTI TTAYYRGAMGFILMYDVTNEESFNSVQDWVTQIKTYSWDNAQVILVGNKCDMEDERVVSTERGKQLADQLSLEFFETSAKENINVK AVFERLVDIICDKMSESLDSDPTLVSASKGTRLTENPAPPNGGCQC	3.76
>TRINITY_DN11898_c0_g1::TRINITY_DN11898_c0_g1_i1::g.25234::m.25234 XP_023244316.1 ras-related protein Rab-2 [*Centruroides sculpturatus*]	>Tcham_03 MSYAYLFKYIIIGDTGVGKSCLLLQFTDKRFQPVHDLTIGVEFGARMITIDGKQIKLQIWDTAGQEAFRSITRSYYRGAAGALLVYDITR RDTFNHLTTWLEDARQHSNSNMVIMLIGNKSDLESRREVKKDEGEAFAHEHGLVFMETSAKTAANVEEAFINTAKEIYEKIQEGVFD INNEANGIKIGPQHSPTNPSMPAGGQGSNQSGGCC	23545.56
>TRINITY_DN13752_c4_g1::TRINITY_DN13752_c4_g1_i2::g.70978::m.70978 XP_067127455.1 ras-related protein Rab-11A [*Centruroides vittatus*]	>Tcham_04 MGTKDDEYDYLFKVVLIGDSGVGKSNLLSRFTRNEFNLESKSTIGVEFATRSIQVDGKTIKAQIWDTAGQERYRAITSAYYRGAVGALL VYDIAKHLTYENVERWLKELRDHADQNIVIMLVGNKSDLRHLRAVPTDEARTFAEKNNLSFIETSALDSTNVEAAFQQILTEIYRIVSQ KQIRNDTDNDPSPTGDNIKPITIPPTDSSDLRKKQCCQS	24512.52
>TRINITY_DN7146_c0_g1::TRINITY_DN7146_c0_g1_i1::g.52109::m.52109 XP_067132834.1 rab GDP dissociation inhibitor beta-like [*Centruroides vittatus*]	>Tcham_05 MDEEYDAIVLGTGLKECILSGLLSVSGKKVLHIDRNKYYGGESASITPLEELFAKFNLPSAQLDEYGRSRDWNIDLIPKFLMANGQLVKL LIHTGVTRYLEFKSVEGSYVYKGGKIFKVPADEKEALVSNLMGMFEKRRFRNFLIFVQEFSVEDPKTWKDIDPNKTNTAQMYEKFGLD KDTADFTGHALALYRDDEYLNRPCVETISKIKLYSDSLARYGKSPYLYPLYGLGELPQGFARLSAIYGGTYMLDKPIDEIVLEDGKVVGVR SGKEIAKCKQVYCDPSYVPDRSEKVGQVVRCICLMNHPIPNTKDALSCQIIIPQKQVGRKSDIYVSLVSYTHQVAAKGWFVAMVSTTV ETNNPENEIKPGLDVLGPIRQKFIWVSDVYRPKDDGRESQIFISESYDATTHFETTCLDVLDIFHRGTGEDFDFSKVKHNLGDEQD	50166.23
>TRINITY_DN10978_c0_g3::TRINITY_DN10978_c0_g3_i1::g.35926::m.35926 XP_023215707.1 ras-related protein Ral-A-like [*Centruroides sculpturatus*]	>Tcham_06 MSKVKNAQPALHKVIMVGSGGVGKSALTLQFMYDEFVEDYEPTKADSYRKKVMLDGEEVQIDILDTAGQEDYAAIRDNYFRSGEGF LCVFSITEHESFSATMDFREQILRVKGDDNIPFLLVGNKADLDDRRQVSLEEAQNRAKQWNVPYVETSAKTRANVDKVFFDLMREIR NRKQTESKASNGPRKPGKGKIKCVIL	22580.64
>TRINITY_DN10545_c0_g1::TRINITY_DN10545_c0_g1_i1::g.53795::m.537	>Tcham_07	2076

Sequence identifier	Amino acid sequence[a]	MM*
**Alfa-amylase**		
95 XP_023212825.1 ras-related protein Rap-1A [*Centruroides sculpturatus*]	MREYKIVVLGSGGVGKSALTVQFVQGIFVEKYDPTIEDSYRKQVEVDGQQCMLEILDTAGTEQFTAMRDLYMKNGQGFVLVYSITA<br>QSTFNDLQDLREQILRVKDTDDVPMILVGNKCDLEDERVVGKDQGANLARSFNNCAFLESSAKAKINVSEIFYDLVRQINRKNPEKKI<br>KPPPKKCTIL	5.84
>TRINITY_DN11197_c0_g1::TRINITY_DN11197_c0_g1_i2::g.28901::m.28901 XP_067144915.1 ADP ribosylation factor 4 [*Centruroides vittatus*]	>Tcham_08<br>MGLTLSSILTRLFGKKQVRILMVGLDAAGKTTILYKLKLGEIVTTIPTIGFNVETVEYKNICFTVWDVGGQDKIRPLWRHYFQNTQGLIF<br>VVDSNDRERISEAQDELQKMLNEDELRDAVLLLFANKQDLPNAMNAAELTEKLGLNQLRNRRWYIQATCATQGEGLYEGLDWLSN<br>ELSKAK	20625.75
>TRINITY_DN14027_c1_g2::TRINITY_DN14027_c1_g2_i1::g.13919::m.13919 XP_023244829.1 ras-related protein Rab-1A [*Centruroides sculpturatus*]	>Tcham_09<br>MSTMNPEYDYLFKLLLIGDSGVGKSCLLLRFADDTYTESYISTIGVDFKIRTIELDGKTIKLQIWDTAGQERFRTITSSYYRGAHGIIVVYD<br>VTDQESFNNVRQWLQEIDRYACENVNKLLVGNKCDLSAKKVVDYTTAKEFADQLGIPFLETSAKNATNVEQAFMTMAAEIKNRMG<br>PPTSGGDAVQGGGKINPGIPIKPQGSGCC	22762.88
>TRINITY_DN13120_c0_g1::TRINITY_DN13120_c0_g1_i1::g.49446::m.49446 XP_067126872.1 ras-related protein Rab-7a [*Centruroides vittatus*]	>Tcham_10<br>MSSRKKVLLKVIILGDSGVGKTSLMNQYVNKKFSNQYKATIGADFLTKEVTVDDRLVTMQIWDTAGQERFQSLGVAFYRGADCCVL<br>VFDVSTPNTFKSLDSWRDEFLIQASPRDPENFPFVVIGNKIDLENRAISTKRAQGWCQSKNNIPYFETSAKEAINVEQAFQTVAKNAL<br>AQETEVELYNDFPDQIKLSNEQKSKAEGCSC	23061.11
>TRINITY_DN12570_c0_g1::TRINITY_DN12570_c0_g1_i3::g.8035::m.8035 XP_023215218.1 actin, clone 403 [*Centruroides sculpturatus*]	>Tcham_11<br>MCDDDVAALVVDNGSGMCKAGFAGDDAPRAVFPSIVGRPRHQGVMVGMGQKDSYVGDEAQSKRGILTLKYPIEHGIVTNWDD<br>MEKIWHHTFYNELRVAPEEHPVLLTEAPLNPKANREKMTQIMFETFNTPAMYVAIQAVLSLYASGRTTGIVLDSGDGVSHTVPIYEG<br>YALPHAILRLDLAGRDLTDYLMKILTERGYSFTTTAEREIVRDIKEKLCYVALDFEQEMATAASSSSLEKSYELPDGQVITIGNERFRCPEA<br>LFQPSFLGMESCGIHETTYNSIMKCDVDIRKDLYANTVLSGGTTMYPGIADRMQKEITALAPSTMKIKIIAPPERKYSVWIGGSILASLS<br>TFQQMWISKQEYDESGPSIVHRKCF	41809.79
>TRINITY_DN14395_c2_g1::TRINITY_DN14395_c2_g1_i3::g.4837::m.4837 XP_023217176.1 vasotocin-neurophysin	>Tcham_12<br>CYITNCPNGGKKRNVNIINHNSCGLAEKNKCSVRGLCCGPTIACLINAEGIPICQQLSHYHLCKKHGRICSLNGICNGEGICCVKDVRIE<br>GNFCAMKTENTFMKNNLRKKIISKLRN	12913.24

Sequence identifier	Amino acid sequence[a]	MM*
**Alfa-amylase**		
VT-like [*Centruroides sculpturatus*]		
>TRINITY_DN14306_c9_g2::TRINITY_DN14306_c9_g2_i3::g.5577::m.5577 XP_067139466.1 sorting nexin-2-like isoform X2 [*Centruroides vittatus*]	>Tcham_13<br>DRWLALSTGGVSKMADDRVPPPLVEDDDSVVKDDTEDLFISAVDTQLNISNGGDKKAVPPNTIEDLMAGGGVEVSLDDVPLPTPQSKLEFCTPEEEFSKDISTMDKSKEASVENDNSTAAEITLSQPVKPTVAKKIVKPGGEELEEDQFLEIGVDNPHKIGDGMGAYVVYKVTTKTNLPYFKKKLFSVSRRFSDFLGLHDKLVERHLHLGRIIPPAPEKSVIGMTKIKMSKDETVSSTDFIEKRRAALERYLNRTAAHPVLRMDPDFRDFLETEAELPKATNTSALSGAGVIRLFNRVGDTVNKMTFKMDESDPWFEEKQHQIENLDQQLRKLHSSVEALVHHRKELSSSTASFAKSAAMMGNCEEHTALSRALSQLAEVEEKIEQLQCQQANHDFYLLSELLKDYISLIAAVKDVFHQRVKVYQTWQHAVQILTKKREQKAKLELAGKTDKIPQAREEVIEWEAKVERGQEEFENISKMIRKEVERFEQTRVQDFKATIIKYMEQLLETQQQLIKYWEGFIPEAKAIA	59905.04
>TRINITY_DN7296_c0_g1::TRINITY_DN7296_c0_g1_i1::g.48731::m.48731 XP_023224526.1 protein/nucleic acid deglycase DJ-1-like [*Centruroides sculpturatus*]	>Tcham_14<br>LRKVRGNVVLQWIRQGCVVGYVSLRKMSKSALLILADGAEEMEAVISVDVMRRGGIKVTIAGLDGSEAVKCSRDVAVVPDKSLNDALKDSPYDVIVLPGGLKGSEKMAESNQVGSLLKEQQDAGRYIAAICAAPIALKSHGITVGKLLTSHPSKKEEMKEGDKYKYSEDRVVVDGQLITSRGPGTAFEFALTIVEKLMGKEKANSLITPMLVRV	23026.87
>TRINITY_DN43758_c0_g1::TRINITY_DN43758_c0_g1_i1::g.33831::m.33831 XP_023231107.1 elongation factor 1-alpha [*Centruroides sculpturatus*]	>Tcham_15<br>MGKEKTHINIVVIGHVDSGKSTTTGHLIYKCGGIDKRTIEKFEKEAQEMGKGSFKYAWVLDKLKAERERGITIDITLWKFETQKYYITVIDAPGHRDFIKNMITGTSQADCAVLIVAAGTGEFEAGISKNGQTREHALLAYTLGVKQMIVGVNKMDTTEPPFSESRFEEIKKEVSAYIKKIGYNPATVPFVPISGWNGDNMLEPSTNMPWYKGWTLERKNAKLDGKTLLQALDAMEPPTRPLDKPLRLPLQDVYKIGGIGTVPVGRVETGVLKPGMVVTFAPVNLTTEVKSVEMHHEALSEAVPGDNVGFNVKNVSVKELRRGYVCGDSKDTPPKCTEEFTAQVIVLNHPGQISNGYTPVLDCHTAHIACKFREIKEKCDRRSGKKLEDNPKFIKSGDAAIIELAPTKPMCVETFTEFPPLGRFAVRDMRQTVAVGVIKAVKTKDVSGGKVTKAAEKAQKKK	50980.97
>TRINITY_DN13840_c7_g6::TRINITY_DN13840_c7_g6_i1::g.11359::m.11359 XP_067131452.1 dihydropteridine reductase *[Centruroides vittatus]*	>Tcham_16<br>YDFKMAAPVGRVLIYGGKGALGSSCVSYFKSKDWWVGSIDLAANEEANECVVVKPSNDWIDQDNDVTSGVEKMLNGEKVDAVICVAGGWKGGNAASKDLVAASDLMWKQSVWTSVISAELAAKYLKNGGLLTLTGAKAALEGTPGMIGYGLAKAAVHQLVDSLASSKSGLPAKSHALAILPITLDTPMNRKFMPDADHSKWTPLPFVAELLFKWANGKERPTNGSLVQLVTNDGNTDLIIA	25539.27
>TRINITY_DN144_c0_g1::TRINITY_DN144_c0_g1_	>Tcham_17	2175

Sequence identifier	Amino acid sequence[a]	M<br>M*
**Alfa-amylase**		
i1::g.23628::m.23628<br>XP_023233908.1<br>peptidyl-prolyl cis-trans<br>isomerase [*Centruroides*<br>*sculpturatus*]	VHVLALRSFPAACRIIVRQSLSSEVRSLVRRISNEMALPRCYFDMVADGQPIGRITMELRSDVVPKTAENFRALCTGEKGFGYKNSTFH<br>RVIPNFMCQGGDFTRHNGTGGKSIFGEKFEDENFALKHTGPGVLSMANAGPNTNGSQFFLTTVKTSWLDGKHVVFGQVVEGMD<br>VVKKIEGYGSQSGKTSKKIVIGDSGQL	1.9<br>0
>TRINITY_DN13803_c0_<br>g2::TRINITY_DN13803_c<br>0_g2_i1::g.9928::m.9928<br>XP_023212112.1 FK506-<br>binding protein 2-like<br>[*Centruroides*<br>*sculpturatus*]	>Tcham_18<br>EDAELEIDVLHKPTECDKSTKRGDMLSMHYKGTLDDGKVFDSSYGRSEPFKFQLGVGQVIKGWDEGLLNMCIGEKRRLTIPPHMGY<br>GDSGAGDTIPPKATLYFEVECIGIEDGPPSLNIFKEIDFDKDNQLSREEVSDYIKKQIPEAEAAGIKDLPDQDKLVEDIFQHEDKDKNGFI<br>SHEEFSGPKHDEL	21<br>37<br>7.8<br>3
>TRINITY_DN14025_c0_<br>g1::TRINITY_DN14025_c<br>0_g1_i1::g.13804::m.138<br>04 XP_023234347.1<br>peptidyl-glycine alpha-<br>amidating<br>monooxygenase A-like<br>[*Centruroides*<br>*sculpturatus*]	>Tcham_19<br>FYPEPAEYFYPEQFLDYQTNDKPGGNENKKMSIEEEIEWPKENVFKDVLQVAGVDVDKSGNVHVFHRGDRMWDSRSFKGDVYQL<br>EELGPIKTDAVLILDPFSGELITGWGKNRFYLPHGITLDNEENSWLTDVALHQVFKFAANKEEPSIVLGKKFQPGSDRRHFCKPTDVAV<br>ASSGVVYVSDGYCNSRIIAFQPDGTFFGEFAQSDKMVIPHGLSLIEERKLLCVADRENARILCYSLKEDDSLGQLIINNDVPTGAPYAIV<br>HRGEYMFAVTLSYAGRGTAMGITTKLNDGNVINTWHSYEGFSMPHDMTLSSNGTFLYVADVGKFTPKKLFKFKITDEA	38<br>41<br>8.3<br>2
>TRINITY_DN8767_c0_g<br>1::TRINITY_DN8767_c0_<br>g1_i2::g.30479::m.30479<br>XP_023234878.1<br>lysosome-associated<br>membrane glycoprotein<br>1-like [*Centruroides*<br>*sculpturatus*]	>Tcham_20<br>EDNSSDVSENTTIVISSTPIFSNTTTEIITTLSTNATTLIPNSTTESTTVLPSTTTVLPSTTTVLPPSVSDPSKGSWTMYIDGIACVKLDAAIQ<br>FRINYQQNSGNNVTAKLNVPKNATVDNTTVTTCDTLLLKFDDNAFEIKFQNVSNEISATNFTLHYLVDPKDFPDSIDTGKTLKASATNL<br>TEFKVSSGKSYLCNIETKIDMENVVLSMQEVKVEAFRDSKDDKFSEAIECSGDEKINDIVPIAVGCALAVLVIIVLIAYLVGRQRSRQQG<br>YQSV	30<br>07<br>7.8<br>1
>TRINITY_DN51526_c0_<br>g1::TRINITY_DN51526_c<br>0_g1_i1::g.6492::m.6492<br>XP_023219527.1<br>lysosomal acid	>Tcham_21<br>TDDGIPIDTTSLQLLHIIVRHGARTPISLYPNDPNENFEYVEGNGQLTNTGKLQQYTLGKYFRERYRNYITSNPREVVARSSEKDRTIISG<br>LCHLAGLYAPKEEWQFTEELHWQPIRILTSPPKTDGMLYEGAICPEAKAEQHRIRNSPEGEAYRNQYTELFNYVTNHSGMTVDDWE<br>MAADIFSTLKIEKSHNLIIPDWVTDDVYQKLETIFHDSFYYSYKTPKVQRLRAGVLIGNMITHMQERNNGQLNNLTKVFAYFTH	30<br>31<br>3.0<br>3

Sequence identifier	Amino acid sequence[a]	MM*
**Alfa-amylase**		
phosphatase-like [*Centruroides sculpturatus*]		
>TRINITY_DN2060_c0_g1::TRINITY_DN2060_c0_g1_i1::g.35180::m.35180 XP_067126432.1 lysosomal thioesterase PPT2-A [*Centruroides vittatus*]	>Tcham_22<br>YKPVVIIHGLFDKPENLEYLASYISKKHSRTNVTVINLYDGLESLSPLWKQIHKFGNSVSQIMKANPNGIHMIGYSQGGLIARGIISTLDNHNVNTFISLSSPQMGQYGDTDIIKVWFRDLIKKYAYKFFYTDEGQLISIGNYWNDPHHRDIFLNKSNFLPYLNNEIPHKQNLNYKKNFMRLKKLVLIGGPDDGVICPWQSSQFGFYTENENILPMREQWMYKKDIFGLKTLDKRKAIEVLTMAGIDHYQWHKNQTIFDNFIIPYLD	31190.83
>TRINITY_DN14204_c0_g1::TRINITY_DN14204_c0_g1_i1::g.66586::m.66586 XP_023224226.1 hexosaminidase D-like isoform X2 [*Centruroides sculpturatus*]	>Tcham_23<br>MMMTQVARHCLQHLVLSMWRRKTFLLAAVLGTSLVVICLQYGSVGENVVRGEFDNHEFPLGLSGPGTKSHHTLIPRRNLSAVAHPNNKQDGGVFRRGSAEIREGHLGIASRDQGAEVKREPRYRPIYQRDAQDSYGHSQNNLQLQNNVVQQKSHYQTYDGQLGRQQNNLYKQQSRSWQQQGLNHQTYYIPPLRLVHIDLKGAPPKISYFKQIFPMLKDAGANGILLEYEDMFPFWGQLAPVAAGNHYTKDDIKTLLSYASTYNFEVIPLVQTFGHLEFVLKIKSFQHLREVDDFPQALCPSKNNSFTLVQTIIDQVMSLHSNSKWLHIGCDEVYHLGYCPKCMRQDRDNLFLSHVERVARYVKEKHNIIPIIWDDMLRQMTSEKLKEFQLGNLVEPMVWTYVKDVYRFIPYNVWLMYSEVFPFVWAASAFKGAFGETLTIPNVKMHLENNEAWLEVMEEQQRKFTSFRGIAITGWQRYDHLATLCELLPASMPSLILNLLTVSHGYFSHQLIPKFQQILQCTIHSRSHLDLDSDPYLWQKAASCFFPGSAVFRLTQHHSEAIKRVTDYIYDVTVHKAWLTEFNVRHNISSPMRVDEGLADYSSIYYPLTSLVRTARDALREVFDEYTVAEWIEQNIYPYITKMEKVWKEGMDLKKQKTWPVRPLPPLEELKKYSVGLLESNDENKV	79642.17
>TRINITY_DN12890_c2_g1::TRINITY_DN12890_c2_g1_i3::g.71554::m.71554 XP_023236636.1 fatty acid-binding protein-like isoform X2 [*Centruroides sculpturatus*]	>Tcham_24<br>MADMKGKYKLVSSENFEDFMKALGVGMVMRKLGATSKPNVEISQEDDVWSIKTVTTFKTTEIKFKLGEEFEETRMDGSVVKTVVTLEDNKLIQKQFGDKEVVITRELDGDKLKVICTVDNIVSTRIYDKTD	14867.12
>TRINITY_DN13438_c0_g1::TRINITY_DN13438_c0_g1_i3::g.22841::m.22841 XP_023241316.1 phospholipid scramblase 1-like [*Centruroides sculpturatus*]	>Tcham_25<br>MSEKQKNAINNSSKNGVSIPLTPPENEEIPFADDDVNNEEIVKGVKGEWMPAPTSPIDCPPGLEYLCSLDKLFVEQEVQVLEALTGFETANKYSIKNELGQIVYYAVEETDCLLRNLCGPIRPFEMKLLNSSADEIITLSRPLRCMSCWCPCFLQELEVMAPPGTKIGLIKQDWSIMRTKFSIQNNEGEKKLSIHGPCCNCKCCTCCCDVNYKVYDAEGSDVGKISKKWSNLAKEAFTNADVFGISFPKDLDVTMKAVVLGACFLIDFMFFERSPEMKEVEK	31578.43

Sequence identifier	Amino acid sequence[a]	MM*
**Alfa-amylase**		
>TRINITY_DN14529_c0_g2::TRINITY_DN14529_c0_g2_i3::g.45629::m.45629 XP_023242599.1 myoferlin-like isoform X1 [*Centruroides sculpturatus*]	>Tcham_26<br>MKRLSHNVYTLGSQKQPEVENEENEGKEEKDKDQKGGTGDVLHQQAGTKSNRKEKFQVRVKIIEGRQLAGANINPVCKVSLHGVS<br>KQTRVVKSSANPWWDEIFFFHLETSPAELFSVNIDFHVNNARAFRSNTLLGSFKLDLGIIYEQPEHVFLNKWLLLSSMDDTVSRAMGY<br>LKFSAVVLGPGDECPNLATPEHKEDDDVESNLLLASGVQLKPASFIIDVYQAIDLPQMDSSMTDGVKKMLHVERNKDLVDPYLAVVF<br>AGHKAQSLIIHGKDHPEWNERIFLDFQFPSMCEKIKLVLTDWDRLTEDDIIATYLMSLNHISAPGEYGYLPTYGPAWVNFYGSPREYT<br>DLNSGCDGLNKGIGEGCAYRGRALLSLRTNLGSFSEVKSAPIEKTELLQVMPYCRRRKYRLHAAFIDASMVSVIDTPVEFEVSIGNFGN<br>KFEETLTPSPSTTQPTNAVYDGCNYYYLPWKEVKPCVIITSQWEDVAFRLETVNLISKVIDNLEQHIKTIQNDTKAGKPLPELAVQFIYM<br>MDDLLKDLDFKLPEPIPGQHIENELDNQLRQLRNEELRNIIKRAAELREKATDIEEAMFETESYLKTLQSLSIETQNSIPDVVIWMLSGK<br>KRVAYCRIPTHQILFSPDLERCGKYCGKLQSVMLKYPILEANNVDGDNSMKNNKNFSKYWKVPALLRIKLWFGLEVYDVFWSTSQTD<br>AEMSVFAETYENQVSVFGKWTTGSPAMTRPAWSDASGSIALPQDNFISPYGWKFDGDWFISPEPSMMYEDEVSHRVSTDEIYENE<br>SRMQGGPWGPALSPWTDFSGEPTIAKNEVQIPSGWIWEGNWEIDLNRAVDEDGWEYTTEAEESGYQPIEMTHHLCRRRRWTRQ<br>RACLRGISDYLSEKDFDTMSITSMDSQVVEDGWEYAPLFNMKFHAKERRMDLVRRRRWHRKLIAEDPSAPLVFNLAITPKKTSIVRK<br>FMGSEASTSDSKQTVEDAKLAMLSCPRLYLIYKHAHKFQLRAYVYQARDLIASDRSGFSDPYICVSILNQSMKTAIIFQSLCPIWDQTLI<br>FDDVIIYGDVDTVKCEPPTVIVEGYDQDAIGDDEFLGRSFATPFVHLNPDEYQPPKLEWYSLKRGKRPGGELLASFELLLADVGDLPF<br>MPPKRGDLYIVPAGIRPVLQRTAIEILCWGVRNMKTFEFTHISRPSIEFECGGTIVESKFIKDAKVNPNFDEPLLYIEMLLPIEEIYMPPM<br>HIKVRDHRQFGRKPIVGTHVIPCLHTFVIEPVYPSKDSFGSVVLTIGNEGDKQKTLSEETAIDIPDYPEEVKDKTDEETEGIDWWAKYYA<br>SLGENQKAGKYLEKGYNVIEVYRTELEKVDIFHGFTDFLNTFPLKRGKSKDMEESDTVGEFKGTLRVYPLPSDPKAPLPMKIFQGLTQA<br>TVEEIIVRVYIIKAFDLAPMDPNGLADPYVCIKLGKKKQDNKRRYKPNTINPVFGEMFELSGKLPIDKDLTISVWDYDLTSGDDLIGTTVI<br>DLENRYVTKHRATCGLPKTYQISGPNKWRDSKTPRQILMDVCRLHNLPPPIFNDEKVYLDNKVYTINDFEKTPPKNPHLGTMDQRLA<br>LHILNEFTLVSEHVETRALYNPATPELEQGKLEMWVDIFPKSLGKPGPTIDITPRSTTKFELRIVIWNTSDVLLTETNIMGEQMSDIYVK<br>GWISGIDDLQKTDVHYRSMDGEGNFNWRFVFPFEYLMAENNIVVKKKEHFWSLDVTEHHIPPKLILQIWDNDTFSSDDFIGSLEMD<br>LTCMPQPAKSSDKCSLQQMGYERKQTALDKFILKSQKPKVEYINIFEKRRIYGFWPCVSEDPEEGMILGGKIEMEMEIVSEEEVEKKP<br>AGHGRDEPNMNPVLDPPNRPATSFFWFTSPWKSFKHIIWKKYRLFIITSLIILFSVLLVLLFVYSFPEVLAYKIIHI	220918.32
>TRINITY_DN14684_c7_g7::TRINITY_DN14684_c7_g7_i1::g.61693::m.61693 XP_023242780.1 glycoprotein hormone beta-5-like [*Centruroides sculpturatus*]	>Tcham_27<br>IILGISLGINGDYSESSLGCYRRSYSYTAKQTDEFGRKCWDDITTIACWGRCDTAEIAHWKFPYKISFHRVCIHDDVKLTQINLRFCDPG<br>VDPSLRIYRYVEAKSCSCKICISGNTSCEPYRTMFPSE	14688.73
>TRINITY_DN14151_c0_g1::TRINITY_DN14151_c0_g1_i1::g.76216::m.76216 XP_023224525.1	>Tcham_28<br>MSDGGPKLGINGFGRIGRLVLRAAIDKNVEVLAVNDPFLDVNYMVYMFKYDSTHGRFKGKVEAEGGLLVIDGKKIHVFQERDPAQI<br>PWGKIGVDYVVESTGVFTTIEKAKAHVTGGAKKVIISAPSADAPMFVMGVNHDKYETSMEIVSNASCTTNCLAPLAKVINDKFGILEG	36191.43

Sequence identifier	Amino acid sequence[a]	MM*
**Alfa-amylase**		
glyceraldehyde-3-phosphate dehydrogenase-like [*Centruroides sculpturatus*]	LMTTVHAVTATQKTVDGPSGKNWRDGRGAGQNIIPASTGAAKAVGKVIPELNGKLTGMAFRVPTPDVSVVDLTCRLAKDATYDEIKAAVKAASASDHWKGILGYTEDQVVSSDFIGDTHSSIFDAGAGISLTKNFVKLISWYDNEYGYSNRVVDLAKYMYKLG	
>TRINITY_DN14145_c0_g1::TRINITY_DN14145_c0_g1_i1::g.76082::m.76082 XP_023244832.1 cysteine proteinase inhibitor 8-like isoform X2 [*Centruroides sculpturatus*]	>Tcham_29<br>RTFVGGWKKVDDPNATKIQEFAKWSLSAMNDDSGVLKICEAETQVVSGINYKISFLLGEPGDENNGKSYCEVMIYDQVWTKTRKLTSSSCKPQKTCPTTE	11173.64
>TRINITY_DN12308_c0_g1::TRINITY_DN12308_c0_g1_i2::g.43720::m.43720 XP_067120019.1 thyrostimulin alpha-2 subunit-like [*Centruroides vittatus*]	>Tcham_30<br>ENVWEKPGCYRVGHTRNISIPDCVEFQVTTNACRGYCISFAVPSPEGTIKANPNQLLTSYAKCCNIMETEDVRVQVLCLDGFRDLTFKSAIKCSCFQCLKY	11382.13
>TRINITY_DN11671_c0_g1::TRINITY_DN11671_c0_g1_i1::g.3144::m.3144 XP_067121879.1 lysosomal aspartic protease [*Centruroides vittatus*]	>Tcham_31<br>ENLVRIPLYRFQSVRRHLHEVGTPIKLVKHQLFDSNGPFPEPLSNYLDAQYYGPITIGTPPQNFKVVFDTGSSNLWVPSKQCKWTNIACLIHNKYDHSKSSTYEKNGTSFVIHYGSGSMTGFLSTDTVAIDNIPVKHQTFAEAVTEPGLTFVAAKFDGILGLGFDNIAVDGVTPVFTNMVRQRLVDRPVFSFYLNRNPDASVGGEIIFGGSDRKHYKGNFTYVPVTRKGYWQFKMDGISVGSGNGKYCAGGCQAIADTGTSLIAGPTDEISKLNKEIGATPLAAGEYMIDCDLIPKLPTVTFVLGGRKYPLQGSDYAMKITQFGQTVCLSGFMGLDVPKPMGPLWILGDVFIGRYYTEFDYGNARVGLAETV	40839.59
>TRINITY_DN12937_c0_g1::TRINITY_DN12937_c0_g1_i1::g.30231::m.30231 XP_067138356.1 lysosomal alpha-mannosidase-like [*Centruroides vittatus*]	>Tcham_32<br>VCGYESCPVPKPNTLNVHVICHTHDDVGWVRTVEEYYQIFVKHILNSVVRHLLVNETRKFIYVESAFLHRWWSEQSELSKYLTRKLINDGQLEIISGGWSMNDEATTHYLAIIDQMTLGMRWLNETFGKCATPKIAWQIDPFGHSREQASIYAQMKFDGMFLGRIDYQDKQFRQQTRNLEFTWKTNPGYNLDTDIFTGILPNVYWPPHGFCFDDLCGEPEITSFNINRKARQLMDVIRFQANYYRTNHTVLTMGMDFHYRDADRWFRNLDKLIDYINDLQESGSNINIFYSTPSCYLHSLNQANLTWPTFTEDFFPYASDPDAYWTGYYSSKPAIKRFTRYSNGFLQACKQVIALNNISQDYSYNLQKAMGVMQHHDAVTGTEKDYVAKDYAVMLSLGVDDCELAFNEAYRNIIEKNEKKFIEHFFCQNLNISECYWSENEVGFFLVVYNPLSRGITEYVRLPVVAENYLVIDSSNNEIPIQITPIPNQVQLLPERLSWADRELIFPVIVPPLGISVYEVLQRGGLS	107778.03

Sequence identifier	Amino acid sequence[a]	MM*
**Alfa-amylase**		
	ENTNQETNVGDVTIGNENLKVVIESRTGLMKEMILNDLHIPLRQSFYWYEAQPGFRHERPSGAYAFNPRFDYAFSLHENVTFRVNKG<br>PLVEEIHQTFTPWLSQTISVYKGEHHVEFNWLVGPIPIEDRIGKEIITTYETSLATDGIFYTDSNARQTIKRKRDFRSAFPLNLTEKIASNY<br>YPINSWVHIKDEERKLQMTIIPDRAEGASSISDGSIEIMLHRRLLFDDGFGVEEPLNEIGIDGKGLIVRGKHWLLLRPIGDSLYRQLAQR<br>MFLPPILSFTFGNLTDLEVLDQFSGLNDRLPENINLITLESLGTNILLRLEHFYGFGEHPIFSEPVNVSLAELFSPFSIVNAKEMALGANIFL<br>DETEKLTWNDQAQFADGNEEVENLTISFQPMQIRTFLLDVQT	
>TRINITY_DN13929_c0_<br>g1::TRINITY_DN13929_c<br>0_g1_i2::g.70098::m.700<br>98 XP_023244342.1<br>beta-hexosaminidase<br>subunit alpha-like<br>[*Centruroides*<br>*sculpturatus*]	>Tcham_33<br>HNYDADGRLESQVKETPTVGMPWPHPQQYVIYKSYMGFKPDNFKIINNSENCTILINAIKRYQKLLFLDKCLPQDNLSHDDHSNSIEH<br>QTINFIKSDNFAGYLYNISVKINHCEQWPHMNMDEQYLLKINSPDIPGQALLFSNTVWGALRGLETLSQIIYKNADGMFIVNSTFILDY<br>PRFSHRGVLLDTSRHFIPVNILLQNLDAMAQNKMNVFHWHIVDDQSFPYVSLTFPGLSAKGAFNPKTHIYSFSDVANVINYAKERGIR<br>VVPEFDTPGHTLSWGKGQPGLLTPCYKQGVADGTYGPIDPTVNSSYKFLTSFFAEVAKVFPDTYLHLGGDEVSFDCWKSNPNITAFM<br>KMHEFGDDYSKLEEYYETRILKIASALKKSYIIWQEVFDNNVKVKPDTVIHVWKNPQADELTKVTAAGYNTLLSTCWYLNYINYGSDW<br>TSFYKCDPYAFNGTEKQKTLVLGGEACMWGEYVDGINVMSRTWPRASAVAERLWSSINVTDIQSAIPRLEQHRCRMLKRGLKVEP<br>VNGPGFCKCDYVAN	61<br>61<br>9.0<br>2
>TRINITY_DN12002_c0_<br>g1::TRINITY_DN12002_c<br>0_g1_i1::g.40028::m.400<br>28 XP_067124461.1 acid<br>ceramidase-like<br>[*Centruroides vittatus*]	>Tcham_34<br>LLPPTGQCVKDAYPPSLKNKVPIYAINLDLPPEVRWNKLVYDKRKELNATINVIKNLTKSIFKGKFVWLVDNGLPLILKSLPEPYSTEIASI<br>AKITKIPLGEIILYNIFYEIFTFCTSVIAQDEKGNLYHGRNLDFGIFMGWDAKNHTWLLTETLRYTVAQLEFYKGNKLLYKSVGFAGYVG<br>VLTAIKPKHFTLTVNERFNINGGYIGILEWILGDHSSHWMGFLTRQVMENGYNYSTVKHILATTKLMAPVYFILGGNKGGEACIITRD<br>RGTNKSDILTMENGWYVLQTNYDHWNKPPFFDDRRTPAIKCLNKMTQRNASFAGLFNVLSTQPNLNKLTAYTSLMEAKTGKLETYF<br>QYCNDPCWPW	41<br>87<br>5.5<br>4
>TRINITY_DN14578_c6_<br>g1::TRINITY_DN14578_c<br>6_g1_i1::g.46841::m.468<br>41 XP_067126965.1 LOW<br>QUALITY PROTEIN:<br>calcium-dependent<br>secretion activator 1<br>[*Centruroides vittatus*]	>Tcham_35<br>MLEPSSSEEESDNETVEDESSEATALPPASTNLELPRSSSVSRSLSPSGAGDTLSVGSGSQQWTNSQRPSSPSPSLVSEKEKEKEDPEKS<br>IEKEEEERKKRLQLYVFVLRCIAYPFNAKQPTDMTRRQTKVIKQQLETILGRFQSFLKGETQIAADEAFQNAVQNYTEVFLKSDRVMR<br>MVQSGACSAHDFREVFRNNIEKRVRSLPEIDGLSKETVLTSWMTKFDAIFRGDEDSRKPQSRIQQNIASELILSKEQLYDMFQNILGV<br>KKFEHQLLYNALQLDSADEQAAAIRRELDGRVQKVNEMEKNRKLMPKFVLKEMESLYIEELRSSINQLMVNLESLPVSKGNFSDSKY<br>GLQKFRRYNHSRQSLLSASANFFPENSLHKASSYLQAPIWIIIYEALMIAFTSISQSSLSKEGLSEDGETMLSKLDIVLSFTLEVVVMEVK<br>GLKSLAPNRIVYCTMEVEGGEKLQTDHAEASKPMWDTQGDFTTTHPLPIVKIKLYTESPGLLSLDDKELGKVIIKPTPLSPKSPEWYKM<br>IVPKNVPDQDLRIKITVRMDKPQNMKHCGYLFAQGKQVWKKWKKRYFVLVQVSQYTFAMCSFREKKCEPTEMMQLDSYTVDYIE<br>PVPDLEGGRFFFNAVKEGDSVLFASEDENECHLWVMAMYRATGQSHKPTPLVQPLPSKNSTISRLQGDADRARKHGMDEFISADP<br>CNFHHHALFRLLQTQALEYRLADIFCSLGWYSPGQLFVLDEYTARYGVRSCYRHLCYLNDLLDRAEKGIMIDPTLIHYSFAFCASHVHG<br>NRPDGIGTVTVEEKDMFAEIKERLRILLENQITHFRYCFPFGRPEGALKATLSLLERVLMKDVMTPAAPEEVRNVIKKCLENAALVNYT<br>RISEQAKNEGAESAPKQDETEEEPAESNKNLEDLIHLAELCVDLLQQNNEHYAEAFAWFSDLLVEHAEIFWSLFAVDMDAVLAQQPP<br>DTWDSFPLFQVLNDYLRIDENLCGGRFHQHLRDTFAPQVVRYVDLMESSIAQSLHKGYEKERWEIKGHGCGTSEDLFWKLDALQTFI<br>RDLHWPDEVFAKHLEQRLKLMACDMLESCINRTLQAFQQWERKGSRWISTDYIVPSEMCAMINVVLEAKNQSLKLCTVDGVDMH<br>QYHAKIDQLVDETLNLMMTGLINKLTSVLEATLNKLSRYDEGSILAPILSLTYRQGMSSSGREVGLAYINFARNSMEQLRQKVTDELW	15<br>13<br>84.<br>31

Sequence identifier	Amino acid sequence[a]	M M*
**Alfa-amylase**		
	VLSLFEQWYASQMQMLCTWLSERIDRGLHSFQLSALSHIGKKLYSDFELQGIEEEKLNSKIYQTISSRLQLENATASVSEAAHREKEDS DINSMK	
>TRINITY_DN11865_c0_ g1::TRINITY_DN11865_c 0_g1_i1::g.25384::m.253 84 Tja_SC_01	>Tcham_SC_01 FDRDRQHFDFHDEVTVIGEYHKENLTTNDFKEDRDCNRLNQPCNSDSQCCSYGERCIGTGSEYVCRPDPGP	82 61. 88
>TRINITY_DN14235_c0_ g1::TRINITY_DN14235_c 0_g1_i1::g.62421::m.624 21 Tja_SC_01	>Tcham_SC_02 KSVGEEARGCNKLGKKCDSNSDCCRYGEQCINSGSGYSCKVDPGP	47 31. 23
>TRINITY_DN9629_c0_g 1::TRINITY_DN9629_c0_ g1_i1::g.2710::m.2710 XP_067134649.1 superoxide dismutase [Cu-Zn] [*Centruroides vittatus*]	>Tcham_38 MSSCPLKAVCVLVGESVSGTVHFSQLKPNCPVTVEGEVRGLKPGLHGFHVHEFGDNTNGCTSAGAHFNPHGKTHGSPCDEERHIG DLGNVEAGPDGVAKVNITDSLITLYGPLSILGRSMVVHADPDDLGKGGHELSKTTGNAGGRLACGVIGITK	16 02 8.0 7
>TRINITY_DN13402_c0_ g1::TRINITY_DN13402_c 0_g1_i2::g.22952::m.229 52 XP_067124462.1 copper transport protein ATOX1 [*Centruroides vittatus*]	>Tcham_39 MASTHEFQVEMTCEGCSGAVKRVLDKLGDKINKVDIDLGKQRVYVESSMSSDELLSTIKKTGKNCSYIGEKS	79 18. 05
>TRINITY_DN12390_c0_ g1::TRINITY_DN12390_c 0_g1_i1::g.43909::m.439 09 XP_067142637.1 putative protein-lysine deacylase ABHD14B [*Centruroides vittatus*]	>Tcham_40 MTSNDLCIPSFWQNVNLVANEIPVDVQETAKKLNIVTKTVKVDENEVFYREVEPFKGFDIPTTITLLLLHGAAFSSETWEKLNTIQLLSA LGYRTVAVDLPGFGNSPNAKVDKQTFISKLVKTLNLNSPVVISPSLSGLYSIPYLLQNWLEMGGYVPVAPVGTDLLENIEVSMEGSDER LKRVPDSLQQFLHPPLPNLDKYLVPTMVVYGEKDRKKNSALLTLLPCSQAREIPNGKHPAYLDNPALWHQLLYNFLKQIEISKTK	29 59 3.1 8
>TRINITY_DN13742_c0_ g1::TRINITY_DN13742_c 0_g1_i2::g.70668::m.706	>Tcham_41 LDNGLAIKPPMGWLSWARFLCNVDCKNDPNNCISESLYMEMADRLASDGFKDAGYEYVNIDDCWMSKERDSNSQLVADPDRFP HGIKALADYVHSKGLKLGIYEDIGTETCGGYPGSAGHFDIDAQTFAKWGVDMLKLDGCYAEPKDMDKTYPQVTAALNRTGRPILFSC	43 91

Sequence identifier	Amino acid sequence[a]	MM*
**Alfa-amylase**		
68 XP_067142793.1 alpha-N-acetylgalactosaminidase-like [*Centruroides vittatus*]	SWPDYQRASGMSPNYTKIAENCNIWRNFDDISDSWQSVLSIIDYYAKEQDILRAAAGKGHWNDPDMLVLGNFGLSYDQSKVQMA<br>LWSVMSAPLLMSNDLRNLKPEFKNLLLNKDLIAINQDSLGTMGKKLLSVDNIEIWSKAINPTSAQDNSYALVFFNRRSLGGPVNVSV<br>VLNNIGLTHDAGYECYEVFDHKFLGTYKPENTLTVQVNPSGASTVVAKVQNQI	9.59
>TRINITY_DN11910_c0_g2::TRINITY_DN11910_c0_g2_i1::g.54326::m.54326 XP_067135991.1 CD9 antigen-like [*Centruroides vittatus*]	>Tcham_42<br>MALQGCYACIKYLVIIFNFLFWLAGIGVLALSIYSYIKGSTYASGSEDIKSFFTPLYILMAAGSLMTILGFLGCCGALRESQCMLGTFFFLV<br>LVVLVAEITAGVWAILNKKETKKIIADELTKIIQKQYGNSTVANNTIDAIQHDLHCCGANKPEDWANSNFNRLKTNVNLFDTLTQSVGI<br>YEVPESCCKNPESCIRVPLKGGVFGESIYSKGCIDAMEEFIKNNLNIVVGIGIGLGVIQILGLIFSIVLCCAIKNGEGVSYHV	28734.78
>TRINITY_DN14115_c5_g4::TRINITY_DN14115_c5_g4_i1::g.74075::m.74075 XP_067137151.1 protein disulfide-isomerase-like [*Centruroides vittatus*]	>Tcham_43<br>EVREDDVLVLKKDDFDQQIKTYKYILVEFYAPWCGHCKALTPEYAKAAKQLLEEKSEIQLGKVDATEETELAEKYKVKGYPTIKFFRDGE<br>PIDYTGGRTAKDIVNWLKKKTGPPAATLSTVEEAVKFKDSQDVVIIGFFKDSESKLAKEYLDTAFTIDDYPFGITSDENVYKEFKVEKDTII<br>LFKKFDEGKNEFEGEITKDNIKKFIKSNSLPLVIEFSQENAQKIFGGDIKLHNLLFVSKKSEDFDEILKKYRNVAKDFKNKVLFVLINADED<br>DHEKIIEFFGIKKAEQPSMRLIKLEGGMLKYKPETTEFSEENIQTFVKGVLDGKIKQHLLSQDLPEDWDKHPVKVLVNKNFDEVAFDKT<br>KDVIVEFYAPWCGHCKQLVPIYEELGEKYKDREDIVIAKMDATANELEHTKIHSFPTIKLYKKETNEVIEYRGERNLEGLKNFIDSGGKKE<br>KVEKVRYTNILKLEDF	54506.31
>TRINITY_DN14258_c0_g1::TRINITY_DN14258_c0_g1_i2::g.61568::m.61568 XP_067140417.1 polyunsaturated fatty acid 5-lipoxygenase-like isoform X1 [*Centruroides vittatus*]	>Tcham_44<br>MGNLIPNLKSDYIIEIKSSDRSDAEPGTIIWLILHDERGLKTNAIRLNISVKNEKEMYTFQVTCPLNFDNVDKIEIWRNCYGVGDVFYLEY<br>LTVEKKNGKKLWFPLHRWISSQHRYYFRVYDCCLPQHELSLTQRNDELNDKRKLYEYTEHIEGGPLQIKILPEEEDFTTYLFQWELIHN<br>CKKEKLSIVVEMTSGKWQTFDEIKSFLRKNYTEPSCVNRWNDDSWFAMQRLQGINPVSIKLCRELPENFAVDGGELEAFLEGLSVQE<br>ALENKKIFIVDHKIMKNLPCKDDKLVCAPIAVFYLNSNGEILPIAIQLFQDRTVDNPVFFPSDPPYTWLLAKMYFNNADALYHRACLCV<br>GRTHLLMESLWISINRNLSPSHPLYKLMAPHFKYIFSINKLMLEKFLVMGGWIDRVTTIGVRGLCHLIARRFPNWKFEEINIPKEIESRG<br>VNDPCVLPHYPYRDDAIILYDSIFDYVGKIVKNYYDYPKKISQDTELQNWRKELVQKKKDGGCQLQGVPGAENGFSSRLQITDTITSIIA<br>NCTIFHAAVDMPQYEECAFVPNYPLLLTGPIPRSKVKNTLLIQLKKFLL	68279.95
>TRINITY_DN13939_c0_g1::TRINITY_DN13939_c0_g1_i3::g.70294::m.70294 XP_067144653.1 neuroendocrine convertase 1-like [*Centruroides vittatus*]	>Tcham_45<br>MSYPALLLLTTLLTYVKLHDDNGRYTNDWVVRITGGSYVADELAKELGYRYLGELKGFKDTYLMRRIDHPQRSRRNSAVLTKRLSDDR<br>RVVWAEQQVIKPRVKRGVFRTKRGGDDFPYKDDCSNKFKDPYWSHQWYLCDTRDRQDLPRIDFNPSSVWDGMNITGRGIVLTIM<br>DDGLEWNHTDIFPNYDPQASWDSNDNDPDPFPRYDEFDSNNHGTRCAGEIAMVANNDKCGVGVAYNAKIGGIRMLDGDVSDA<br>VESVSIAFRIDHIDIFSASWGPSDDGMTVDGPKRLAIEALEKGIFQGRNGKGVIYVWASGNGGSKGDNCNCDGYTSSIYTLSIGSASQ<br>HGRFPWYGERCASTMASTFSSGAYTDQKISSTDLHNQCTDDHTGTSAAAPLAAGMIALVLEANGNLTWRDVQHIVTWTSDVEPLK<br>DNNGWKRNAVGLMYNSRFGFGLMDAESMVKTALKWKTVPPKSITTARPQTELPQPVSSGRETEIYFETDCGKNTENEVRYLEHVQ<br>VTVDIDYNRRGALELYLISPSDSKTMLLSKREKDKSAIGFKNWTFLTVHLWGEQCAGRYKLLIRDKIGEDNRGEVKMAILTLHGTKDIP	78126.09

Sequence identifier	Amino acid sequence[a]	MM*
**Alfa-amylase**		
	EHMKNGKTYPEDWDADKILDKSEYEDESDAERNDLSDRINQLKEDKGKDLDWDQLIGEKLRSLSQKNPARLLNDFEEERDFDYVQDYY	
>TRINITY_DN14628_c5_g1::TRINITY_DN14628_c5_g1_i6::g.59198::m.59198 XP_067145024.1 synaptic vesicle membrane protein VAT-1 homolog-like isoform X1 [*Centruroides vittatus*]	>Tcham_46<br>MADTNNAEQEKGEEVQNAEEQKQESTPPPKEMRAVVLTGFGGLKSVKVMKKPEPTAKEGEVLIRVKSGGLNFLDVMVRQGVLDNPVKTPFIMGFECAGVIESLGENVTDFQVGDEVAALSEYKAWAELTAVPAKFIYKLPPGMSFQDASALLLNNVVAYILLFEVGNLREGNTILLHSAGGGVGQAVAQMAKIIPNITLIGTASKHKHEDIKSSVTHLIDHGVDYVQEVKKLSPEGVDLVLDCLCGEDVNKGYSLLKPLGRYILYGTGSIVSGETKSFFSFAKSWWQVDKVNPIKLFDENKSISGFNLRHLLYQQGGHEYIRGIVEKVFRLWKEGKIKTVIDSTWAFEDVPEAMQRLHDRKNVGKITLDPNMEPKPKPQPTKGKEKEAKDGKATSVPSGESTDTEKEAKEESSPPPNSS	45968.49
>TRINITY_DN5402_c0_g1::TRINITY_DN5402_c0_g1_i1::g.63866::m.63866 Cbi_TRINITY_DN166_c0_g1_i1.p1 XP_023235202.1hemocyanin II-like *[Centruroides sculpturatus]*	>Tcham_47<br>QEILGHSFVARRSIMVLHERQIRILKLFKHLSLATVGGEVPRDQRDSRLADLNLSVRPGEIFSCFHPDHLEDARHLYEIFYEAKDFDDFVHLAQQARTFVNEGLFAFAAEVAVLYRPDCKGLSVPPIQQIFPERFVPAATLAEIINKASTKPAGDESPILVDVKDTGNVFDPEYNLAYFREDVGINAHHWHWHLVYPATWNASYFGKEKDRKGELFYYMHQQMCARYDCERLSNGLNRMVPFHNFDEELEGYAPHLSHLSSGRYYAARPAGLRLSDLKDVDVQEMERWRERIMDAIHLGHIEDRDGKEIALDEEHGADILGDLIESSYESKNRVFYGSLHNMGHVMMARIHDPDGRFQETLGVMCDTSTSLRDPIFYRWHRFIDNIFQDYKATLPVYTKDQLHFAGVEVTDIHINARIPNVIDTFMLRDELEMSYGFIFGSDGSYKARYQHLDHEHFSYTINVQSTLPAKKEATVRIFLAPKYDELGNIIPLNELRRLCIEMDKFKTELTPGINTVVRNSVDSSVALSREYTFEELERGDGITEDRTEYCSCGWPQHLLVPKGNPKGMVYYLFVMLTDRDQDKVTDGAGDHSICSDAVSYCGAKDDKYPDKKSMGYPFDRPLHYETLEGFLTKNMHVQEITVKFHP	74550.17
>TRINITY_DN13735_c0_g1::TRINITY_DN13735_c0_g1_i1::g.71015::m.71015 Cbi_TRINITY_DN37835_c0_g1_i1.p1 XP_023224715.1transferrin-like [*Centruroides sculpturatus*]	>Tcham_48<br>HSHKVRFCAVEKNLPLCLQMAEEHSDLVECVSRIDGLSCMESIAANESDIMSIDAANLFVAGNNFNLSVLAFEENNGEPYKYRSVALVRKSLELSSLADLKGKKSCHTGVGRTAGWQIPIAKLTGKGIMTPDCRGELETVENFFNQSCAPGKWSNDPIVDAELKKKHPSLCSLCENSELCSKSDLYSGYEGALRCLHDDRGDIAFSKTEVVEKYIQDNPAFVEEHEYLCIDNTRKGLREGADLSCSWGVRPSNAFVICSHQSPEERERYYSLLRQLFARYSSNIPQIQNQERPEWFTKFFVSSVNVSDILQATPEQQNPLSYLGEFRYTMDRESDECPPKTVRFCTTSEQGVNKCNDLNKMTKSRRINAGVECVLEASVADCINAVKDGRADLITLDGGDVYRAGRYSNFVPIASELYGEENANYYSVAIIAANSEINELEDLRGLRSCHTGMGRTAGWVMPIGFLADKGLISKQQCDKAAAIADLFSASCVPGANDPKYNPSGSGSIKLCQQCIGDSNGENKCARGSVERYSGYAGAFRCLAENSGDISYVRHTTASEYTDGNSEESWAKDLKSENFKLLCRNGSKDSIENYKNCNLGRVPSHTVMISSLATPEKRLCLTEFVAALNKWFGLNSTNYNLFKPYGGKSDLLFKDATTSLRAVPSQYTYKDVLGEEYSIAVESVDPLLCTQEA	76542.91
>TRINITY_DN14172_c0_g1::TRINITY_DN14172_c0_g1_i1::g.75383::m.75383 XP_023242593.1 transferrin-like	>Tcham_49<br>QTIRKIKFCLTKTNLQYCQQLSNLNAECVIGTDKIDCMRLVQDGEADIVLLEPPEIYTAGSYFNLEVAATNLVTDELLTYRFRTVAVVRTDGEIKKLDDLKGKKACFSSVGEAEGWNIPIGIILNKVNNIECKNRNELSTISSYFGPSCAAGIWSSDVEVNEKLKKENKNLCSICKSPDSCDDKDEYFGINGSLKCFEDNTADVLFTTVEHIKEFDNSKYSLLCLNNTIVPVNDENPCVWAEMPTDGYLTKEGNTGDISASLKKMLDDVNAQESSLSWLKYVFGDYKKIAEITEERKDWKNYLGNYIMTIDRAVNCEKNVTFCTKSKDELKKCDSFTKAAYGRSLKPIIECVEKNSS	76179.68

Sequence identifier	Amino acid sequence[a]	MM*
**Alfa-amylase**		
[*Centruroides sculpturatus*]	DDCISAIKNRNADLITLSGRDVYSAGKYDNLVPIASEEYSEENNISYYAVVVVKADSDIQSLKDLKGKKSCHTGMGRNAGWIIPIGEFM<br>KLGIINKKECDVAKSVANFFSESCIPGAKDKKYNPKLSGVDKLCKLCKDPDKCSMDDEYSGYFGAFRCLAEGIGDVAFVKNSTVLSLTD<br>GNSQLPWAKDLKSSDYNLLCPDGSKANVSDYLKCNLAPIPSQRFVVTSNKMDQSDRLNTVYMLTSAGKLFNESSPIFSLFESYNNKSD<br>LLFKNSAISLKSVKFDSKYDEVLDKDYLEALKLTDPKRCNSATTAVATLGVVICSALLSFFM	
>TRINITY_DN14049_c0_g2::TRINITY_DN14049_c0_g2_i2::g.13009::m.13009 XP_067138103.1 endoplasmic reticulum aminopeptidase 1-like [*Centruroides vittatus*]	>Tcham_50<br>TLDVLMSESPGHLLFQNYIQNILKSVVKAVNWENEEQHLRKKLRSLVLYAAAEHGNSEIIDKAKEMFNNWMKKGVKVEPDLRNVVY<br>MVGIKFGTQEEWNFCWKKYQESLIPSENKLLLNALGSSRDIWQLSQYLNYSLDKDTIRPQDTVSVISRVSYNPVGRLLTWRFVRDNW<br>SKLLDTFGQGSFAMGSIISSVISDFSTRFDYNEIKAFFDKVDVGVGARALNQSLERIRANIYWRENVESSVVQWLRQQTNQNIQS	29998.12
>TRINITY_DN13980_c0_g1::TRINITY_DN13980_c0_g1_i3::g.70110::m.70110 Cbi_TRINITY_DN3945_c0_g2_i1.p1 XP_023233706.1neprilysin-2-like isoform X1 [*Centruroides sculpturatus*]	>Tcham_51<br>IEASKICNSRECVFAAADLLTYMDHNVDPCKDFYSFACGRWEKSNPHITSVTTRLTEMTKNVHLRLKNLFESPDNSYEPSHWRKAKKF<br>YRTCLDLDRREKFDKYLFVNDINKLGGWPVLKNVNWTEDYFDWIGTLEKMFNMGFNKNILLTVSIGADYKNTSIRRVFIDQPTFGM<br>KKQLFLHGKFVNAYMKMILKIIVLLKPHSNIHEIAYEISRALAVERKLAQLALPILNKRTPQETYHLVKIKQLMEYMPQINWLRLINSFLP<br>DLILTENDNVVVKEPKFLRQFSIFINTLNKERRRDLANYMFYRMAIANTINLPKLFRWLYFDFRSTLVNMEIHPPVWSLCIRATTSVFSY<br>SASSVYTKQFFRKETKEAVNDMVRRIQERLIYILKHVKWMDNITRERALEKAHSMSVVIGCSDDIYNDLMINKHYEKIKVGNSHYENV<br>RNARKYQYYLLMEGLNKPVNKTVLRGHTRILMANAYYSPFLNTMNIPIGILQDVMFNEHYPNYINYGRLGSIVGHEMTHAFDDKGA<br>WYDKNGNWKLWWADDTYQKFMKTSECLVKQYNRFYITELHRRLNGTQTLGENIADNCGVRESYMAYKTWVTDHHISEPKLPGLD<br>YTPYQLFWIAYATSWCSAYSKNNLRSRVVYDVHPPARNRILAIVSNSKDFAKDFNCPNGSPLNPSTKCTVWI	80881.41
>TRINITY_DN14429_c1_g1::TRINITY_DN14429_c1_g1_i1::g.17513::m.17513 XP_067141325.1 hemocytin-like [*Centruroides vittatus]*	>Tcham_52<br>TCLEVCEAILLQERGKKTFRISYHRVYQNEERFTHHVIIVVDNYSYNLPQKELVIVIKDDVETVVSLSASSSYYNEQGAVSVQVIGDAIQF<br>RSEQKGFTVIWRERNIIDIELDACEIDRVEGLCGYFATRAIHDEYRGKDGTCFNKDAEMCSDWTFDKSNDVCGSIYEMENEKLLTAER<br>VCDEISKKVYYTCNVDVTEQTSDNSFRANDKVCKYVIYPNCTTDNRLCQCEALKYLLVEASCKNGIADLLLEYNCRELCPEGFQWDMC<br>DADNTCEDLTFNGNSKKKIAGCFCPKHLVRKGDKCIPSHLCENCVCDIYGDPNYQTFNKRNYRFLSTCPGPYVLVQRKLPSNEPFFKV<br>LSVNEKCIEYVNGTCISGIHVYYNNTKIEIYRNTTVLKNDRLYTSYCEDTSCNFNGIDFKIETETNIYIVKLVEIHVVIEYKIGNATLHIAGEV<br>SIKFSSYTFFNNTEGLCGSCNGDSSDEFRLRNGSVVDDISTFALDWLEEKNKSLCEIYKTDNCTNIQNVSKCEFPQHLNCSIVDNNRYYS<br>RHSGSCKNDLRCNDQITDSDICKYKFLYADNCCAFRLPMDKWLEIENCVIPCKENRTFRCVDICETKCPAERNEQCEFGKQMRMVCA<br>CPDGYVERNDKCIKNTECDACSPKPTLNIPARYFNIDNCTNLDPIVNMYECNGKCNDDFSHYTIERPYNFKEPIDYIYKKKCHCCQPSK<br>YINDIITLTCINGLKKNIIIKKPIDCTCIECNATVI	86474.11
>TRINITY_DN14011_c0_g1::TRINITY_DN14011_c0_g1_i2::g.13144::m.131	>Tcham_53<br>MNYPYHNFYPLPNYPPVYSMMNEGQIPNIQNQSSSQQEIYPQVPTYPFHPINPYPMGGGPICPPVPAGSIYFPPTEFVIEPVTTASPY<br>PLYPGIPNTAQQPLAGGIFHPNIATIDPTYPPTLDPGFAPGIQYPYQYPSVYSSWKLKMPEGTVKPYHPFNPEDDAKQLRSAMKGLG	5188

Sequence identifier	Amino acid sequence[a]	MM*
**Alfa-amylase**		
44 XP_023220268.1 annexin A13-like [*Centruroides sculpturatus*]	TDEDAIIDVLAHRTNQQRQEITTAYKTQFGRDLVEDLNSELSGRFEDVINALMMPVVEYIAKELHNALSGAGTDEDVLIEILCSSENKEI LEIKEAYQTLFERSLEEDLVSDTSGDFRRLLVSMTTGARNDSYADPDGAKQDAQDLYNAGEGQWGTDESVFNMILATRSWDHLAM VFYEYQQLTGHTMSEAVEREFSGDVKQGWLTVVHCVEYRPSFFAERLNKAMKGAGTCDKDLIRLIVTRCEKDLVQIKQEYYDQYDKT LEEAVESDTSGDYKRMLLALVSGV	1.20
>TRINITY_DN14311_c0_g1::TRINITY_DN14311_c0_g1_i1::g.5231::m.5231 XP_067138118.1 complement C3-like, partial [*Centruroides vittatus*]	>Tcham_54 RKIFIAAPNTLHLDTNERIALVAYGIEANEQFDVYLQDYPRKLKQFSSSKVTITNGRPVFVNIRFTADDIPDIELTDAKEKVYVILVVKGN NFNEEFKFLVDYNAGFVFLQTDKPIYNPQQDVQIRIIPLNERLLPKNTDVHVKIKNPQNVIVYENTYKEPWKTHSVYFPTVNFKFPPYP LFGEWSVEASYGYKLQQNSSVSFKLEEYVLPTFTVELETPNVILPQHETVDILVKAEYVYGKKVDGTAIFKISVKNEMGVIEVGHTKTKE VEEGESRLSFSLDEVTQHPEINKFPHGSKLIVEVSVVDMATGNKENAINDQCEFSKSPYLISFKNSLKDFKPGLISAIVADVAYVNGKPA ANVPTYISVVDSNNNKIEVAKQRYTSDENGHVLYHVNTPIGPEFFTVTVTTNDTRYKVDQQFSAKHVLLKSTSKGFIVISRTEGTRKVK VGDKFTATIFTVPPEKFENLFFLVMAQGKIIHISTVPKGQLLQRQLEFPVTADMSPVLRVVVFSLHENELVVDSLRILVENQCSRFAEVE IKTDFRTTEPGHDGKFILEGEPDTKVGILAVDNAVYALRNKDRLTKEKLFKTLATRDLGCGPGGGINSEAVLSNAGLIIFGNFITENKDK TAVGCSERIRRKREAIQDMVDGHEEEDKKCCFLGTRSDKHKRSCEERAEILKTFLKEKEENDTCIPAFLKCCLKLAPQLLASTDDNKESH SSELSRAFDKPFEDLEFIEESEEADFERTLIRRDFRETWLFKDYIIGPDRTTEVITSLPHSITTWVIQAISLSDQWGICISEPKKIVSFKNLFIH LNIPYSVVRNEQVEVQATVFNYNSQRIPVTVYMYGVEDLCTGVQAGEKSERKKIWVDPHSAENVGFPVVPLKDQKFSIHVVALSPV GSDVIIKELNVVPEGITKVQDVVIILDPTNQQKRKRRNVQTEVYTDSIDESQKLQVTAIKLLPTQNFVPGTESCVVSAIGTEYGPTVETVI HDPETLLRMPVGCGEQNMIYMGPTLYTLRFLKVKGDVTPEREGKIYHFVREGYKRQLTYRKQADGSYGAWATTPSSTWLTAFVMK VFCQANKFIFIDENVICSGIKWLISRQKDDGSFEDAHPVYHRDMMGGNNGKIPMTAFVLIALHECSCQTEELSVARLKAELFLEIALPA INDPYIMAVTAYALSLSESETKYEANRKLKEMSIFDEELNQRHWKENTRSHSIEVAAYALLTQLSLNDLTYSLPIVNWLNTQRLVGGSF PSTQDTVIALQALSEYSIQSQEPDLSLVCNITSSNDRNLAKTLEFQKDNALTLQQFQVSKIGGTLFFRTAGRGIGSLSVQLRYNVPTPVE KLCKFEININVSEAKEKPKPPKINQNIDLDVFQNFDNAADEIGVNKNDLDVNAFNKFKVQLELKGKVVRRRRNAEDDDYGDYEDRGR VGEIAATDEVERSNSKVMLEVRICTRYLSDKDSEMTIVDAGIFSGFRPVKEDLDNLVNSTDIPVDRYEIAGRGVIFYLDRVSHKNPVCLK FRIRRDYIVGNVQASVIKIYDYYNNDDSCTKFYSPDSNSPLLRTICEGSVCQCAEGGCPLQKPFFEVSEMISISKQRQRLLLMACDEHDY VWIGKIKNLREENGFLYIDFLVSAVLKAGIEPKEDLEGIHRELMMRDACKKSHLFLDQEYLIMGRDGARYENKFTKELSYRYLVDKTTKL QRYVTLIKDAEDKNLQKAMIWLKKELLKNGCRL	197075.00
>TRINITY_DN14206_c3_g2::TRINITY_DN14206_c3_g2_i4::g.60787::m.60787 XP_023214856.1 polyubiquitin-B *[Centruroides sculpturatus]*	>Tcham_55 MQIFVKTLTGKTITLEVEPSDTIENVKAKIQDKEGIPPDQQRLIFAGKQLEDGRTLSDYNIQKESTLHLVLRLRGGMQIFVKTLTGQPRL KPSRTKDLTTLLTNYAPIKLVKSNHVTHHAICVLCNLN	14418.84
>TRINITY_DN13901_c0_g1::TRINITY_DN13901_c	>Tcham_56	3988

Sequence identifier	Amino acid sequence[a]	M M*
**Alfa-amylase**		
0_g1_i1::g.69719::m.697 19 Cbi_TRINITY_DN1030_c3 _g1_i1.p1 GBM11417.1Nucleobindi n-2	APVDPKKQAPPDPFEDLSIEYKNYMREVAKILETDEDFLNEIRNLANSSTYQPEDISFALQFAKHNVRTKLDELKRIELDRLRTYAVRQ MELQDPAQRKPFQIPTHLDLKNPHTFEAEDLKKLLKTAQKDMEEIARKRKEEFKTYEMEKEHAYRENLKKLNETERKEEEKKHEELIKK HKDHPKVHEPGSKSQLEEVWEEEDHLPADEFNPETFFSMHDLNGDQHLDEDEIQAFLIHEVQKMYNNDNEEDDPVERREELARMS EYIFKEADKNEDYLISKEEFLKMTKQKRFEQDEGWKGLDEELPYTEEELAEYEKMLEKKKKTISENKI	9.5 6
>TRINITY_DN1609_c0_g 1::TRINITY_DN1609_c0_ g1_i1::g.77623::m.77623 XP_023218885.1 nucleoside diphosphate kinase-like [*Centruroides sculpturatus*]	>Tcham_57 AHRERTFIMVKPDGVQRGLIGQIIQRFEQKGFKLVAMKFMQADDALLKKHYADLAGRPFFNGLVKYMQMGPVLPMVWEGFNVV KTGRDMIGATNPIESNPGTIRGDLCIQVGRNIIHGSDSVPSAEKEIGLWFTDKELNNWQPAMDSWIYEEN	17 35 5.0 4
>TRINITY_DN13274_c0_ g1::TRINITY_DN13274_c 0_g1_i2::g.18792::m.187 92 XP_067125559.1 14-3-3 protein zeta isoform X1 [*Centruroides vittatus*]	>Tcham_58 MDKDELVQRAKLAEQAERYDDMASAMKQVTETGVELSNEERNLLSVAYKNVVGARRSSWRVISSIEQKTEGSERKQQMAREYREK VEKELREICYDVLGLLDKYLIPKASNAESKVFYLKMKGDYYRYLAEVATGDQRSSVVEESQKAYQEAFDISKSKMQPTHPIRLGLALNFS VFYYEILNSPDKACQLAKQAFDDAIAELDTLNEDSYKDSTLIMQLLRDNLTLWTSDAQGDGDEQQEGGDN	28 02 0.3 2
>TRINITY_DN14051_c0_ g1::TRINITY_DN14051_c 0_g1_i1::g.13016::m.130 16 XP_023228896.1 14-3-3 protein epsilon-like [*Centruroides sculpturatus*]	>Tcham_59 MTEREDNVYKAKLAEQAERYDEMVEAMKKVASLDLELTVEERNLLSVAYKNVIGARRASWRIISSIEQKEENKGSENRLEMIRQYRL QVEGELREICQDILDVLEKHLVPTATTGESKVFYYKMKGDYHRYLAEFATGNDRKEAADNSLAAYKAASDIAMTELPPTHPIRLGLAL NFSVFYYEILNSPDRACRLAKAAFDDAIAELDTLSEESYKDSTLIMQLLRDNLTLWTSDMQHDGEGEQREQGQEVEDQDVS	29 34 7.8 8
>TRINITY_DN14510_c0_ g2::TRINITY_DN14510_c 0_g2_i1::g.47682::m.476 82 XP_067138268.1 carboxypeptidase E-like isoform X1 [*Centruroides vittatus*]	>Tcham_60 DDVPAGFVFKHHNNEELLQVLVDVHNKCPNITRLYELSHRSVNGQPLAVLEITKNPGKHEFLKPEFKYVGNMHGNEVLGREMLLALS SYLCNEYTEGNKDIQLLVNTTRIHILPSLNPDGWQIASTANGDNWLKGRGNANGVDLNRDFPDLNQIVYEHNDDTDDKFNNHLFSK DAVDHKFQPETLAAMKWILKNPFVLSANLHGGALVANFPYDESFHHSSQEYTATPDDDTFRHLASSYSKPHKTMAADKPIACNGDD FTHQGGITNGAAWYSVAGGMQDFNYLGSNDFEITIELGCNKYPPASELEKEWFNNKDALINFIWQSHIGVKGVVKDAETDQPLNG VNIKTVNITDDKHKLINHDVTTVTNGEYWRLLTPGKYEITALTPGYKAETETVTVSNPLHQEAIHLNFALEPESSENYDNMMEETTEEK RYYDNSEFARFLRFFHKKNRFNKM	51 92 3.7 7

Sequence identifier	Amino acid sequence[a]	MM*
**Alfa-amylase**		
>TRINITY_DN13938_c0_g2::TRINITY_DN13938_c0_g2_i1::g.69473::m.69473 XP_067141565.1 TOM1-like protein 2 isoform X1 [*Centruroides vittatus*]	>Tcham_61<br>MAAILAQLGGNPFNTPVGQKIEQATDASLASENWAVNMEICDIINETDEGPKDAIKAIRKRLQQNAGKNYTVVMYTLTVLETCVKNCGRRFHLLVTQKDFVQDLVKLIGPKNDPPTAVQEKVLSLIQSWADAFRAIAEMQGVVLVYNDLKQKGIEFPMTDLDAMAPIHTPQRSVPPSKPPKMQPVERVPPQVQPQIQPATENPTSPLNLLPDQLAKLRSELDVVQGNMKVFGEMLTELTPGQEHPSDMELLQDLQKTCHCMQSRIVELIDKVANEEVTNELLRVNDELNNLFLRYERFEKKRTTVNQSASSPIQNKQSPPQPEGKPLIDFGESEGTSADDLHKQLAGMDVGSASASVALSQINTVTATNKAPTQSNDDFDMFAQSRNVTYEQSKSSGSTYADNVDPDQVGVTLTSLTNARTQGVEGEENVSKELQKEQDFAEMERWLEDKPPAEESQESITSSEFDKFLAERTAAAEKLPNVEHTSTLSKSPTGSSSASSAANRNAPNNAANRRKMDREDTDNALFAL	58208.21
>TRINITY_DN10723_c0_g1::TRINITY_DN10723_c0_g1_i1::g.39196::m.39196 XP_067141684.1 cytosol aminopeptidase-like isoform X2 [*Centruroides vittatus*]	>Tcham_62<br>RRLVISTSRKKDRRIKMVKRGLILGAYEKGEKEEGFVLTAAAQKYDAEVQGRLSELLKISGPTLKKGKTRIFYGIDQQYCCVNVVGLGSSTAGYCEQEECDEKKENIRVAVSVGTKQLRECGAVEIDVDTCNDAEAAAEGGHLGLFTYDELKAKSSQKPIVNLRPFGNEGLDKWKIGMVKAEGQNIARRLMESPANVMTPTRFGEIAHSLLTPLGVQVIVRDKAWAESQKMGSYLSVAKGSDEPPKFVELSYDGAGPGVKHLALVGKGITFDSGGISIKPSLHMDLMRADMGGAACIIGSVYAVASLKIPINIVGLMPLCENLPSGHANKPGDVVTAMNGKTIQVDNTDAEGRLILADALCYAHTFNPQMILDLATLTGAMDVALGSGATGAFTNSTKMWEIIHDAGKYTGDRIWRMPLFHHFSKQVTECHLADLCNIGKHGRSGGCCTAAAFLWEFVTHPQWMHLDIAGVMENKDEYQYLCKGLAGRPTRTLVEFMSALTKHS	54731.05
>TRINITY_DN9159_c0_g1::TRINITY_DN9159_c0_g1_i2::g.22407::m.22407 XP_023214029.1 eosinophil peroxidase-like [*Centruroides sculpturatus*]	>Tcham_63<br>HGLPPYVDFVYYFHGFRIETFDDLVNRKLMDGSTVQRLKTVYSCVSCIDLFTGLCCEIYLQNAVVGPTAAILIAKQFERLKFGSRHFYTHQGYLTQNQKAFIQKMKLSYLFCYANRLPEITENVFLGRSPMLNCRNAPPLDLSAWKE	17019.76
>TRINITY_DN14116_c0_g3::TRINITY_DN14116_c0_g3_i1::g.73259::m.73259 XP_023237525.1 polyubiquitin-C [*Centruroides sculpturatus*]	>Tcham_64<br>FVKTLTGKTITLEVEPSDTIENVKAKIQDKEGIPPDQQRLIFAGKQLEDGRTLSDYNIQKESTLHLVLRLRGGMQIFVKTLTGKTITLEVEPSDTIENVKAKIQDKEGIPPDQQRLIFAGKQLEDGRTLSDYNIQKESTLHLVLRLRGGMQIFVKTLTGKTITLEVEPSDTIENVKAKIQDKEGIPPDQQRLIFAGKQLEDGRTLSDYNIQKESTLHLVLRLRGGMQIFVKTLTGKTITLEVEPSDTIENVKAKIQDKEGIPPDQQRLIFAGKQLEDGRTLSDYNIQKESTLHLVLRLRGGMQIFVKTLTGKTITLEVEPSDTIENVKAKIQDKEGIPPDQQRLIFAGKQLEDGRTLSDYNIQKESTLHLVLRLRGGDF	42641.95
>TRINITY_DN7218_c0_g1::TRINITY_DN7218_c0_g1_i1::g.48712::m.48712 XP_023217657.1 probable nucleoredoxin	>Tcham_65<br>MELITGTLVKKDGSEVSSSEALNDADVIGIYFSAHWCPPCRNFTPVLADAYEEMKDLNYKFEVVFVSSDRNEEALLDYMKECHGDWYALQFGSDKIKELKLKYDITGIPTLVVIRKDGTLITTNGRSDIHSKGAEAYTEWTK	16053.14

Sequence identifier	Amino acid sequence[a]	MM*
**Alfa-amylase**		
1 [*Centruroides sculpturatus*]		
>TRINITY_DN12663_c0_g1::TRINITY_DN12663_c0_g1_i4::g.43336::m.43336 XP_023219848.1 tumor protein D54-like isoform X2 [*Centruroides sculpturatus*]	>Tcham_66<br>MTKTSKLDLTDNYHQYLKIAADENDDKYSFNEPPEVKGYDATPDSGIHDLSSMYPEQRAKLEEELKNELAKTEDEITTLRQVLSDRVR<br>HAQELKRKLGITPWKEFKDDMEQGFRNIQESSAYKEVGTKLTEWNSAVVGAPIYQKTSSTLKTAGEKTTTAIGSISASVTKKLSEVKNS<br>TAFKSFEERVGSAYSNVKTKMAGSRSSSMNSFEEALSNAEGRSSNVASPATTPVIPEGKPLS	26433.39
>TRINITY_DN14094_c0_g1::TRINITY_DN14094_c0_g1_i1::g.16916::m.16916 XP_023224333.1 heat shock 70 kDa protein cognate 4 [*Centruroides sculpturatus*]	>Tcham_67<br>MAKTPAIGIDLGTTYSCVGVFQHGKVEIIANDQGNRTTPSYVAFTDTERLIGDAAKNQVAMNPSNTVFDAKRLIGRRFDDTTVQSD<br>MKHWPFQVINDNTKPKIQVDYKGETKTFFPEEISSMVLTKMKEIAEAYLGKTVTNAVVTVPAYFNDSQRQATKDSGTIAGLNVLRIIN<br>EPTAAAIAYGLDKKGSGERNVLIFDLGGGTFDVSCLTIEDGIFEVKSTAGDTHLGGEDFDNRMVNHFVQEFKRKHKKDLATNKRALR<br>RLRTACERAKRTLSSSTQASIEIDSLFEGIDFYSTITRARFEELNADLFRGTLEPVEKALRDAKMDKSQIHDIVLVGGSTRIPKIQKLLQDFF<br>NGKDLNKSINPDEAVAYGAAVQAAILNGDKSEQVQDLLLLDVTPLSLGIETAGGVMTVLIKRNTTIPTRQTQTFTTYSDNQPGVLIQV<br>YEGERAMTKDNNLLGKFELTGIPPAPRGVPQIEVTFDIDANGIMNVSAVDKSTGKENKITITNDKGRLSKEEIERMVKDAEKYKDDDD<br>KQKGRISAKNSLESYAFNIKSTVDDEKTKDKISEDDKKKLLDKVEETIKWLDTNQLAEKEEFEHKQKELEGVCNPIITKLYQAGGVPGG<br>MPGGFPGGAPGAGPTSGGGSGPTIEEVD	71017.19
>TRINITY_DN6070_c0_g1::TRINITY_DN6070_c0_g1_i1::g.77226::m.77226 XP_067124239.1 V-type proton ATPase subunit E [*Centruroides vittatus*]	>Tcham_68<br>MALTDSGVQKQINHMMAFIEREADEKAEEIDTKADEEFTLEKGKLVQEQRLKIIEFYDRKEKQVEMQKKIQSSNMMNQARLRVLKA<br>REQHVMTVLDEARRYLFDITKDQTKYQEIVRRLIIQALLQLMESEVTIRCRHQDMRLVQACVDSALKEYQNLTKQKCKVVIDKENFLP<br>SDICGGIDAYSQSGKIKVSNTLESRLEMISQQLLPEIRVALFGRNPNRKFSD	26474.59
>TRINITY_DN14436_c3_g3::TRINITY_DN14436_c3_g3_i1::g.15553::m.15553 XP_067135662.1 neural-cadherin-like isoform X4 [*Centruroides vittatus*]	>Tcham_69<br>QFFSLADNDFSPYFALLSNGILMATTSLVHLANTPITLVVREEYPNITTERMIILHVVERSKMLKFDREVYNGVVSENQPVGTVVEEVE<br>RIIASGDQNHKITYSLISVNDGERFGILQPQANDTGVILVTKSVLDREGQSTYELVLRATDVMAADSSEALIRIEILDVNDNSPVPDSKSY<br>RFKIPDDAELYSVVGNITASDADGDKPVYHFTTRHPVFAIVPKTGQILLISSPLLKTYKLSVRADDGRKPPRYSPVIPVFVEVFSNQYDFD<br>SDLDSTNEVLVRTRRSVRPTKSYDYKETDGSVTGRVMFQLDKKHPHEVYKIENPNHWVEVEPSGEVKVKEPWDYELLGKEKTIDFW<br>VFVTGPNVEEPERQRIIIHIKDVNDEPPYFINRPLPMQAVVQLNALPGTPVFKLQARDPDTDHNIHYFLVRDRTGGRFEVDERSGEVR<br>TLGSDPFMLDKEYVLYVKSEDHNGITSERGHYQTTGEKRLSIMGGKRPPQFYMSKYEATIPENQKKDSDIIEVKAKSFADREIRYTLRA<br>QGKGAGTFNIGPTTGVVKLAKELDYEDLRQPKSYSLIVTATEDSGGFSTSVELTIKVTDVNDNAPRFELPDYQAHNVDEDVPIGTSILQ<br>VSASDMDQGKNAEIEYSVDKDDFTIDNKGIIYSNKRLDADINNTYVLTVRATDRGDPPLTGTATIRIYTENKNDEPPKFSQDVYTPNV<br>DENAGPDTLVTTVVASDKDGDNIYFGFVGGGTVSGMFQIEERTGVIRLINGPIHLDKDKYELNVTARDDGSCCKNGAHTTHTSTALV	3297722.88

Sequence identifier	Amino acid sequence[a]	MM*
**Alfa-amylase**		
	VVFITDVNDNKPVFEECGTYAPQVEEGAPSGTSVIKVKATDQDKGHNGQVRYSIVQQPNQKGTKFMIDEVTGEIRTNKVFDREGDD<br>GRFVSVTVKATDRGNPPLEGVCSFKVEITDINDNPPLFDRQEYRENVKQDTPKGTNILRVSASDEDADNNGAIVYNLTAPYDPSDLEY<br>FEINPDSGWVSLKKALDRAQYHLRAVALDKGIPQHRATVEVIIDVVDRANNPPIWDQPVYGPIFIKENLEVGQRVISIKARSGIPDNPT<br>VFYTLMKGSTEQTNKKDTFYLSQRSENGDTWADLYVNYPLDYERIQQYNLTVRVENNGIHQLASEATVYIVLEDVNDEIPLFIEREQE<br>TVLEGMPPGTKVTQVQAVDKDGTYPNNKVYYSIESKDHGDKYFTIDRETGEIYTKIEFDREEKQAYAILVKAEDGAPSDRPHMRQGE<br>PNSVTKYIRIGIGDKNDNPPYFDQTRYEAEVNEDEDIQHTVITVTAKDKDESSRIRYEITQGNIGGAFAVKNETGAIYVAGPLDYETRKE<br>YNLTLVASDTLHENTTVVLIKIKDINDLPPKFQQPSYETTIFEEDSDGLPKKILKVTATDGDRDRRPDIVYFLTGQGVDDQDPANSKFAI<br>NTTTGEIYVLKTLDRDLPHGRSQWRFTVFAEDEGGNGLVGYADVLVNLKDINDNAPFFPYAIYTGNVTENGTAGMTVMTMTATDY<br>DDPNEGSNARLKYSIEQNQVNENGELIFTIDEETGVISTAVCCLDRETNPEYTIKVVAMDGGGLKGTGTATIKIKDINDMPPEFTKKE<br>WQVEVDETESDNIPETPILVVSVNDGDLLETNRFSYKVIDNAFGADKFTMVTNSDGTGSLKIAKPLDFEDLQQRFGFNITIQVSDHGG<br>ESTEPYHMDYAKVRVRLRDINDNKPEFEKPNIEFTVQEDAKVGTSLATFRATDADQGGKSKVSYTIDRSSDKKRQFKIDQNGVVKIQR<br>KLDREETPRHQVKILAIDDGTPPRTATATLTVVVSDINDNAPRFLRDYRPVIMEHSPPQKVEEILATDDDDRSKGNGPPFTFRLDSNA<br>PDIIRQFFDVQHDPTGANGDGMAIVSSRERFDREQQKEYLVPIVIRDSGTPSMTGTSTLTVIIGDTNDNRMHPGSKTIFVYNFKGESP<br>PTPIGRVHVEDLDDWDLPDKIFYWENHVVHPNFDLDKDTGMITMKNVTSGGTYFLKFTVHDRVYTHDVLANVSVIVKEIPEEAIYNS<br>GSIRISGISAEDFVRVWNWKENRQVKSKYEKFRDMIADLTNTSPNNVDIFSVILKQERPPITDVRFSAHGSPYYKASMLNGIVALNREL<br>IEQRVEINITMVGIDECLFENINCETSCTNVLMVDRQPIVVNANRTSFVGVNTWVHPKCACGARDFSEMETCRKRPSPCYNNGRCS<br>DTSSGVSCKCSSSYEGPQCQQTTRNFNGKGWAWFPSLQQCENSHLSLEFMTRSPNGLLFYNGPIANPDPGEISVQDFISLELKDGFP<br>RLLIDFGSGTAEVTVQHTSLSDGEWHQIDIFWERETVRIVIDNCKQAKLEDSDPPKIDRSLCEKIETIPPFNEFLNVNTPLQIGGVHHKL<br>LADYSWEHRHTRAGFDGCIKNFIHNSEMYDLGSPGSYSNSEQGCPPAEKQCQENGMKKFCEHGQCIGTYMEARCICHPGWYGTH<br>CDKETQSKMFQQNSYIKYALSFDLNPYETDIQLQFRTRQKHGELFRATSKHGREYCILEIRDKKIRLRFNLNHLRSSDERELWLPYVQIS<br>DGQWHTVRVLRFGSTASVSLDGGGGRRYNELLDYVGQHQLMQIEKQNVIAGGDVQYVGPGVTVVDNDFQEGCMNDIRLNQRYL<br>PMENGSENAAVVEWRNLIDGCPSNNPCQGIICPRPFHCEDLWMQHECRCQDGFVVTEDGKNCTDENECLSFPCMNGGNCVNLP<br>NGEGFYCVCLDGFGGDLCDARREEKVMRLSMAALAAILVCLLNILILVLVIVAYTRSRRADQKYGHGDVDDDVRENIISYDDEGGGED<br>DMNAYDITPLRIPINPAGTPLGTKPGPEKAPIKEPRQRQVPPGDTYPDVGDFISDHLDKADNDPNAPPFDDLRNYAYEGCGSTAGSL<br>SSLASGTDDNEQDFDYLNTWGPRFGKLADMYGQGESEED	
>TRINITY_DN12127_c0_g1::TRINITY_DN12127_c0_g1_i2::g.34125::m.34125 XP_067137896.1 V-type proton ATPase subunit C 1-B isoform X2 [*Centruroides vittatus*]	>Tcham_70<br>MFKLSRTEIGSKIWKIICAEKLEEMTEFWLISAPGEKTCQQTYETLNNLTSKHHNLSINFKFDVPDLKVGTLDQLVGLSDDLDKLDLFVE<br>GVTWKLANYLGDVLEDKKDKLTENLLINGASLSEYLTRFKWIEAKYPTKQPLPSITEIISKQISQIDADLKKKSSDYNNIKTDLMTFEKKST<br>GSLLVRSLGDLVKKNHFILGSEYLTTLLVVVPKALYKDWEANYETLTKNVVPRSSQLIYEDSEHGLYNITLFIKDVEEFKRVAREKRFSVR<br>EFTYSEEELAKGKNELAQLESDKKRQYSQLVRWLKINFSEAFTAWIHVKALRVFVESVLRYGLPVNFQAMLLKPQKKTMRKLREVLN<br>QHYSHLDTSIAQGPIDDIPGVNLGHQEYYPYVYFKININVLEGLSR	47071.18
>TRINITY_DN11678_c0_g1::TRINITY_DN11678_c	>Tcham_71	4941

Sequence identifier	Amino acid sequence[a]	MM*
**Alfa-amylase**		
0_g1_i1::g.3499::m.3499 XP_067142681.1 synaptotagmin-15-like [*Centruroides vittatus*]	MAVYKTFSEKSHTNNSQKVDGKYIWIIIGGCLSAAIGIILLIVLILYLRKRTFKAQSKESETEADLKRKRLSSVYSSSGDDADQSLSYQILARSFSTEAAIPYKMNSECVQPCHPTLTRWHSFNSTTYPPTFLGTVQPDLYRESDDEDTTIPTGMNGRLWFSLYYRSEAQELDVNLVKAKHLSGRGLNNTSRDPFVRIYLLPDEENYLQSKVRKRTLSPKFNEKFTFKVEESDLCNRTLRLSIYDVDKRKVRHTLGHVMIPLDKVDLSSNMLIWRDLESSALQTPTIRGEIQITLSCNPVNGRIKTTVCRVRNIQGIDSGEAGIYTRVLLYHGRKQVKSKRTSLQHFNPAAGDVIFDESFAFAVSGRFFDSCSFEFVVVVAGQSPLVKDEILGHVVIGPFMYARGEQLLHWQEMLTNQRSPVTKWHGLEAIFEP	5.44
>TRINITY_DN13386_c0_g1::TRINITY_DN13386_c0_g1_i1::g.51348::m.51348 XP_023212377.1 protein D3-like isoform X1 [*Centruroides sculpturatus*]	>Tcham_72<br>SLFSSASTTQRVFLPTGFLYRVSTEPGNLTLVNSMEENGVVPDVLDTAPRYIAEVKYGEEMANLGNELTPTQVKDPPTHISYPVETEALYTLCMTDPDAPSRQNPKYREWHHWLVVNIPGTDVSKGEVLSEYVGAGPPKGTGLHRYVYVIYKQPNTLQPDEKRLTNRSGNHRGNFKIRNFAQKYNLGAPHAGNFFQAQWDDYVPKLYEQLSD	23936.78
>TRINITY_DN14060_c0_g1::TRINITY_DN14060_c0_g1_i1::g.13071::m.13071 XP_023214020.1 ADP-ribosylation factor-like protein 8B-A [*Centruroides sculpturatus*]	>Tcham_73<br>MLALINRILDWFKSLFWKEEMELTLVGLQYSGKTTFVNVISSGQFCEDMIPTVGFNMRKVTKGNVTIKIWDIGGQPRFRSMWERYCRGVNAIVYMVDAADHEKIEASRNELHNLLDKPQLAGIPVLVLGNKRDLESALDEKDLIEQMNLQAIQDREICCYSISCKEKDNIDITLQWLISHSKSRAH	21436.80
>TRINITY_DN11972_c0_g1::TRINITY_DN11972_c0_g1_i1::g.54599::m.54599 XP_023220952.1 PI-PLC X domain-containing protein 1-like isoform X1 [*Centruroides sculpturatus*]	>Tcham_74<br>KSIDNLSLSATADTDCSKSEKEVQIYLSVSSLAWENDSVVTKRQLELNWVSKNPKPKDRVALYDSLPDGKVDPIEIVYVAEHPAGYYRTKHELPTFTFTKDNITYGCLNFYIGYISENKITHLKCLETRPRWMNNIRKYIDNFTLMELMIPGTHDSGSYSSFPYNDGFFTVITKFIYAQEETIFNQLVYGIRYFDLRVEKYNNKFIIKHGIFPTKTTLEQVLSDVRDFVKTTKEIVILDFHKHPTDCIEKELFSYLKDKLDPYIIPRGSKLTIGEILDSPYRIILSFNEAKITNSEIWPSVCQIWGNVLKVKKLKEYFDAFFNKTRVPPFLWSAMVELTPSPSFIVQHTDEGLRVLADSVNRNVTRWFRDMWGKHSNIVATDYFLGNDIISLAIDINKNRRCESRKPPAEDLSTFSCSPE	48747.74
>TRINITY_DN13449_c0_g1::TRINITY_DN13449_c0_g1_i1::g.23149::m.23149 XP_023221466.1 cathepsin L1-like	>Tcham_75<br>ADSVTFHDVILQEWEEFKLKHGKHYESSEDVFRMKVFMDNKHKIAKHNSLYERGIKKYKLAINEFGDKLHHEFVQTMNGFKRQYQSHYENGSTYLSPANVQIPKSVDWREEGYVTPIKNQGSCGSCWAFSTTGSLEGQHFRKTGVLVSLSEQNLVDCSKKYGNEGCNGGLMDQAFSYVKDNHGIDTEQSYPYEGMDGKCKFSKRNIGATDSGFVDIPEGSEKKLKEAVATVGPVSVAIDASHESFQFYTEGVYDEPDCSTEQLDHGVLVVGYGTNEMAEDYWIVKNSWGTTWGKSGYIFMSRNKKNQCGIASQASYPLV	35978.06

Sequence identifier	Amino acid sequence[a]	MM*
**Alfa-amylase**		
[*Centruroides sculpturatus*]		
>TRINITY_DN11889_c0_g1::TRINITY_DN11889_c0_g1_i2::g.25655::m.25655 XP_023223106.1 peroxiredoxin 1-like isoform X1 [*Centruroides sculpturatus*]	>Tcham_76<br>MTNLSTNLYLGPFGEGKEYKVMSAKLPLLTQSAPDFCGTAVIDGEFKEIKLSDYKGKYLVLFFYPMDFTFVCPTEIIAFSDRVDDFKKIN<br>CEIIGCSTDSHFTHLAWINTPRKEGGLGKMKIPLLADKSTQISKDYGVLKDDAGVSFRGLFIIDDKGKLRQITVNDMPVGRSVDETVRL<br>VQAFQHTDKYGEVCPAGWKPGSDTMVPDPVGSKEYFKKH	24372.06
>TRINITY_DN14458_c1_g2::TRINITY_DN14458_c1_g2_i1::g.17268::m.17268 XP_023230069.1 beta-mannosidase-like isoform X1 [*Centruroides sculpturatus*]	>Tcham_77<br>LKLNSVLKESNSIRISFTSPVEYSGNKSKQYIQKNKYVVPPICPPPVQNGECHVNFIRKVQSSFSWDWGPSFPTMGIWKNIGIEAYDTV<br>IIRDITVATIPDKSVGEVISLWKLNLTIYYENVAPINSTVAIKLNKLILMNKTLQLNEKGVLNITVNIPTNSIELWYPNGYGKQQLYNLTVIF<br>NPLSSSEVAIKTIRIGFRFIELIQEEIKGSSGLSFYFKINNIPIFAKGSNWIPADSFPERITEKYIRSLLQSVRDANMNMLRVWGGGMYEQ<br>DSFYEIADELGILIWQDLMFAVALYPVNKEFLDSVSVEVKQQIRRLHHHPSIVLWAGNNENEAALAQKWWPAVYLSYKRYKKDYITL<br>YINTIAPIVTEDYTRPFLSSSPSNGKITKQDGWISSNPQDNHYGDVHFYDYTSDSWDWKIYPRPRFASEYGFQSYPSFVTIANISLPED<br>WTYPFGKFLSHRQHHLFGGLEILKSIRNHLFLPKIKCGPQAFKDLLYLSQITQAMSIKTETEFYRRSQSDIINGEGKTMGALYWQLNDI<br>WQAPTWSSIEFGGRWKMLHYFAVNFFNDFLVSPYINGTNITVSFISDKTQKFNNLSLYLNVYRWSSFNAIYKRNLTFNQLPQSSQIIF<br>TVLQTELLKEAGCNSAKTCFLSFFVKNKDGQQLGPSNYLFLSSFNEANGLNKANVSIESIGIINNTIAENYEFKIILNSDNIAPFVWLETTI<br>EGRFSTNGFLLISSKFDVIFFS	85114.25
>TRINITY_DN12949_c0_g1::TRINITY_DN12949_c0_g1_i1::g.29979::m.29979 XP_023232280.1 triosephosphate isomerase-like [*Centruroides sculpturatus*]	>Tcham_78<br>MSQRKFFVGGNWKMNGNKAGIEEIVGFMSKGPLDPNTEVVLGCPSIYLDFTRSKMPANIGIAAQNCYKVEKGAFTAEISPAMIKDV<br>GVNWVILGHSERRNVFGETDELIADKVAHALSSGLKVIACIGELLEEREGGKTTEVVNRQTKAIANKVSDWNNVVLAYEPVWAIGTG<br>KTASPEQAQEVHAQLRQWLKDNISEDVANKTRIIYGGSVTAANCKELAKKPDVDGFLVGGASLKPEFVQIVNARQ	26947.82
>TRINITY_DN14449_c0_g2::TRINITY_DN14449_c0_g2_i1::g.16788::m.16788 XP_023232959.1 aldehyde dehydrogenase, mitochondrial-like	>Tcham_79<br>MAPLKNPQIKYTQIFINNEWRNSVSGKVFPTVNPSTGEKICDVQEGDKADIDLAVKAAKEAFKLNSPWRTLDASKRGVLLNKFADLL<br>ERDIEYLASLETLDNGKPFKLAYGDIIFSINVIRYYAGCSDKVHGKTIPADGDVVSFTRLEPMGVCGCIIPWNYPVLLFCQKVGPALAGG<br>NTVVIKPAEQTPLTALYCTSLLKEAGIPSGVVNVVPGYGPTAGAALCYHTDVDKIAFTGSTEVGHLIQQAAGASNIKRVTLEMGGKSP<br>LVIFADADLDEAVRLAHEMVFVNQGQCCCAATRTFVQEEIYDEFVKRSTELALKRSVGDPFDDKSAQGPQIDDIQFNKILDLIKSGQK<br>EGAKLNCGGERLGSKGYFIKPTVFSNVSDNMRIAKEEIFGPVQQLLKFKTLEEAIVRANDTNYGLAAGVLTKNIDTAMTFAQAVQAG<br>SVWVNCYLHATPQTPFGGFKMSGIGREMGFDSMMEYLETKTVTIKIPKKNS	53443.42

Sequence identifier	Amino acid sequence[a]	MM*
**Alfa-amylase**		
[*Centruroides sculpturatus*]		
>TRINITY_DN12330_c0_g1::TRINITY_DN12330_c0_g1_i1::g.44053::m.44053 XP_023234091.1 epididymal secretory protein E1-like [*Centruroides sculpturatus*]	>Tcham_80<br>TEYKSCSKEYGTVQSVTVTNCDSSPCILKVGSTPEITINFNSLVDSKKVEAKIYGVIGSVPLPFPNPHTDACENSGLTCPLEKAKAYTYKN<br>SFDIKSQYPRIPVTVKYTLVGDNDEQVVCVEIPSKIQD	14129.03
>TRINITY_DN14006_c0_g1::TRINITY_DN14006_c0_g1_i3::g.15378::m.15378 XP_067126796.1 ninjurin-1-like isoform X2 [*Centruroides vittatus*]	>Tcham_81<br>MAAVGPTGAVKPRALDANAYATKKSVAQGMLDVALLTANASQLKYLLRMGNRLDFYYPMMVLVCMSIALQVSLSSLPSVGPSRRS<br>SFRSNLTIGQPVFTSDDRDVFS	11519.41
>TRINITY_DN9643_c0_g1::TRINITY_DN9643_c0_g1_i2::g.2613::m.2613 XP_023239632.1 cofilin/actin-depolymerizing factor homolog [*Centruroides sculpturatus*]	>Tcham_82<br>MSSGVTVSTEAKTVYEEVKKDKKYRFIIYHIKDEKVIDVESTGPRNATYSDFLDTMQQFKTECRYCVFDFPAPMPVEGSHEKTSMSVD<br>RLVLMTWCPEGSKIKQKMLYSSSYDALKKALVGVYKYVQACDFEEISQEAIEDAFRKGGK	16976.42
>TRINITY_DN13513_c0_g1::TRINITY_DN13513_c0_g1_i1::g.57756::m.57756 XP_067121295.1 cathepsin L-like [*Centruroides vittatus*]	>Tcham_83<br>VGEWEKVPTSDNVRILALFGAKHINLGINSIFHHKLIRVLSAERQVVAGYNYKINITIQETECKKSEHSFEAAQKCNLLSSKSPKVCQVV<br>VYQRPWLNETKVTSFACSAASSNAKRSNVKSLHRRLFEKFIKKYKRSYSDEYEKEKRFKIFRANLKQIKELNSNERGTAIYGVTKFADLN<br>ENEFRKHLGFRPDLAQANSIPPPAEIPSISIPKSFDWRHYNVVTEVKNQQQCGSCWAFSTTGNIEGQWALKKKKLVSLSEQELVDCD<br>KVDQGCNGGLPSNAYKEIIRLGGLETEKEYPYEAEDETCEFKKSDVRVYINSSVSISQNETEMAVWLVKHGPISIGINANAMQFYYGGI<br>SHPWKFLCSPDNLDHGVLIVGYGVHSYPLFKTVLPFWIIKNSWGADWGEQGYYRVYRGDGTCGLNKMATSSVVD	48843.55
>TRINITY_DN13651_c0_g1::TRINITY_DN13651_c0_g1_i2::g.28126::m.28126 XP_067123697.1	>Tcham_84<br>TDEFESENSDSNFILPHTEYHYKTTVEHLDQKFMVGWYKMVKGFISAIHSKSAETVDVLMKYFSHKKFVVPESIYELLKLQYAVIACIVI<br>GLLYVILMWVTGLIVFCCRCCCDNCGGKRVHDQTRKTTLWRKILILFLLIFVILLLIAVICMFVTHNSLSTNYSTAEKKTVIMFDDVQSMI<br>LNTENQMKYIASNSVNETLINIFLKLNDIESFLTSEITEKMEPLQKSMNFANSVLSGTLNFTQNLQGLSNAGDILLNNSEEITNGLQNISL	96210.87

Sequence identifier	Amino acid sequence[a]	M M*
**Alfa-amylase**		
prominin-1-like [*Centruroides vittatus*]	QLDKIKQKCPKDECEEIDPSQFNITGLNFTIEFIGNAANSLKNLNPKDYEESLKNVNEIIKKLPKQVNEQTKPLQNDMKTSLNKVSEEFQ KSATKLFDDIFPETKKQLSKIYETTKQNIHKGKKYDNYGKYTTLGICIIMTVIVSFLVIGLFFGICGYQRNRPPTKRTTMSNVGGHLLLFSV TWMFIFSGIFMLFTIILFVFGSSVDTYICRFFYDEKLIMANKILENFLNNSRTTVKVPYVGEIEPGDILNKCDSNETLFTIFDLENRLNLTEK LDFEKRMNFEEKIKELKNNIKFPKIKILSEKAETFMKNTIDSLKLEDIQNGIVQFNKSENLNFEEQKTMLTNIANSKEELKTEITEILIDIKTI EKSLEKFDEQKMNLLGSLTQIINAGKNYSGIIQQIYQKMKFEDEHLKENLTRNTIEIAKKFVSSLKTHTDKLIKFILQSFKKSIAPCKPIWNI FASARSLVCEYYIQVLNGFWFALGWCLFCFIPTIIMAFKLSKHFLRMSKLGKPDEYKKEKPEYELELIDEESSSRSWVTQRNKIRPDNG WHPPVQHM	
>TRINITY_DN14086_c3_g1::TRINITY_DN14086_c3_g1_i1::g.13507::m.13507 XP_067124801.1 plastin-1 isoform X1 [*Centruroides vittatus*]	>Tcham_85 MSSVIRRSLLLSEEQREELADNFNTLDKDGNGFLDMSELKQALEIVGFKIPQWKVRQMIDDLERGQVGDKKGRLSFEEFQQVCIDIKS RDVANTFKSMVTKRENVETLGGMSEASSEGTTHSVRHEEQVAFSDWINSNLKNDPDLKHLLPIDIEGKLLYDKVKDGILLCKIINHSCP DTIDERAINKKNLTVYTKHENLTLALNSSQAIGCNIINIDAHDLLKGKPHLVLGLIWQIIRIGLFNQITLEHCPGLVQLVKPGEEMSDLMK LSPESILLRWVNYHLDRAGTNRRITNFTNDIKDSEIYTHLLKQIAPVGAGVTCEALMQRDLLSRAETMLQQAEKIGCRSFLTPNDVVEG VYKLNLAFVANLFNNHPGLDQPDEPIEFINLEETREEKTYRNWMNSMGVSPYVNWLYSDLADGLIIFQLYDIIKPGIVEWNKVHKSFN RLKAFMEKLENCNYTVELGKQLKFSLVGIAGQDISEGNPTLTLALVWQLMRAYTLNILTQLANTGHPLVEREIVDWVNNKIKSAGKSS FIRSFQDSTIATAHPVIDLIDAIKPDSINYSQVKPGDNEEERMDNAKYAISMARKAGARIYALPEDITEVKPKMVMTVFACLMARDYL PNMGEKNGTSSSPVRE	72 08 4.6 4
>TRINITY_DN11809_c0_g1::TRINITY_DN11809_c0_g1_i1::g.25398::m.25398 XP_067131751.1 degenerin unc-8-like [*Centruroides vittatus*]	>Tcham_86 MCENAQFVMDASPRRKAFKFTVFILCTVGFVYQFYEFILFYLTYPTTLESFIKYTSPENLPKPVITVCDQNNIRRTDFCKSFPKLCYKAKNI DDFCKFYQYSCVNRKIIKEEDMMIPRTSNITNSYYNRTILEKYGMKVEDVLKLYRNNTGEIVLTNENRELVNCYTLDLAKFNKIRFKKRR ITSVKILGSNKIGNILINFNLKDLIHPYEPGIKISVHSKDKLANPFIDGFLVKPGRKYIVQLKPKEHHLLKDPFNTNCTTYNVTNDVPYNET YNQERCIIECKKLNWLKQCGCLSIQYPFRYDGKYCNADDINCLIQVNHENCYHNYRTSCKFIEFSYEVSNSPNYHINYDIPEELSSDPYFS RNKSLSEMSFIYKSPGLLISEHKPRLNLIEIFSFAGGYIGIWLGISLYSVIDFFEWLTIYIYYKRKKERACIINSEKLINVLKVNNIRRY	53 87 0.3 5
>TRINITY_DN13684_c1_g1::TRINITY_DN13684_c1_g1_i2::g.27625::m.27625 XP_067132021.1 V-type proton ATPase subunit B-like [*Centruroides vittatus*]	>Tcham_87 HLKMSLKNKQKKMREVQIGHKEHVMAVKRDYISQPRLTYKTVTGVNGPLVILDSVKFPKYAEIVHLVLADGTARTGQVLEVAGSKAV VQVFEGTSGIDAKHTLCEFTGDILRTPVSEDMLGRVFNGSGKPIDKGPPVLAEDFLDIQGQPINPWSRIYPEEMIQTGISAIDVMNSIA RGQKIPIFSAAGLPHNEIAAQICRQGGLVKIPEKSVLDSHQDNFAIVFAAMGVNMETARFFKQDFEENGSMENVCLFLNLANDPTIE RIITPRLALTTAEYLAYQCEKHVLVILTDMSSYAEALREVSAAREEVPGRRGFPGYMYTDLATIYERAGRVEGRNGSITQIPILTMPNDD ITHPIPDLTGYITEGQIYVDRQLHNRQIYPPINVLPSLSRLMKSAIGEGMTRKDHPDVSNQLYACYAIGKDVQAMKAVVGEEALSSEDL LYLDFLTKFEKNFIAQGHYENRSVFESLDLGWQLLRIFPKEMLKRIPQHILTEYYPREGRKSRN	56 74 1.2 0
>TRINITY_DN13684_c1_g1::TRINITY_DN13684_c1_g1_i1::g.27626::m.27626 XP_067132021.1 V-type proton ATPase	>Tcham_88 HLKMSLKNKQKKMREVQIGHKEHVMAVKRDYISQPRLTYKTVTGVNGPLVILDSVKFPKYAEIVHLVLADGTARTGQVLEVAGSKAV VQVFEGTSGIDAKHTLCEFTGDILRTPVSEDMLGRVFNGSGKPIDKGPPVLAEDFLDIQGQPINPWSRIYPEEMIQTGISAIDVMNSIA RGQKIPIFSAAGLPHNEIAAQICRQGGLVKIPEKSVLDSHQDNFAIVFAAMGVNMETARFFKQDFEENGSMENVCLFLNLANDPTIE RIITPRLALTTAEYLAYQCEKHVLVILTDMSSYAEALREVRHFYS	34 18 5.4 5

Sequence identifier	Amino acid sequence[a]	M M*
**Alfa-amylase**		
subunit B-like [*Centruroides vittatus*]		
>TRINITY_DN14038_c0_g4::TRINITY_DN14038_c0_g4_i1::g.12987::m.12987 XP_067136737.1 furin-like protease 2 [*Centruroides vittatus*]	>Tcham_89<br>AERLYINEFAVKIRGGESIANEIAQKHGFVNRGQVGNLKGYYLFENHKIVKRSLDYNHHHHGILKKEPSVLWVEQQHSKQRVKRDVF<br>QMHFTGMKDPLYRDQWYLVSGGEGGYDMNVLPVWKQGISGKGVVVTILDDGIQTNHPDLVQNYDGLASADINGRDDDPMPQ<br>DNGDNKHGTRCAGEVAATAFNDFCGVGIAYNASIGGVRMLDGTVTDEVEATALSLNPNHIDIYSASWGPEDDGKTVDGPGKLAKQ<br>AFLDGINSGRNGLGSIFVWASGNGGRFSDNCNCDGYTNSIYTLSISSATQGGLKPWYLEECSSTLATTYSSGTPGRDRSVVTVDMDT<br>TYFKELRNGRKPNPFTLCTRSHTGTSASAPIAAAICALALEANRFLSWRDMQHLVVLTSRPEPLLKETGWQTNGVGKKVSHKFGYGL<br>MDAAAMVKLAKQWTPVGKQHICNTPVDNTERIIPNSPGKELKVSLFTKACAGRSNVVHYLEHVQVLISLYYKPRGNLHIRLISPSGTS<br>SSVLLPRPRDVDDGSLNNWPLLSVHFWGEPAEGKWTLIINTESHRSHSSSPGKLQRWSLILYGTETPSPIHLLRNNHPYRYNPLIRETS<br>ENSKNPCRTDDDKNEKKLFDPKSGKCVEECPDNKFENKTHNICSSCNFPCFNCFGPNSDNCLSCIKSYYLQEDFCVQECSDGYYKDDE<br>LNECLPCMSQCMLCENNPSFCLSCKTGFYLFENHCVSECPEHMYHGSDHRCLPCHSNCETCKGLQENECTGCAIGNFLHLGNCIEKC<br>PDHYFEDVEHKECVKCDIGCKTCDQEKCYSCDKDWKLQNGYCIFEKACKSGESCVRGYKCNENCKECNGPRKNDCTVCDFSSHYLF<br>NGQCVNECPDGYFANQYHHCQLCHVTCSACIGHEMGQCQACIQEYVLHGGNCLPDCPKGFYKSDTNHCEPCDKSCLTCLSGTNKN<br>CTTCESSKYLINGMCHDESECPESYYLRELPDGNECLPCHHSCKSCSGPGPTSCLSCSDNATFRKGVCILCLNDEYFDQDTKFCEHCHP<br>SCGKCFGPAEDQCLSCLAPLKFDAIHHRCLPCCRFDITNSLDCCTCNENSDVCVNSDNYPRSVINEGKDKDKLQLSGDNSMSSNVAFT<br>ILIVCSLAVMLFLVIFVILQASSLGWCRRFVSNHSYKRVPEEITNSYDNKMEKVLLTQHDQDDEEDDIYYKM	13 38 35. 58
>TRINITY_DN14637_c1_g1::TRINITY_DN14637_c1_g1_i1::g.59291::m.59291 XP_067139281.1 D-3-phosphoglycerate dehydrogenase-like [*Centruroides vittatus*]	>Tcham_90<br>MSFCLENVLIAEPVDPSCAKILEDNGVKTQTKIGLSRDQLIAELKNYDGLVVRSATKVTAEVIEAADRLKVVGRAGTGVDNIDCDAATR<br>NGILVINAPGGNAISAAELTCAMVITLSRHLPQAYSSLKNGKWDRKSFMGQELLNKTLAIIGLGRIGREVAHRMQAFGMKTVGFDPL<br>VPPEESIKFGVKSYDLNDLWAMADYITVHTPLIPQTKNLINDNVLGKCKKGVRIINCARGGIVDESALLRALESGQCGGAGLDVYIEEP<br>PKNTALIQHPKVICSPHLGASTVEAQNRVAVEIAEQFVDLLHGKDIMGVVNSTSFCQLLSPVNKSWAELSLSLGKISSALIPSGSGDIKV<br>TVTTQGMQLEKKVTFLSIAIIVGLLQQRIKLGVNYINAPILAKEAGIEIEKKHEDVCLKRNIDSAVVINISAGEKKHKVVGLIAGNKAFLSSI<br>DGADFFPHVELKGNLLLCRSNDSSATFSKLIGVFNPKDIQAVWCSSVINNSIWYVFNVISDNLNEKAISSLVEFYAFVTL	56 93 2.9 9
>TRINITY_DN9159_c0_g3::TRINITY_DN9159_c0_g3_i1::g.22413::m.22413 XP_067141033.1 peroxidase-like [*Centruroides vittatus*]	>Tcham_91<br>IHRTANLGSLSNIYSALVSAFGQFIDHDMEFTTQPTTENNAPIQCCGNPSPNENCLSIQIPRDDPDLPGITCMNFVRDARCTVCELGY<br>REQMNSLTTALDCSQIYGTEVDLARELRRLDGSGKLKSRTTSVGEFPPADPNITNQCNPPGQCFRTGDFRSNQNLPLATFHVATLRL<br>HNVFADEMKRINQQWNEERLFQETRKLVICIYQYIIYSEFLPLIIGPDLIQLSRANADNTRFDSSVPLKLINSFAAAGYRLHSIVPDPIFTIT<br>EPGVKGEIPLRDSFFNMTYILQNGIENILRGGTRQQEENYDRLLVQSLTTFMYRRNTSSLGLDLASINIQRGRDHGLPPYVKFVKYFH	40 32 7.6 9
>TRINITY_DN14686_c1_g1::TRINITY_DN14686_c1_g1_i8::g.59438::m.59438 XP_067141325.1	>Tcham_92<br>GSSSSASPTVSTRGSSSSASPTVSTTQFSSSASPIVSITGSSTFASPTVSTTVSSSSASPTVSTTGFSFSASPTVSTTFTPTSTTFSPTQSTGF<br>SYSASPTVSTTLTPKLTTFKSPTLSTESSTVPESHRLSYRLQDPRHLHLNISKIECKTNEWLSDEIILDINCSKRYICLPVPFEEIKISVKGQD<br>NDLQYWEIHLKHFRDIAFIEIVDSKDLVLVLVYSLDGSESNMTNQLLYFNEFEKVKFKETIRVKSFAFVPLAINESDISQVIMKISVCKDKE	13 56 99. 32

Sequence identifier	Amino acid sequence[a]	MM*
**Alfa-amylase**		
hemocytin-like [*Centruroides vittatus*]	CENLNETYCPENCEKNNLECNGTACVTSGSCPCVVNSIVYPPGIYIDECRNRTCENSKFTETYISCEDRGKNICKEKQTLFEDRDTCDCDCRCNNISVNCNTSELCIEREKICDGIKDCINGTDEKCCPVNVTELSNCKRTNGECGRSNATCKFRENTVIMFSKVVYTCLEVCEAILLQERGKKTFRISYHRVYQNEERFTHHVIIVVDNYSYNLPQKELVIVIKDDVETVVSLSASSSYYNEQGGVSVQVIGDAIQFRSEQKGFTVIWRERNIVDIELDACEIDRVEGLCGYFATRAIHDEYRGKDGTCFNKDAEMCSDWTFDKSNDVCGSIYEMENEKLLTAERVCDEISKKVYYTCNVDVTEQTSDNSFRANDKVCKYVIYPNCTTDNRLCQCEALKYLLVEASCKNGIADLLLEYNCRELCPEGFQWDMCDADNTCEDLTFNGNSKKKIAGCFCPKHLVRKGDKCIPSHLCENCVCDIYGDPNYQTFNKRNYRFLSTCPGPYVLVQRKLPSNEPFFKVLSVNEKCIEYVNGTCISGIHVYYNNTKIEIYRNTTVLKNDRLYTSYCEDTSCNFNGIDFKIETETNIYIVKLVEIHVVIEYKIGNATLHIAGEVSIKFSSYTFFNNTEGLCGSCNGDSSDEFRLRNGSVVDDISTFALDWLEEKNKSLCEIYKTDNCTNIQNVSKCEFPQHLNCSIVDNNRYYSRHSGSCKNDLRCNDQITDSDICKYKFLYADNCCAFRLPMDKWLEIENCVIPCKENRTFRCVDICETKCPAERNEQCEFGKQMRMVCACPDGYVERNDKCIKNTECDACSPKPTLNIPARYFNIDNCTNLDPIVNMYECNGKCNDDFSHYTIERPYNFKEPIDYIYKKKCHCCQPSKYINDIITLTCINGLKKNIIIKKPIDCTCIECNATVI	
>TRINITY_DN14206_c3_g2::TRINITY_DN14206_c3_g2_i3::g.60784::m.60784 XP_023214856.1 polyubiquitin-B *[Centruroides sculpturatus]*	>Tcham_93<br>MQIFVKTLTGKTITLEVEPSDTIENVKAKIQDKEGIPPDQQRLIFAGKQLEDGRTLSDYNIQKESTLHLVLRLRGGMQIFVKTLTGKTITLEVEPSDTIENVKAKIQDKEA	12466.37
>TRINITY_DN12059_c0_g1::TRINITY_DN12059_c0_g1_i1::g.39709::m.39709 XP_067143319.1 leukocyte elastase inhibitor-like *Centruroides vittatus*	>Tcham_94<br>ECSTEDNDSLDPNWDSILKTFELGNMEFGLELFRNLNSELNNDTANLFYSPLSIWSALSSLYIGAKGQTAKELETVLGLDFTKKFFLPKLFDKFLELCEQCGDNENSAFRMANRIYMDKNIELKLCEDIIKNIVRRTDFSENPEESSKEINEWVEEKTNGKIRNLIPASAVNSATQMIVVNAIYFKEHWKTQFNPEMTRSTRFYMNRDTIYRVDMMNTHGTFIYGVSEDMKCQALEIPYSGDELSMLILLPQHPFNGFSHLAKTITGNRLKNLINSMSHRELWITIPKFKVEEEFELSNILQKMGLRNAFNPVFADFSGFTGDKTLSVDAVYHKSYIKVNEKGTEAAVATSVLLSRVARPSGITRFVADKPFLYIIRHVQSNVILFMGTVKSPNYQ	45425.84
>TRINITY_DN12708_c0_g1::TRINITY_DN12708_c0_g1_i1::g.77932::m.77932 XP_067144376.1 neuroendocrine convertase 1-like [*Centruroides vittatus*]	>Tcham_95<br>RHFTNDWAVQLNGGDDMAEQLANEMGYYNLGQIQGFPEVYLLRKKDHPWRNKREASNLNKRLKRDPRVLWAEQQEIRKRDKRDLFSVDGNYHIHGNRFNDQLWPKQWYLHDVRKDNDTQRDMRVIPVWNMGFTGKGIVLSVMDDGLEWNHTDIVRNYDSKASWDSNDNDPDPFPRYDMTNSNRHGTRCAGVIAMEANNYKCGVGIAYNSRIGGIRMLDGPITDVTEGLSLAFNIDYIDIFSASWGPTDNGETLDGPKTLAYKGLEKGVTKGRNGKGVIYIWAAGNGGSIDNCNCDGYTSSIYTLSIGSVSQSGKFPWYAEKCASTMATTYSGGSSERRISTTELYNNCTTHHSGTSASAPIAAAIVALVLEANSNMTWRDIQHLTVWTADVSPLLNNKGWKINSAGLMYNSKFGFGLMNAETMVKSALKWENVPEKTLCWIDARQSFPASLSSNKSAEINFLANGCGVNYLEHVQVRLTIDYTVRGVLDLYLISPSGTATVLLTRRIKDKSMEGFQNWDFLSVHLWGENPSGLWKLVIRDRSGKNYNGLIRVASLHLHGTKDIPKHMLNGKKDYRNQQRALSAIDHNPEYEETTAEKSDRLVFSQLEYFYKQKYYL	69788.39

Sequence identifier	Amino acid sequence[a]	MM*
**Alfa-amylase**		
>TRINITY_DN14275_c2_g1::TRINITY_DN14275_c2_g1_i9::g.62241::m.62241 XP_067127317.1 nicotinamide N-methyltransferase-like *[Centruroides vittatus]*	>Tcham_96<br>MEAVCEDKYAFSKFYEDVNFTLNYRDTITNNMKNEEEFVSYCLHKIFQDKAFQGKRLIDIGSGPTVHKLAAASAYFSEIILSDYSQHNR<br>KEIQKWLKNESSAINWDSLFQQEAILEGYRDVEEGINEIKLRLRQKIKNVIHCDVLQKEILPINIGKFDILFTSYCLEAACISLESFDSVIKNL<br>SNYILPNGGLVMATVLGCPQYQIEGYDKTISSLSIDPEFVNNTIKEAGFIIKHSYLMENHEENPCGKFNGILVLSATKSI	30041.27
>TRINITY_DN14258_c0_g1::TRINITY_DN14258_c0_g1_i9::g.61574::m.61574 XP_067140417.1 polyunsaturated fatty acid 5-lipoxygenase-like isoform X1 *[Centruroides vittatus]*	>Tcham_97<br>MGNLIPNLKSDYIIEIKSSDRSDAEPGTIIWLILHDERGLKTNAIRLNISVKNEKEMYTFQVTCPLNFDNVDKIEIWRNCYGVGDVFYLEY<br>LTVEKKNGKKLWFPLHRWISSQHRYYFRVYDCCLPQHELSLTQRNDELNDKRKLYEYTEHIEGGPLQIKILPEEEDFTTYLFQWELIHN<br>CKKEKLSIVVEMTSGKWQTFDEIKSFLRKNYTEPSCVNRWNDDSWFAMQRLQGINPVSIKLCRELPENFAVDGGELEAFLEGLSVQE<br>ALENKKIFIVDHKIMKNLPCKDDKLVCAPIAVFYLNSNGEILPIAIQLFQDRTVDNPVFFPSDPPYTWLLAKMYFNNADALYHRACLCV<br>GRTHLLMESLWISINRNLSPSHPLYKLMAPHFKYIFSINKLMLEKFLVMGGWIDRVTTIGVRGLCHLIARRFPNWKFEEINIPKEIESRG<br>VNDPCVLPHYPYRDDAIILYDSIFDYVGKIVKNYYDYPKKISQDTELQNWRKELVQKKKDGGCQLQGVPGAENGFSSRLQITDTITSIIA<br>NCTIFHAAVDMPQYEECAFVPNYPLLLTGPIPRSKNALSESEIVEQLPNVCTIKEIINVVKILSADNKSRLGYSKTVYFAQENDISAHKEFL<br>EELSFLINSFKRRNEYRKYTYIWMNPEVIPNHITA	77376.08
>TRINITY_DN13878_c0_g1::TRINITY_DN13878_c0_g1_i1::g.9605::m.9605 XP_067137235.1 alpha-2-macroglobulin-like protein 1 isoform X2 *[Centruroides vittatus]*	>Tcham_98<br>KRSFIAISPKIFDVGKKETIHVTVIDVDTPGKVSVRLIDNNGTVFSEVNQDFDKEGSKKVELDVPPNLVNDHYSGNSNYRLQLIGNFGD<br>YKFDTNETITFRTNRELLFLSTDKPFYKAGQTVKFRVFVINSDLKPLKEDPVGSVWITDRNTIRVAQWMNVTFENGFLHRELELPEEPA<br>LGSWTLNVELNNLYTTNYFDVKEYVLPTFEVKIISPPYYLQHQENVTWKICAKYTYGKPVKGNMKSEVKFSTGYYKPDLTLRKEEEING<br>CTNVTVNLNDTDEYRKHSYHGLQFFAEVTEEGTDTKVNATSYIYPTINEYIISRDDNRQYFRPNIPFRTKVIVRKPDNKPVVDEVFKVC<br>ASVRRENWRKDRQITDNVNICKEFKTNKDGVIFITIPPQLPNTLDINIEVKLAYSTKTGVRGFRFSLTQWYSATGHNLQIEPVSAVAEC<br>GNSTEITVFYTSNGAETRKIYYQVASKGDIVDDGLLSFTFDPKDDLSKQKDETYLDLESGLECQKEEDWDNKFPSLEYLGKSTFKLDVDF<br>KISPRARVILYSVTEDGNIYADSTNFEVKPCFKNQVEMKFSDTKQFPGSNVFIHVTAAPRSLCSIGVVDKSVYLLQGGDVLTKSKVYDR<br>LRLLDTSEYAYPYQRTSDFVYCSKKNQSAAEPVGFVDAITAFTRAGVLVLSDLELETRPCKPSFLLGGRYGGPCIGSHVESDMAFGSPR<br>PTAFAAEVAPLSGVAGPSVGLTTTNGVDSFDVTRGGFGGQQPTTTVRVYFPETWLWGLHFVDKEGKLFLKETVPDSITEWEGTSVC<br>LHPDTGLGMSDTASIVAFQPFFVSLTLPYSIVRGEVLPVVVTVFNYLKECIRIKITLDQTDDFEVYNGTLFSNECVCGDKSVTKRFYIKPK<br>TLGTVNITVTAATTKEVEVCENFDVTDIDRSDSLTKPLIVEAEGFPKEEINNVFFCMEDITGSVFTTEHELLRRDDEVEGSGRAFVFVTG<br>DVMGPTLEGLERLVRVPTGCGEQNMVTLVPNIVVLNYLKSTNRLKDNVKQKAESNIRKGYARQMQQYRHPDASYSAFGKHDQSGS<br>MWLTAFVVVSFSEASKYIDIDKYELEKSRYWLTSHQQENGCFPIIGMVHNKNMKGGVGKGNLATLTAYVTSALVRSGNVEESVIRDS<br>KKCIKAKNNPSNYDLAIFANMASLTNDTDAAVEYLKDLLANSVTEGFTTYWENKDKSIAIETASYALLALLRSANEEHFKKAGSIARWL<br>SLQRNSYGGFMSTQDTVMAIQALAEYALRINRHDLNMELAVNGNNLDEIFQLNEENELLTQRTRVPTLPTGIELEAKGEGCALVQIV	166073.68

Sequence identifier	Amino acid sequence[a]	MM*
**Alfa-amylase**		
	HRFNVPNVQTNENFILSVQAQRKQDNCQTASIKICASLGKAENESNMVVLEMKMVSGFEVDKGSVEELLSLSELEIKRYEIKDNQLNL<br>YFTKLTENNKCFSFDVQEKIPIENKQPAIAKIYDYYNQDEQETISYFIDDECRISA	
>TRINITY_DN13878_c0_g1::TRINITY_DN13878_c0_g1_i2::g.9615::m.9615 XP_067137235.1 alpha-2-macroglobulin-like protein 1 isoform X2 *[Centruroides vittatus]*	>Tcham_99<br>KRSFIAISPKIFDVGKKETIHVTVIDVDTPGKVSVRLIDNNGTVFSEVNQDFDKEGSKKVELDVPPNLVNDHYSGNSNYRLQLIGNFGD<br>YKFDTNETITFRTNRELLFLSTDKPFYKAGQTVKFRVFVINSDLKPLKEDPVGSVWITDRNTIRVAQWMNVTFENGFLHRELELPEEPA<br>LGSWTLNVELNNLYTTNYFDVKEYVLPTFEVKIISPPYYLQHQENVTWKICAKYTYGKPVKGNMKSEVKFSTGYYKPDLTLRKEEEING<br>CTNVTVNLNDTDEYRKHSYHGLQFFAEVTEEGTDTKVNATSYIYPTINEYIISRDDNRQYFRPNIPFRTKVIVRKPDNKPVVDEVFKVC<br>ASVRRENWRKDRQITDNVNICKEFKTNKDGVIFITIPPQLPNTLDINIEVKLAYSTKTGVRGFRFSLTQWYSATGHNLQIEPVSAVAEC<br>GNSTEITVFYTSNGAETRKIYYQVASKGDIVDDGLLSFTFDPKDDLSKQKDETYLDLESGLECQKEEDWDNKFPSLEYLGKSTFKLDVDF<br>KISPRARVILYSVTEDGNIYADSTNFEVKPCFKNQVEMKFSDTKQFPGSNVFIHVTAAPRSLCSIGVVDKSVYLLQGGDVLTKSKVYDR<br>LRLLDTSEYAYPYQRTSDFVYCSKKNQSAAEPVGFVDAITAFTRAGVLVLSDLELETRPCKPSFLLGGRYGGPCIGSHVESDMAFDIAA<br>PQLHSFKKGSPRPTAFAAEVAPLSGVAGPSVGLTTTNGVDSFDVTRGGFGGQQPTTTVRVYFPETWLWGLHFVDKEGKLFLKETVP<br>DSITEWEGTSVCLHPDTGLGMSDTASIVAFQPFFVSLTLPYSIVRGEVLPVVVTVFNYLKECIRIKITLDQTDDFEVYNGTLFSNECVCG<br>DKSVTKRFYIKPKTLGTVNITVTAATTKEVEVCENFDVTDIDRSDSLTKPLIVEAEGFPKEEINNVFFCMEDITGSVFTTEHELLRRDDEV<br>EGSGRAFVFVTGDVMGPTLEGLERLVRVPTGCGEQNMVTLVPNIVVLNYLKSTNRLKDNVKQKAESNIRKGYARQMQQYRHPDAS<br>YSAFGKHDQSGSMWLTAFVVVSFSEASKYIDIDKYELEKSRYWLTSHQQENGCFPIIGMVHNKNMKGGVGKGNLATLTAYVTSALV<br>RSGNVEESVIRDSKKCIKAKNNPSNYDLAIFANMASLTNDTDAAVEYLKDLLANSVTEGFTTYWENKDKSIAIETASYALLALLRSANEE<br>HFKKAGSIARWLSLQRNSYGGFMSTQDTVMAIQALAEYALRINRHDLNMELAVNGNNLDEIFQLNEENELLTQRTRVPTLPTGIELE<br>AKGEGCALVQIVHRFNVPNVQTNENFILSVQAQRKQDNCQTASIKICASLGKAENESNMVVLEMKMVSGFEVDKGSVEELLSLSELE<br>IKRYEIKDNQLNLYFTKLTENNKCFSFDVQEKIPIENKQPAIAKIYDYYNQDEQETISYFIDDECRISA	16<br>74<br>10.<br>24
>TRINITY_DN14115_c5_g4::TRINITY_DN14115_c5_g4_i2::g.74085::m.74085 XP_067137151.1 protein disulfide-isomerase-like *[Centruroides vittatus]*	>Tcham_100<br>EVREDDVLVLKKDDFDQQIKTYKYILVEFYAPWCGHCKALTPEYAKAAKQLLEEKSEIQLGKVDATEETELAEKYKVKGYPTIKFFRDGE<br>PIDYTGGRTAKDIVNWLKKKTGPPAATLSTVEEAVKFKDSQDVVIGFFKDSESKLAKEYLDTAFTIDDYPFGITSDENVYKEFKVEKDTII<br>LFKKFDEGKNEFEGEITKDNIKKFIKSNSLPLVIEFSQENAQKIFGGDIKLHNLLFVSKKSEDFDEILKKYRNVAKDFKNKVLFVLINADED<br>DHEKIIEFFGIKKAEQPSMRLIKLEGGMLKYKPETTEFSEENIQTFVKGVLDGKIKQHLLSQDLPEDWDKHPVKVLVNKNFDEVAFDKT<br>KDVIVEFYAPWCGHCKQLVPIYEELGEKYKDREDIVIAKMDATANELEHTKIHSFPTIKLYKKETNEVIEYRGERNLEGLKNFIDSGGKKE<br>KVEKEPEEEPKEQDSKKDEL	54<br>92<br>5.5<br>7
>TRINITY_DN13938_c0_g2::TRINITY_DN13938_c0_g2_i2::g.69467::m.69467 XP_067141566.1 TOM1-like protein 2 isoform X2 *[Centruroides vittatus]*	>Tcham_101<br>MAAILAQLGGNPFNTPVGQKIEQATDASLASENWAVNMEICDIINETDEGPKDAIKAIRKRLQQNAGKNYTVVMYTLTVLETCVKN<br>CGRRFHLLVTQKDFVQDLVKLIGPKNDPPTAVQEKVLSLIQSWADAFRAIAEMQGVVLVYNDLKQKGIEFPMTDLDAMAPIHTPQR<br>SVPPSKPPKMQPVERVPPQVQPQIQPATENPTSPLNLLPDQLAKLRSELDVVQGNMKVFGEMLTELTPGQEHPSDMELLQDLQKT<br>CHCMQSRIVELIDKVANEEVTNELLRVNDELNNLFLRYERFEKKRTTVNQSASSPIQNKQSPPQPEGKPLIDFGESEGTSADDLHKQLA<br>GMDVGSASASVALSQINTVTATNKAPTQSNDDFDMFAQSRNVTYEQSKSSGSTYADNVDPDQVGVTLTSLTNARTQGVELQKEQ	57<br>33<br>5.3<br>3

Sequence identifier	Amino acid sequence[a]	M M*
**Alfa-amylase**		
	DFAEMERWLEDKPPAEESQESITSSEFDKFLAERTAAAEKLPNVEHTSTLSKSPTGSSSASSAANRNAPNNAANRRKMDREDTDNAL FAL	
>TRINITY_DN14094_c0_g1::TRINITY_DN14094_c0_g1_i4::g.16919::m.16919 XP_067143623.1 heat shock 70 kDa protein cognate 4 *[Centruroides vittatus]*	>Tcham_102 MAKTPAIGIDLGTTYSCVGVFQHGKVEIIANDQGNRTTPSYVAFTDTERLIGDAAKNQVAMNPSNTVFDAKRLIGRRFEDSSVQGD MKHWPFDVVSDGGKPKIKVDYKGESKTFYPEEISSMVLTKMKEIAEAYLGKTVTNAVVTVPAYFNDSQRQATKDSGTIAGLNVLRIIN EPTAAAIAYGLDKKGSGERNVLIFDLGGGTFDVSCLTIEDGIFEVKSTAGDTHLGGEDFDNRMVNHFVQEFKRKHKKDLATNKRALR RLRTACERAKRTLSSSTQASIEIDSLFEGIDFYSTITRARFEELNADLFRGTLEPVEKALRDAKMDKSQIHDIVLVGGSTRIPKIQKLLQDFF NGKDLNKSINPDEAVAYGAAVQAAILNGDKSEQVQDLLLLDVTPLSLGIETAGGVMTVLIKRNTTIPTRQTQTFTTYSDNQPGVLIQV YEGERAMTKDNNLLGKFELTGIPPAPRGVPQIEVTFDIDANGIMNVSAVDKSTGKENKITITNDKGRLSKEEIERMVKDAEKYKDDDD KQKGRISAKNSLESYAFNIKSTVDDEKTKDKISEDDKKKLLDKVEETIKWLDTNQLAEKEEFEHKQKELEGVCNPIITKLYQAGGVPGG MPGGFPGGAPGAGPTSGGGSGPTIEEVD	70 81 9.9 6
>TRINITY_DN14345_c0_g1::TRINITY_DN14345_c0_g1_i1::g.3946::m.3946 XP_067144333.1 ras-related protein rab7-like *[Centruroides vittatus]*	>Tcham_103 MASRKKVLLKVIILGDSGVGKTSLMNQYVNKKFSNQYKATIGADFLTKEVMVDDRLVTMQIWDTAGQERFQSLGVAFYRGADCCV LVFDVTAPSTFKSLDSWRDEFLIQASPRDPENFPFVVVGNKIDLENRAVLTKRAQSWCQSKNNIPYYETSAKEAINVQQAFQTVAKN AIAQESDMDLYNEFPDQIKLTNEQRPKTDTCAC	23 17 5.3 8
>TRINITY_DN14378_c6_g1::TRINITY_DN14378_c6_g1_i1::g.5090::m.5090 XP_023237525.1 polyubiquitin-C [*Centruroides sculpturatus*]	>Tcham_104 MQIFVKTLTGKTITLEVEPSDTIENVKAKIQDKEGIPPDQQRLIFAGKQLEDGRTLSDYNIQKESTLHLVLRLRGGMQIFVKTLTGKTITL EVEPSDTIENVKAKIQDKEGIPPDQQRLIFAGKQLEDGRTLSDYNIQKESTLHLVLRLRGGMQIFVKTLTGKTITLEVEPSDTIENVKAKI QDKEGIPPDQQRLIFAGKQLEDGRTLSDYNIQKESTLHLVLRLRGGMQIFVKTLTGKTITLEVEPSDTIENVKAKIQDKEGIPPDQQRLI FAGKQLEDGRTLSDYNIQKESTLHLVLRLRGGMQIFVKTLTGKTITLEVEPSDTIENVKAKIQDKEGIPPDQQRLIFAGKQLEDGRTLSD YNIQKESTLHLVLRLRGGMQIFVKTLTGKTITLEVEPSDTIENVKAKIQDKEGIPPDQQRLIFAGKQLEDGRTLSDYNIQKESTLHLVLRL RGGDF	51 56 1.2 6
>TRINITY_DN14378_c6_g2::TRINITY_DN14378_c6_g2_i1::g.5092::m.5092 XP_067132182.1 ubiquitin-ribosomal protein eL40 fusion protein [*Centruroides vittatus*]	>Tcham_105 MQIFVKTLTGKTITLEVEPSDTIENVKAKIQDKEGIPPDQQRLIFAGKQLEDGRTLSDYNIQKESTLHLVLRLRGGVIEPSLRILAQKYNC DKMICRKCYARLHPKATNCRKRKCGHSNNLRPKKKIK	14 70 1.2 9
>TRINITY_DN14378_c6_g3::TRINITY_DN14378_c	>Tcham_106	59 98

Sequence identifier	Amino acid sequence[a]	M M*
**Alfa-amylase**		
6_g3_i2::g.5094::m.5094 XP_023237525.1 polyubiquitin-C *[Centruroides sculpturatus]*	MQIFVKTLTGKTITLEVEPSDTIENVKTKIQDKEGIPPDQQRLIFAGKQLEDGRTLSDYNIQKESTLHLVLRLRGGMQIFVKTLTGKTITLEVEPSDTIENVKAKIQDKEGIPPDQQRLIFAGKQLEDGRTLSDYNIQKESTLHLVLRLRGGMQIFVKTLTGKTITLEVEPSDTIENVKAKIQDKEGIPPDQQRLIFAGKQLEDGRTLSDYNIQKESTLHLVLRLRGGMQIFVKTLTGKTITLEVEPSDTIENVKAKIQDKEGIPPDQQRLIFAGKQLEDGRTLSDYNIQKESTLHLVLRLRGGMQIFVKTLTGKTITLEVEPSDTIENVKAKIQDKEGIPPDQQRLIFAGKQLEDGRTLSDYNIQKESTLHLVLRLRGGMQIFVKTLTGKTITLEVEPSDTIENVKAKIQDKEGIPPDQQRLIFAGKQLEDGRTLSDYNIQKESTLHLVLRLRGGMQIFVKTLTGKTITLEVEPSDTIENVKAKIQDKEGIPPDQQRLIFAGKQLEDGRTLSDYNIQKESTLHLVLRLRGGN	9.95
>TRINITY_DN14378_c6_g4::TRINITY_DN14378_c6_g4_i1::g.5098::m.5098 XP_067119932.1 ubiquitin-ribosomal protein eS31 fusion protein *[Centruroides vittatus]*	>Tcham_107<br>MQIFVKTLTGKTITLEVEPSDTIENVKAKIQDKEGIPPDQQRLIFAGKQLEDGRTLSDYNIQKESTLHLVLRLRGGAKKRKKKNYTTPKKNKHKKKKVKLAVLKFYKVDENGKITRLRRECPNDECGAGVFMASHFDRQYCGKCCLTFVVYNKPPEA	17900.90
>TRINITY_DN14306_c9_g2::TRINITY_DN14306_c9_g2_i4::g.5584::m.5584 XP_067139465.1 sorting nexin-2-like isoform X1 *[Centruroides vittatus]*	>Tcham_108<br>DRWLALSTGGVSKMADDRVPPPLVEDDDSVVKDDTEDLFISAVDTQLNISNGGDKKAVPPNTIEDLMAGGGVEVSLDDVPLPTPQSKLEFCTPEEEFSKDISTMDKSKEASVENDNSTAAEITLSQPVKPTVAKKIVKPGGEELEEDQFLEIGVDNPHKIGDGMGAYVVYKVTTKTNLPYFKKKLFSVSRRFSDFLGLHDKLVERHLHLGRIIPPAPEKSVIGMTKIKMSKDETVSSTDFIEKRRAALERYLNRTAAHPVLRMDPDFRDFLETEAELPKATNTSALSGAGVIRLFNRVGDTVNKMTFKMDESDPVLLTNPHYAIQWFEEKQHQIENLDQQLRKLHSSVEALVHHRKELSSSTASFAKSAAMMGNCEEHTALSRALSQLAEVEEKIEQLQCQQANHDFYLLSELLKDYISLIAAVKDVFHQRVKVYQTWQHAVQILTKKREQKAKLELAGKTDKIPQAREEVIEWEAKVERGQEEFENISKMIRKEVERFEQTRVQDFKATIIKYMEQLLETQQQLIKYWEGFIPEAKAIA	61155.51
>TRINITY_DN14315_c0_g4::TRINITY_DN14315_c0_g4_i1::g.5821::m.5821 XP_067144536.1 heat shock 70 kDa protein cognate 4-like *[Centruroides vittatus]*	>Tcham_109<br>MAKTPAIGIDLGTTYSCVGVFQHGKVEIIANDQGNRTTPSYVAFTDTERLIGDAAKNQVAMNPSNTVFDAKRLIGRRFDDTTVQSDMKHWPFQVINDNTKPKIQVDYKGETKTFFPEEISSMVLTKMKEIAEAYLGKTVTNAVVTVPAYFNDSQRQATKDSGTIAGLNVLRIINEPTAAAIAYGLDKKGSGERNVLIFDLGGGTFDVSCLTIEDGIFEVKSTAGDTHLGGEDFDNRMVNHFVQEFKRKHKKDLTTNKRALRRLRTACERAKRTLSSSTQASIEIDSLFEGIDFYSTITRARFEELNADLFRGTLEPVEKALRDAKMDKSQIHDIVLVGGSTRIPKIQKLLQDFFNGKDLNKSINPDEAVAYGAAVQAAILNGDKSEQVQDLLLLDVTPLSLGIETAGGVMTVLIKRNTTIPTRQTQTFTTYSDNQPGVLIQVYEGERAMTKDNNLLGKFELTGIPPAPRGVPQIEVTFDIDANGIMNVSAVDKSTGKENKITITNDKGRLSKEEIEKMVKDAEKYREEDDKQKERVSAKNSLESYAFNIKSTIDDEKLKDKISEDDKKKVLDKVEETIKWLDVNQLAEKEEFEHKQKELEGICNPIVTKLYQAGGMPGGMPGGMPGAPGGAGAGAGAGTGGSGPTIEEVD	71302.61
>TRINITY_DN13246_c0_g1::TRINITY_DN13246_c	>Tcham_110	5426

Sequence identifier	Amino acid sequence[a]	MM*
**Alfa-amylase**		
0_g1_i8::g.18200::m.18200 XP_023236966.1 kielin/chordin-like protein [*Centruroides sculpturatus*	MCPDITDPQFKHCSPPKCNSGCQIKTDVAPGECPRCECHDQINYCSPPKCDPWCHIQTDVAPGECPKCICPDIKNPNFKHCSPPKCEEPCSIHNDVAPGSCPGCVCPDGLMTDPNIKHCSLPKCYPWCHVKTDVAPGECPGCVCPDITDPNFKHCSPPKCDPQCHIETNVAPGNCPGCVCPDVSITNSHFKHCSPPKCDSSCYIQINVSHGSCPGCVCPAGDRIGQRHCSMPLCVAPCHFQWNVNPSECPSCVCPATEDFSLHCSMPKCDPHCEIVKNVLPGHCPKCICPENEKIPVVLCSDPKCSPQCYPRKLPEEPCPGCVCPVKDAYKRYCSMPKCEVPCKIKLDVPFGMCPSCICPGSDNEKFCSSPKCEKPCEIQRNVISGECPGCICPKNIMTEQHRFQKYCSPPLCPSQCTIQTDLPPGECPGCDCPNKDLHCSTPRCDSPCRALVVKPGGCPTCVCPENLRKHCSIPNCQPPCEVEKIVTYGECPGCICPAT	3.04
>TRINITY_DN13246_c0_g1::TRINITY_DN13246_c0_g1_i1::g.18218::m.18218 XP_067145227.1 kielin/chordin-like protein isoform X1 [*Centruroides vittatus*]	>Tcham_111<br>NFKHCSLPKCDPGCHIKIDVAPGECPGCICPDITDPQFKHCSSPKCNPGCHIKTDVAPGECPRCECLDQIKHCSPPKCDPWCHIQTDVAPGECPGCVCPDITDPQFKHCSSPNCNPGCHIKTDVAPGECPRCECHDQINHCSPPKCDPWCHIQTDVAPGECPGCVCPDITDPQFKHCSPPKCNSGCQIKTDVAPGECPRCECHDQINYCSPPKCDPWCHIQTDVAPGECPKCICPDIKNPNFKHCSPPKCEEPCSIHNDVAPGSCPGCVCPDGLMTDPNIKHCSLPKCYPWCHVKTDVAPGECPGCVCPDITDPNFKHCSPPKCDPQCHIETNVAPGNCPGCVCPDVSITNSHFKHCSPPKCDSSCYIQINVSHGSCPGCVCPAGDRIGQRHCSMPLCVAPCHFQWNVNPSECPSCVCPATEDFSLHCSMPKCDPHCEIVKNVLPGHCPKCICPENEKIPVVLCSDPKCSPQCYPRKLPEEPCPGCVCPVKDAYKRYCSMPKCEVPCKIKLDVPFGMCPSCICPGSDNEKFCSSPKCEKPCEIQRNVISGECPGCICPKNIMTEQHRFQKYCSPPLCPSQCTIQTDLPPGECPGCDCPNKDLHCSTPRCDSPCRALVVKPGGCPTCVCPENLRKHCSIPNCQPPCEVEKIVTYGECPGCICPAT	71911.21
>TRINITY_DN13246_c0_g1::TRINITY_DN13246_c0_g1_i3::g.18224::m.18224 XP_023236966.1 kielin/chordin-like protein *[Centruroides sculpturatus]*	>Tcham_112<br>AYRGKDGCAHCMCAPLCPTISCDSECEVIRNKATDCPVCKCKDTAIHRLFCSSPKCEEGCHIQSALPGECPGCVCPGTSLVEVHCSQPKCNPGCSIQSVSHGQCPGCVCFDITNPNFKHCSPPKCDVPCSIQTNVAPGECPRCICPGDSITDPNFKHCSSPKCEAPCFIKIDVAPGLCPGCVCPADFIIDPNVPHCSTPKCEPWCHIKTNVDIGECPGCECPDLTDSHFKHCSLPKCDAPCHIQTNVVPGECPVCVCPGDPNFNYCSPPKCDPWCHIQTDVAPGECPGCNPGCHIKTDVAPGECPRCECHDQINHCSPPKCDPWCHIQTDVAPGECPGCVCPDITDPQFKHCSPPKCNSGCQIKTDVAPGECPRCECHDQINYCSPPKCDPWCHIQTDVAPGECPKCICPDIKNPNFKHCSPPKCEEPCSIHNDVAPGSCPGCVCPDGLMTDPNIKHCSLPKCYPWCHVKTDVAPGECPGCVCPDITDPNFKHCSPPKCDPQCHIETNVAPGNCPGCVCPDVSITNSHFKHCSPPKCDSSCYIQINVSHGSCPGCVCPAGDRIGQRHCSMPLCVAPCHFQWNVNPSECPSCVCPATEDFSLHCSMPKCDPHCEIVKNVLPGHCPKCICPENEKIPVVLCSDPKCSPQCYPRKLPEEPCPGCVCPVKDAYKRYCSMPKCEVPCKIKLDVPFGMCPSCICPGSDNEKFCSSPKCEKPCEIQRNVISGECPGCICPKNIMTEQHRFQKYCSPPLCPSQCTIQTDLPPGECPGCDCPNKDLHCSTPRCDSPCRALVVKPGGCPTCVCPENLRKHCSIPNCQPPCEVEKIVTYGECPGCICPAT	90327.45
>TRINITY_DN12930_c0_g1::TRINITY_DN12930_c0_g1_i3::g.30108::m.30108 XP_023219848.1 tumor protein D54-like isoform X2 *[Centruroides sculpturatus]*	>Tcham_113<br>MLDHAEDMSTREMTKTFKLDLTDNYHQYLKIAADENDDKYSFNEPPEVKGYDATPDSGIHDLSSMYPEQRAKLEEELKNELAKTEDEITTLRQVLSDRVRHAQELKRKLGITPWKEFKDDMEQGFRNIQESSAYKEVGTKLTEWNSAVVGAPIYQKTSSTLKTAGEKTTTAIGSISASVTKKLSEVKNSTAFKSFEERVGSAYSNVKTKMAGSRSSSMNSFEEALSNAEGRSSNVASPATTPVIPEGKPLS	27910.03

Sequence identifier	Amino acid sequence[a]	MM*
**Alfa-amylase**		
>TRINITY_DN12930_c0_g1::TRINITY_DN12930_c0_g1_i2::g.30109::m.30109 XP_023219850.1 tumor protein D54-like isoform X4 *[Centruroides sculpturatus]*	>Tcham_114<br>MAHTPEGNFTDFSIQDSFNEPPEVKGYDATPDSGIHDLSSMYPEQRAKLEEELKNELAKTEDEITTLRQVLSDRVRHAQELKRKLGITPWKEFKDDMEQGFRNIQESSAYQKTSSTLKTAGEKTTTAIGSISASVTKKLSEVKNSTAFKSFEERVGSAYSNVKTKMAGSRSSSMNSFEEALSNAEGRSSNVASPATTPVIPEGKPLS	22764.20
>TRINITY_DN12930_c0_g1::TRINITY_DN12930_c0_g1_i1::g.30110::m.30110 XP_023219850.1 tumor protein D54-like isoform X4 *[Centruroides sculpturatus]*	>Tcham_115<br>MAHTPEGNFTDFSIQDSFNEPPEVKGYDATPDSGIHDLSSMYPEQRAKLEEELKNELAKTEDEITTLRQVLSDRVRHAQELKRKLGITPWKEFKDDMEQGFRNIQESSAYKEVGTKLTEWNSAVVGAPIYQKTSSTLKTAGEKTTTAIGSISASVTKKLSEVKNSTAFKSFEERVGSAYSNVKTKMAGSRSSSMNSFEEALSNAEGRSSNVASPATTPVIPEGKPLS	24908.66
>TRINITY_DN12972_c0_g1::TRINITY_DN12972_c0_g1_i1::g.30308::m.30308 XP_067136263.1 actin, clone 403-like *[Centruroides vittatus]*	>Tcham_116<br>MCDDEVAALVVDNGSGMCKAGFAGDDAPRAVFPSIVGRPRHQGVMVGMGQKDSYVGDEAQSKRGILSLKYPIEHGIVTNWDDMEKIWHHTFYNELRVAPEEHPVLLTEAPLNPKANREKMTQIMFETFNTPAMYVAIQAVLSLYASGRTTGIVLDSGDGVSHTVPIYEGYALPHAILRLDLAGRDLTDYLMKILTERGYSFTTTAEREIVRDIKEKLCYVALDFEQEMATAASSSSLEKSYELPDGQVITIGNERFRCPEALFQPSFVGMESCGIHETTFNSIMKCDIDIRKDLYANTVLSGGTTMYPGIADRMQKEITALAPSTMKIKIIAPPERKYSVWIGGSILASLSTFQQMWISKQEYDESGPSIVHRKCF	41793.79
>TRINITY_DN11296_c0_g1::TRINITY_DN11296_c0_g1_i2::g.38490::m.38490 XP_067128565.1 ras-related protein Rab-2-like isoform X2 *[Centruroides vittatus]*	>Tcham_117<br>MSYAYLFKYIIIGDTGVGKSCLLLQFTDKRFQPVHDLTIGVEFGARMITIEGKQVKLQIWDTAGQEAFRSITRSYYRGAAGALLVYDITRKDTFSHLASWLEDAKQHSNSNMVIMLIGNKCDLESKREVNREEGEIFAREHGLIFMETSAKTAANVEEAFINTAKEIYDKIQGGAFDISNEANGIKIGTQHSPTNRPLPVGSQGSGQGNGCC	23451.58
>TRINITY_DN14595_c0_g2::TRINITY_DN14595_c0_g2_i10::g.45143::m.45143 XP_067127317.1 nicotinamide N-methyltransferase-like *[Centruroides vittatus]*	>Tcham_118<br>MEAVCEDKYAFSKFYEDVNFTLNYRDTITNDMKNEEEFVSYCLHKIFQDKAFQGKRLIDIGSGPTVHKLAAASAYFSEIILSDYSQHNRKEIQKWLKNESSAINWDSLFQQEAILEGYRDVEEGINEIKLRLRQKIKNVIHCDVLQKEILPINIGKFDILFTSYCLEAACISLESFDSVIKNLSNYILPNGGLVMATVLGCPQYQIEGYDKTISSLSIDPEFVNNTIKEAGFIIKHSYLMENHEENPCGKFNGILVLSATKSI	30042.25

Sequence identifier	Amino acid sequence[a]	MM*
**Alfa-amylase**		
>TRINITY_DN14595_c0_g2::TRINITY_DN14595_c0_g2_i6::g.45152::m.45152 XP_067138462.1 nicotinamide N-methyltransferase-like *[Centruroides vittatus]*	>Tcham_119<br>MEAVCEDKYAFSKFYEDVNFTLNYRDTITNDMKNEEEFVSYCLHKIFQDKAFQGKRLIDIGSGPTVHKLAAASAYFSEIILSDYSQHNRKEIQKWLKNESSAINWDSLFQQEAILEGYRDVEEGINEIKLRLRQKIKNVIHCDVLQKEILPINIGKFDILFTSYCLEAACISLESFDSVIKNLSNYILPNGGLVMATVLGCSQYQMKGYDKTFPV	247 60.29
>TRINITY_DN14578_c6_g1::TRINITY_DN14578_c6_g1_i3::g.46850::m.46850 XP_067126965.1 LOW QUALITY PROTEIN: calcium-dependent secretion activator 1 *[Centruroides vittatus]*	>Tcham_120<br>MLEPSSSEEESDNETVEDESSEATALPPASTNLELPRSSSVSRSLSPSGAGDTLSVGSGSQQWTNSQRPSSPSPSLVSEKEKEKEDPEKSIEKEEEERKKRLQLYVFVLRCIAYPFNAKQPTDMTRRQTKVIKQQLETILGRFQSFLKGETQIAADEAFQNAVQNYTEVFLKSDRVMRMVQSGACSAHDFREVFRNNIEKRVRSLPEIDGLSKETVLTSWMTKFDAIFRGDEDSRKPQSRIQQNIASELILSKEQLYDMFQNILGVKKFEHQLLYNALQLDSADEQAAAIRRELDGRVQKVNEMEKNRKLMPKFVLKEMESLYIEELRSSINQLMVNLESLPVSKGNFSDSKYGLQKFRRYNHSRQSLLSASANFFPENSLHKASSYLQAPIWIIIYEALMIAFTSISQSSLSKEGLSEDGETMLSKLDIVLSFTLEVVVMEVKGLKSLAPNRIVYCTMEVEGGEKLQTDHAEASKPMWDTQGDFTTTHPLPIVKIKLYTESPGLLSLDDKELGKVIIKPTPLSPKSPEWYKMIVPKNVPDQDLRIKITVRMDKPQNMKHCGYLFAQGKQVWKKWKKRYFVLVQVSQYTFAMCSFREKKCEPTEMMQLDSYTVDYIEPVPDLEGGRFFFNAVKEGDSVLFASEDENECHLWVMAMYRATGQSHKPTPLVQPLPSKNSTISRLQGDADRARKHGMDEFISADPCNFHHHALFRLLQTQALEYRLADIFCSLGWYSPGQLFVLDEYTARYGVRSCYRHLCYLNDLLDRAEKGIMIDPTLIHYSFAFCASHVHGNRPDGIGTVTVEEKDMFAEIKERLRILLENQITHFRYCFPFGRPEGALKATLSLLERVLMKDVMTPAAPEEVRNVIKKCLENAALVNYTRISEQAKNEGAESAPKQDETEEEPAESNKNLEDLIHLAELCVDLLQQNNEHYAEAFAWFSDLLVEHAEIFWSLFAVDMDAVLAQQPPDTWDSFPLFQVLNDYLRIDENLCGGRFHQHLRDTFAPQVVRYVDLMESSIAQSLHKGYEKERWEIKGHGCGTSEDLFWKLDALQTFIRDLHWPDEVFAKHLEQRLKLMACDMLESCINRTLQAFQQWERKGSRWISTDYIVPSEMCAMINVVLEAKNQSLKLCTVDGVDMHQYHAKIDQLVDETLNLMMTGLINKLTSVLEATLNKLSRYDEGSILAPILSLTYRQGMSSSGREVGLAYINFARNSMEQLRQKVTDELWVLSLFEQWYASQMQMLCTWLSERIDRGLHSFQLSALSHIGKKLYSDFELQGIEEEKLNSKIYQTISSRLQLENATASVSEAAHREKEDSE	150824.63
>TRINITY_DN14528_c8_g2::TRINITY_DN14528_c8_g2_i3::g.47183::m.47183 XP_067140417.1 polyunsaturated fatty acid 5-lipoxygenase-like isoform X1 *[Centruroides vittatus]*	>Tcham_121<br>MGNLIPNLKSDYIIEIKSSDRSDAEPGTIIWLILHDERGLKTNAIRLNISVKNEKEMYTFQVTCPLNFDNVDKIEIWRNCYGVGDVFYLEYLTVEKKNGKKLWFPLHRWISSQHRYYFRVYDCCLPQHELSLTQRNDELNDKRKLYEYTEHIEGGPLQIKILPEEEDFTTYLFQWELIHNCKKEKLSIVVEMTSGKWQTFDEIKSFLRKNYTEPSCVNRWNDDSWFAMQRLQGINPVSIKLCRELPENFAVDGGELEAFLEGLSVQEALENKKIFIVDHKIMKNLPCKDDKLVCAPIAVFYLNSNGEILPIAIQLFQDRTVDNPVFFPSDPPYTWLLAKMYFNNADALYHRACLCVGRTHLLMESLWISINRNLSPSHPLYKLMAPHFKYIFSINKLMLEKFLVMGGWIDRVTTIGVRGLCHLIARRFSNWKFEEINIPKEIESRGVNDPCVLPHYPYRDDAIILYDSIFDYVGKIVKNYYDYPKKISQDTELQNWRKELVQKKKDGGCQLQGVPGAENGFSSRLQITDTITSIIANCTIFHAAVDMPQYEECAFVPNYPLLLTGPIPRSKNALSESEIVEQLPNVCTIKEIINVVKILSADNKSRLGYSKTVYFAQENDISAHKEFLEELSFLINSFKRRNEYRKYAYIWMNPEVIPNHITA	77336.01

Sequence identifier	Amino acid sequence[a]	MM*
**Alfa-amylase**		
>TRINITY_DN13120_c0_g1::TRINITY_DN13120_c0_g1_i1::g.49446::m.49446 XP_067126872.1 ras-related protein Rab-7a *[Centruroides vittatus]*	>Tcham_122<br>MSSRKKVLLKVIILGDSGVGKTSLMNQYVNKKFSNQYKATIGADFLTKEVTVDDRLVTMQIWDTAGQERFQSLGVAFYRGADCCVL<br>VFDVSTPNTFKSLDSWRDEFLIQASPRDPENFPFVVIGNKIDLENRAISTKRAQGWCQSKNNIPYFETSAKEAINVEQAFQTVAKNAL<br>AQETEVELYNDFPDQIKLSNEQKSKAEGCSC	23061.11
>TRINITY_DN14686_c1_g1::TRINITY_DN14686_c1_g1_i32::g.59393::m.59393 XP_067141325.1 hemocytin-like *[Centruroides vittatus]*	>Tcham_123<br>DPLLLHRQPYRLEDPRLLHRLPYPIVSITGSSTFASPTVSTTVSSSSASPTVSTTFTPTSTTFSPTQSTGFSYSASPIVSTTLTPTLTTFKSPTL<br>STESLTVPESHRLSYRLQDPRHLHLNISKIECKTNEWLSDEIILDINCSKRYICLPVPFEEIKISVKGQDNDLQYWEIHLKHFRDIAFIEIVDS<br>KDLVLVLVYSLDGSESNMTNQLLYFNEFEKVKFKETIRVKSFAFVPLAINESDISQVIMKISVCKDKECENLNETYCPENCEKNNLECNG<br>TACVTSGSCPCVVNSIVYPPGIYIDECRNRTCENSKFTETYISCEDRGKNICKEKQTLFEDRDTCDCDCRCNNISVNCNTSELCIEREKIC<br>DGIKDCINGTDEKCCPVNVTELSNCKRTNGECGRSNATCKFRENTVIMFSKVVYTCLEVCEAILLQERGKKTFRISYHRVYQNEERFTH<br>HVIIVVDNYSYNLPQKELVIVIKDDVETVVSLSASSSYYNEQGGVSVQVIGDAIQFRSEQKGFTVIWRERNIVDIELDACEIDRVEGLCG<br>YFATRAIHDEYRGKDGTCFNKDAEMCSDWTFDKSNDVCGSIYEMENEKLLTAERVCDEISKKVYYTCNVDVTEQTSDNSFRANDKV<br>CKYVIYPNCTTDNRLCQCEALKYLLVEASCKNGIADLLLEYNCRELCPEGFQWDMCDADNTCEDLTFNGNSKKKIAGCFCPKHLVRKG<br>DKCIPSHLCENCVCDIYGDPNYQTFNKRNYRFLSTCPGPYVLVQRKLPSNEPFFKVLSVNEKCIEYVNGTCISGIHVYYNNTKIEIYRNTT<br>VLKNDRLYTSYCEDTSCNFNGIDFKIETETNIYIVKLVEIHVVIEYKIGNATLHIAGEVSIKFSSYTFFNNTEGLCGSCNGDSSDEFRLRNGS<br>VVDDISTFALDWLEEKNKSLCEIYKTDNCTNIQNVSKCEFPQHLNCSIVDNNRYYSRHSGSCKNDLRCNDQITDSDICKYKFLYADNCC<br>AFRLPMDKWLEIENCVIPCKENRTFRCVDICETKCPAERNEQCEFGKQMRMVCACPDGYVERNDKCIKNTECDACSPKPTLNIPARY<br>FNIDNCTNLDPIVNMYECNGKCNDDFSHYTIERPYNFKEPIDYIYKKKCHCCQPSKYINDIITLTCINGLKKNIIIKKPIDCTCIECNATVI	134283.31
>TRINITY_DN10723_c0_g1::TRINITY_DN10723_c0_g1_i2::g.39193::m.39193 XP_067141683.1 cytosol aminopeptidase-like isoform X1 *[Centruroides vittatus]*	>Tcham_124<br>RRLVISTSRKKDRRIKMVVSSVRIFLKVNRIIKFTQHRRSWMSTLVNEKRGLILGAYEKGEKEEGFVLTAAAQKYDAEVQGRLSELLKIS<br>GPTLKKGKTRIFYGIDQQYCCVNVVGLGSSTAGYCEQEECDEKKENIRVAVSVGTKQLRECGAVEIDVDTCNDAEAAAEGGHLGLFT<br>YDELKAKSSQKPIVNLRPFGNEGLDKWKIGMVKAEGQNIARRLMESPANVMTPTRFGEIAHSLLTPLGVQVIVRDKAWAESQKMG<br>SYLSVAKGSDEPPKFVELSYDGAGPGVKHLALVGKGITFDSGGISIKPSLHMDLMRADMGGAACIIGSVYAVASLKIPINIVGLMPLCE<br>NLPSGHANKPGDVVTAMNGKTIQVDNTDAEGRLILADALCYAHTFNPQMILDLATLTGAMDVALGSGATGAFTNSTKMWEIIHDA<br>GKYTGDRIWRMPLFHHFSKQVTECHLADLCNIGKHGRSGGCCTAAAFLWEFVTHPQWMHLDIAGVMENKDEYQYLCKGLAGRPT<br>RTLVEFMSALTKHS	58359.35
>TRINITY_DN13938_c0_g2::TRINITY_DN13938_c0_g2_i2::g.69467::m.69467 XP_067141566.1 TOM1-like protein 2	>Tcham_125<br>MDGSPEAYDTLCGSISYEYMKPTLDYHLFTMDWSDDDIILDIGCGPGRITKNIFLPKCPKFKKIVAIDIASNYIELAKNKYFHERIEYKTQ<br>DILQSLNPEEIGKYDKIVSFFAFHLITDYQRLFSIISSLLKPGGYFLFSIITKCPLFFALRELSTDQEWNEFIKTEDVLAAVPPTINWKDPGAE<br>FNNIVSKFGLRVSKNKCDSPNFVYSDIKHLLDFNFVALPRGVFKNLEPVVKNRLWKKAESIYPKYCTQMPNGKTSISYEVLNISGKKVM	31560.53

Sequence identifier	Amino acid sequence[a]	MM*
**Alfa-amylase**		
isoform X2 [Centruroides vittatus]		
>TRINITY_DN14231_c7_g1::TRINITY_DN14231_c7_g1_i2::g.64906::m.64906 XP_023238447.1 nicolin-1-like isoform X1 *[Centruroides sculpturatus]*	>Tcham_126 MAAVGPTGAVKPRALDANAYATKKSVAQGMLDVALLTANASQLKYLLRMGNRLDFYYPMMVLVCMSIALQVGEITFKNVYTAYMTVLCKRVSSDGDANNRSALKWHKCIHKYVLMPNPHFENGSQSFFSFTQKDSRLALLDLVTIRLVLYQPSPLWRKFGLEKFAVYSAPSWEEALGMTVPRPFLSQSHGPSHPHPSVEERNKCAASHVQNLWALLEQAKSNKSDLSVGRFEVNGSYDINFLSCN	27371.60
>TRINITY_DN14231_c7_g1::TRINITY_DN14231_c7_g1_i1::g.64912::m.64912 XP_067126796.1 ninjurin-1-like isoform X2 *[Centruroides vittatus]*	>Tcham_127 MAAVGPTGAVKPRALDANAYATKKSVAQGMLDVALLTANASQLKYLLRMGNRLDFYYPMMVLVCMSIALQICVGVVILVKSRFSINDVNRQSSAEKLNDALVWLIFATTVVNVFLSVFGIEPVFQVASNNSTAAP	14530.13
>TRINITY_DN14231_c7_g1::TRINITY_DN14231_c7_g1_i6::g.64920::m.64920 XP_067126798.1 ninjurin-1-like isoform X4 *[Centruroides vittatus]*	>Tcham_128 MAAVGPTGAVKPRALDANAYATKKSVAQGMLDVALLTANASQLKYLLRMGNRLDFYYPMMVLVCMSIALQIAVGGIIVILGSTDLNQQKNYRKADVLNNVVTLFIFFISILNILISAFGMQETDQLVI	13915.55
>TRINITY_DN14565_c3_g1::TRINITY_DN14565_c3_g1_i3::g.46143::m.46143 Tja_PK_01	>Tcham_PK_01 RCEEQSDCPPGYCCKQASSFQSPNCFPLLKKGEKCSVKDDINYGIYKFRCPCEEGLSCTQFAAGIWEGQCN	7940.99
**Uncharacterized Protein**		
>TRINITY_DN12484_c0_g1::TRINITY_DN12484_c0_g1_i1::g.57435::m.57435 XP_023217415.1 uncharacterized protein LOC111619828 isoform X2 [*Centruroides sculpturatus*]	>Tcham_Unk_01 QSRPNVPNGWKLGRRNRNWLREMSWKKRSYAFRPSEVARECCPSVLEISEPRGGRSRSGMLMDLYRDRNSTQKFYEMVCRRDVLDKRCDYVGEKLRPYSRCVQRYGLSYALVRKHGTEEDWRLDYIQIRSGCSCELRFEILRHSEDGL	17734.22

Sequence identifier	Amino acid sequence[a]	MM*
**Alfa-amylase**		
>TRINITY_DN10660_c0_g1::TRINITY_DN10660_c0_g1_i1::g.34473::m.34473 XP_023218154.1 uncharacterized protein LOC111620464 [*Centruroides sculpturatus*]	>Tcham_Unk_02<br>MADECYNEDELILIPFDRSNLWEKKLKPTLATMKIWGLYVDHNICHSIWSILVFVYIHIYTFYMLICTGKIIYLKESFFDAIHSIIYLIIAANVLLAYYKMNFCMHRLNISFSSISNHNFLEFADYRVSLRDKFRMTIFWLSCISYTIYIVWDFVTSELNYYYQKRLFGLDPEIFPKFIAYAIPIVIELSFALTIKAAEKLMCFYFASICRFIAFEYTSLTRSVSNISSNWNRDPGRQRLFLSMDRYTDLCDMVAIIDSVMSPLLFLWTLSLALQITYDGNYICKAVNFCSDPCYCIQYFIEFLIVLVALIDVFMSSSSITKEAQELGTIIYKIAGSQDCFGKDHLRSRIGLWYWKIVTDPPKISGFGTYSINRSCLLNILGVVITFVAIMYELA	46123.20
>TRINITY_DN14121_c1_g2::TRINITY_DN14121_c1_g2_i6::g.73483::m.73483 XP_023225614.1 uncharacterized protein LOC111626467 [*Centruroides sculpturatus*]	>Tcham_Unk_03<br>IVCSPNYCDSVRCQNVENCPYGVIKKSASFCGCCDLCVTLIYKGAACPIPIRGLPVTSECATGLHCDNVTNTCIEP	8076.40
>TRINITY_DN13641_c0_g1::TRINITY_DN13641_c0_g1_i1::g.29946::m.29946 XP_067131550.1 uncharacterized protein Sap-r isoform X2 [*Centruroides vittatus*]	>Tcham_Unk_04<br>QKPPSACIDPTFACQDLQSAKLCGTVKDCITDIWSTLSAPEDNNEICDLCKQMVEEARNQLLSNETQEEIKEVFEGSCALIPIKVVADECKKLVDEFIPELIEMLASRMDPNMVCTVAGLCNGEISKELSSADIISLFTKFKNNYCDDCKSVFNAMKQIFTKIPQNEMKDMLSKVCDQLPESDKCQVIIKSYLPLVYDFVTSMDASDVCSMIGECNGQKTLNKVNKYQDNMTCEFCEAMVQHLRDLILTNTTKEEFKTALLNLCSLFKSFASECKELVNDYFDMLYDLLNQVLNPQTLCTYLGLCSSSNKIEAKIIITPLIKMWNGKHADRDRQSTQSNSVTPLRNSIPALKIIPQYISHENKYNRKESNDIKSIINQLPLERLQHPILSFKQDTDCAFCKAVMFFLSKDISQRDEKSIEDSLDKVCTKWIPSYSKECKSFVDGYYKKVVELLLLGVKFEEVCDHLNTCRPEKIIATRILTPTESKSDFQCEICEKVMTYLEDELKDKATEEEIREALDKVCTVLPTSMSTQCKSFVDKYTDTIVALIIQEVKPSAICSTLGFCSKNFKVPVKSQFTCELCQDVMTYLKQVLKDPKTEDDIKQALDEVCSILPQSLTQECQDFVNQYTDLIVSLLLQEMDPDKICAEIGLCPKNNTPSKVKDQNVECTLCKQIMAALENELKDKSTENEIREALDTVCSLLPGSYGKKCQDFIDKYTETIVTLLVENIKPRMICSLLNICPSENLECTLCQYFLHFLQKELMSNSTESEIKNLLEKVCSYLPSSLSTECDAFVGEYGDAIIVLVAQQIDPSLVCPLFKVCPSVSSVKEVKLNTCQVCRAAVNYIDQLLEEDSIDKEITDLASRVCKFLPATSQQECVSIIELYGPSILKEIGKLANNQQVCQSIDLCPRTEGLAHLVGSEKCTYGPGYWCKSSAHAAACKAEEFCRRKVWKTT	107509.11
>TRINITY_DN12417_c0_g1::TRINITY_DN12417_c0_g1_i2::g.57479::m.57479 XP_067128167.1 uncharacterized protein [*Centruroides vittatus*]	>Tcham_Unk_05<br>MGVQDKQNRILPLFEHLTSLTTDQVPVGERDDRLKDVGHLERGKLFSCFHTDHLDEAESLFTVLFQAKDFEDFMHLCEQARNVVNEGLFVFAVSVAVLHRDDCKGVTVPPIQEIFPDRFVPAETINHALKADRKRAGDEDVIIKIEETGNILDPEYHLAYFREDIEVNAHHWHWHLVYPANWKPEVMGKVKDRKGELFYYMHQQMCARYDCDRLSTGLKRMTPFHNFDEALEGYSPHLTSLISGLNYASRPAGLSMHDLNDVNVQEMVRWRERVLDAIHVGHVIDDHGHEIPLDEEHGIDILGSLLESSAESKNKGYYGTLHNWGHVILARVHDPDGRFRENPGVMSDTSTSLRDPIFYRLHRFMDNMFQEYKASLPCYDEHDLDFPGVHVEHVTDAKVPDVVTTTMKEDVLQLSHGIKTKGSVKVKFEH	72039.28

Sequence identifier	Amino acid sequence[a]	M M*
**Alfa-amylase**		
	LDHEPFSYSIEIQNKTGAVKHATVRVFLAPKYDELGNRMTLNDQRGLYIELDKFHKILEPGKNEIKRDASSSSVTITHVPTFDELKQGKG VSEDNTEFCSCGWPSHMLLPRGNERGMVFQLFVMLSDYEKDHVGGVNDKAICSDAVSYCGAKDSKYPDKRAMGFPFDRVIKART PSQFATPNMSFTDIRIQHVEHDHH	
>TRINITY_DN13550_c0_ g2::TRINITY_DN13550_c 0_g2_i1::g.57956::m.579 56 XP_023244022.1 uncharacterized protein LOC111641995 [*Centruroides sculpturatus*]	>Tcham_Unk_06 KPTWTTLRVTWGKNPFSSYNFAKMPLTIGETSDKGFIKLQGECNEGDFYGHRYIKGDDPATILIYDSKGYIAGIQTGIPAYFINQINTTY PYTKNGWFQLGKIDDKDVYFLTVYFMDPRTICESNRTHDEFNENIGRGLWFQQGPMSSQNFMNVPLYQANLSNTKWMKGKCFP TMGKHYWYDTSEDMNCDKTFPFFLLFNRNKLTGFGFFLLGNFQFTNRYEHPTKDVFTKFLEPVPKCLYDFYDKSGGLTTFHIFFTVNP WNLFC	31 15 2.3 8
>TRINITY_DN14227_c6_ g3::TRINITY_DN14227_c 6_g3_i1::g.66387::m.663 87 TaPBC_Unk_03	>Tcham_Unk_07 YTAIGIAPMVDGKCKINGKLHNAGEEYFEEKNCELWKCNGVSPVIDGKIREDESYIDISGCGVATTVTDGKLCRYKSKAGKYPDCCFG KLECSEDKIDDIFS	11 22 7.7 4
>TRINITY_DN11569_c0_ g2::TRINITY_DN11569_c 0_g2_i2::g.493::m.493 Tja_Unk_05	>Tcham_Unk_08 LYDIFKRQICHDLGSSCSGSRQDCCDPYCCSFGECSVSC	43 16. 84
>TRINITY_DN14478_c5_ g1::TRINITY_DN14478_c 5_g1_i11::g.16749::m.16 749 Tja_Unk_07	>Tcham_Unk_09 RERRTSIHIIRRISYRGEVRPCRIQTKSYKKVSSVTTRPRRVHFKIFAEKKIRLITNMEGKLWIIFLLVMSITVKCEATSLYDLYCDNKDIAKF YDCFKKTASQKTVNTFNECASKQKIPIAPLERRNFFCDRANEDEVRIWYFCLFKNLKPFELEEFHTSNEKCLTS	20 11 0.4 9
>TRINITY_DN14478_c5_ g1::TRINITY_DN14478_c 5_g1_i27::g.16761::m.16 761 Tja_Unk_07	>Tcham_Unk_10 RERRTSIHIISSIFYRRISYRGEVRPCRIQTKSYKKVSSVTTRPRRVHFKIFAEKKIRLITNMEGKLWIIFLLVMSITVKCEATSLYDLYCDNK DIAKFYDCFKKTASQKTVNTFNECASKQKIPIAPLERRNFFCDRANEDEVRIWYFCLFKNLKPFELEEFHTSNEKCLTS	20 70 8.1 6
>TRINITY_DN14304_c6_ g6::TRINITY_DN14304_c 6_g6_i1::g.4913::m.4913 Tja_Unk_43	>Tcham_Unk_11 EKTCFIVICIKKCITRQKIAACSKTTDCSCSK	35 54. 33
>TRINITY_DN14409_c0_ g1::TRINITY_DN14409_c 0_g1_i2::g.14767::m.147	>Tcham_Unk_12 MACLRWKKKLLIAVKDPIGLNFSNRGKICFGVFAFIVLLLIAGFISYLVLTWKLRPFGRDEHEHDDAQYKPVQLYPNTDSELGIYERAAIS TDTKPCAAVGKGILMKNGNAMETAIATLVCMGAVNSHSMGLGGGFFMTYYDRKNRKVGVLDAREVAPAAATEDMFSEGPQSSQ	69 48

Sequence identifier	Amino acid sequence[a]	MM*
**Alfa-amylase**		
67 XP_067130270.1 scoloptoxin SSD14-like isoform X3 [*Centruroides vittatus*]	EGGLAVAVPGELLGYEAAYNKFKGNLTWKELFTPTIEMCRNGFQVTRHLANALTENRDAVLNEITLRNVYFNNETGDLYKEGEIMRRLDLAATLEKVANSGSDVLYGVGETRRKFVEDIKSFGGIITEDDLHNYTPRWKSPTKITLKNGFEVNSTPIPGSGSLLSYMLNVLDGYNLTKEENWGDLDQISLTFHRVLETFKFAYSQRMNLEDSKDHAVTQIASLLLSKQYADETRAKIYDTKTIHDPEYYGFKQHAREDGGTGHLSVIGPNGDAVSVTSSINVYFGSKRVSPSTGIVLNNQMDDFSLPNVTNYYGLPPSDRNRIKPGRRPMSSMSPTIILDDKGEVWLAVGGNGGSRITSSIALVAMQILWFDKNVKEAIDMPRLHHQLFPDYIMHEKVFPKAILRKLQEYGHAAEVKTGRMSVIMAVNRGKDGKLYSNADYRKNGKTDGF	6.22
>TRINITY_DN14409_c0_g1::TRINITY_DN14409_c0_g1_i3::g.14771::m.14771 XP_023218575.1 uncharacterized protein LOC111620813 [*Centruroides sculpturatus*]	>Tcham_Unk_13<br>MIEKLSREVTSYDVTKDEIKQNKDEIKANKGKEEPAEENFIEFFKWRLNFSNRGKICFGVFAFIVLLLIAGFISYLVLTWKLRPFGRDEHEHDDAQYKPVQLYPNTDSELGIYERAAISTDTKPCAAVGKGILMKNGNAMETAIATLVCMGAVNSHSMGLGGGFFMTYYDRKNRKVGVLDAREVAPAAATEDMFSEGPQSSQEGGLAVAVPGELLGYEAAYNKFKGNLTWKELFTPTIEMCRNGFQVTRHLANALTENRDAVLNEITLRNVYFNNETGDLYKEGEIMRRLDLAATLEKVANSGSDVLYGVGETRRKFVEDIKSFGGIITEDDLHNYTPRWKSPTKITLKNGFEVNSTPIPGSGSLLSYMLNVLDGYNLTKEENWGDLDQISLTFHRVLETFKFAYSQRMNLEDSKDHAVTQIASLLLSKQYADETRAKIYDTKTIHDPEYYGFKQHAREDGGTGHLSVIGPNGDAVSVTSSINVYFGSKRVSPSTGIVLNNQMDDFSLPNVTNYYGLPPSDRNRIKPGRRPMSSMSPTIILDDKGEVWLAVGGNGGSRITSSIALVAMQILWFDKNVKEAIDMPRLHHQLFPDYIMHEKVFPKAILRKLQEYGHAAEVKTGRMSVIMAVNRGKDGKLYSNADYRKNGKTDGF	72994.83
>TRINITY_DN12417_c0_g1::TRINITY_DN12417_c0_g1_i1::g.57486::m.57486 XP_023227775.1 uncharacterized protein LOC111628254 [*Centruroides sculpturatus*]	>Tcham_Unk_14<br>MTVKEKQARLMPLFKHLTALTREKKPLSERDERLKGVGILPRGTLFSCFHSRHLAEATELYVILYGAKDFEDLIHLCEEARQIVNEGIFVYAVSVAILHREDCKGLTVPPIQEVFPDRFIPTETINRANKEAINHPDQANIVVEAEHTGNILDPEYKLSYFREDIGTNAHHWHWHIVYPATWNPAVMKKEKDRKGELFFYMHQQMCARYDSERLSNGLQRMIPFHNFEEPLEGYAPHLTSLVSGLQYASRPEGYSIKDLSDVDVQDMVRWRERILDAINMHYIVDKDNKKIALDVENGTDILGDIIESSDESKNVEYYGSLHNWGHVMMANITDPDHRFQENPGVMSDTSTSLRDPIFYRWHRFIDNIFQEYKCSLHPYTKEELSFPDVTVVNISVNSKTANLITTLTKESELELSHGINFGSDQSVKVKYHHLDHEPFSYSIVVENASSAEKHATVRIFLAPKYDELNNKLEPDEQRRLFIELDKFHYTLPSGKTTIARDHRLSSVTISKVKTFKELNAGELEEASTEYCSCGWPEHMLIPRGSHKGMEFELFVMMTDHDEDTVAGLNENAVCSDAVSYCGAKDDRYPDKKPMGFPFDRKILARTAAEFLTPNMALIDIKIKYQG	72053.58
>TRINITY_DN14545_c3_g1::TRINITY_DN14545_c3_g1_i9::g.44680::m.44680 XP_067129367.1 uncharacterized protein [*Centruroides vittatus*]	>Tcham_Unk_15<br>ADITVDEGNSKVDDILKSIQQNQELEPLQLPDHDIPIVRKILFIDTKVEIQLRKGEIFGLSNISRRNDVRIRKEKTSTLIVSDLTLRNIEVKYQIQLILLNSEHKPTNITGYMNNNRINLKLRVDDETNKLSVDDLTVSRLSGFLVNLNIFGNMFGWIENVFKTVFINILKEDITNLVESNLREVFEKEVEKKFSMI	22841.31
>TRINITY_DN12103_c0_g1::TRINITY_DN12103_c0_g1_i1::g.34282::m.34282 XP_067142110.1	>Tcham_Unk_16<br>DYSKEDINRPNPDLYSLNHVNRLMNIFPRQIDNRNRGVWALPYAISTYDGYDLEGTVSPGHMMKRDGPPLSIVNALDVLRQQLMLDMARQKMKENQNKIRQNDEYLKNLGKRSISSQKDVSRSLDGLHRSSYLDKKVLQTSEV	16578.78

Sequence identifier	Amino acid sequence[a]	MM*
**Alfa-amylase**		
uncharacterized protein Dh44 [*Centruroides vittatus*]		
>TRINITY_DN1870_c0_g1::TRINITY_DN1870_c0_g1_i1::g.36444::m.36444 XP_023238156.1 uncharacterized protein LOC111636989 isoform X1 [*Centruroides sculpturatus*]	>Tcham_Unk_17 WIHECPRLEDIHPCSCNSIHTRNPYEPDVIILCQNIRAEQILKTTLRGMRNHSIDVLIFDNCKLPSFPNDLFKDILVGTTELLYSTVQLNKNFLQCLRNC	11655.50
>TRINITY_DN11795_c0_g1::TRINITY_DN11795_c0_g1_i2::g.34689::m.34689 XP_023238156.1 uncharacterized protein LOC111636989 isoform X1 [*Centruroides sculpturatus*]	>Tcham_Unk_18 WIHECPRMEDIHPCNCYSTLATDPKIPDIVVICQNIKNNRVLSTSLREMRNHTIDLFIFDNCEIPSFPNTVFKDIRIGILELMRSTVQLKKEFVECANRC	11696.63
>TRINITY_DN14377_c0_g1::TRINITY_DN14377_c0_g1_i4::g.5509::m.5509 XP_023232019.1 uncharacterized protein LOC111631914 [*Centruroides sculpturatus*]	>Tcham_Unk_19 TTSMDASDVTWAHAVNSKELLEENLKNDINFLEADVSLGDVTGPNPEYSVPIMAHPPKMISDISLQSWIERVISAKTGKGAKLDFKSIEIVEPSLKILKDQETEGLANERKRTSPLKSSDS	13295.99
>TRINITY_DN14377_c0_g1::TRINITY_DN14377_c0_g1_i1::g.5510::m.5510 XP_023232019.1 uncharacterized protein LOC111631914	>Tcham_Unk_20 TTSMDASDVTWAHAVNSKELLEENLKNDINFLEADVSLGDVTGPNPEYSVPIMAHPPKMISDISLQSWIERVISAKTGKGAKLDFKSIEIVEPSLKILKDQETELTFPIWLNADILPGPINADTEPVNSTAFLKLCKQYFPNVTLSVGWTTRVVLTAEGSYTWHHIKEMLDTLHCTDTEQPVTFPIRAIFVKNSIDQLVWLLGAIPNSTLTVWSSLIDVVDVESLLKLRSRLPFSAVYYDLPPTLLKQFNDRKGSVHPPSVPYNFNHYNWVPINDKEECNDKIYISSLGALFPSGFHGLMLKMEPQNELKLISEIAFINSEAYSSLRLIVCKDEYFPSVFPSGKDLIQLLQESSLISYTFDNKEEYKYFKLTISAHEVSLLVVFEKTSNGKNTQKTIRHDIKHIKNINIVFLSEGESAVLYNAESDAISSSTIPKLSFLNALFIVILSLLRMVVVGYDKM	51814.55

Sequence identifier	Amino acid sequence[a]	MM*
**Alfa-amylase**		
[*Centruroides sculpturatus*]		
>TRINITY_DN14377_c0_g1::TRINITY_DN14377_c0_g1_i2::g.5516::m.5516 XP_023232019.1 uncharacterized protein LOC111631914 [*Centruroides sculpturatus*]	>Tcham_Unk_21<br>TTSMDASDVTWAHAVNSKELLEENLKNDINFLEADVSLGDVTGPNPEYSVPIMAHPPKMISDISLQSWIERVISAKTGKGAKLDFKSI<br>EIVEPSLKILKDQETEVKLINILIPHLNCNK	13182.12
>TRINITY_DN14565_c3_g1::TRINITY_DN14565_c3_g1_i2::g.46136::m.46136 TaPBC_Unk_06	>Tcham_Unk_22<br>RCEEQSDCPPGYCCKQASSFQSPNCFPLLKKGEKCSVKDDINYGIYKFRCPCEEGLSCTQFAVCNFL	7558.66
>TRINITY_DN14049_c0_g2::TRINITY_DN14049_c0_g2_i1::g.13011::m.13011 XP_023220888.1 uncharacterized protein LOC111622699 [*Centruroides sculpturatus*]	>Tcham_Unk_23<br>MKLRSLVLYAAAEHGNSEIIDKAKEMFNNWMKKGVKVEPDLRNVVYMVGIKFGTQEEWNFCWKKYQESLIPSENKLLLNALGSSR<br>DIWQLSQYLNYSLDKDTIRPQDTVSVISRVSYNPVGRLLTWRFVRDNWSKLLDTFGQGSFAMGSIISSVISDFSTRFDYNEIKAFFDKV<br>DVGVGARALNQSLERIRANIYWRENVESSVVQWLRQQTNQNIQS	25324.81
>TRINITY_DN14466_c0_g1::TRINITY_DN14466_c0_g1_i2::g.17169::m.17169 XP_023210948.1 uncharacterized protein LOC111613829 [*Centruroides sculpturatus*]	>Tcham_Unk_24<br>MDKSILQGYLLLKLSGGLSILKPPRKLWLVYQEEKCQLFCYKNEFESVNVYKAPLDVIKLQKAAITPHAEENNHFIIYSDQREYHLFADD<br>YESMMRWIFELQKRRDEWNNQFNTVPIQTDYEKENIENRDEILPNKEEMQIVIQNTKRAEERSENRGNFLSEYSLSQTFGVYKLNND<br>DVISDERQRTNPTPEDPTQTIPKELEESNISCYYPVEECDGSISSDSAVCTSEYHNAKIQEMEAEMRMTHKILSEALSREAAYKSTMERL<br>EDLVEELEDRMSRVQGGIGFWRYLSGDKTLVNFRKLEEKCQILKNHNVFLNDEILKLIRMLQQEQFRFQAKESQIQDIKNQIEQMKR<br>DYIFLFLSSIRIYYGDGPETTEVYLYGGSRHKVRVLSLLEEARKINKDLPTYESLIKPCYVDPMGFEHCFTEESVVLHYICSLLYTHYSKQLR<br>SYEKHVNSWNKYCKQEEKLSIKKEVKFLVRKGIPDYLRKTIWRQFYRDKVHDVMKDKGPYYYKTLVNTVTESQIILYHNRQIALDLLRT<br>MPNNIRFSTPKSEGVNKLEEVLLAFCLHNPYLGYCQGMNFLVGMCLLIFEPEDAFWCLVAITEKFFPSTYFDQSLIGAQADQEVLKDL<br>MKEKLPNLYRHLSSLDIDLRTVTLNWYMSIFFDCVPFETLLRIWDCFLLEGPKVLFRFTLAILKMQEEAILKKKDTVSVMRQLKASAKFC<br>YNVDDLVKVAFEDFHPFPRRSDLATKQTYYAKMLKDSSKKRERRRKSFIDMLPEMMIDWKLSATNRPCIDCATVYDNNKLWLAYCH<br>TNGSQIVKINVEDCIMYKLNIECESRIICMHSATSDILLLGTVSNVLQVYNNKTGRLLSEVKLNDSVLCLHYHNKEEANVIYAGLADGTL<br>AVIESIYEIEKKKEVFYISIGSSPVSCLLELDWRLLCACGNRIFILNSRTLDTLHQLQITCNDMDHISNLMLDGNNIWTSVRGSSILHVWD	154859.54

Sequence identifier	Amino acid sequence[a]	MM*
**Alfa-amylase**		
	SHTLACKLIFDIKHNRPTCHASKEDDSFLQSMNITNILPIDDGVIVGTSDGSLLIYDVEKKMTRSISCTESSIYESVGENIEEDSEFANHPT QNYKFIDKSSDVSLPAAITEYDIDHELTISLQPRGESAPPRLGESVQISRELSTNSLILELENNKNKPTRLDDVENNSISSVDLRQACGFYA VEDNKTDLAECKSLLLANKSNHIVEGKEISFQPVNLTPAVSLVSFNSTDYLYCLELVIKEKIKIGDKPIKCLLNVKCNNEINFVSCTGLYGE EDSVMKWSKEGKMLWTGKQILDICPITPKQSSLSDQKLFSNSMVGAAGSSFIKTRKKSMTEKIELSGINKVQTLFFKLPGK	
>TRINITY_DN14466_c0_g1::TRINITY_DN14466_c0_g1_i8::g.17177::m.17177 XP_023210948.1 uncharacterized protein LOC111613829 [*Centruroides sculpturatus*]	>Tcham_Unk_25 MDKSILQGYLLLKLSGGLSILKPPRKLWLVYQEEKCQLFCYKNEFESVNVYKAPLDVIKLQKAAITPHAEENNHFIIYSDQREYHLFADD YESMMRWIFELQKRRDEWNNQFNTVPIQTDYEKENIENRDEILPNKEEMQIVIQNTKRAEERSENRGNFLSEYSLSQTFGVYKLNND DVISDERQRTNPTPEDPTQTIPKELEESNISCYYPVEECDGSISSDSAVCTSEYHNAKIQEMEAEMRMTHKILSEALSREAAYKSTMERL EDLVEELEDRMSRVQGGIGFWRYLSGDKTLVNFRKLEEKCQILKNHNVFLNDEILKLIRMLQQEQFRFQAKESQIQDIKNQIEQMKR DYIFLFLSSIRIYYGDGPETTEVYLYGGSRHKVRVLSLLEEARKINKDLPTYESLIKPCYVDPMGFEHCFTEESVVLHYICSLLYTHYSKQLR SYEKHVNSWNKYCKQEEKLSIKKEVKFLVRKGIPDYLRKTIWRQFYRDKVHDVMKDKGPYYYKTLVNTVTESQIILYHNRQIALDLLRT MPNNIRFSTPKSEGVNKLEEVLLAFCLHNPYLGYCQGMNFLVGMCLLIFEPEDAFWCLVAITEKFFPSTYFDQSLIGAQADQEVLKDL MKEKLPNLYRHLSSLDIDLRTVTLNWYMSIFFDCVPFETLLRIWDCFLLEGPKVLFRFTLAILKMQEEAILKKKDTVSVMRQLKASAKFC YNVDDLVKVAFEDFHPFPRRSDLATKQTYYAKMLKDSSKKRERRRKSFIDMLPEMMIDWKLSATNRPCIDCATVYDNNKLWLAYCH TNGSQIVKINVEDCIMYKLNIECESRIICMHSATSDILLLGTVSNVLQVYNNKTGFVSHFENKIVIRSKIKRFCPLFALP	10 38 96. 66
>TRINITY_DN14466_c0_g1::TRINITY_DN14466_c0_g1_i3::g.17196::m.17196 XP_023210948.1 uncharacterized protein LOC111613829 [*Centruroides sculpturatus*]	>Tcham_Unk_26 MDKSILQGYLLLKLSGGLSILKPPRKLWLVYQEEKCQLFCYKNEFESVNVYKAPLDVIKLQKAAITPHAEENNHFIIYSDQREYHLFADD YESMMRWIFELQKRRDEWNNQFNTVPIQTDYEKENIENRDEILPNKEEMQIVIQNTKRAEERSENRGNFLSEYSLSQTFGVYKLNND DVISDERQRTNPTPEDPTQTIPKELEESNISCYYPVEECDGSISSDSAVCTSEYHNAKIQEMEAEMRMTHKILSEALSREAAYKSTMERL EDLVEELEDRMSRVQGGIGFWRYLSGDKTLVNFRKLEEKCQILKNHNVFLNDEILKLIRMLQQEQFRFQAKESQIQDIKNQIEQMKR DYIFLFLSSIRIYYGDGPETTEVYLYGGSRHKVRVLSLLEEARKINKDLPTYESLIKPCYVDPMGFEHCFTEESVVLHYICSLLYTHYSKQLR SYEKHVNSWNKYCKQEEKLSIKKEVKFLVRKGIPDYLRKTIWRQFYRDKVHDVMKDKGPYYYKTLVNTVTESQIILYHNRQIALDLLRT MPNNIRFSTPKSEGVILLFLKILLLFF	66 99 5.5 2
>TRINITY_DN14185_c0_g1::TRINITY_DN14185_c0_g1_i11::g.73850::m.73850 No Match	>Tcham_Unk_27 NNEAEIYDESKIRNEEKCKCNNEGEIYDENTKTCTAQCRDKVAEECTKNSATCEIIRNEEKCKCNNEAEIYDESKKTCTALCGNAAEEEC NRNNAKCERIRNEEKCTCNNEGEIYDENTKTCTGGAECLRSNYLFLTFMTLLLKKILL	16 94 8.8 6
>TRINITY_DN14185_c0_g2::TRINITY_DN14185_c0_g2_i4::g.73858::m.73858 No Match	>Tcham_Unk_28 KLIRCSVSCEQRKIDECFRSGKGCETLDNEEICTEKCEGLEMFDKKLKRCSEPCSNEKMKECEGTGKNCEILDNVETCTEKCEGKERFDK KLKRCLEPCSDKKITECEGTGKNCEILDNVETCTEKCKGKERFDRKLIRCSVSCEQRKIDECFRSGKGCE	18 37 8.0 7
>TRINITY_DN11402_c0_g1::TRINITY_DN11402_c	>Tcham_Unk_29 HHDRFCPQDEAGCHRSCVNDHGAGCGYCTGYKDLSCLCLEKSDACKN	51 48. 69

Sequence identifier	Amino acid sequence[a]	MM*
**Alfa-amylase**		
0_g1_i1::g.24934::m.24934 TaPBC_Unk_152		
>TRINITY_DN14051_c2_g2::TRINITY_DN14051_c2_g2_i4::g.14810::m.14810 TaPBC_Unk_86	>Tcham_Unk_30<br>DGDKACSECPPGMGCDSHYKRLCAPIAKTTVPATTAKTTTVNDEILWKCDFSIGTKKQCESEMQCYANWKTVCGLESCHKEIMLHKQKSSLLFLTPILVKNQACLIFEYKKEHLPTAQGESILTAVAVWNEGENYEAIYIDDDTNKWNLARLNIHAENREIQVGFVVRKARDSEGQKISISNLYVLNGSC	212996.34
>TRINITY_DN14063_c0_g1::TRINITY_DN14063_c0_g1_i2::g.13748::m.13748 XP_067123419.1 uncharacterized protein [*Centruroides vittatus*]	>Tcham_Unk_31<br>RKSSRLGYDCNVCQHGGSDVHLETLHVCRRPAPPSPGYISCTYDDKHMKCKGECPSGYKFPDGRNSIEIQCLTKDAVWYPIRSFPNCLPLDYRPYDQPGDQSHDNLDDQSVTLSRCKKPTAPSFGSIRCSYDSENLICTGKCNQGYSFPDGRTEKQLQCNLVRASWYPHGNFPHCYLNKHVGSDDGDSLSVTVRRCRRPPSPAPGTVSCTYDASKMVCVGNCDSGYYFPNGRSEMTLQCNTRDAYWYPLEQFPKCVTYDGIHRQPKSNEGNDEKNIYAVILIRCRKPEAPQAGTSYCSYDTVSMNCTGTCSAGYYFPNGKREMLLQCKAESAVWYPQTDFPACSAYNERYTDDSQQQKVNEYLAVTLSKCGRKKAPAGGYSSCRYDALGMNCTGTCDKGYYFPGKRSEIILRCRTQNGYWYPRNEFPSCVRDENNRYTDESQQQALQVTLSRCGRKEAPARGYVNCRYDAFGMNCTGTCVTGYYFPENSAEIILQCRTENGYWYPRNEFPSCAPYDDGGVQTIDNTDDDTSTYTATLIKCRKPVAPEFGVRFCKYDSVVMNCTGTCRTGYYFPDGEREMPLRCRTESGVWYPRADFPACSQFDSERYTNLDATQQREQINRLLAVTLNKCGRKGSPPSGYVNCSYDISGMNCTGTCRSGYYFPGRRAEMILRCRTENGYWYPENEFPACALHENDKHTDNSQQRPQEVNSQLQITVMKCGRIETPHSGYVNCNYDTLGMNCTGTCKSGYYFPEKRTEIILRCITENGYWYPRNEFPACRSYENENVEESPSQTQVEEDRLIQETLVRCGRKNQPENGYVNCNYDSSIMNCTGRCITNYYFPTGLREMVLQCNKEYGRWYPENEFPKCRQFIYDKSEENPKPENEDLRVTLDKCGMRKAPIAGYVNCEYNAVEMNCTATCLNGYYFPNGRTEMIIQCKTSSRLWVPRSEFPKCSKDITSHIDTTLKPNQEGENSQQEGENSQFLETLRMCIRRTNPTSGYVVYCTHDAVGMNCTGRCRIGEHFPNGGREIVLRCRTDNGLWYPQREFPICSPSTAANLHENQEKDHLVCQNLHTPAHGYISCSDDTWSKNKICVARCKLGYSFPNHQFEMTLRCSKEDGKWSLLQKFPACLPEDEDNMDNQNQLNQENQDEMTGEKCGKPNPPITGYLSCLNHSHTLECTGTCMPDYAFSVGITSMTLTCSFASGKWSPSNTFTPCYQKNQIPSISNEENEDTGNPTTNILCPKLSTPKGGYIHCEQQFSHGYRRCTGTCMTGLSFPNGENSMVFRCSTVRGWLPASSFPACEENKDRIYQPESEISTNNQGTDLTENQTPNTSENNYGNTGNVDHGNTGTTRSFCRSPPSPKNGYVSCLPNVETLDCTARCSQGFHFPNGQNKLLYRCISKNGRWSPSGYFPDCISACDVPCNNGGVCVGRNQCFCTQDYRGNFCQYPLSNCDPLHLKFAGSIRCNHTSDETVCQLGCPQRMILEPSYSTVRCNLEGIWSPRVSPRCIEDPRYKSVDSNYRLCPTSQCSSSSLTADEGGLCRRPPPPKPGYIRCIYNRLIMECTSKCADGYRYANGQTEQIRTCDNRTGIWTPSHYMSECQPVCERPCQNEGSCMSPNFCFCPVKYRGKFCQYPLSNCDPHRLRFAGSIRCSHSSEETVCKLGCPEGTVLDNPSLDTVRCSLEGYWTPQYVPRCIEANVGGRPTYSNQDYHQQSNYQQQYEQSQTNEHSLNYEQRMQRQVQNSQIRESSAENGGSAESQRINTDKEKFRDSELEKESYEQRLQMKQEEEQRKLYDRILKQSSTNRTDQRGNQSRLSDRL	206581.13
>TRINITY_DN14063_c0_g1::TRINITY_DN14063_c0_g1_i5::g.13738::m.13738 XP_067123419.1	>Tcham_Unk_32<br>RKSSRLGYDCNVCQHGGSDVHLETLHVCRRPAPPSPGYISCTYDDKHMKCKGECPSGYKFPDGRNSIEIQCLTKDAVWYPIRSFPNCLPLDYRPYDQPGDQSHDNLDDQSVTLSRCKKPTAPSFGSIRCSYDSENLICTGKCNQGYSFPDGRTEKQLQCNLVRASWYPHGNFPHCYLNKHVGSDDGDSLSVTVRRCRRPPSPAPGTVSCTYDASKMVCVGNCDSGYYFPNGRSEMTLQCNTRDAYWYPLEQFPKCVTYDGIHRQPKSNEGNDEKNIYAVILIRCRKPEAPQAGTSYCSYDTVSMNCTGTCSAGYYFPNGKREMLLQCKAESAVWYPQTDFPACSAY	199036.45

Sequence identifier	Amino acid sequence[a]	MM*
**Alfa-amylase**		
uncharacterized protein [*Centruroides vittatus*]	NERYTDDSQQQKVNEYLAVTLSKCGRKKAPAGGYSSCRYDALGMNCTGTCDKGYYFPGKRSEIILRCRTQNGYWYPRNEFPSCVRD<br>ENNRYTDESQQQALQVTLSRCGRKEAPARGYVNCRYDAFGMNCTGTCVTGYYFPENSAEIILQCRTENGYWYPRNEFPSCAPYDD<br>GGVQTIDNTDDDTSTYTATLIKCRKPVAPEFGVRFCKYDSVVMNCTGTCRTGYYFPDGEREMPLRCRTESGVWYPRADFPACSQFD<br>SERYTNLDATQQREQINRLLAVTLNKCGRKGSPPSGYVNCSYDISGMNCTGTCRSGYYFPGRRAEMILRCRTENGYWYPENEFPACA<br>LHENDKHTDNSQQRPQEVNSQLQITVMKCGRIETPHSGYVNCNYDTLGMNCTGTCKSGYYFPEKRTEIILRCITENGYWYPRNEFPA<br>CRSYENENVEESPSQTQVEEDRLIQETLVRCGRKNQPENGYVNCNYDSSIMNCTGRCITNYYFPTGLREMVLQCNKEYGRWYPENE<br>FPKCRQFIYDKSEENPKPENEDLRVTLDKCGMRKAPIAGYVNCEYNAVEMNCTATCLNGYYFPNGRTEMIIQCKTSSRLWVPRSEFP<br>KCSKDITSHIDTTLKPNQEGENSQQEGENSQFLETLRMCIRRTNPTSGYVYCTHDAVGMNCTGRCRIGEHFPNGGREIVLRCRTDNG<br>LWYPQREFPICSPSTAANLHENQEKDHLDEDNMDNQNQLNQENQDEMTGEKCGKPNPPITGYLSCLNHSHTLECTGTCMPDYAF<br>SVGITSMTLTCSFASGKWSPSNTFTPCYQKNQIPSISNEENEDTGNPTTNILCPKLSTPKGGYIHCEQQFSHGYRRCTGTCMTGLSFPN<br>GENSMVFRCSTVRGWLPASSFPACEENKDRIYQPESEISTNNQGTDLTENQTPNTSENNYGNTGNVDHGNTGTTRSFCRSPPSPKN<br>GYVSCLPNVETLDCTARCSQGFHFPNGQNKLLYRCISKNGRWSPSGYFPDCISACDVPCNNGGVCVGRNQCFCTQDYRGNFCQYPL<br>SNCDPLHLKFAGSIRCNHTSDETVCQLGCPQRMILEPSYSTVRCNLEGIWSPRVSPRCIEDPRYKSVDSNYRLCPTSQCSSSSLTADEG<br>GLCRRPPPPKPGYIRCIYNRLIMECTSKCADGYRYANGQTEQIRTCDNRTGIWTPSHYMSECQPVCERPCQNEGSCMSPNFCFCPVK<br>YRGKFCQYPLSNCDPHRLRFAGSIRCSHSSEETVCKLGCPEGTVLDNPSLDTVRCSLEGYWTPQYVPRCIEANVGGRPTYSNQDYHQ<br>QSNYQQQYEQSQTNEHSLNYEQRMQRQVQNSQIRESSAENGGSAESQRINTDKEKFRDSELEKESYEQRLQMKQEEEQRKLYDRIL<br>KQSSTNRTDQRGNQSRLSDRL	
>TRINITY_DN14063_c0_g1::TRINITY_DN14063_c0_g1_i4::g.13761::m.13761 XP_067123419.1 uncharacterized protein [*Centruroides vittatus*]	>Tcham_Unk_33<br>RKSSRLGYDCNVCQHGGSDVHLETLHVCRRPAPPSPGYISCTYDDKHMKCKGECPSGYKFPDGRNSIEIQCLTKDAVWYPIRSFPNCL<br>PLDYRPYDQPGDQSHDNLDDQSVTLSRCKKPTAPSFGSIRCSYDSENLICTGKCNQGYSFPDGRTEKQLQCNLVRASWYPHGNFPH<br>CYLNKHVGSDDGDSLSVTVRRCRRPPSPAPGTVSCTYDASKMVCVGNCDSGYYFPNGRSEMTLQCNTRDAYWYPLEQFPKCVTYD<br>GIHRQPKSNEGNDEKNIYAVILIRCRKPEAPQAGTSYCSYDTVSMNCTGTCSAGYYFPNGKREMLLQCKAESAVWYPQTDFPACSAY<br>NERYTDDSQQQKVNEYLAVTLSKCGRKKAPAGGYSSCRYDALGMNCTGTCDKGYYFPGKRSEIILRCRTQNGYWYPRNEFPSCVRD<br>ENNRYTDESQQQALQVTLSRCGRKEAPARGYVNCRYDAFGMNCTGTCVTGYYFPENSAEIILQCRTENGYWYPRNEFPSCAPYDD<br>GGVQTIDNTDDDTSTYTATLIKCRKPVAPEFGVRFCKYDSVVMNCTGTCRTGYYFPDGEREMPLRCRTESGVWYPRADFPACSQFD<br>SERYTNLDATQQREQINRLLAVTLNKCGRKGSPPSGYVNCSYDISGMNCTGTCRSGYYFPGRRAEMILRCRTENGYWYPENEFPACA<br>LHENDKHTDNSQQRPQEVNSQLQITVMKCGRIETPHSGYVNCNYDTLGMNCTGTCKSGYYFPEKRTEIILRCITENGYWYPRNEFPA<br>CRSYENENVEESPSQTQVEEDRLIQETLVRCGRKNQPENGYVNCNYDSSIMNCTGRCITNYYFPTGLREMVLQCNKEYGRWYPENE<br>FPKCRQFIYDKSEENPKPENEDLRVTLDKCGMRKAPIAGYVNCEYNAVEMNCTATCLNGYYFPNGRTEMIIQCKTSSRLWVPRSEFP<br>KCSKDITSHIDTTLKPNQEGENSQQEGENSQFLETLRMCIRRTNPTSGYVYCTHDAVGMNCTGRCRIGEHFPNGGREIVLRCRTDNG<br>LWYPQREFPICSPSTAANLHENQEKDHLVCQNLHTPAHGYISCSDDTWSKNKICVARCKLGYSFPNHQFEMTLRCSKEDGKWSLLQ<br>KFPACLPEDEDNMDNQNQLNQENQDEMTGEKCGKPNPPITGYLSCLNHSHTLECTGTCMPDYAFSVGITSMTLTCSFASGKWSPS<br>NTFTPCYQKNQIPSISNEENEDTGNPTTNILCPKLSTPKGGYIHCEQQFSHGYRRCTGTCMTGLSFPNGENSMVFRCSTVRGWLPAS<br>SFPACEENKDRIYQPESEISTNNQGTDLTGQSLISENQTPNTSENNYGNTGNVDHGNTGTTRSFCRSPPSPKNGYVSCLPNVETLDCT	207166.78

Sequence identifier	Amino acid sequence[a]	MM*
**Alfa-amylase**		
	ARCSQGFHFPNGQNKLLYRCISKNGRWSPSGYFPDCISACDVPCNNGGVCVGRNQCFCTQDYRGNFCQYPLSNCDPLHLKFAGSIR<br>CNHTSDETVCQLGCPQRMILEPSYSTVRCNLEGIWSPRVSPRCIEDPRYKSVDSNYRLCPTSQCSSSSLTADEGGLCRRPPPPKPGYIR<br>CIYNRLIMECTSKCADGYRYANGQTEQIRTCDNRTGIWTPSHYMSECQPVCERPCQNEGSCMSPNFCFCPVKYRGKFCQYPLSNCD<br>PHRLRFAGSIRCSHSSEETVCKLGCPEGTVLDNPSLDTVRCSLEGYWTPQYVPRCIEANVGGRPTYSNQDYHQQSNYQQQYEQSQT<br>NEHSLNYEQRMQRQVQNSQIRESSAENGGSAESQRINTDKEKFRDSELEKESYEQRLQMKQEEEQRKLYDRILKQSSTNRTDQRGN<br>QSRLSDRL	
>TRINITY_DN14401_c6_<br>g1::TRINITY_DN14401_c<br>6_g1_i4::g.14745::m.147<br>45 TaPBC_Unk_84	>Tcham_34<br>KVSYEELKALQVKCDPYDTCNTTQDNIEDPLRSCQCDNSCHRYKDCCLDAPNRVTTESSNTICLNMKSSHSYFAISTCKETWKDVDTK<br>RKCERSPNSDDPYSLIPVTNRRSRATYKNRYCAKCNQDEQGLEFWKIGVTCELSNANINFDIQKDLVYNSSLKTWGVRLPDHTFLKCN<br>IEPVAPKNKQHLRPCYSNMISKCLPNWEDDYIREKCQSYTAVILGNRKTKTYRNVHCAICNGVDVRDLDCLYLIPRMQLPGNANFVC<br>LFSVGNCFCENKVRDTAFGICRELTCGLPFLEYKDGKCVKKT	35<br>09<br>9.0<br>1
>TRINITY_DN14063_c0_<br>g1::TRINITY_DN14063_c<br>0_g1_i3::g.13773::m.137<br>73 XP_067123419.1<br>uncharacterized protein<br>[*Centruroides vittatus*]	>Tcham_Unk_35<br>RKSSRLGYDCNVCQHGGSDVHLETLHVCRRPAPPSPGYISCTYDDKHMKCKGECPSGYKFPDGRNSIEIQCLTKDAVWYPIRSFPNCL<br>PLDYRPYDQPGDQSHDNLDDQSVTLSRCKKPTAPSFGSIRCSYDSENLICTGKCNQGYSFPDGRTEKQLQCNLVRASWYPHGNFPH<br>CYLNKHVGSDDGDSLSVTVRRCRRPPSPAPGTVSCTYDASKMVCVGNCDSGYYFPNGRSEMTLQCNTRDAYWYPLEQFPKCVTYD<br>GIHRQPKSNEGNDEKNIYAVILIRCRKPEAPQAGTSYCSYDTVSMNCTGTCSAGYYFPNGKREMLLQCKAESAVWYPQTDFPACSAY<br>NERYTDDSQQQKVNEYLAVTLSKCGRKKAPAGGYSSCRYDALGMNCTGTCDKGYYFPGKRSEIILRCRTQNGYWYPRNEFPSCVRD<br>ENNRYTDESQQQALQVTLSRCGRKEAPARGYVNCRYDAFGMNCTGTCVTGYYFPENSAEIILQCRTENGYWYPRNEFPSCAPYDD<br>GGVQTIDNTDDDTSTYTATLIKCRKPVAPEFGVRFCKYDSVVMNCTGTCRTGYYFPDGEREMPLRCRTESGVWYPRADFPACSQFD<br>SERYTNLDATQQREQINRLLAVTLNKCGRKGSPPSGYVNCSYDISGMNCTGTCRSGYYFPGRRAEMILRCRTENGYWYPENEFPACA<br>LHENDKHTDNSQQRPQEVNSQLQITVMKCGRIETPHSGYVNCNYDTLGMNCTGTCKSGYYFPEKRTEIILRCITENGYWYPRNEFPA<br>CRSYENENVEESPSQTQVEEDRLIQETLVRCGRKNQPENGYVNCNYDSSIMNCTGRCITNYYFPTGLREMVLQCNKEYGRWYPENE<br>FPKCRQFIYDKSEENPKPENEDLRVTLDKCGMRKAPIAGYVNCEYNAVEMNCTATCLNGYYFPNGRTEMIIQCKTSSRLWVPRSEFP<br>KCSKDITSHIDTTLKPNQEGENSQQEGENSQFLETLRMCIRRTNPTSGYVYCTHDAVGMNCTGRCRIGEHFPNGGREIVLRCRTDNG<br>LWYPQREFPICSPSTAANLHENQEKDHLDEDNMDNQNQLNQENQDEMTGEKCGKPNPPITGYLSCLNHSHTLECTGTCMPDYAF<br>SVGITSMTLTCSFASGKWSPSNTFTPCYQKNQIPSISNEENEDTGNPTTNILCPKLSTPKGGYIHCEQQFSHGYRRCTGTCMTGLSFPN<br>GENSMVFRCSTVRGWLPASSFPACEENKDRIYQPESEISTNNQGTDLTGQSLISENQTPNTSENNYGNTGNVDHGNTGTTRSFCRS<br>PPSPKNGYVSCLPNVETLDCTARCSQGFHFPNGQNKLLYRCISKNGRWSPSGYFPDCISACDVPCNNGGVCVGRNQCFCTQDYRG<br>NFCQYPLSNCDPLHLKFAGSIRCNHTSDETVCQLGCPQRMILEPSYSTVRCNLEGIWSPRVSPRCIEDPRYKSVDSNYRLCPTSQCSSS<br>SLTADEGGLCRRPPPPKPGYIRCIYNRLIMECTSKCADGYRYANGQTEQIRTCDNRTGIWTPSHYMSECQPVCERPCQNEGSCMSPN<br>FCFCPVKYRGKFCQYPLSNCDPHRLRFAGSIRCSHSSEETVCKLGCPEGTVLDNPSLDTVRCSLEGYWTPQYVPRCIEANVGGRPTYS<br>NQDYHQQSNYQQQYEQSQTNEHSLNYEQRMQRQVQNSQIRESSAENGGSAESQRINTDKEKFRDSELEKESYEQRLQMKQEEEQ<br>RKLYDRILKQSSTNRTDQRGNQSRLSDRL	19<br>96<br>22.<br>11

Sequence identifier	Amino acid sequence[a]	MM*
**Alfa-amylase**		
>TRINITY_DN12542_c0_g1::TRINITY_DN12542_c0_g1_i2::g.8242::m.8242 TaPBC_Unk_57	>Tcham_Unk_36 GILDIADDLLPVTTNFFREKNGHRMVETIELNTYIGGKKLIDCFLYGDNYIIEKMLEFVPKNLVKTVPKNEMSKLVNKCNALLYRKVREGTFKIIQTPFDFARKIFKSFLIFPGTKWCGAGDVADDYDDLGPAVETDLCCRTHDHCNNSIEGFKTKYDLKNKDFYTKSHCDCDNGFHQCLIDGETLISDAVGHLFFNILQTQCFKEDYPIVKCLKKWGIPIVRDICQEYELDESKPQKYQFFDGKMYQGKHEPSFLKDLLSH	30336.88
>TRINITY_DN11610_c0_g1::TRINITY_DN11610_c0_g1_i1::g.3122::m.3122 Cbi_TRINITY_DN329_c0_g1_i1.p1 XP_023224710.1uncharacterized protein LOC111625725 *[Centruroides sculpturatus]*	>Tcham_Unk_37 SYLGAEYVHTLRRSTKMTVKEKQQRILSLFEHLTSLTEKQLAPADRDPRLASVGILKRGSLFSCFHAKDLDEATKLYEILYHAKDFDDFFLLAKQAHSFVNEGLFVYAVSVALLHRDDCKGVTVPPIQEVFPDRFVPAETVNLAIKEASNNPNSDVVVEVEETGNILDPEYKLAYFREDIGTNAHHWHWHIVYPATWKESDMNKTKDRKGELFYYMHQQMCARYDCERLSNGLNRMIPFHNFDEKLEGYAPHLTSLISGLQYGSRPRGFSLRDLKDVDVQDMERWRERILDAIHLGHIHTSKDEEIALDEAHGADILGDIIESSAESRNPEFYGSLHNWGHVLMARIHDPDGRFNENPGVMSDTSTSLRDPIFYRWHRFVDNIFQDYKATLHSYTREQLDFAGVEVTDVTVNAKKNNTIHTFLKEEELELSHGIQFPSEHSVKVKYHHLDHEPFSYTIDVNNTTGAAKHATVRIFLGPKFDELGNILDVNEQRRLYIELDKFHTNLTPGKNTISRTAIESSVTVSEKHTFDQLKKGEGVSEDATEFCSCGWPEHMLVPRGNHRGMIFELFVMLTNADEDHVGAGGKLLCQDAISYCGAKDAIYPDKKPMGFPFDRPIHARNLREFITPNVHISEIDIKFDG	73464.65
>TRINITY_DN12001_c0_g1::TRINITY_DN12001_c0_g1_i4::g.39724::m.39724 Tja_Unk_05	>Tcham_Unk_38 LYVNYNIRNFEDIFKRQICHDLGSSCSGSRQDCCDPYCCSFGECSVSC	5467.10
>TRINITY_DN14185_c0_g2::TRINITY_DN14185_c0_g2_i2::g.73852::m.73852 No Match	>Tcham_Unk_39 KLIRCSVSCKQRKIDECLRSGKGCETLDNEEICTEKCEGLEMFDKKLKRCSEPCSNEKMKECEGTGKNCEILDNVETCTEKCEGKERFDKKLKRCLEPCSDKKITECEGTGKNCEILDNVETCTEKCGGKERFDRKLIRCSVSCKQRKIDECLRSGKGCE	18237.03
>TRINITY_DN14553_c2_g1::TRINITY_DN14553_c2_g1_i5::g.47420::m.47420 Tja_Unk_57	>Tcham_Unk_40 IDSFIDNYCGHFDAVKFYDCLYNTAAKETVRKFNKCASEQNIPTDSKERRDFICDRNNADQLRQGYTCLFGIMNSEEVIDFHKTVNTCVNQ	10588.78
>TRINITY_DN14128_c0_g3::TRINITY_DN14128_c0_g3_i1::g.75894::m.75894 Tja_Unk_63	>Tcham_Unk_41 EDSLYTEYCGIKDIKKFYVCLKESVSKKTWGTFIQCANKENIPIDPVEQKKFICNRSNVLAILNLYQCLLKNLNPSEIQELTEHNEKCLNV	10587.26
>TRINITY_DN14375_c2_g1::TRINITY_DN14375_c2_g1_i7::g.5587::m.5587 No Match	>Tcham_Unk_42 KDPRYLPTLFLNVILSALEDVHNSTESVSDDNSRSNLKLSDTTRKLPPYGVIRNK	6229.01

Sequence identifier	Amino acid sequence[a]	MM*
**Alfa-amylase**		
>TRINITY_DN14090_c1_g1::TRINITY_DN14090_c1_g1_i8::g.14715::m.14715 Tja_Unk_57	>Tcham_Unk_43 IDSFIDNYCGHFDAVKFYDCLYNTAAKETVRKFNKCASEQNIPTDSKERRDFICDRNNADQLRQGYTCLFGIMNSEEVIDFHKTVNTCMNQ	10620.84
>TRINITY_DN14418_c1_g3::TRINITY_DN14418_c1_g3_i4::g.18271::m.18271 Tja_Unk_59	>Tcham_Unk_44 EETSVHDRYCSLNPIEKYYDCMKKNAPEKTWNTFNGCINKLNLPSDFNEQRKFFCDAAKDEETRKWYFCVMDELNTSELKKFHEYVEICLK	11006.43
>TRINITY_DN14420_c1_g1::TRINITY_DN14420_c1_g1_i17::g.16460::m.16460 No Match	>Tcham_Unk_45 KLIRCSVSCKQRKIDECLRSGKGCETLDNEEICTEKCEGLEMFDKKLKRCSEPCSNKKITECEGTGKNCEILDNVETCTEKCEGKERFDKKLKRCLEPCSDKKITECEGTGKNCEILDNVETCTEKCGGKERFDRKLIRCSVSCKQRKIDECLRSGKGCE	18190.99
>TRINITY_DN14463_c1_g1::TRINITY_DN14463_c1_g1_i2::g.16865::m.16865 TaPBC_Unk_86	>Tcham_Unk_46 DKACSECPPGMGCDSHYKRLCAPIAKTTVPATTAKTTTVNDEILWKCDFSIGTKKQCKSEMQCYANWKTVCGLNSCHKEIILNKQKSSLLFLTPILVKNQACLIFQYKKELLPAAQGESNLTAVAVWN	14160.56
>TRINITY_DN37398_c0_g1::TRINITY_DN37398_c0_g1_i1::g.32221::m.32221 XP_023213360.1 uncharacterized protein LOC111616173 *[Centruroides sculpturatus]*	>Tcham_Unk_47 GKGICWNPCIHTPDNNIWHSKAEIVPYLEESLKFEGKHTGNTLEAIKYATTILPFDM	6473.40
>TRINITY_DN14571_c0_g1::TRINITY_DN14571_c0_g1_i7::g.48228::m.48228 No Match	>Tcham_Unk_48 FSLRKEQIKIYYCNSEILKMNSQRFATFFIVIISFSAFSIARYLPTLFLNVILSALEDVHNSTESVSDDNSRSNLKLSDTTRKLPPYGVIRNK	10726.35
>TRINITY_DN14375_c2_g1::TRINITY_DN14375_c2_g1_i6::g.5592::m.5592 No Match	>Tcham_Unk_49 FSLRKQKKKIYYCNSEILKMNSQRFATFFIVIISFSAFSIARYLPTLFLNVILSALEDVHNSTESVSDDNSRSNLKLSDTTRKLPPYGVIRNK	10740.4
>TRINITY_DN14375_c2_g1::TRINITY_DN14375_c	>Tcham_Unk_50 FSLRKQQIKIYYCNSEILKMNSQRFATFFIVIISFSAFSIARYLPTLFLNVILSALEDVHNSTESVSDDNSRSNLKLSDTTRKLPPYGVIRNK	1072

Sequence identifier	Amino acid sequence[a]	M<br>M*
**Alfa-amylase**		
2_g1_i2::g.5593::m.5593<br>No Match		5.3<br>7
>TRINITY_DN14202_c0_<br>g1::TRINITY_DN14202_c<br>0_g1_i2::g.62519::m.625<br>19 XP_023210948.1<br>uncharacterized protein<br>LOC111613829<br>*[Centruroides<br>sculpturatus]*	>Tcham_Unk_51<br>MDKSILQGYLLLKLSGGLSILKPPRKLWLVYQEEKCQLFCYKNEFESVNVYKAPLDVIKLQKAAITPHAEENNHFIIYSDQREYHLFADD<br>YESMMRWIFELQKRRDEWNNQFNTVPIQTDYEKENIENRDEILPNKEEMQIVIQNTKRAEERSENRGNFLSEYSLSQTFGVYKLNND<br>DVISDERQRTNPTPEDPTQTIPKELEESNISCYYPVEECDGSISSDSAVCTSEYHNAKIQEMEAEMRMTHKILSEALSREAAYKSTMERL<br>EDLVEELEDRMSRVQGGIGFWRYLSGDKTLVNFRKLEEKCQILKNHNVFLNDEILKLIRMLQQEQFRFQAKESQIQDIKNQIEQMKR<br>DYIFLFLSSIRIYYGDGPETTEVYLYGGSRHKVRVLSLLEEARKINKDLPTYESLIKPCYVDPMGFEHCFTEESVVLHYICSLLYTHYSKQLR<br>SYEKHVNSWNKYCKQEEKLSIKKEVKFLVRKGIPDYLRKTIWRQFYRDKVHDVMKDKGPYYYKTLVNTVTESQIILYHNRQIALDLLRT<br>MPNNIRFSTPKSEGVNKLEEVLLAFCLHNPYLGYCQGMNFLVGMCLLIFEPEDAFWCLVAITEKFFPSTYFDQSLIGAQADQEVLKDL<br>MKEKLPNLYRHLSSLDIDLRTVTLNWYMSIFFDCVPFEVAFEDFHPFPRRSDLATKQTYYAKMLKDSSKKRERRRKSFIDMLPEMMID<br>WKLSATNRPCIDCATVYDNNKLWLAYCHTNGSQIVKINVEDCIMYKLNIECESRIICMHSATSDILLLGTVSNVLQVYNNKTGRLLSEV<br>KLNDSVLCLHYHNKEEANVIYAGLADGTLAVIESIYEIEKKKEVFYISIGSSPVSCLLELDWRLLCACGNRIFILNSRTLDTLHQLQITCND<br>MDHISNLMLDGNNIWTSVRGSSILHVWDSHTLACKLIFDIKHNRPTCHASKEDDSFLQSMNITNILPIDDGVIVGTSDGSLLIYDVEKK<br>MTRSISCTESSIYESVGENIEEDSEFANHPTQNYKFIDKSSDVSLPAAITEYDIDHELTISLQPRGESAPPRLGESVQISRELSTNSLILELEN<br>NKNKPTRLDDVENNSISSVDLRQACGFYAVEDNKTDLAECKSLLLANKSNHIVEGKEISFQPVNLTPAVSLVSFNSTDYLYCLELVIKEKI<br>KIGDKPIKCLLNVKCNNEINFVSCTGLYGEEDSVMKWSKEGKMLWTGKQILDICPITPKQSSLSDQKLFSNSMVGAAGSSFIKTRKKS<br>MTEKIELSGINKVQTLFFKLPGK	14<br>78<br>58.<br>07
>TRINITY_DN14556_c2_<br>g2::TRINITY_DN14556_c<br>2_g2_i1::g.47470::m.474<br>70 Tja_Unk_148	>Tcham_Unk_52<br>AAVRQSARSRRALVEVDEPEKTLQVLGNLADRILHAEEMESLTSNEKRGLDLGLSRGFSGSQAAKHLMGLAAASFANGPGRRRREV<br>PGQN	97<br>08.<br>92
>TRINITY_DN14543_c1_<br>g3::TRINITY_DN14543_c<br>1_g3_i1::g.48036::m.480<br>36 Tja_Unk_01	>Tcham_Unk_53<br>GQKNCYSKKDCETVCMVVKSCQYGTCYCKGNGKRQLS	41<br>41.<br>81
>TRINITY_DN14304_c6_<br>g7::TRINITY_DN14304_c<br>6_g7_i1::g.4915::m.4915<br>Tja_Unk_04	>Tcham_Unk_54<br>ADRCFIISCAIKCNKRREEVMACSKSKDCSCSI	36<br>99.<br>39

MM* means the calculated molecular average mass of the peptide/protein.

[a]Letters in bold represent post-translational amino acid modifications that are recognition sites for C-terminal amidation
