# Supplementary material for: Proteomic characterization of Tityus championi venom and recombinant expression of its major neurotoxin
Source: J Venom Anim Toxins Incl Trop Dis. 2026 Sep 14;32:e20260024. doi: 10.1590/1678-9199-JVATITD-2026-0024 (PMC13574325; doi:10.1590/1678-9199-JVATITD-2026-0024)
Supplement: Supplementary file 5 [file 1678-9199-jvatitd-32-e20260024-s5.pdf]

J Venom Anim Toxins incl Trop Dis,
https://doi.org/10.1590/1678-9199-JVATITD-2026-0024

Supplementary Material to: Proteomic characterization of *Tityus championi* venom and recombinant expression of its major neurotoxin

Additional file 5. Protein groups identified in the venom of *T. championi* by DIA-NN and classified into protein families based on homology.

Protein Group	Protein Names	Genes	N. Sequences	N. Proteotypic sequences	G:\Compartida\championi\GC_4_2_Tityus_championi_tryp_1.raw	G:\Compartida\championi\GC_5_2_Tityus_championi_tryp_2.raw	G:\Compartida\championi\GC_6_2_Tityus_championi_tryp_3.raw	G:\Compartida\championi\GC_16_2_Tityus_championi_cht_1.raw	G:\Compartida\championi\GC_17_2_Tityus_championi_cht_2.raw
TRINITY_DN14578_c6_g1::TRINITY_DN14578_c6_g1_i1::g.46841::m.46841; TRINITY_DN14578_c6_g1::TRINITY_DN14578_c6_g1_i3::g.46850::m.46850	XP_067126965.1	XP_067126965.1	32	32	4.65E+06	4.73E+06	4.65E+06	4.81E+06	2.78E+06
TRINITY_DN13740_c0_g1::TRINITY_DN13740_c0_g1_i1::g.71239::m.71239	XP_067126753.1	XP_067126753.1	23	23	2.15E+07	2.20E+07	2.16E+07	2.19E+07	2.11E+07
TRINITY_DN14075_c1_g2::TRINITY_DN14075_c1_g2_i6::g.16841::m.16841	metalloasthenase01	metalloasthenase01	22	9	7.33E+08	7.76E+08	7.61E+08	5.48E+08	4.76E+08
TRINITY_DN13685_c0_g1::TRINITY_DN13685_c0_g1_i1::g.28779::m.28779; TRINITY_DN13870_c0_g1::TRINITY_DN13870_c0_g1_i2::g.10108::m.10108	Tja_AA01	Tja_AA01	22	22	7.46E+07	7.21E+07	8.50E+07	2.87E+07	3.37E+07
TRINITY_DN14507_c0_g1::TRINITY_DN14507_c0_g1_i5::g.48408::m.48408	XP_067121543.1	XP_067121543.1	22	4	3.46E+07	3.42E+07	3.33E+07	6.68E+06	6.60E+06
TRINITY_DN13980_c0_g1::TRINITY_DN13980_c0_g1_i3::g.70110::m.70110	Cbi_TRINITY_DN3945_c0_g2_i1.p1	21	21		2.10E+07	2.22E+07	2.19E+07	963219	811025
TRINITY_DN14401_c6_g1::TRINITY_DN14401_c6_g1_i4::g.14745::m.14745	TaPBC_Unk_84	TaPBC_Unk_84	20	20	4.97E+07	5.07E+07	5.07E+07	3.89E+06	4.06E+06
TRINITY_DN13590_c0_g2::TRINITY_DN13590_c0_g2_i1::g.56898::m.56898; TRINITY_DN14001_c0_g1::TRINITY_DN14001_c0_g1_i1::g.14237::m.14237	TaPBP_CRISP02	TaPBP_CRISP02	20	20	1.48E+08	1.56E+08	1.52E+08	1.46E+08	1.29E+08
TRINITY_DN13735_c0_g1::TRINITY_DN13735_c0_g1_i1::g.71015::m.71015	Cbi_TRINITY_DN37835_c0_g1_i1.p1	19	19		3.78E+06	3.71E+06	3.71E+06	2.35E+06	2.51E+06
TRINITY_DN13885_c0_g1::TRINITY_DN13885_c0_g1_i1::g.10008::m.10008	Tja_Hyal01	Tja_Hyal01	19	19	1.50E+08	1.48E+08	1.55E+08	5.19E+07	4.49E+07
TRINITY_DN12815_c0_g3::TRINITY_DN12815_c0_g3_i3::g.70355::m.70355; TRINITY_DN13624_c2_g1::TRINITY_DN13624_c2_g1_i2::g.28246::m.28246	Tja_PHM01	Tja_PHM01	18	18	2.18E+07	2.44E+07	1.95E+07	4.78E+07	4.40E+07
TRINITY_DN14269_c0_g1::TRINITY_DN14269_c0_g1_i1::g.65444::m.65444	Tja_CRISP02	Tja_CRISP02	17	17	7.09E+07	6.51E+07	7.18E+07	1.29E+07	1.27E+07

Protein Group	Protein Names	Genes	N. Sequences	N. Proteotypic sequences	G:\Compartida\championi\GC_4_2_Tityus_championi_tryp_1.raw	G:\Compartida\championi\GC_5_2_Tityus_championi_tryp_2.raw	G:\Compartida\championi\GC_6_2_Tityus_championi_tryp_3.raw	G:\Compartida\championi\GC_16_2_Tityus_championi_cht_1.raw	G:\Compartida\championi\GC_17_2_Tityus_championi_cht_2.raw
TRINITY_DN13641_c0_g1::TRINITY_DN13641_c0_g1_i1::g.29946::m.29946	XP_067131550.1	XP_067131550.1	17	17	2.27E+06	2.23E+06	2.20E+06		
TRINITY_DN14368_c0_g1::TRINITY_DN14368_c0_g1_i5::g.7133::m.7133; TRINITY_DN14602_c0_g2::TRINITY_DN14602_c0_g2_i7::g.60863::m.60863	Metallojaimease127	Metallojaimease127	15	1	1.11E+08	1.17E+08	1.18E+08	3.34E+07	3.25E+07
TRINITY_DN14297_c0_g2::TRINITY_DN14297_c0_g2_i5::g.59942::m.59942	Metallojaimease03	Metallojaimease03	14	2	1.45E+08	1.48E+08	1.48E+08	6.44E+07	3.99E+07
TRINITY_DN14529_c0_g2::TRINITY_DN14529_c0_g2_i3::g.45629::m.45629	XP_023242599.1	XP_023242599.1	14	3	550894	561776	556394		
TRINITY_DN14413_c1_g2::TRINITY_DN14413_c1_g2_i3::g.15175::m.15175; TRINITY_DN14413_c1_g2::TRINITY_DN14413_c1_g2_i6::g.15190::m.15190	metalloasthenase19	metalloasthenase19	13	13	5.43E+07	5.41E+07	5.06E+07	3.08E+07	3.17E+07
TRINITY_DN14589_c0_g1::TRINITY_DN14589_c0_g1_i3::g.48611::m.48611	TaPBC_Lectin_16	TaPBC_Lectin_16	13	3	1.87E+08	1.94E+08	2.07E+08	6.98E+07	3.08E+07
TRINITY_DN13965_c0_g1::TRINITY_DN13965_c0_g1_i1::g.69707::m.69707	Tja_NPP01	Tja_NPP01	13	12	3.28E+07	3.34E+07	3.36E+07	3.63E+07	3.51E+07
TRINITY_DN14319_c2_g3::TRINITY_DN14319_c2_g3_i1::g.6831::m.6831; TRINITY_DN14319_c2_g3::TRINITY_DN14319_c2_g3_i4::g.6839::m.6839; TRINITY_DN14500_c3_g1::TRINITY_DN14500_c3_g1_i2::g.44839::m.44839; TRINITY_DN14500_c3_g1::TRINITY_DN14500_c3_g1_i3::g.44843::m.44843	metalloasthenase129	metalloasthenase129	12	8	9.45E+06	9.35E+06	9.36E+06	7.97E+06	6.60E+06
TRINITY_DN14257_c2_g3::TRINITY_DN14257_c2_g3_i2::g.61252::m.61252	Tja_CIIMET_04	Tja_CIIMET_04	12	9	5.95E+08	5.37E+08	5.54E+08	5.71E+08	7.31E+08
TRINITY_DN14405_c0_g1::TRINITY_DN14405_c0_g1_i1::g.14673::m.14673	Tja_SP04	Tja_SP04	12	3	9.84E+07	9.62E+07	9.77E+07	9.21E+07	9.28E+07
TRINITY_DN14628_c5_g1::TRINITY_DN14628_c5_g1_i6::g.59198::m.59198	XP_067145024.1	XP_067145024.1	12	3	2.65E+06	2.76E+06	2.54E+06		
TRINITY_DN14567_c2_g1::TRINITY_DN14567_c2_g1_i14::g.48267::m.48267; TRINITY_DN14567_c2_g1::TRINITY_DN14567_c2_g1_i16::g.48259::m.48259	metalloasthenase25	metalloasthenase25	11	11	4.57E+07	4.73E+07	4.63E+07	4.68E+07	4.54E+07
TRINITY_DN14326_c0_g6::TRINITY_DN14326_c0_g6_i4::g.4424::m.4424	Metallojaimease109	Metallojaimease109	11	2	1.27E+08	1.41E+08	1.43E+08	1.20E+08	1.06E+08
TRINITY_DN14615_c1_g2::TRINITY_DN14615_c1_g2_i2::g.60939::m.60939	Tja_CIIMET_03	Tja_CIIMET_03	11	4	2.51E+09	2.75E+09	2.16E+09	1.50E+09	1.53E+09
TRINITY_DN14025_c0_g1::TRINITY_DN14025_c0_g1_i1::g.13804::m.13804	XP_023234347.1	XP_023234347.1	11	11	7.04E+06	7.48E+06	7.29E+06		
TRINITY_DN14306_c9_g2::TRINITY_DN14306_c9_g2_i3::g.5577::m.5577; TRINITY_DN14306_c9_g2::TRINITY_DN14306_c9_g2_i4::g.5584::m.5584	XP_067139465.1;XP_067139466.1	11	0		2.82E+06	3.04E+06	3.00E+06		
TRINITY_DN14567_c2_g1::TRINITY_DN14567_c2_g1_i11::g.48264::m.48264	metalloasthenase13	metalloasthenase13	10	10	3.64E+07	3.94E+07	3.74E+07	1.02E+07	1.00E+07

Protein Group	Protein Names	Genes	N. Sequences	N. Proteotypic sequences	G:\Compartida\championi\GC_4_2_Tityus_championi_tryp_1.raw	G:\Compartida\championi\GC_5_2_Tityus_championi_tryp_2.raw	G:\Compartida\championi\GC_6_2_Tityus_championi_tryp_3.raw	G:\Compartida\championi\GC_16_2_Tityus_championi_cht_1.raw	G:\Compartida\championi\GC_17_2_Tityus_championi_cht_2.raw
TRINITY_DN12002_c0_g1::TRINITY_DN12002_c0_g1_i1::g.40028::m.40028	XP_067124461.1	XP_067124461.1	10	10	4.49E+06	4.90E+06	4.94E+06	994916	984836
TRINITY_DN13939_c0_g1::TRINITY_DN13939_c0_g1_i3::g.70294::m.70294	XP_067144653.1	XP_067144653.1	10	10	3.30E+06	3.55E+06	3.64E+06	3.00E+06	2.68E+06
TRINITY_DN14208_c2_g2::TRINITY_DN14208_c2_g2_i1::g.66459::m.66459	metalloasthenase08	metalloasthenase08	9	9	2.17E+07	2.09E+07	2.06E+07	4.54E+06	6.51E+06
TRINITY_DN14406_c1_g2::TRINITY_DN14406_c1_g2_i20::g.18700::m.18700	metalloasthenase109	metalloasthenase109	9	1	1.18E+07	1.25E+07	1.24E+07	1.05E+07	8.45E+06
TRINITY_DN14351_c5_g1::TRINITY_DN14351_c5_g1_i7::g.4648::m.4648	Tja_Unk_51	Tja_Unk_51	9	5	1.68E+09	1.82E+09	2.46E+09	6.59E+08	5.77E+08
TRINITY_DN12570_c0_g1::TRINITY_DN12570_c0_g1_i3::g.8035::m.8035; TRINITY_DN12972_c0_g1::TRINITY_DN12972_c0_g1_i1::g.30308::m.30308	XP_023215218.1;XP_067136263.1	9	0		4.49E+06	4.70E+06	4.38E+06		
TRINITY_DN14011_c0_g1::TRINITY_DN14011_c0_g1_i2::g.13144::m.13144	XP_023220268.1	XP_023220268.1	9	9	2.35E+06	2.43E+06	2.59E+06		
TRINITY_DN14258_c0_g1::TRINITY_DN14258_c0_g1_i2::g.61568::m.61568; TRINITY_DN14258_c0_g1::TRINITY_DN14258_c0_g1_i9::g.61574::m.61574; TRINITY_DN14528_c8_g2::TRINITY_DN14528_c8_g2_i3::g.47183::m.47183	XP_067140417.1	XP_067140417.1	9	9	1.49E+06	1.66E+06	1.61E+06	484652	542924
TRINITY_DN14444_c2_g3::TRINITY_DN14444_c2_g3_i7::g.18296::m.18296; TRINITY_DN14444_c2_g3::TRINITY_DN14444_c2_g3_i4::g.18304::m.18304	metalloasthenase05	metalloasthenase05	8	6	2.24E+07	2.35E+07	2.34E+07	5.27E+06	3.83E+06
TRINITY_DN14447_c1_g2::TRINITY_DN14447_c1_g2_i5::g.18167::m.18167	TaPBC_Unk_90	TaPBC_Unk_90	8	0	1.97E+07	2.16E+07	2.11E+07	2.04E+07	1.98E+07
TRINITY_DN14447_c1_g2::TRINITY_DN14447_c1_g2_i1::g.18177::m.18177	Tja_CRISP18	Tja_CRISP18	8	2	3.30E+07	3.72E+07	3.68E+07	8.09E+06	8.90E+06
TRINITY_DN13324_c0_g1::TRINITY_DN13324_c0_g1_i1::g.49375::m.49375	Tja_NaTx03	Tja_NaTx03	8	8	1.28E+08	1.05E+08	1.27E+08	5.21E+08	4.65E+08
TRINITY_DN12913_c0_g1::TRINITY_DN12913_c0_g1_i1::g.30036::m.30036	Tja_NaTx32	Tja_NaTx32	8	7	8.44E+07	8.07E+07	7.81E+07	6.76E+06	1.19E+07
TRINITY_DN14624_c0_g1::TRINITY_DN14624_c0_g1_i2::g.61100::m.61100	Tja_SP10	Tja_SP10	8	7	5.01E+06	5.79E+06	6.08E+06	3.83E+06	3.18E+06
TRINITY_DN13934_c1_g1::TRINITY_DN13934_c1_g1_i2::g.67165::m.67165	Tppa2	Tppa2	8	0	1.67E+08	1.65E+08	1.55E+08	8.83E+07	8.80E+07
TRINITY_DN13878_c0_g1::TRINITY_DN13878_c0_g1_i1::g.9605::m.9605; TRINITY_DN13878_c0_g1::TRINITY_DN13878_c0_g1_i2::g.9615::m.9615	XP_067137235.1	XP_067137235.1	8	8	607581	614232	633838		
TRINITY_DN14586_c1_g1::TRINITY_DN14586_c1_g1_i2::g.47219::m.47219	metalloasthenase109	metalloasthenase109	7	1	1.67E+08	1.70E+08	1.64E+08		
TRINITY_DN14295_c2_g2::TRINITY_DN14295_c2_g2_i2::g.62101::m.62101	metalloasthenase110	metalloasthenase110	7	6	5.54E+08	5.17E+08	5.10E+08	520708	421438

Protein Group	Protein Names	Genes	N. Sequences	N. Proteotypic sequences	G:\Compartida\championi\GC_4_2_Tityus_championi_tryp_1.raw	G:\Compartida\championi\GC_5_2_Tityus_championi_tryp_2.raw	G:\Compartida\championi\GC_6_2_Tityus_championi_tryp_3.raw	G:\Compartida\championi\GC_16_2_Tityus_championi_cht_1.raw	G:\Compartida\championi\GC_17_2_Tityus_championi_cht_2.raw
TRINITY_DN14639_c1_g2::TRINITY_DN14639_c1_g2_i5::g.59357::m.59357	metalloasthenase116	metalloasthenase116	7	0	1.47E+07	1.44E+07	1.46E+07		
TRINITY_DN14199_c0_g2::TRINITY_DN14199_c0_g2_i2::g.74071::m.74071	Metallojaimease80	Metallojaimease80	7	0	5.82E+06	5.97E+06	5.88E+06	3.57E+06	3.30E+06
TRINITY_DN12542_c0_g1::TRINITY_DN12542_c0_g1_i2::g.8242::m.8242	TaPBC_Unk_57	TaPBC_Unk_57	7	7	1.14E+07	1.14E+07	1.12E+07		
TRINITY_DN14382_c0_g1::TRINITY_DN14382_c0_g1_i5::g.5192::m.5192	Tja_NPP05	Tja_NPP05	7	6	402959	442537	443085		
TRINITY_DN10660_c0_g1::TRINITY_DN10660_c0_g1_i1::g.34473::m.34473	XP_023218154.1	XP_023218154.1	7	7	5.52E+06	5.38E+06	5.30E+06	5.61E+06	5.44E+06
TRINITY_DN14409_c0_g1::TRINITY_DN14409_c0_g1_i2::g.14767::m.14767; TRINITY_DN14409_c0_g1::TRINITY_DN14409_c0_g1_i3::g.14771::m.14771	XP_023218575.1;XP_067130270.1	7	0		1.18E+06	1.11E+06	1.11E+06		
TRINITY_DN13438_c0_g1::TRINITY_DN13438_c0_g1_i3::g.22841::m.22841	XP_023241316.1	XP_023241316.1	7	2	1.49E+06	1.42E+06	1.40E+06		
TRINITY_DN11671_c0_g1::TRINITY_DN11671_c0_g1_i1::g.3144::m.3144	XP_067121879.1	XP_067121879.1	7	7	964199	945781	961273	985311	954107
TRINITY_DN14115_c5_g4::TRINITY_DN14115_c5_g4_i1::g.74075::m.74075; TRINITY_DN14115_c5_g4::TRINITY_DN14115_c5_g4_i2::g.74085::m.74085	XP_067137151.1	XP_067137151.1	7	7	1.79E+06	1.60E+06	1.60E+06		
TRINITY_DN14311_c0_g1::TRINITY_DN14311_c0_g1_i1::g.5231::m.5231	XP_067138118.1	XP_067138118.1	7	7	550800	553523	513891		
TRINITY_DN12059_c0_g1::TRINITY_DN12059_c0_g1_i1::g.39709::m.39709	XP_067143319.1	XP_067143319.1	7	7	2.16E+06	2.17E+06	2.03E+06		
TRINITY_DN14326_c0_g1::TRINITY_DN14326_c0_g1_i7::g.4390::m.4390; TRINITY_DN14567_c2_g7::TRINITY_DN14567_c2_g7_i2::g.48276::m.48276	metalloasthenase26	metalloasthenase26	6	6	4.20E+06	4.60E+06	4.45E+06		
TRINITY_DN14208_c2_g1::TRINITY_DN14208_c2_g1_i3::g.66451::m.66451	Metallojaimease20	Metallojaimease20	6	6	4.51E+06	4.78E+06	4.76E+06		
TRINITY_DN14421_c1_g3::TRINITY_DN14421_c1_g3_i2::g.18660::m.18660; TRINITY_DN14638_c0_g3::TRINITY_DN14638_c0_g3_i4::g.59906::m.59906	Metallojaimease32	Metallojaimease32	6	0	1.90E+07	1.86E+07	1.77E+07		
TRINITY_DN14500_c3_g1::TRINITY_DN14500_c3_g1_i4::g.44849::m.44849	Metallojaimease55	Metallojaimease55	6	3	5.42E+06	5.97E+06	5.45E+06	446839	483705
TRINITY_DN14185_c0_g2::TRINITY_DN14185_c0_g2_i2::g.73852::m.73852; TRINITY_DN14185_c0_g2::TRINITY_DN14185_c0_g2_i4::g.73858::m.73858; TRINITY_DN14420_c1_g1::TRINITY_DN14420_c1_g1_i17::g.16460::m.16460	No	No	6	6	2.77E+08	2.45E+08	2.38E+08	1.85E+08	1.62E+08

Protein Group	Protein Names	Genes	N. Sequences	N. Proteotypic sequences	G:\Compartida\championi\GC_4_2_Tityus_championi_tryp_1.raw	G:\Compartida\championi\GC_5_2_Tityus_championi_tryp_2.raw	G:\Compartida\championi\GC_6_2_Tityus_championi_tryp_3.raw	G:\Compartida\championi\GC_16_2_Tityus_championi_cht_1.raw	G:\Compartida\championi\GC_17_2_Tityus_championi_cht_2.raw
TRINITY_DN14589_c0_g1::TRINITY_DN14589_c0_g1_i5::g.48616::m.48616	TaPBC_Lectin_16	TaPBC_Lectin_16	6	2	6.83E+07	6.18E+07	6.46E+07	6.36E+07	7.04E+07
TRINITY_DN14489_c2_g1::TRINITY_DN14489_c2_g1_i2::g.16650::m.16650	TaPBC_SIGF_06	TaPBC_SIGF_06	6	6	9.36E+08	9.20E+08	9.10E+08	2.84E+06	2.23E+06
TRINITY_DN14227_c6_g3::TRINITY_DN14227_c6_g3_i1::g.66387::m.66387	TaPBC_Unk_03	TaPBC_Unk_03	6	6	4.76E+08	4.39E+08	4.67E+08	2.06E+06	1.70E+06
TRINITY_DN14565_c3_g1::TRINITY_DN14565_c3_g1_i2::g.46136::m.46136; TRINITY_DN14565_c3_g1::TRINITY_DN14565_c3_g1_i3::g.46143::m.46143	TaPBC_Unk_06;Tja_PK_01	TaPBC_Unk_06;Tja_PK_01	6	0	7.66E+06	8.37E+06	7.10E+06	2.25E+07	2.46E+07
TRINITY_DN14128_c0_g1::TRINITY_DN14128_c0_g1_i3::g.75886::m.75886	TaPBC_Unk_54	TaPBC_Unk_54	6	4	6.24E+07	8.10E+07	8.07E+07	7.38E+06	5.88E+06
TRINITY_DN14187_c9_g1::TRINITY_DN14187_c9_g1_i2::g.75299::m.75299; TRINITY_DN14187_c9_g1::TRINITY_DN14187_c9_g1_i4::g.75296::m.75296	TaPBP-KTx_01	TaPBP-KTx_01	6	6	2.39E+09	2.41E+09	2.40E+09	3.35E+07	4.64E+07
TRINITY_DN14392_c0_g4::TRINITY_DN14392_c0_g4_i2::g.3856::m.3856	TaPBP-KTx_04	TaPBP-KTx_04	6	6	9.67E+08	7.60E+08	7.71E+08	5.01E+08	5.16E+08
TRINITY_DN14084_c7_g1::TRINITY_DN14084_c7_g1_i5::g.13702::m.13702	TaPBP-NaTx31	TaPBP-NaTx31	6	4	1.06E+08	1.11E+08	8.69E+07	1.68E+08	1.43E+08
TRINITY_DN14264_c2_g2::TRINITY_DN14264_c2_g2_i1::g.64274::m.64274	Tja_NaTx16	Tja_NaTx16	6	5	3.18E+08	3.19E+08	3.18E+08	2.19E+08	2.09E+08
TRINITY_DN12351_c0_g1::TRINITY_DN12351_c0_g1_i1::g.43835::m.43835	Tja_NPP07	Tja_NPP07	6	6	2.37E+06	2.36E+06	2.33E+06		
TRINITY_DN652_c0_g1::TRINITY_DN652_c0_g1_i1::g.68126::m.68126	Tja_NPP08	Tja_NPP08	6	6	834103	902383	860799	812068	789039
TRINITY_DN14274_c8_g3::TRINITY_DN14274_c8_g3_i2::g.65880::m.65880	Tja_SIGF01	Tja_SIGF01	6	6	1.99E+07	2.00E+07	1.95E+07	1.73E+06	1.46E+06
TRINITY_DN14418_c1_g3::TRINITY_DN14418_c1_g3_i2::g.18270::m.18270	Tja_Unk_59	Tja_Unk_59	6	6	1.33E+07	1.45E+07	1.40E+07	3.97E+06	3.66E+06
TRINITY_DN43758_c0_g1::TRINITY_DN43758_c0_g1_i1::g.33831::m.33831	XP_023231107.1	XP_023231107.1	6	6	1.34E+06	1.28E+06	1.26E+06		
TRINITY_DN14684_c7_g7::TRINITY_DN14684_c7_g7_i1::g.61693::m.61693	XP_023242780.1	XP_023242780.1	6	6	2.78E+06	2.88E+06	2.84E+06	2.82E+06	3.21E+06
TRINITY_DN12937_c0_g1::TRINITY_DN12937_c0_g1_i1::g.30231::m.30231	XP_067138356.1	XP_067138356.1	6	6	398262	419749	396328		
TRINITY_DN13742_c0_g1::TRINITY_DN13742_c0_g1_i2::g.70668::m.70668	XP_067142793.1	XP_067142793.1	6	6	421903	416575	388431		
TRINITY_DN14567_c2_g1::TRINITY_DN14567_c2_g1_i12::g.48236::m.48236; TRINITY_DN14567_c2_g1::TRINITY_DN14567_c2_g1_i1::g.48269::m.48269; TRINITY_DN14567_c2_g1::TRINITY_DN14567_c2_g1_i5::g.48257::m.48257	metalloasthenase03	metalloasthenase03	5	5	4.06E+07	4.32E+07	4.07E+07		

Protein Group	Protein Names	Genes	N. Sequences	N. Proteotypic sequences	G:\Compartida\championi\GC_4_2_Tityus_championi_tryp_1.raw	G:\Compartida\championi\GC_5_2_Tityus_championi_tryp_2.raw	G:\Compartida\championi\GC_6_2_Tityus_championi_tryp_3.raw	G:\Compartida\championi\GC_16_2_Tityus_championi_cht_1.raw	G:\Compartida\championi\GC_17_2_Tityus_championi_cht_2.raw
TRINITY_DN14523_c1_g1::TRINITY_DN14523_c1_g1_i5::g.47368::m.47368	Metallojaimease84	Metallojaimease84	5	2	1.19E+07	1.12E+07	1.10E+07		
TRINITY_DN14185_c0_g1::TRINITY_DN14185_c0_g1_i11::g.73850::m.73850	No	No	5	3	1.33E+07	1.58E+07	1.55E+07		
TRINITY_DN14685_c3_g4::TRINITY_DN14685_c3_g4_i1::g.59875::m.59875	TaPBC_Unk_90	TaPBC_Unk_90	5	2	8.06E+06	7.91E+06	7.86E+06	5.53E+06	5.03E+06
TRINITY_DN14452_c1_g5::TRINITY_DN14452_c1_g5_i1::g.15266::m.15266	Tja_CIIMET_03	Tja_CIIMET_03	5	4	6.69E+06	7.36E+06	7.13E+06	4.60E+06	3.89E+06
TRINITY_DN14553_c3_g1::TRINITY_DN14553_c3_g1_i2::g.47431::m.47431	Tja_CIIMET_12	Tja_CIIMET_12	5	1	2.58E+07	2.64E+07	2.48E+07	3.09E+07	3.00E+07
TRINITY_DN14469_c7_g6::TRINITY_DN14469_c7_g6_i3::g.15898::m.15898; TRINITY_DN14469_c7_g6::TRINITY_DN14469_c7_g6_i7::g.15878::m.15878	Tja_NaTx01	Tja_NaTx01	5	5	3.43E+07	3.44E+07	3.97E+07	3.05E+08	2.24E+08
TRINITY_DN14174_c0_g1::TRINITY_DN14174_c0_g1_i4::g.72819::m.72819; TRINITY_DN14174_c0_g1::TRINITY_DN14174_c0_g1_i7::g.72816::m.72816	Tja_NaTx31;sp|C9X4K6|SCNA8_TITDI	5	0		5.64E+08	5.78E+08	5.40E+08	9.83E+07	9.31E+07
TRINITY_DN14055_c0_g1::TRINITY_DN14055_c0_g1_i2::g.15332::m.15332; TRINITY_DN14055_c0_g1::TRINITY_DN14055_c0_g1_i3::g.15337::m.15337; TRINITY_DN14527_c6_g2::TRINITY_DN14527_c6_g2_i2::g.46713::m.46713	Tja_SP16	Tja_SP16	5	4	882851	856471	849895		
TRINITY_DN14478_c5_g1::TRINITY_DN14478_c5_g1_i11::g.16749::m.16749; TRINITY_DN14478_c5_g1::TRINITY_DN14478_c5_g1_i27::g.16761::m.16761	Tja_Unk_07	Tja_Unk_07	5	2	2.10E+08	2.01E+08	2.06E+08	1.14E+08	1.18E+08
TRINITY_DN14090_c1_g1::TRINITY_DN14090_c1_g1_i4::g.14718::m.14718; TRINITY_DN14553_c2_g1::TRINITY_DN14553_c2_g1_i7::g.47427::m.47427	Tja_Unk_57	Tja_Unk_57	5	1	4.97E+07	6.00E+07	5.59E+07	1.28E+07	1.24E+07
TRINITY_DN14395_c2_g1::TRINITY_DN14395_c2_g1_i3::g.4837::m.4837	XP_023217176.1	XP_023217176.1	5	5	1.26E+07	1.35E+07	1.31E+07		
TRINITY_DN1609_c0_g1::TRINITY_DN1609_c0_g1_i1::g.77623::m.77623	XP_023218885.1	XP_023218885.1	5	5	941709	979448	890634		
TRINITY_DN51526_c0_g1::TRINITY_DN51526_c0_g1_i1::g.6492::m.6492	XP_023219527.1	XP_023219527.1	5	5	1.69E+06	1.73E+06	1.76E+06		
TRINITY_DN12417_c0_g1::TRINITY_DN12417_c0_g1_i1::g.57486::m.57486	XP_023227775.1	XP_023227775.1	5	4	245906	245300	254437		
TRINITY_DN8767_c0_g1::TRINITY_DN8767_c0_g1_i2::g.30479::m.30479	XP_023234878.1	XP_023234878.1	5	5	1.23E+06	1.29E+06	1.36E+06		
TRINITY_DN13550_c0_g2::TRINITY_DN13550_c0_g2_i1::g.57956::m.57956	XP_023244022.1	XP_023244022.1	5	5	9.70E+06	9.19E+06	9.06E+06	4.67E+06	4.26E+06
TRINITY_DN13274_c0_g1::TRINITY_DN13274_c0_g1_i2::g.18792::m.18792	XP_067125559.1	XP_067125559.1	5	4	676723	731134	665511		

Protein Group	Protein Names	Genes	N. Sequences	N. Proteotypic sequences	G:\Compartida\championi\GC_4_2_Tityus_championi_tryp_1.raw	G:\Compartida\championi\GC_5_2_Tityus_championi_tryp_2.raw	G:\Compartida\championi\GC_6_2_Tityus_championi_tryp_3.raw	G:\Compartida\championi\GC_16_2_Tityus_championi_cht_1.raw	G:\Compartida\championi\GC_17_2_Tityus_championi_cht_2.raw
TRINITY_DN13752_c4_g1::TRINITY_DN13752_c4_g1_i2::g.70978::m.70978	XP_067127455.1	XP_067127455.1	5	1	458552	518392	480419		
TRINITY_DN14545_c3_g1::TRINITY_DN14545_c3_g1_i9::g.44680::m.44680	XP_067129367.1	XP_067129367.1	5	5	4.38E+06	4.74E+06	4.69E+06		
TRINITY_DN11610_c0_g1::TRINITY_DN11610_c0_g1_i1::g.3122::m.3122	Cbi_TRINITY_DN329_c0_g1_i1.p1	4	4		250050	301443	281680		
TRINITY_DN14430_c4_g1::TRINITY_DN14430_c4_g1_i2::g.17145::m.17145	metalloasthenase101	No	4	0	890789	873439	868256	980388	919760
TRINITY_DN14639_c1_g2::TRINITY_DN14639_c1_g2_i10::g.59348::m.59348	metalloasthenase116	metalloasthenase116	4	0	6.79E+06	6.80E+06	6.80E+06		
TRINITY_DN14355_c0_g1::TRINITY_DN14355_c0_g1_i12::g.4706::m.4706	TaPBC_Lectin_31	TaPBC_Lectin_31	4	0	8.60E+06	1.05E+07	8.76E+06	1.79E+06	2.02E+06
TRINITY_DN14215_c4_g4::TRINITY_DN14215_c4_g4_i3::g.66249::m.66249	TaPBC_Unk_131	TaPBC_Unk_131	4	4	2.07E+07	2.16E+07	2.06E+07	2.13E+07	2.06E+07
TRINITY_DN14128_c0_g1::TRINITY_DN14128_c0_g1_i4::g.75891::m.75891; TRINITY_DN13468_c0_g2::TRINITY_DN13468_c0_g2_i2::g.24479::m.24479	Tja_CIIMET_08	Tja_CIIMET_08	4	4	2.10E+07	2.20E+07	2.37E+07		
TRINITY_DN14293_c0_g2::TRINITY_DN14293_c0_g2_i1::g.64694::m.64694	Tja_CRISP18	Tja_CRISP18	4	0	1.27E+08	1.30E+08	1.26E+08	5.48E+07	4.71E+07
TRINITY_DN14345_c5_g2::TRINITY_DN14345_c5_g2_i1::g.4012::m.4012	Tja_KTx34	Tja_KTx34	4	3	3.28E+08	3.29E+08	3.33E+08	3.12E+08	2.85E+08
TRINITY_DN14293_c0_g2::TRINITY_DN14293_c0_g2_i2::g.59839::m.59839	Tja_NaTx18	Tja_NaTx18	4	3	1.59E+08	1.67E+08	1.64E+08	6.28E+08	6.00E+08
TRINITY_DN14306_c2_g1::TRINITY_DN14306_c2_g1_i4::g.5771::m.5771; TRINITY_DN14537_c1_g1::TRINITY_DN14537_c1_g1_i3::g.48584::m.48584	Tja_NaTx43	Tja_NaTx43	4	4	7.74E+07	8.55E+07	8.26E+07	1.15E+07	1.14E+07
TRINITY_DN14653_c5_g2::TRINITY_DN14653_c5_g2_i5::g.62160::m.62160; TRINITY_DN14653_c5_g2::TRINITY_DN14653_c5_g2_i8::g.62162::m.62162	Tja_Unk_51	Tja_Unk_51	4	1	1.50E+08	1.83E+08	1.73E+08	6.07E+07	5.61E+07
TRINITY_DN14090_c1_g1::TRINITY_DN14090_c1_g1_i8::g.14715::m.14715; TRINITY_DN14553_c2_g1::TRINITY_DN14553_c2_g1_i5::g.47420::m.47420	Tja_Unk_57	Tja_Unk_57	4	3	1.53E+08	1.57E+08	1.46E+08	9.32E+07	9.36E+07
TRINITY_DN14128_c0_g3::TRINITY_DN14128_c0_g3_i1::g.75894::m.75894	Tja_Unk_63	Tja_Unk_63	4	4	4.65E+07	5.68E+07	4.97E+07	1.71E+07	1.89E+07
TRINITY_DN13803_c0_g2::TRINITY_DN13803_c0_g2_i1::g.9928::m.9928	XP_023212112.1	XP_023212112.1	4	4	2.01E+06	2.23E+06	2.47E+06		
TRINITY_DN14556_c3_g1::TRINITY_DN14556_c3_g1_i1::g.47472::m.47472	XP_023220762.1	XP_023220762.1	4	3	5.46E+06	5.27E+06	5.14E+06		
TRINITY_DN14121_c1_g2::TRINITY_DN14121_c1_g2_i6::g.73483::m.73483	XP_023225614.1	XP_023225614.1	4	4	5.97E+06	5.63E+06	5.70E+06	3.46E+06	3.78E+06
TRINITY_DN14051_c0_g1::TRINITY_DN14051_c0_g1_i1::g.13016::m.13016	XP_023228896.1	XP_023228896.1	4	2	162605	195513	210382		

**Protein Group**	**Protein Names**	**Genes**	**N. Sequences**	**N. Proteotypic sequences**	**G:\Compartida\championi\GC_4_2_Tityus_championi_tryp_1.raw**	**G:\Compartida\championi\GC_5_2_Tityus_championi_tryp_2.raw**	**G:\Compartida\championi\GC_6_2_Tityus_championi_tryp_3.raw**	**G:\Compartida\championi\GC_16_2_Tityus_championi_cht_1.raw**	**G:\Compartida\championi\GC_17_2_Tityus_championi_cht_2.raw**
TRINITY_DN12890_c2_g1::TRINITY_DN12890_c2_g1_i3::g.71554::m.71554	XP_023236636.1	XP_023236636.1	4	1	2.81E+06	2.66E+06	2.95E+06		
TRINITY_DN11296_c0_g1::TRINITY_DN11296_c0_g1_i2::g.38490::m.38490; TRINITY_DN11898_c0_g1::TRINITY_DN11898_c0_g1_i1::g.25234::m.25234	XP_023244316.1;XP_067128565.1	4	0		1.05E+06	1.09E+06	1.05E+06		
TRINITY_DN13929_c0_g1::TRINITY_DN13929_c0_g1_i2::g.70098::m.70098	XP_023244342.1	XP_023244342.1	4	4	620316	543142	572505		
TRINITY_DN12308_c0_g1::TRINITY_DN12308_c0_g1_i2::g.43720::m.43720	XP_067120019.1	XP_067120019.1	4	4	3.34E+06	3.22E+06	3.20E+06	2.84E+06	2.47E+06
TRINITY_DN7146_c0_g1::TRINITY_DN7146_c0_g1_i1::g.52109::m.52109	XP_067132834.1	XP_067132834.1	4	4	301468	337910	341932		
TRINITY_DN9629_c0_g1::TRINITY_DN9629_c0_g1_i1::g.2710::m.2710	XP_067134649.1	XP_067134649.1	4	4	821589	902815	855445	878816	854665
TRINITY_DN14510_c0_g2::TRINITY_DN14510_c0_g2_i1::g.47682::m.47682	XP_067138268.1	XP_067138268.1	4	4	3.03E+06	3.16E+06	2.93E+06		
TRINITY_DN13680_c0_g1::TRINITY_DN13680_c0_g1_i2::g.28198::m.28198	XP_067139630.1	XP_067139630.1	4	4	378610	358248	398621		
TRINITY_DN13938_c0_g2::TRINITY_DN13938_c0_g2_i1::g.69473::m.69473; TRINITY_DN13938_c0_g2::TRINITY_DN13938_c0_g2_i2::g.69467::m.69467	XP_067141565.1;XP_067141566.1	4	0		249546	231325	223495		
TRINITY_DN10723_c0_g1::TRINITY_DN10723_c0_g1_i1::g.39196::m.39196; TRINITY_DN10723_c0_g1::TRINITY_DN10723_c0_g1_i2::g.39193::m.39193	XP_067141683.1;XP_067141684.1	4	0		611241	596173	525205		
TRINITY_DN12390_c0_g1::TRINITY_DN12390_c0_g1_i1::g.43909::m.43909	XP_067142637.1	XP_067142637.1	4	4	2.43E+06	2.51E+06	2.27E+06	2.02E+06	1.67E+06
TRINITY_DN11197_c0_g1::TRINITY_DN11197_c0_g1_i2::g.28901::m.28901	XP_067144915.1	XP_067144915.1	4	3	235168	180534	206366		
TRINITY_DN11865_c0_g1::TRINITY_DN11865_c0_g1_i1::g.25384::m.25384	TRINITY_DN11865_c0_g1::TRINITY_DN11865_c0_g1_i1::g.25384::m.25384	No	4	4	7.40E+06	7.53E+06	8.50E+06	6.93E+06	6.89E+06
TRINITY_DN14235_c0_g1::TRINITY_DN14235_c0_g1_i1::g.62421::m.62421	TRINITY_DN14235_c0_g1::TRINITY_DN14235_c0_g1_i1::g.62421::m.62421	No	4	2	2.54E+07	2.63E+07	2.69E+07	4.08E+07	3.91E+07
TRINITY_DN14567_c2_g1::TRINITY_DN14567_c2_g1_i15::g.48261::m.48261	metalloasthenase09	metalloasthenase09	3	3	4.63E+06	4.57E+06	4.75E+06	4.84E+06	4.72E+06
TRINITY_DN14679_c0_g1::TRINITY_DN14679_c0_g1_i21::g.59128::m.59128	metalloasthenase101	metalloasthenase101	3	0	1.12E+06	1.21E+06	1.29E+06		
TRINITY_DN14406_c1_g2::TRINITY_DN14406_c1_g2_i5::g.18707::m.18707	metalloasthenase109	metalloasthenase109	3	0	3.13E+06	2.94E+06	2.96E+06		

Protein Group	Protein Names	Genes	N. Sequences	N. Proteotypic sequences	G:\Compartida\championi\GC_4_2_Tityus_championi_tryp_1.raw	G:\Compartida\championi\GC_5_2_Tityus_championi_tryp_2.raw	G:\Compartida\championi\GC_6_2_Tityus_championi_tryp_3.raw	G:\Compartida\championi\GC_16_2_Tityus_championi_cht_1.raw	G:\Compartida\championi\GC_17_2_Tityus_championi_cht_2.raw
TRINITY_DN14586_c1_g1::TRINITY_DN14586_c1_g1_i6::g.47222::m.47222	metalloasthenase114	metalloasthenase114	3	1	1.06E+08	1.01E+08	9.34E+07	1.30E+07	1.13E+07
TRINITY_DN14567_c3_g1::TRINITY_DN14567_c3_g1_i1::g.48283::m.48283	metalloasthenase54	metalloasthenase54	3	1	8.87E+07	8.85E+07	8.78E+07		
TRINITY_DN14586_c1_g1::TRINITY_DN14586_c1_g1_i8::g.47228::m.47228	Metallojaimease109	Metallojaimease109	3	0	2.70E+07	2.56E+07	2.74E+07	2.76E+07	2.68E+07
TRINITY_DN14068_c0_g3::TRINITY_DN14068_c0_g3_i4::g.14182::m.14182	Metallojaimease129	Metallojaimease129	3	1	1.31E+06	1.30E+06	1.35E+06		
TRINITY_DN14679_c0_g3::TRINITY_DN14679_c0_g3_i9::g.59163::m.59163	Metallojaimease90	Metallojaimease90	3	0	3.73E+06	4.23E+06	4.02E+06		
TRINITY_DN14589_c0_g1::TRINITY_DN14589_c0_g1_i1::g.48623::m.48623	TaPBC_Lectin_16	TaPBC_Lectin_16	3	0	714511		589886	908787	806233
TRINITY_DN14508_c0_g2::TRINITY_DN14508_c0_g2_i18::g.45828::m.45828	TaPBP-KTx_06	TaPBP-KTx_06	3	2	4.09E+06	3.77E+06	3.52E+06	5.80E+06	5.65E+06
TRINITY_DN37510_c0_g1::TRINITY_DN37510_c0_g1_i1::g.32376::m.32376	TaPBP-LVP02	TaPBP-LVP02	3	3	526944	535482	562609	3.21E+06	2.33E+06
TRINITY_DN14573_c2_g4::TRINITY_DN14573_c2_g4_i4::g.48553::m.48553	TaPBP-NaTx29	TaPBP-NaTx29	3	3	1.19E+08	1.29E+08	1.24E+08	1.29E+08	1.25E+08
TRINITY_DN14306_c1_g1::TRINITY_DN14306_c1_g1_i3::g.5758::m.5758;<br>TRINITY_DN14306_c1_g1::TRINITY_DN14306_c1_g1_i4::g.5753::m.5753;<br>TRINITY_DN14400_c1_g1::TRINITY_DN14400_c1_g1_i1::g.17495::m.17495	TaPBP-NaTx30	TaPBP-NaTx30	3	3	1.99E+06	2.07E+06	1.99E+06	1.28E+06	1.02E+06
TRINITY_DN14450_c2_g7::TRINITY_DN14450_c2_g7_i1::g.16139::m.16139	Tja_CIIMET_03	Tja_CIIMET_03	3	3	1.62E+07	1.83E+07	1.76E+07	822112	680599
TRINITY_DN14553_c2_g2::TRINITY_DN14553_c2_g2_i1::g.47430::m.47430	Tja_CIIMET_03	Tja_CIIMET_03	3	3	4.31E+06	5.28E+06	4.74E+06	4.62E+06	4.48E+06
TRINITY_DN12896_c0_g1::TRINITY_DN12896_c0_g1_i1::g.71911::m.71911	Tja_KTx09	Tja_KTx09	3	3	6.06E+08	8.20E+08	7.80E+08	2.31E+08	1.95E+08
TRINITY_DN14508_c0_g2::TRINITY_DN14508_c0_g2_i5::g.45833::m.45833	Tja_KTx45	Tja_KTx45	3	3	8.04E+06	8.19E+06	8.15E+06		7.96E+06
TRINITY_DN12113_c1_g1::TRINITY_DN12113_c1_g1_i1::g.34120::m.34120	Tja_LVP01	Tja_LVP01	3	3	4.43E+06	4.64E+06	4.66E+06		
TRINITY_DN13192_c0_g1::TRINITY_DN13192_c0_g1_i1::g.49048::m.49048	Tja_NaTx09	Tja_NaTx09	3	3	1.73E+06	2.03E+06	2.94E+06		2.20E+06
TRINITY_DN14432_c1_g1::TRINITY_DN14432_c1_g1_i2::g.17392::m.17392	Tja_NaTx51	Tja_NaTx51	3	3	4.15E+07	3.89E+07	3.68E+07		
TRINITY_DN13397_c1_g1::TRINITY_DN13397_c1_g1_i1::g.50918::m.50918	Tja_SVWC_01	Tja_SVWC_01	3	3	3.05E+07	3.21E+07	3.23E+07		
TRINITY_DN14543_c1_g3::TRINITY_DN14543_c1_g3_i1::g.48036::m.48036	Tja_Unk_01	Tja_Unk_01	3	3	9.15E+07	8.70E+07	8.10E+07		

Protein Group	Protein Names	Genes	N. Sequences	N. Proteotypic sequences	G:\Compartida\championi\GC_4_2_Tityus_championi_tryp_1.raw	G:\Compartida\championi\GC_5_2_Tityus_championi_tryp_2.raw	G:\Compartida\championi\GC_6_2_Tityus_championi_tryp_3.raw	G:\Compartida\championi\GC_16_2_Tityus_championi_cht_1.raw	G:\Compartida\championi\GC_17_2_Tityus_championi_cht_2.raw
TRINITY_DN14304_c6_g7::TRINITY_DN14304_c6_g7_i1::g.4915::m.4915	Tja_Unk_04	Tja_Unk_04	3	3	1.92E+06	1.95E+06	1.68E+06	4.19E+06	3.98E+06
TRINITY_DN9159_c0_g1::TRINITY_DN9159_c0_g1_i2::g.22407::m.22407	XP_023214029.1	XP_023214029.1	3	3	639175	599348	589908		
TRINITY_DN14116_c0_g3::TRINITY_DN14116_c0_g3_i1::g.73259::m.73259; TRINITY_DN14206_c3_g2::TRINITY_DN14206_c3_g2_i3::g.60784::m.60784; TRINITY_DN14206_c3_g2::TRINITY_DN14206_c3_g2_i4::g.60787::m.60787; TRINITY_DN14378_c6_g1::TRINITY_DN14378_c6_g1_i1::g.5090::m.5090; TRINITY_DN14378_c6_g2::TRINITY_DN14378_c6_g2_i1::g.5092::m.5092; TRINITY_DN14378_c6_g3::TRINITY_DN14378_c6_g3_i2::g.5094::m.5094; TRINITY_DN14378_c6_g4::TRINITY_DN14378_c6_g4_i1::g.5098::m.5098	XP_023214856.1;XP_023237525.1;XP_067119932.1;XP_067132182.1	3	0	No	5.54E+06	5.49E+06	5.83E+06	6.80E+06	
TRINITY_DN12484_c0_g1::TRINITY_DN12484_c0_g1_i1::g.57435::m.57435	XP_023217415.1	XP_023217415.1	3	3	1.28E+07	1.31E+07	1.34E+07		
TRINITY_DN7218_c0_g1::TRINITY_DN7218_c0_g1_i1::g.48712::m.48712	XP_023217657.1	XP_023217657.1	3	3	2.68E+06	2.80E+06	2.61E+06		
TRINITY_DN12663_c0_g1::TRINITY_DN12663_c0_g1_i4::g.43336::m.43336; TRINITY_DN12930_c0_g1::TRINITY_DN12930_c0_g1_i1::g.30110::m.30110; TRINITY_DN12930_c0_g1::TRINITY_DN12930_c0_g1_i2::g.30109::m.30109; TRINITY_DN12930_c0_g1::TRINITY_DN12930_c0_g1_i3::g.30108::m.30108	XP_023219848.1;XP_023219850.1	3	0		307374	322326	312945		
TRINITY_DN14094_c0_g1::TRINITY_DN14094_c0_g1_i1::g.16916::m.16916; TRINITY_DN14094_c0_g1::TRINITY_DN14094_c0_g1_i4::g.16919::m.16919; TRINITY_DN14315_c0_g4::TRINITY_DN14315_c0_g4_i1::g.5821::m.5821	XP_023224333.1;XP_067143623.1;XP_067144536.1	3	0		423898	305135	381872		
TRINITY_DN14151_c0_g1::TRINITY_DN14151_c0_g1_i1::g.76216::m.76216	XP_023224525.1	XP_023224525.1	3	3	367352	326573	349459		
TRINITY_DN7296_c0_g1::TRINITY_DN7296_c0_g1_i1::g.48731::m.48731	XP_023224526.1	XP_023224526.1	3	3	436466	424328	410791		
TRINITY_DN13910_c0_g1::TRINITY_DN13910_c0_g1_i1::g.65500::m.65500; TRINITY_DN14427_c1_g1::TRINITY_DN14427_c1_g1_i1::g.14927::m.14927	XP_023231741.1	XP_023231741.1	3	1	1.11E+06	1.09E+06	1.24E+06	308590	337995
TRINITY_DN13246_c0_g1::TRINITY_DN13246_c0_g1_i3::g.18224::m.18224; TRINITY_DN13246_c0_g1::TRINITY_DN13246_c0_g1_i1::g.18218::m.18218; TRINITY_DN13246_c0_g1::TRINITY_DN13246_c0_g1_i8::g.18200::m.18200	XP_023236966.1;XP_067145227.1	3	0		928714	685895	783740	567688	
TRINITY_DN14027_c1_g2::TRINITY_DN14027_c1_g2_i1::g.13919::m.13919	XP_023244829.1	XP_023244829.1	3	2	1.71E+06	1.75E+06	1.73E+06		

Protein Group	Protein Names	Genes	N. Sequences	N. Proteotypic sequences	G:\Compartida\championi\GC_4_2_Tityus_championi_tryp_1.raw	G:\Compartida\championi\GC_5_2_Tityus_championi_tryp_2.raw	G:\Compartida\championi\GC_6_2_Tityus_championi_tryp_3.raw	G:\Compartida\championi\GC_16_2_Tityus_championi_cht_1.raw	G:\Compartida\championi\GC_17_2_Tityus_championi_cht_2.raw
TRINITY_DN14145_c0_g1::TRINITY_DN14145_c0_g1_i1::g.76082::m.76082	XP_023244832.1	XP_023244832.1	3	3	4.01E+06	4.10E+06	3.71E+06		
TRINITY_DN14063_c0_g1::TRINITY_DN14063_c0_g1_i2::g.13748::m.13748; TRINITY_DN14063_c0_g1::TRINITY_DN14063_c0_g1_i3::g.13773::m.13773; TRINITY_DN14063_c0_g1::TRINITY_DN14063_c0_g1_i4::g.13761::m.13761; TRINITY_DN14063_c0_g1::TRINITY_DN14063_c0_g1_i5::g.13738::m.13738	XP_067123419.1	XP_067123419.1	3	3	581683	508923	491110		
TRINITY_DN6070_c0_g1::TRINITY_DN6070_c0_g1_i1::g.77226::m.77226	XP_067124239.1	XP_067124239.1	3	3	104668	124158	109209		
TRINITY_DN13402_c0_g1::TRINITY_DN13402_c0_g1_i2::g.22952::m.22952	XP_067124462.1	XP_067124462.1	3	3	604800	692690	618105		
TRINITY_DN2060_c0_g1::TRINITY_DN2060_c0_g1_i1::g.35180::m.35180	XP_067126432.1	XP_067126432.1	3	3	456271	343860	476922		
TRINITY_DN13120_c0_g1::TRINITY_DN13120_c0_g1_i1::g.49446::m.49446; TRINITY_DN14345_c0_g1::TRINITY_DN14345_c0_g1_i1::g.3946::m.3946	XP_067126872.1;XP_067144333.1	3	0		679223	678344	672651		
TRINITY_DN12417_c0_g1::TRINITY_DN12417_c0_g1_i2::g.57479::m.57479	XP_067128167.1	XP_067128167.1	3	1	834442	812695	689669		
TRINITY_DN13840_c7_g6::TRINITY_DN13840_c7_g6_i1::g.11359::m.11359	XP_067131452.1	XP_067131452.1	3	3	479971	490278	423164		
TRINITY_DN14436_c3_g3::TRINITY_DN14436_c3_g3_i1::g.15553::m.15553	XP_067135662.1	XP_067135662.1	3	3	24170.5	23335.9	27788.2		
TRINITY_DN11910_c0_g2::TRINITY_DN11910_c0_g2_i1::g.54326::m.54326	XP_067135991.1	XP_067135991.1	3	3	4.15E+06	3.93E+06	4.10E+06		
TRINITY_DN12127_c0_g1::TRINITY_DN12127_c0_g1_i2::g.34125::m.34125	XP_067137896.1	XP_067137896.1	3	3	70364.1	87528.8	76284.7		
TRINITY_DN11678_c0_g1::TRINITY_DN11678_c0_g1_i1::g.3499::m.3499	XP_067142681.1	XP_067142681.1	3	3	131922	174420	165637		
TRINITY_DN13901_c0_g1::TRINITY_DN13901_c0_g1_i1::g.69719::m.69719	Cbi_TRINITY_DN1030_c3_g1_i1.p1	2	2		8.53E+06	8.15E+06	8.14E+06		
TRINITY_DN5402_c0_g1::TRINITY_DN5402_c0_g1_i1::g.63866::m.63866	Cbi_TRINITY_DN166_c0_g1_i1.p1	2	2		132023	102198	114127		
TRINITY_DN14659_c3_g1::TRINITY_DN14659_c3_g1_i14::g.61333::m.61333	metalloasthenase01	metalloasthenase01	2	2	4.49E+08	4.00E+08	4.07E+08		
TRINITY_DN14659_c3_g1::TRINITY_DN14659_c3_g1_i8::g.61322::m.61322	metalloasthenase01	metalloasthenase01	2	0	2.30E+08	2.41E+08	2.35E+08		
TRINITY_DN14567_c2_g1::TRINITY_DN14567_c2_g1_i17::g.48250::m.48250	metalloasthenase120	metalloasthenase120	2	2	71078.5	83484.9	77819.7		
TRINITY_DN14679_c0_g3::TRINITY_DN14679_c0_g3_i7::g.59166::m.59166	metalloasthenase65	metalloasthenase65	2	0	510363	566811	615972		

Protein Group	Protein Names	Genes	N. Sequences	N. Proteotypic sequences	G:\Compartida\championi\GC_4_2_Tityus_championi_tryp_1.raw	G:\Compartida\championi\GC_5_2_Tityus_championi_tryp_2.raw	G:\Compartida\championi\GC_6_2_Tityus_championi_tryp_3.raw	G:\Compartida\championi\GC_16_2_Tityus_championi_cht_1.raw	G:\Compartida\championi\GC_17_2_Tityus_championi_cht_2.raw
TRINITY_DN14523_c1_g1::TRINITY_DN14523_c1_g1_i3::g.47355::m.47355	Metallojaimease66	Metallojaimease66	2	1	892289	802018	802324		
TRINITY_DN14375_c2_g1::TRINITY_DN14375_c2_g1_i1::g.5594::m.5594;<br>TRINITY_DN14375_c2_g1::TRINITY_DN14375_c2_g1_i2::g.5593::m.5593;<br>TRINITY_DN14375_c2_g1::TRINITY_DN14375_c2_g1_i6::g.5592::m.5592;<br>TRINITY_DN14375_c2_g1::TRINITY_DN14375_c2_g1_i7::g.5587::m.5587;<br>TRINITY_DN14571_c0_g1::TRINITY_DN14571_c0_g1_i7::g.48228::m.48228	No	No	2	2	3.90E+06	3.38E+06	3.60E+06		
TRINITY_DN14508_c0_g2::TRINITY_DN14508_c0_g2_i3::g.45825::m.45825	sp|P84777|KA156_TITDI	sp|P84777|KA156_TITDI	2	2	5.87E+06	5.90E+06	6.99E+06	3.19E+06	3.03E+06
TRINITY_DN13370_c0_g1::TRINITY_DN13370_c0_g1_i1::g.50777::m.50777	TaPBC_Chitinase_01	TaPBC_Chitinase_01	2	2	9.79E+07	1.03E+08	9.82E+07	3.09E+07	3.27E+07
TRINITY_DN14338_c0_g1::TRINITY_DN14338_c0_g1_i4::g.3426::m.3426;<br>TRINITY_DN14553_c3_g1::TRINITY_DN14553_c3_g1_i4::g.47433::m.47433	TaPBC_CIIMET_10;Tja_CIIMET_12	2	0		4.19E+08	4.94E+08	4.39E+08	4.18E+07	2.88E+07
TRINITY_DN14338_c0_g1::TRINITY_DN14338_c0_g1_i5::g.3423::m.3423	TaPBC_CIIMET_11	TaPBC_CIIMET_11	2	1	3.77E+06	3.69E+06	3.77E+06	5.92E+06	6.56E+06
TRINITY_DN14589_c0_g1::TRINITY_DN14589_c0_g1_i17::g.48606::m.48606	TaPBC_Lectin_16	TaPBC_Lectin_16	2	0	2.83E+07	2.84E+07	2.40E+07	4.00E+07	3.84E+07
TRINITY_DN14589_c0_g1::TRINITY_DN14589_c0_g1_i14::g.48597::m.48597	TaPBC_Lectin_17	TaPBC_Lectin_17	2	2				3.54E+07	3.62E+07
TRINITY_DN11402_c0_g1::TRINITY_DN11402_c0_g1_i1::g.24934::m.24934	TaPBC_Unk_152	TaPBC_Unk_152	2	2	4.02E+06	4.16E+06	4.11E+06		
TRINITY_DN14051_c2_g2::TRINITY_DN14051_c2_g2_i4::g.14810::m.14810;<br>TRINITY_DN14463_c1_g1::TRINITY_DN14463_c1_g1_i2::g.16865::m.16865	TaPBC_Unk_86	TaPBC_Unk_86	2	0	1.64E+06	1.75E+06	1.75E+06		
TRINITY_DN14218_c0_g5::TRINITY_DN14218_c0_g5_i1::g.59104::m.59104	TaPBP-KTx_06	TaPBP-KTx_06	2	2	2.59E+06	2.58E+06	2.55E+06	3.10E+06	2.46E+06
TRINITY_DN14508_c0_g2::TRINITY_DN14508_c0_g2_i15::g.45843::m.45843	TaPBP-KTx_53	TaPBP-KTx_53	2	2	799962	833046	857237	857320	830646
TRINITY_DN14573_c2_g7::TRINITY_DN14573_c2_g7_i1::g.48561::m.48561	TaPBP-NaTx39	TaPBP-NaTx39	2	1	1.70E+08	1.61E+08	1.53E+08		
TRINITY_DN14615_c1_g1::TRINITY_DN14615_c1_g1_i5::g.60932::m.60932	Tja_CIIMET_03	Tja_CIIMET_03	2	2	2.21E+06	2.44E+06	2.19E+06		
TRINITY_DN14450_c2_g2::TRINITY_DN14450_c2_g2_i13::g.16112::m.16112	Tja_CIIMET_04	Tja_CIIMET_04	2	2	25839.6	23765.9	24813.5	25949.6	25177.4
TRINITY_DN14442_c2_g5::TRINITY_DN14442_c2_g5_i5::g.17242::m.17242	Tja_KTx05	Tja_KTx05	2	2	4.54E+07	3.97E+07	4.25E+07		
TRINITY_DN13470_c0_g1::TRINITY_DN13470_c0_g1_i1::g.23065::m.23065	Tja_KTx40	Tja_KTx40	2	2	2.07E+07	2.57E+07	5.73E+07		

Protein Group	Protein Names	Genes	N. Sequences	N. Proteotypic sequences	G:\Compartida\championi\GC_4_2_Tityus_championi_tryp_1.raw	G:\Compartida\championi\GC_5_2_Tityus_championi_tryp_2.raw	G:\Compartida\championi\GC_6_2_Tityus_championi_tryp_3.raw	G:\Compartida\championi\GC_16_2_Tityus_championi_cht_1.raw	G:\Compartida\championi\GC_17_2_Tityus_championi_cht_2.raw
TRINITY_DN14508_c0_g2::TRINITY_DN14508_c0_g2_i7::g.45821::m.45821	Tja_KTx45	Tja_KTx45	2	2	4.80E+07	4.98E+07	4.97E+07		
TRINITY_DN14677_c0_g4::TRINITY_DN14677_c0_g4_i11::g.61083::m.61083	Tja_NaTx04	Tja_NaTx04	2	0	1.79E+08	2.05E+08	2.05E+08		
TRINITY_DN14256_c3_g3::TRINITY_DN14256_c3_g3_i1::g.66510::m.66510	Tja_NaTx18	Tja_NaTx18	2	2	2.01E+06	1.84E+06	1.64E+06	1.89E+06	1.72E+06
TRINITY_DN14379_c2_g2::TRINITY_DN14379_c2_g2_i11::g.5476::m.5476	Tja_NaTx48	Tja_NaTx48	2	2	1.18E+07	1.15E+07	1.15E+07	1.58E+07	1.57E+07
TRINITY_DN13881_c0_g1::TRINITY_DN13881_c0_g1_i1::g.10602::m.10602; TRINITY_DN14114_c2_g2::TRINITY_DN14114_c2_g2_i1::g.73106::m.73106; TRINITY_DN14114_c2_g2::TRINITY_DN14114_c2_g2_i3::g.73107::m.73107; TRINITY_DN14114_c2_g2::TRINITY_DN14114_c2_g2_i4::g.73103::m.73103; TRINITY_DN14114_c2_g2::TRINITY_DN14114_c2_g2_i5::g.73104::m.73104; TRINITY_DN14114_c2_g2::TRINITY_DN14114_c2_g2_i6::g.73108::m.73108; TRINITY_DN14114_c2_g2::TRINITY_DN14114_c2_g2_i7::g.73105::m.73105; TRINITY_DN14441_c3_g2::TRINITY_DN14441_c3_g2_i2::g.17616::m.17616	Tja_NBDP_18;sp|A0A1D3IXJ5|NDB32_TITOB	2	0		6.81E+06	7.47E+06	6.94E+06	7.88E+06	6.38E+06
TRINITY_DN11569_c0_g2::TRINITY_DN11569_c0_g2_i2::g.493::m.493; TRINITY_DN12001_c0_g1::TRINITY_DN12001_c0_g1_i4::g.39724::m.39724	Tja_Unk_05	Tja_Unk_05	2	2	1.81E+06	2.04E+06	2.23E+06		
TRINITY_DN14556_c2_g2::TRINITY_DN14556_c2_g2_i1::g.47470::m.47470	Tja_Unk_148	Tja_Unk_148	2	2	2.64E+06	2.92E+06	3.11E+06		
TRINITY_DN14304_c6_g6::TRINITY_DN14304_c6_g6_i1::g.4913::m.4913	Tja_Unk_43	Tja_Unk_43	2	2	1.11E+07	1.10E+07	1.09E+07	1.18E+07	1.14E+07
TRINITY_DN14418_c1_g3::TRINITY_DN14418_c1_g3_i4::g.18271::m.18271; TRINITY_DN14668_c8_g1::TRINITY_DN14668_c8_g1_i2::g.62238::m.62238	Tja_Unk_59	Tja_Unk_59	2	2	5.83E+06	5.07E+06	5.47E+06		
TRINITY_DN12945_c0_g1::TRINITY_DN12945_c0_g1_i1::g.30257::m.30257	Tja_Unk_66	Tja_Unk_66	2	2	1.69E+07	1.71E+07	1.63E+07	4.35E+06	4.17E+06
TRINITY_DN14599_c0_g1::TRINITY_DN14599_c0_g1_i8::g.46295::m.46295	Tja_Unk_72	Tja_Unk_72	2	1	8.57E+08	5.42E+09	7.98E+08	1.31E+09	1.23E+09
TRINITY_DN14599_c0_g1::TRINITY_DN14599_c0_g1_i3::g.46290::m.46290; TRINITY_DN14599_c0_g1::TRINITY_DN14599_c0_g1_i7::g.46292::m.46292	Tja_Unk_73	Tja_Unk_73	2	2	918691	928242	875868		
TRINITY_DN14466_c0_g1::TRINITY_DN14466_c0_g1_i2::g.17169::m.17169; TRINITY_DN14466_c0_g1::TRINITY_DN14466_c0_g1_i8::g.17177::m.17177;	XP_023210948.1	XP_023210948.1	2	1	116168	135220	117333		

Protein Group	Protein Names	Genes	N. Sequences	N. Proteotypic sequences	G:\Compartida\championi\GC_4_2_Tityus_championi_tryp_1.raw	G:\Compartida\championi\GC_5_2_Tityus_championi_tryp_2.raw	G:\Compartida\championi\GC_6_2_Tityus_championi_tryp_3.raw	G:\Compartida\championi\GC_16_2_Tityus_championi_cht_1.raw	G:\Compartida\championi\GC_17_2_Tityus_championi_cht_2.raw
TRINITY_DN14202_c0_g1::TRINITY_DN14202_c0_g1_i2::g.62519::m.62519; TRINITY_DN14466_c0_g1::TRINITY_DN14466_c0_g1_i3::g.17196::m.17196									
TRINITY_DN13386_c0_g1::TRINITY_DN13386_c0_g1_i1::g.51348::m.51348	XP_023212377.1	XP_023212377.1	2	2	1.08E+06		887968		
TRINITY_DN10545_c0_g1::TRINITY_DN10545_c0_g1_i1::g.53795::m.53795	XP_023212825.1	XP_023212825.1	2	2	349237	417898	321445		
TRINITY_DN37398_c0_g1::TRINITY_DN37398_c0_g1_i1::g.32221::m.32221	XP_023213360.1	XP_023213360.1	2	2	5.45E+06	5.64E+06	5.28E+06		
TRINITY_DN14060_c0_g1::TRINITY_DN14060_c0_g1_i1::g.13071::m.13071	XP_023214020.1	XP_023214020.1	2	2	224662		268426		
TRINITY_DN10978_c0_g3::TRINITY_DN10978_c0_g3_i1::g.35926::m.35926	XP_023215707.1	XP_023215707.1	2	2	191116	232798	286567		
TRINITY_DN14049_c0_g2::TRINITY_DN14049_c0_g2_i1::g.13011::m.13011; TRINITY_DN14049_c0_g2::TRINITY_DN14049_c0_g2_i2::g.13009::m.13009	XP_023220888.1;XP_067138103.1	2	0		274352	311861	263190		
TRINITY_DN11972_c0_g1::TRINITY_DN11972_c0_g1_i1::g.54599::m.54599	XP_023220952.1	XP_023220952.1	2	2	341312	342734	387477		
TRINITY_DN13449_c0_g1::TRINITY_DN13449_c0_g1_i1::g.23149::m.23149	XP_023221466.1	XP_023221466.1	2	2	505500	357245	309259		
TRINITY_DN11889_c0_g1::TRINITY_DN11889_c0_g1_i2::g.25655::m.25655	XP_023223106.1	XP_023223106.1	2	2	376478		426954		
TRINITY_DN14204_c0_g1::TRINITY_DN14204_c0_g1_i1::g.66586::m.66586	XP_023224226.1	XP_023224226.1	2	2	63738	52515.7	59299.1		
TRINITY_DN14458_c1_g2::TRINITY_DN14458_c1_g2_i1::g.17268::m.17268	XP_023230069.1	XP_023230069.1	2	2	396001	482292	410743		
TRINITY_DN14377_c0_g1::TRINITY_DN14377_c0_g1_i1::g.5510::m.5510; TRINITY_DN14377_c0_g1::TRINITY_DN14377_c0_g1_i2::g.5516::m.5516; TRINITY_DN14377_c0_g1::TRINITY_DN14377_c0_g1_i4::g.5509::m.5509	XP_023232019.1	XP_023232019.1	2	2	310056	374246	329700		
TRINITY_DN12949_c0_g1::TRINITY_DN12949_c0_g1_i1::g.29979::m.29979	XP_023232280.1	XP_023232280.1	2	2	18467.3	8771.79	13643.8		
TRINITY_DN14449_c0_g2::TRINITY_DN14449_c0_g2_i1::g.16788::m.16788	XP_023232959.1	XP_023232959.1	2	2	214243	231810	243715		
TRINITY_DN144_c0_g1::TRINITY_DN144_c0_g1_i1::g.23628::m.23628	XP_023233908.1	XP_023233908.1	2	2	18642.4	14636.4	16236.2	261969	202485
TRINITY_DN12330_c0_g1::TRINITY_DN12330_c0_g1_i1::g.44053::m.44053	XP_023234091.1	XP_023234091.1	2	2	1.37E+06	1.17E+06	1.02E+06		
TRINITY_DN11795_c0_g1::TRINITY_DN11795_c0_g1_i2::g.34689::m.34689	XP_023238156.1	XP_023238156.1	2	2	2.49E+06	3.41E+06	2.72E+06		
TRINITY_DN1870_c0_g1::TRINITY_DN1870_c0_g1_i1::g.36444::m.36444	XP_023238156.1	XP_023238156.1	2	2	386627	348663	311436		
TRINITY_DN14006_c0_g1::TRINITY_DN14006_c0_g1_i3::g.15378::m.15378; TRINITY_DN14231_c7_g1::TRINITY_DN14231_c7_g1_i1::g.64912::m.64912;	XP_023238447.1;XP_067126796.1;XP_067126798.1	2	0		474203	473249	476023	372721	323029

Protein Group	Protein Names	Genes	N. Sequences	N. Proteotypic sequences	G:\Compartida\championi\GC_4_2_Tityus_championi_tryp_1.raw	G:\Compartida\championi\GC_5_2_Tityus_championi_tryp_2.raw	G:\Compartida\championi\GC_6_2_Tityus_championi_tryp_3.raw	G:\Compartida\championi\GC_16_2_Tityus_championi_cht_1.raw	G:\Compartida\championi\GC_17_2_Tityus_championi_cht_2.raw
TRINITY_DN14231_c7_g1::TRINITY_DN14231_c7_g1_i2::g.64906::m.64906; TRINITY_DN14231_c7_g1::TRINITY_DN14231_c7_g1_i6::g.64920::m.64920									
TRINITY_DN9643_c0_g1::TRINITY_DN9643_c0_g1_i2::g.2613::m.2613	XP_023239632.1	XP_023239632.1	2	2	6121.27		5429.48		
TRINITY_DN14172_c0_g1::TRINITY_DN14172_c0_g1_i1::g.75383::m.75383	XP_023242593.1	XP_023242593.1	2	2	186248	109140	140628		
TRINITY_DN13513_c0_g1::TRINITY_DN13513_c0_g1_i1::g.57756::m.57756	XP_067121295.1	XP_067121295.1	2	2	585099	679055	545219		
TRINITY_DN13651_c0_g1::TRINITY_DN13651_c0_g1_i2::g.28126::m.28126	XP_067123697.1	XP_067123697.1	2	2	34941.3	35869.9	32686.7		
TRINITY_DN14086_c3_g1::TRINITY_DN14086_c3_g1_i1::g.13507::m.13507	XP_067124801.1	XP_067124801.1	2	2	221995	213107	218843		
TRINITY_DN14423_c5_g1::TRINITY_DN14423_c5_g1_i8::g.18333::m.18333; TRINITY_DN14688_c3_g1::TRINITY_DN14688_c3_g1_i1::g.60453::m.60453	XP_067125135.1	XP_067125135.1	2	1	619626	417102	501450		
TRINITY_DN14275_c2_g1::TRINITY_DN14275_c2_g1_i15::g.62238::m.62238; TRINITY_DN14275_c2_g1::TRINITY_DN14275_c2_g1_i9::g.62241::m.62241;TRINITY_DN14595_c0_g2::TRINITY_DN14595_c0_g2_i10::g.45143::m.45143;TRINITY_DN14595_c0_g2::TRINITY_DN14595_c0_g2_i6::g.45152::m.45152	XP_067127317.1;XP_067138462.1	2	0		582186	485177	444688		
TRINITY_DN11809_c0_g1::TRINITY_DN11809_c0_g1_i1::g.25398::m.25398	XP_067131751.1	XP_067131751.1	2	2	590177	467168	486895		
TRINITY_DN13684_c1_g1::TRINITY_DN13684_c1_g1_i1::g.27626::m.27626;TRINITY_DN13684_c1_g1::TRINITY_DN13684_c1_g1_i2::g.27625::m.27625	XP_067132021.1	XP_067132021.1	2	1		124280	235410		
TRINITY_DN14038_c0_g4::TRINITY_DN14038_c0_g4_i1::g.12987::m.12987	XP_067136737.1	XP_067136737.1	2	2	694939	672277	624485		
TRINITY_DN14637_c1_g1::TRINITY_DN14637_c1_g1_i1::g.59291::m.59291	XP_067139281.1	XP_067139281.1	2	2	19534.6	22946.1	19835		
TRINITY_DN9159_c0_g3::TRINITY_DN9159_c0_g3_i1::g.22413::m.22413	XP_067141033.1	XP_067141033.1	2	2	694458	712315	633197		
TRINITY_DN14429_c1_g1::TRINITY_DN14429_c1_g1_i1::g.17513::m.17513;TRINITY_DN14686_c1_g1::TRINITY_DN14686_c1_g1_i32::g.59393::m.59393;TRINITY_DN14686_c1_g1::TRINITY_DN14686_c1_g1_i8::g.59438::m.59438	XP_067141325.1	XP_067141325.1	2	2	69362.8	77586.6	96218		
TRINITY_DN12103_c0_g1::TRINITY_DN12103_c0_g1_i1::g.34282::m.34282	XP_067142110.1	XP_067142110.1	2	2	2.13E+06	2.11E+06	2.11E+06		
TRINITY_DN12708_c0_g1::TRINITY_DN12708_c0_g1_i1::g.77932::m.77932	XP_067144376.1	XP_067144376.1	2	2	474443	640741	626649		
TRINITY_DN12152_c0_g1::TRINITY_DN12152_c0_g1_i1::g.34292::m.34292	XP_067144456.1	XP_067144456.1	2	2	88591	107645			
