# Supplementary material for: Proteomic characterization of Tityus championi venom and recombinant expression of its major neurotoxin
Source: J Venom Anim Toxins Incl Trop Dis. 2026 Sep 14;32:e20260024. doi: 10.1590/1678-9199-JVATITD-2026-0024 (PMC13574325; doi:10.1590/1678-9199-JVATITD-2026-0024)

J Venom Anim Toxins incl Trop Dis,
https://doi.org/10.1590/1678-9199-JVATITD-2026-0024

Supplementary Material to: Proteomic characterization of *Tityus championi* venom and recombinant expression of its major neurotoxin

Additional file 6. Mass spectra of rTcham27 structural isoforms. Fractions **(A)** rTcham27-4 and **(B)** rTcham27-5 showed molecular masses of 8,953.9 and 8,951.3 Da, respectively.

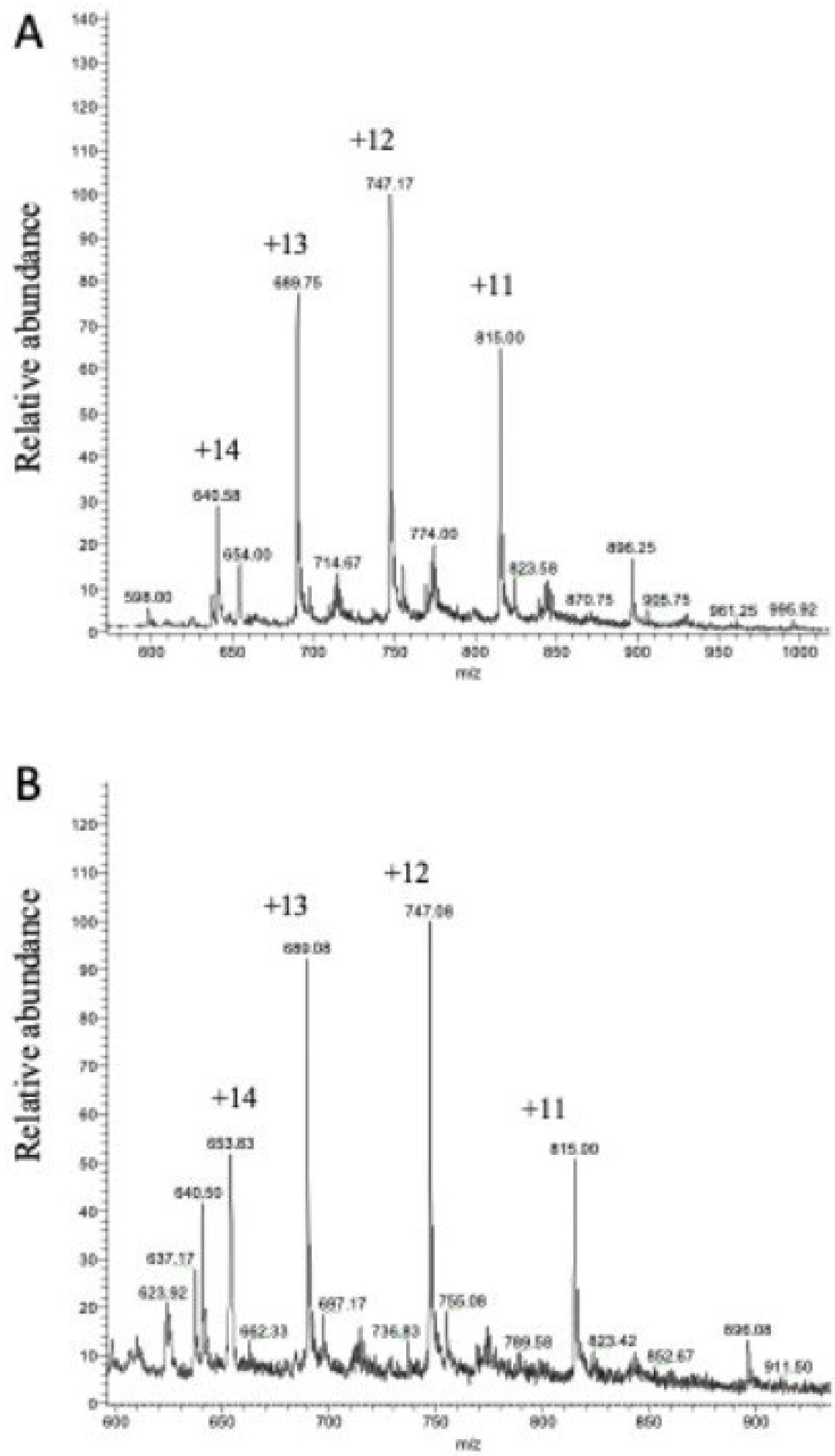

Supplement: Supplementary file 6 [file 1678-9199-jvatitd-32-e20260024-s6.pdf]
