# Supplementary material for: Proteomic characterization of Tityus championi venom and recombinant expression of its major neurotoxin
Source: J Venom Anim Toxins Incl Trop Dis. 2026 Sep 14;32:e20260024. doi: 10.1590/1678-9199-JVATITD-2026-0024 (PMC13574325; doi:10.1590/1678-9199-JVATITD-2026-0024)
Supplement: Supplementary file 8 [file 1678-9199-jvatitd-32-e20260024-s8.pdf]

J Venom Anim Toxins incl Trop Dis,
https://doi.org/10.1590/1678-9199-JVATITD-2026-0024

Supplementary Material to: Proteomic characterization of *Tityus championi* venom and recombinant expression of its major neurotoxin

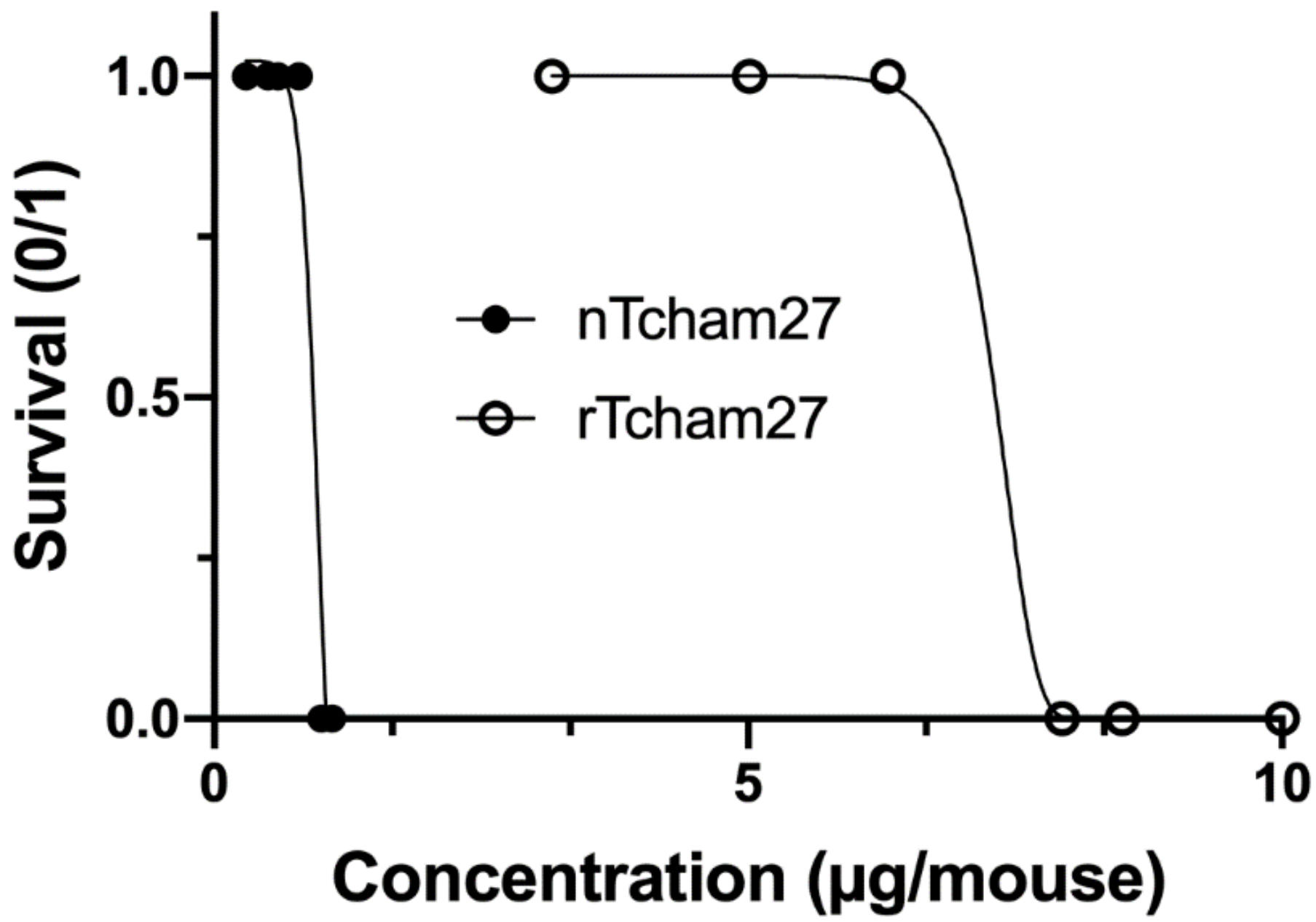

Additional file 8. Mouse survival following intracranial injection. Six doses of nTcham27 and rTcham27 were injected intracranially into mice. Data were analyzed using a nonlinear regression equation, yielding calculated intracranial LD_{50} values of 0.94 (0.19-1.02) and 7.3 (1.6-10.0) µg/mouse for nTcham27 and rTcham27, respectively.
